# Supplementary material for: Hydrothermal alteration of Ryugu from a disruptive impact recorded in a returned sample
Source: Nat Commun. 2025 Dec 9;17:466. doi: 10.1038/s41467-025-67159-9 (PMC12800196; doi:10.1038/s41467-025-67159-9)
Supplement: Supplementary file 1 — Supplementary Information [file 41467_2025_67159_MOESM1_ESM.pdf]

Supplementary Information

Hydrothermal alteration of Ryugu from a disruptive impact recorded in a returned sample

Devin L. Schrader, Thomas J. Zega, Maizey C. Benner, and Jemma Davidson

Part I.

Pages 2–95: Images and data point locations for Ryugu Samples: A0016.

Part II.

Pages 96–145: Images and data point locations for Ryugu Samples: A0094-01 and C0103-01.

Part III.

Pages 146–178: Sulfide Ternaries for Ryugu Samples: A0016, A0094-01, and C0103-01.

Part I

Images and data point locations for Ryugu Samples: A0016.

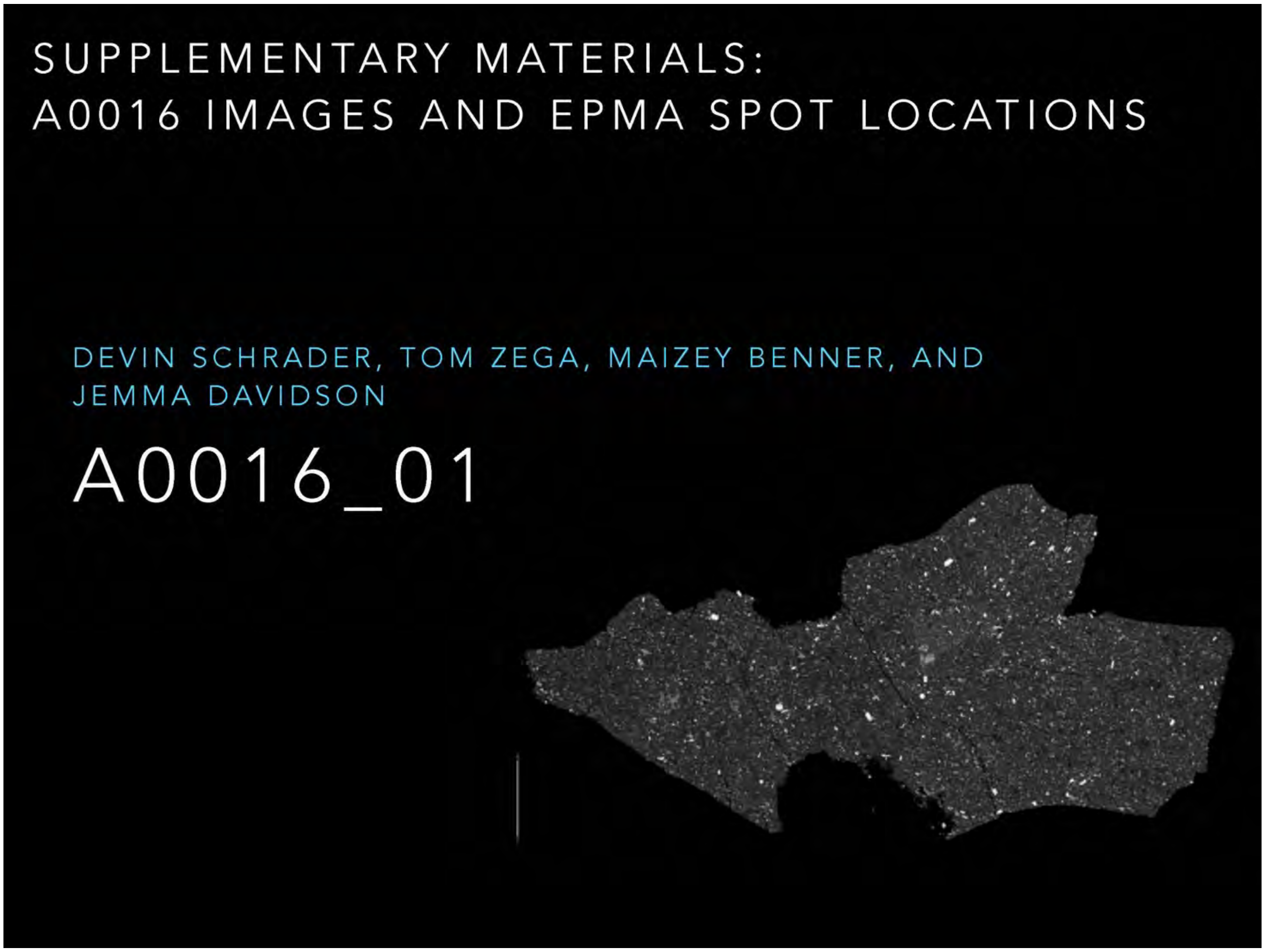

Supplementary Figure 1. Title slide for A0016 images and electron probe microanalyzer (EPMA) spot locations.

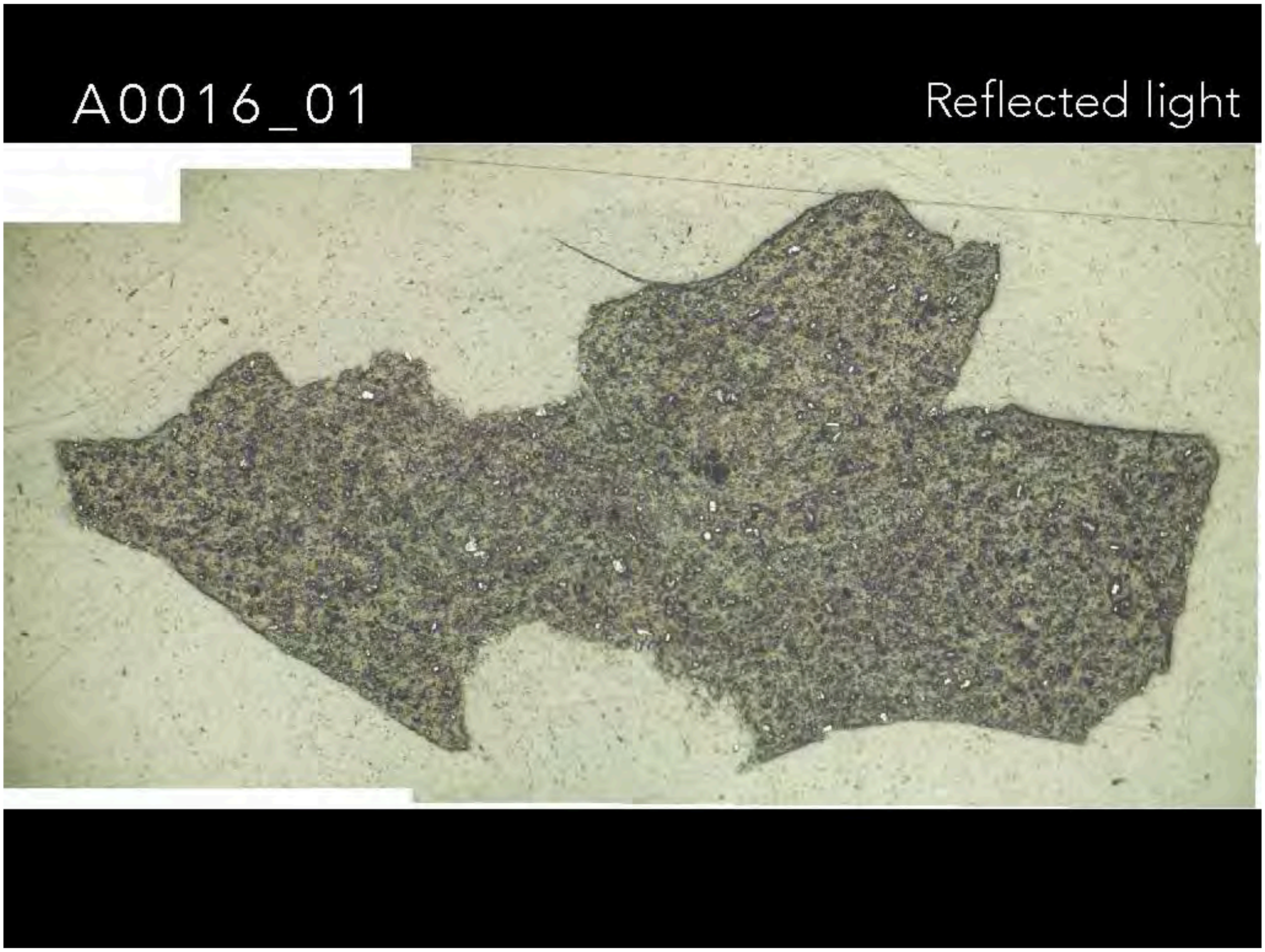

Supplementary Figure 2. Reflected light image of A0016.

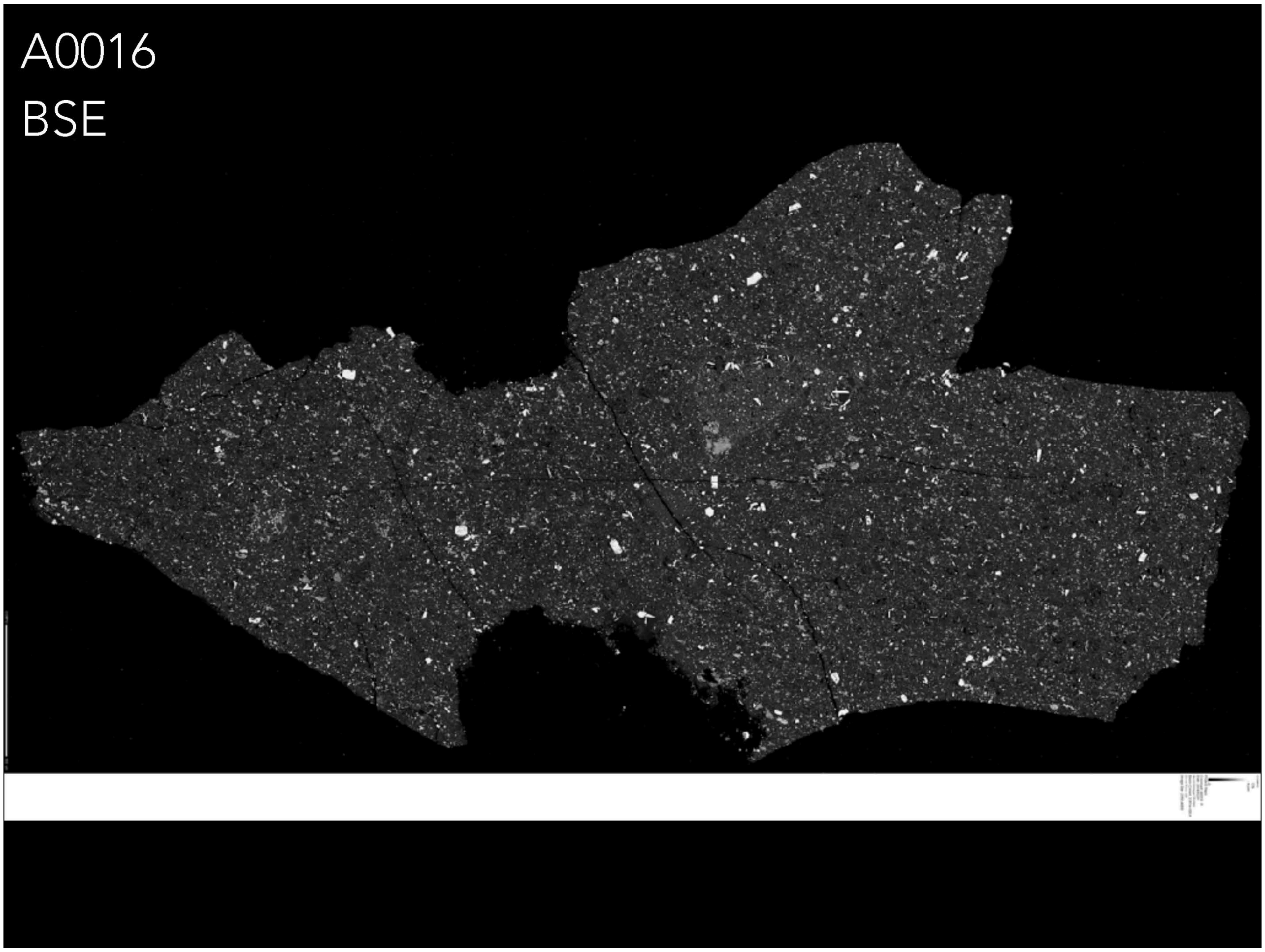

Supplementary Figure 3. Full backscattered electron (BSE) image of A0016.

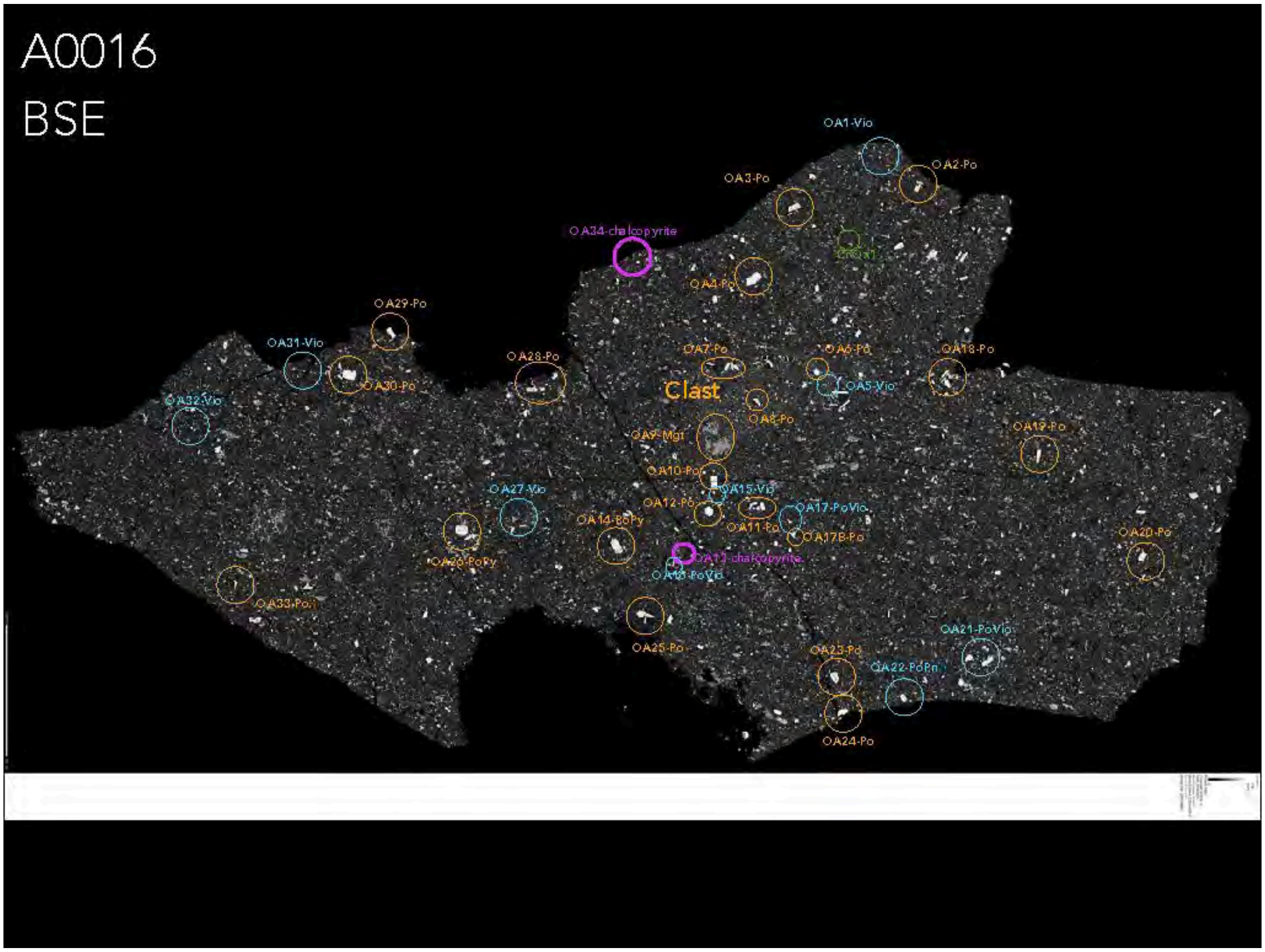

Supplementary Figure 4. Full backscattered electron (BSE) image of A0016 with location of all sulfides imaged in detail and analyzed marked. Where OA = opaque assemblage, Po = pyrrhotite, Pn = pentlandite, Vio = violarite, Py = pyrite, Mgt = magnetite, and CrOx = Cr-bearing oxide.

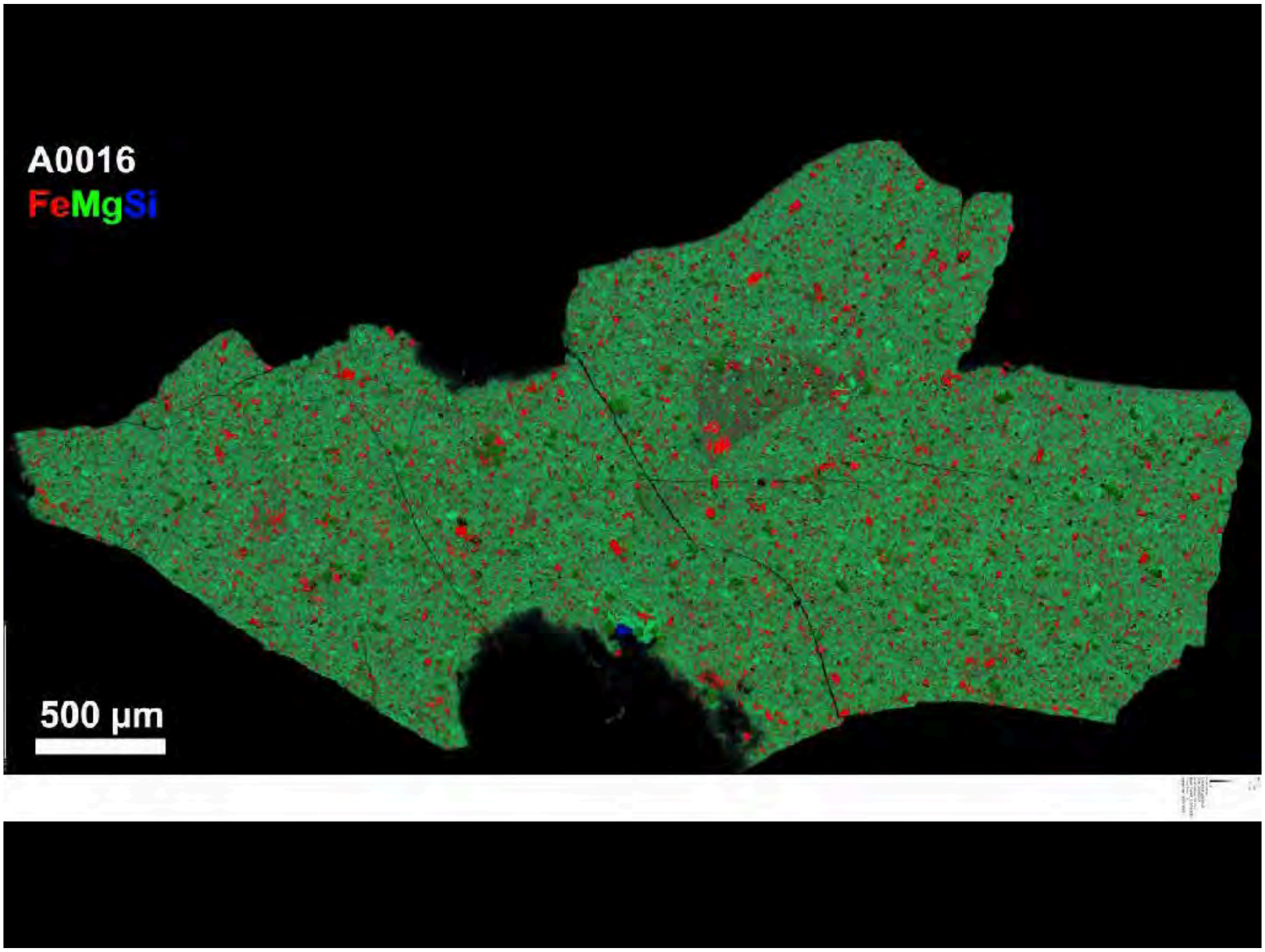

Supplementary Figure 5. Full composite X-ray element RGB image (Fe = red, Mg = green, and Si = blue) of A0016.

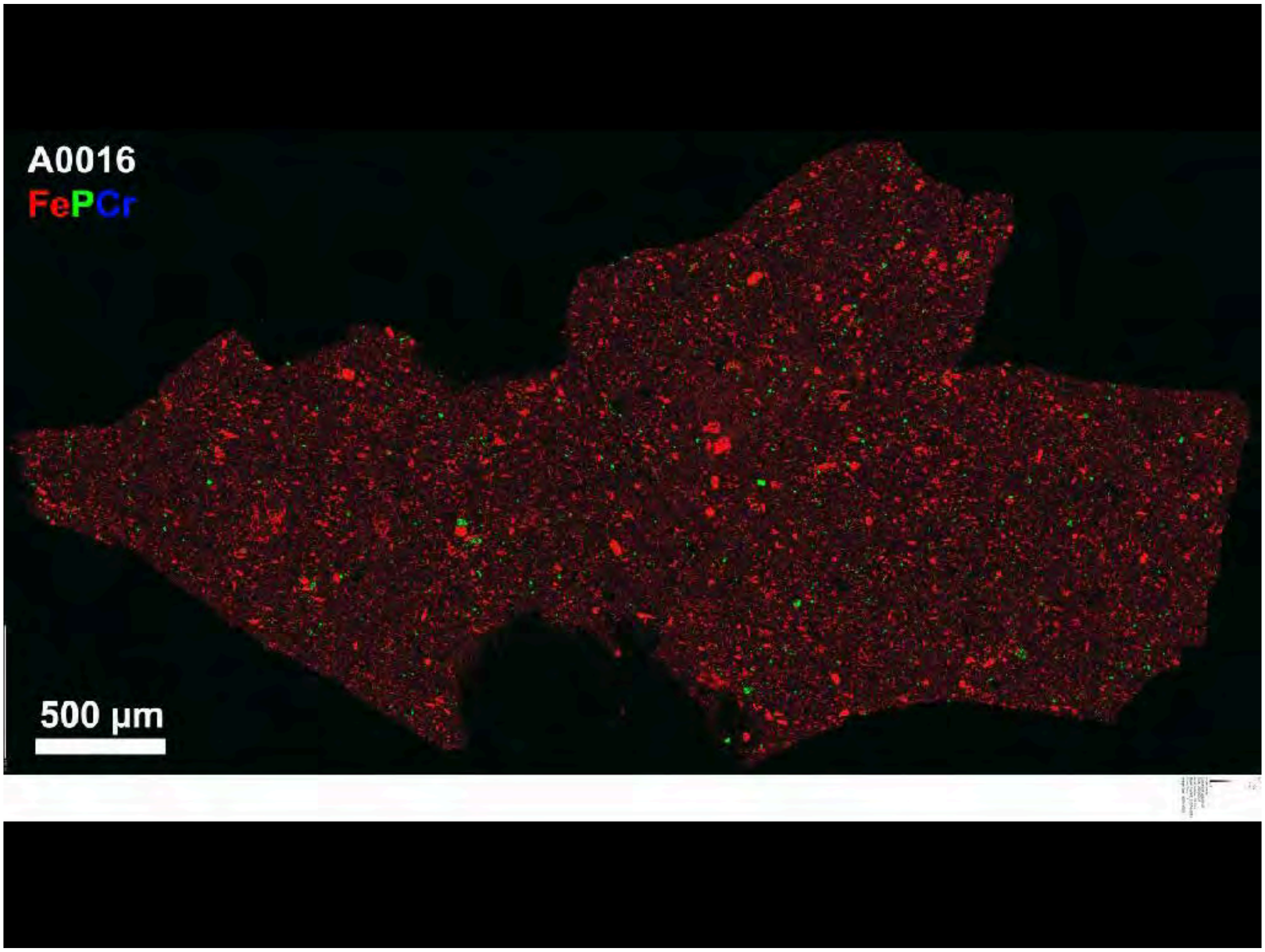

Supplementary Figure 6. Full composite X-ray element RGB image (Fe = red, P = green, and Cr = blue) of A0016.

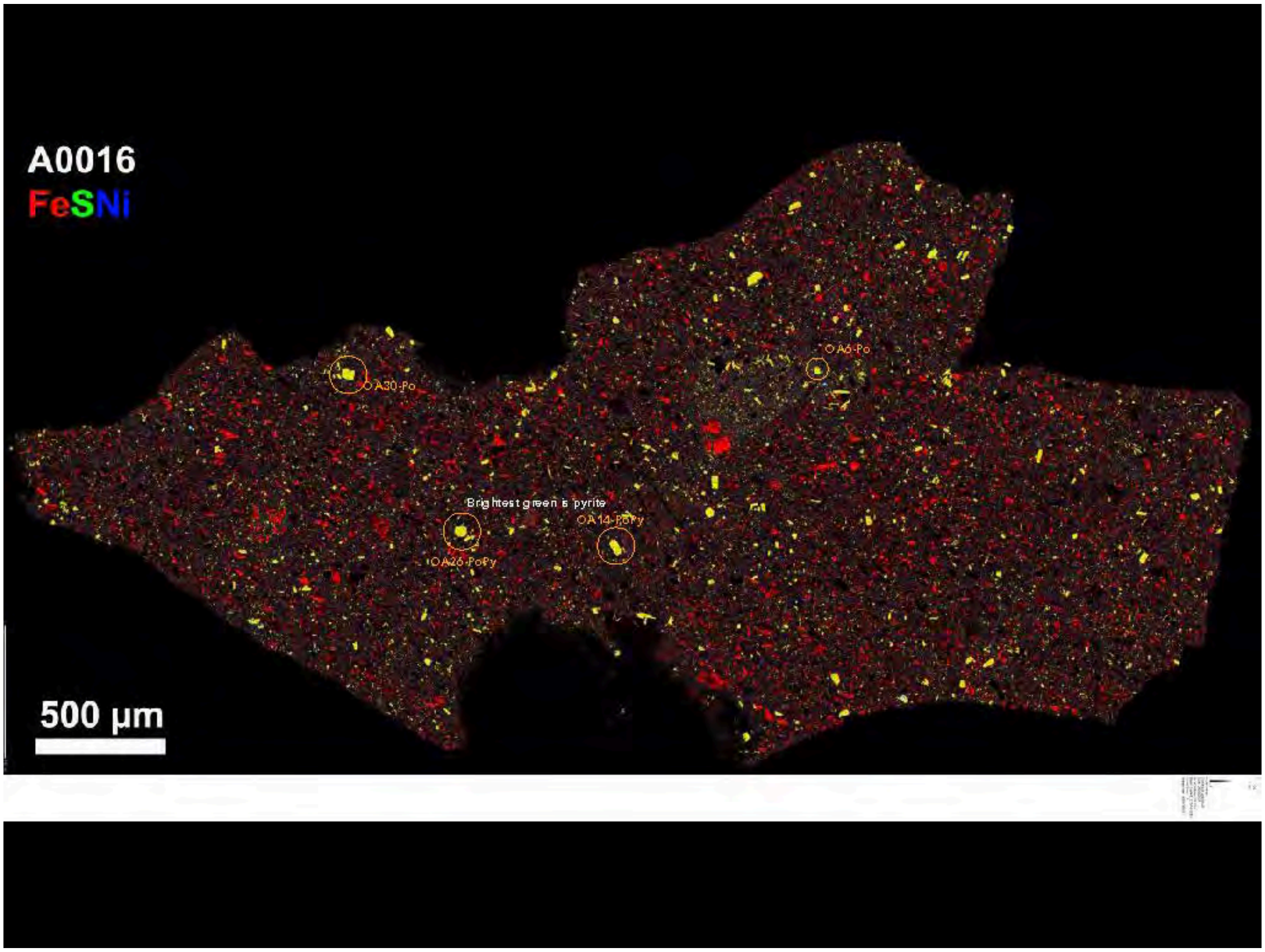

Supplementary Figure 7. Full composite X-ray element RGB image (Fe = red, S = green, and Ni = blue) of A0016.

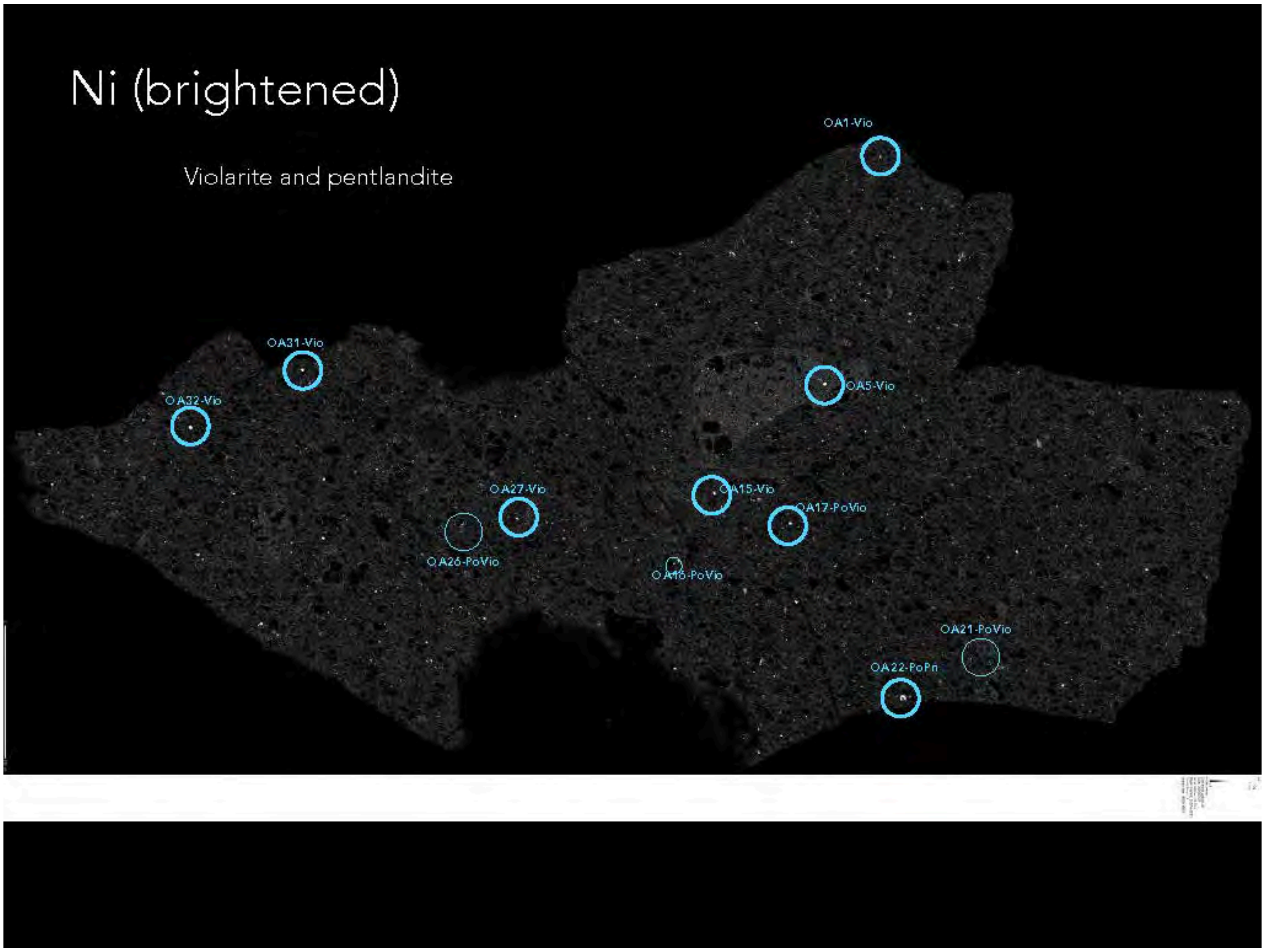

Supplementary Figure 8. Ni X-ray element map of A0016, with Ni-bearing sulfides analyzed marked. Where OA = opaque assemblage, Po = pyrrhotite, Pn = pentlandite, and Vio = violarite.

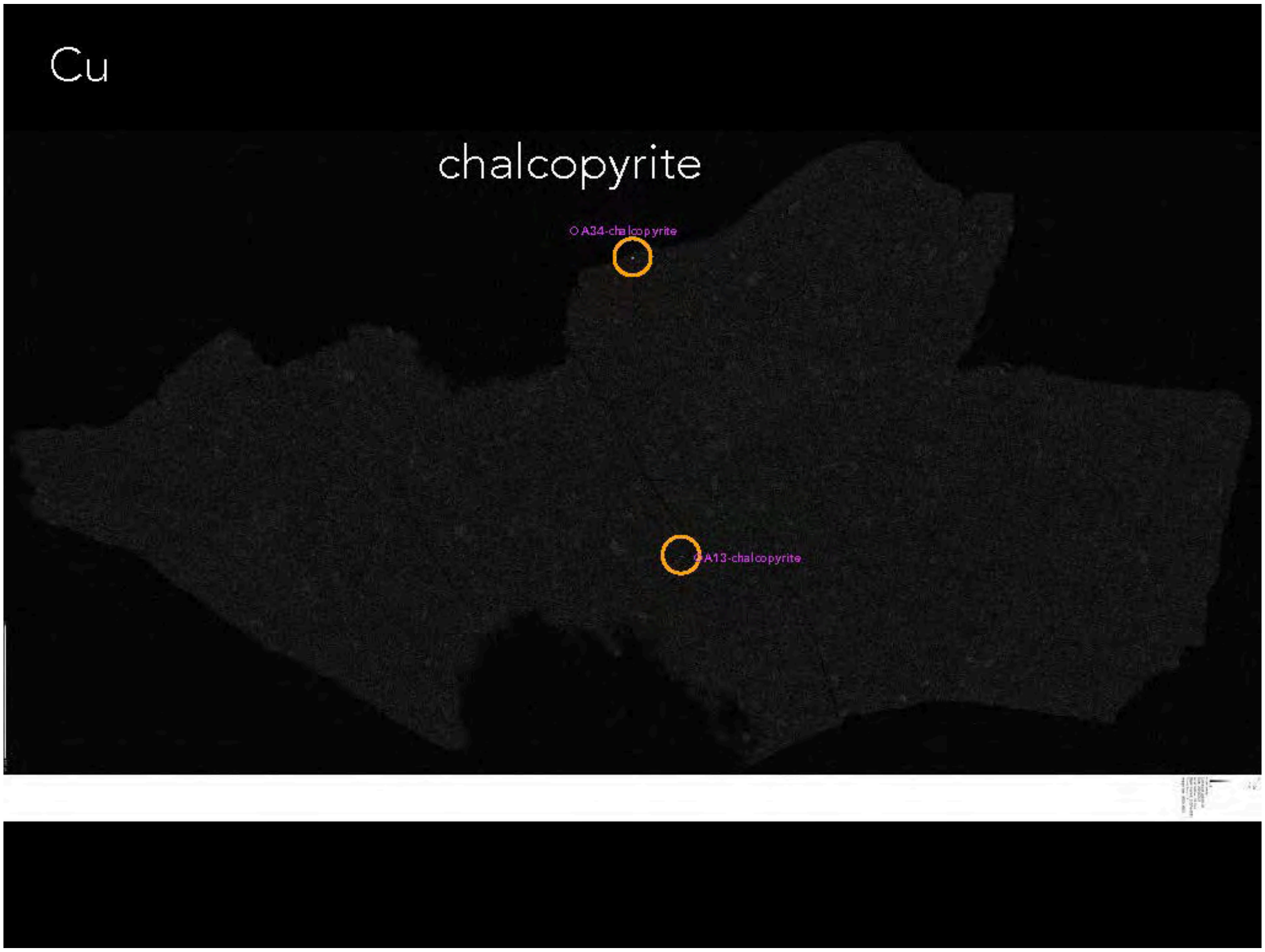

Supplementary Figure 9. Cu X-ray element map of A0016, with the two grains of chalcopyrite marked. Where OA = opaque assemblage.

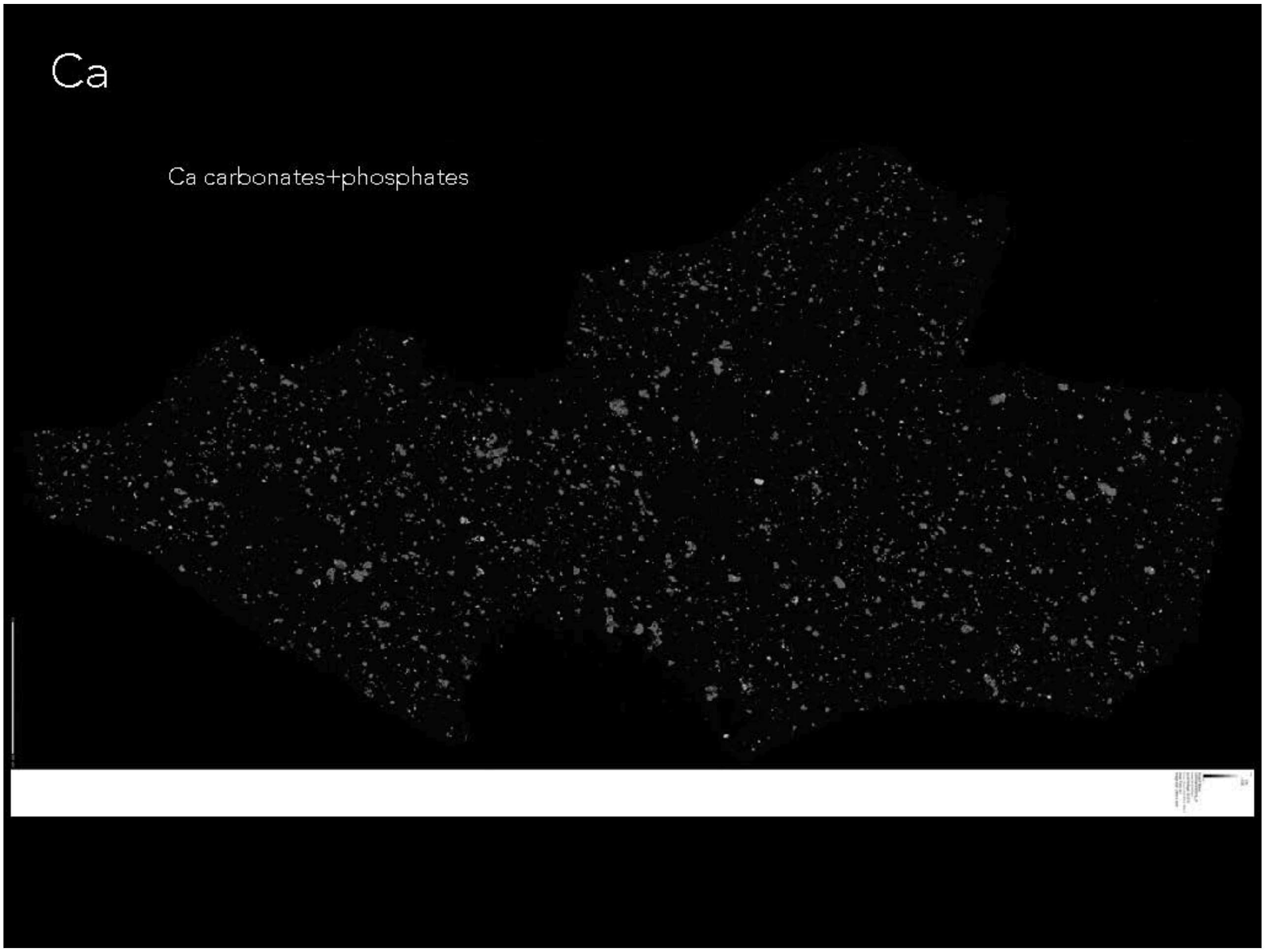

Supplementary Figure 10. Ca X-ray element map of A0016, showing location of Ca-carbonates and phosphates.

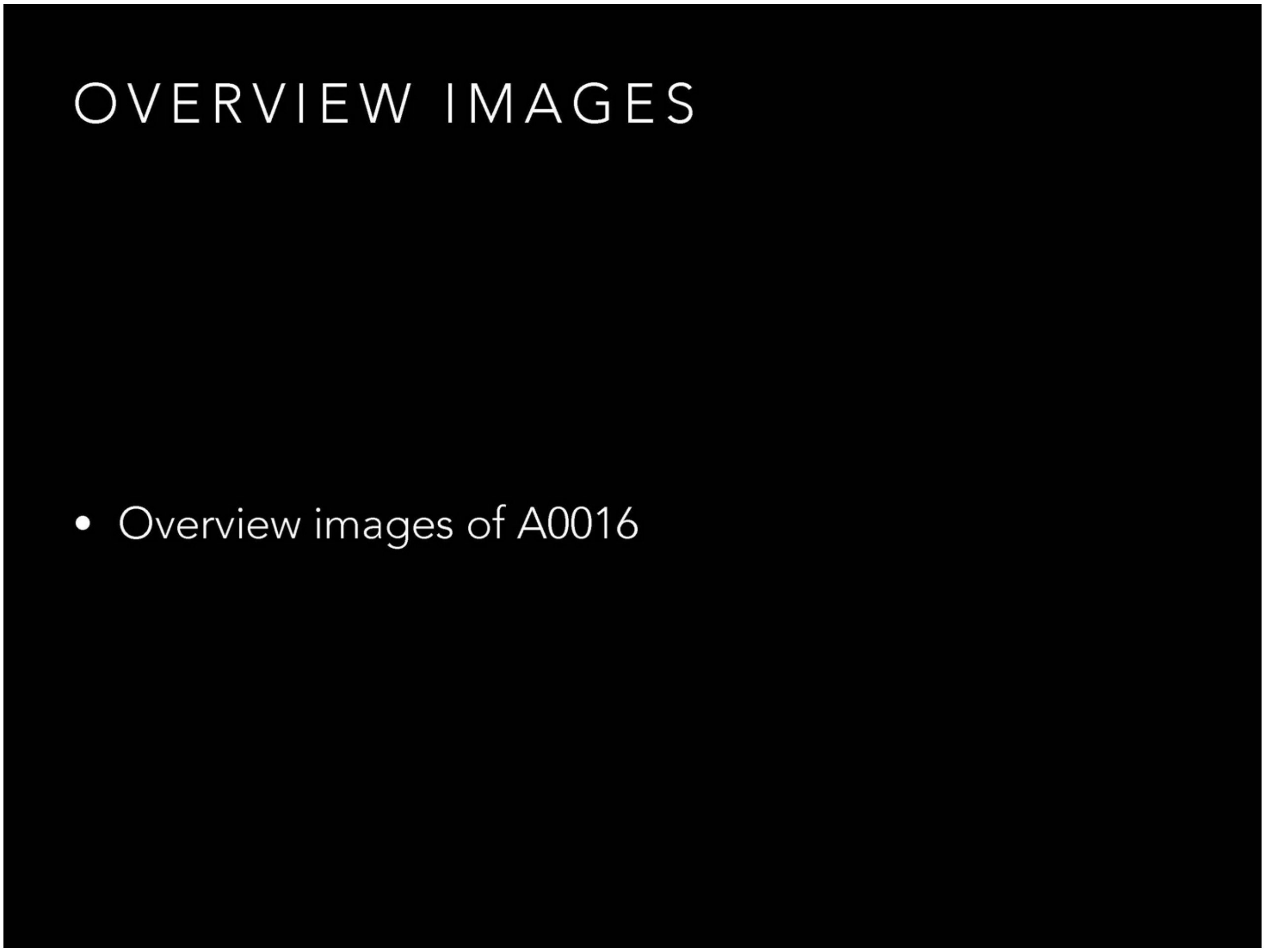

Supplementary Figure 11. Title slide for overview (low-magnification) images of A0016.

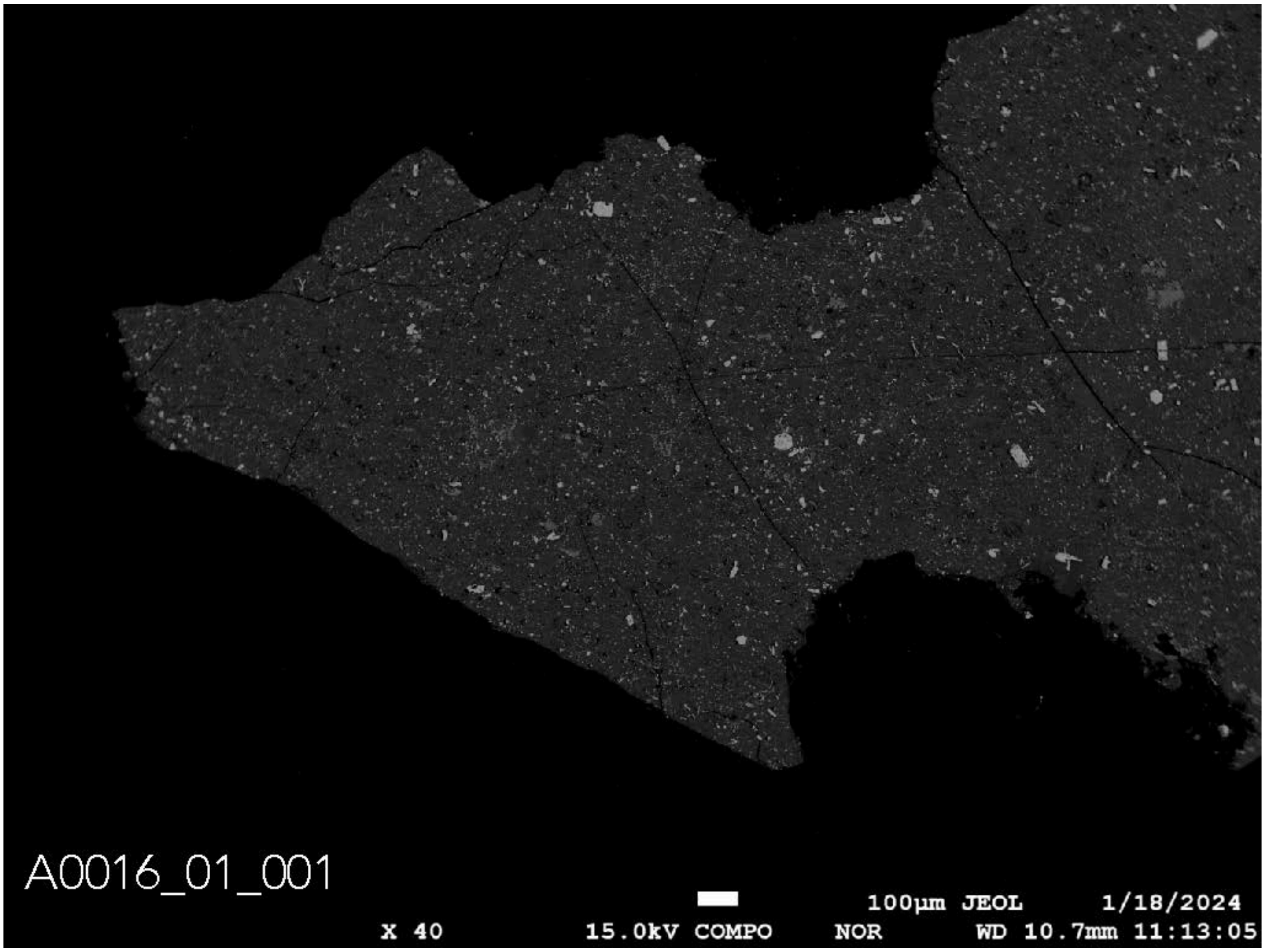

Supplementary Figure 12. Backscattered electron (BSE) image of region of A0016.

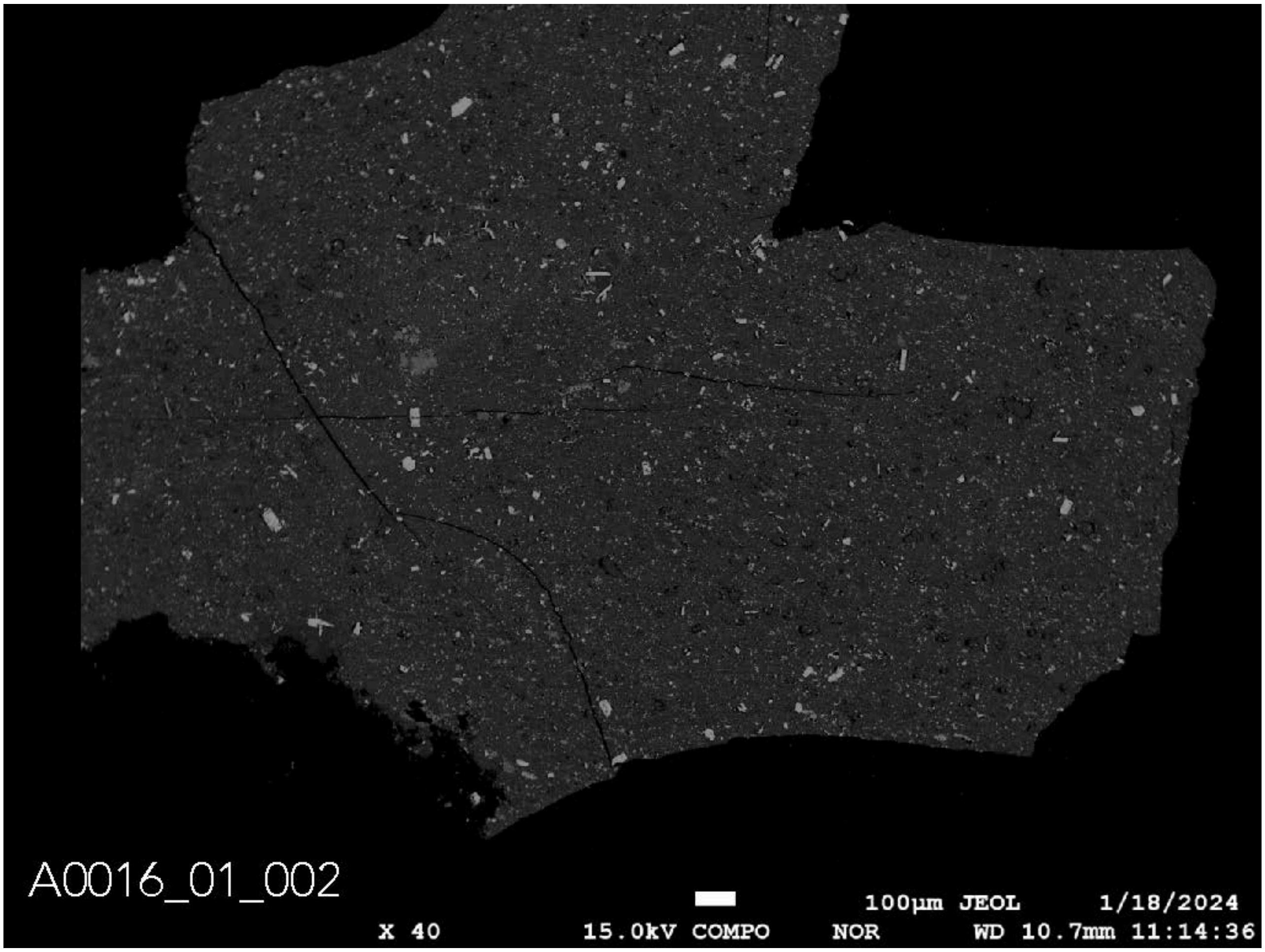

Supplementary Figure 13. Backscattered electron (BSE) image of region of A0016.

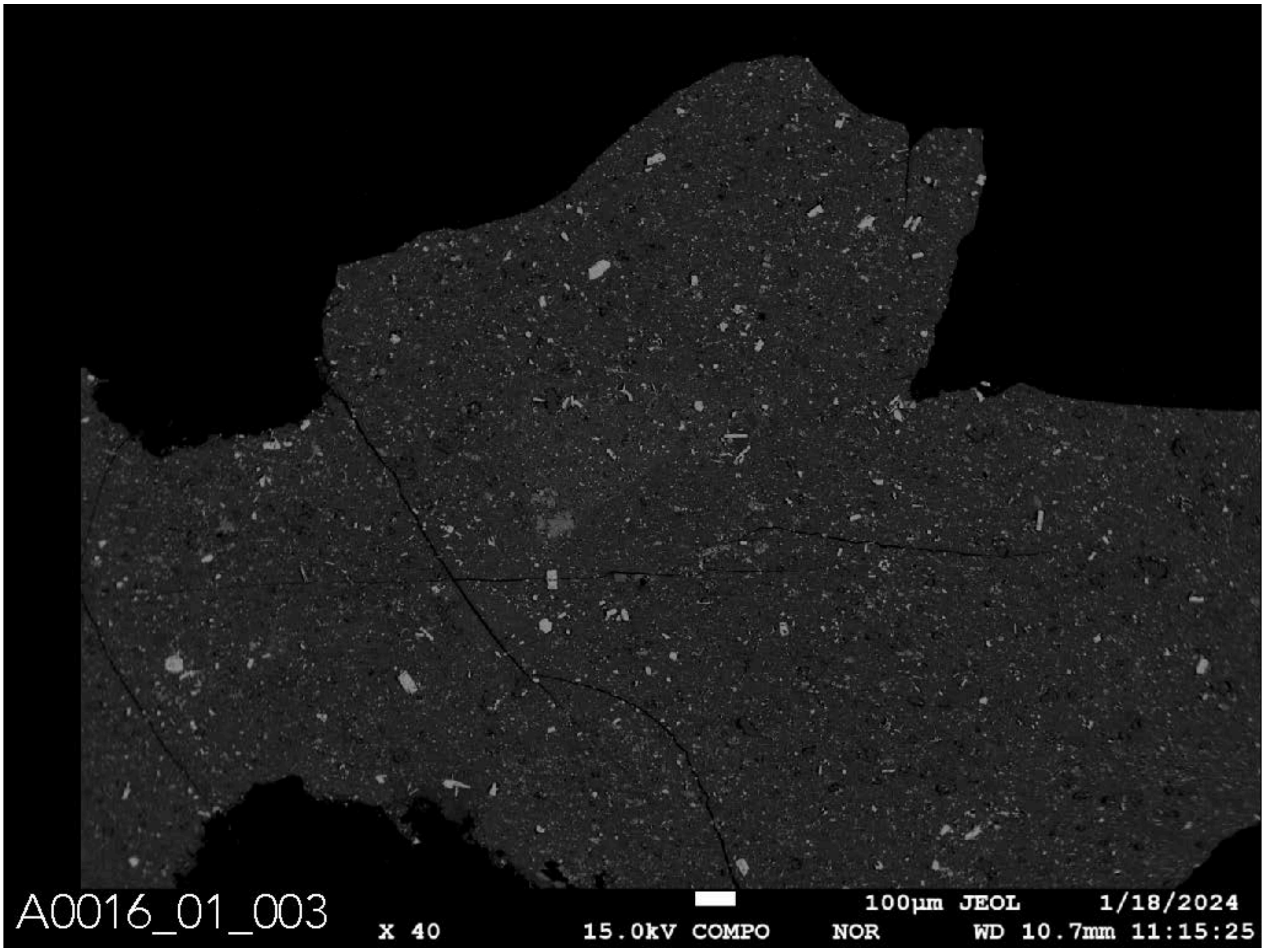

Supplementary Figure 14. Backscattered electron (BSE) image of region of A0016.

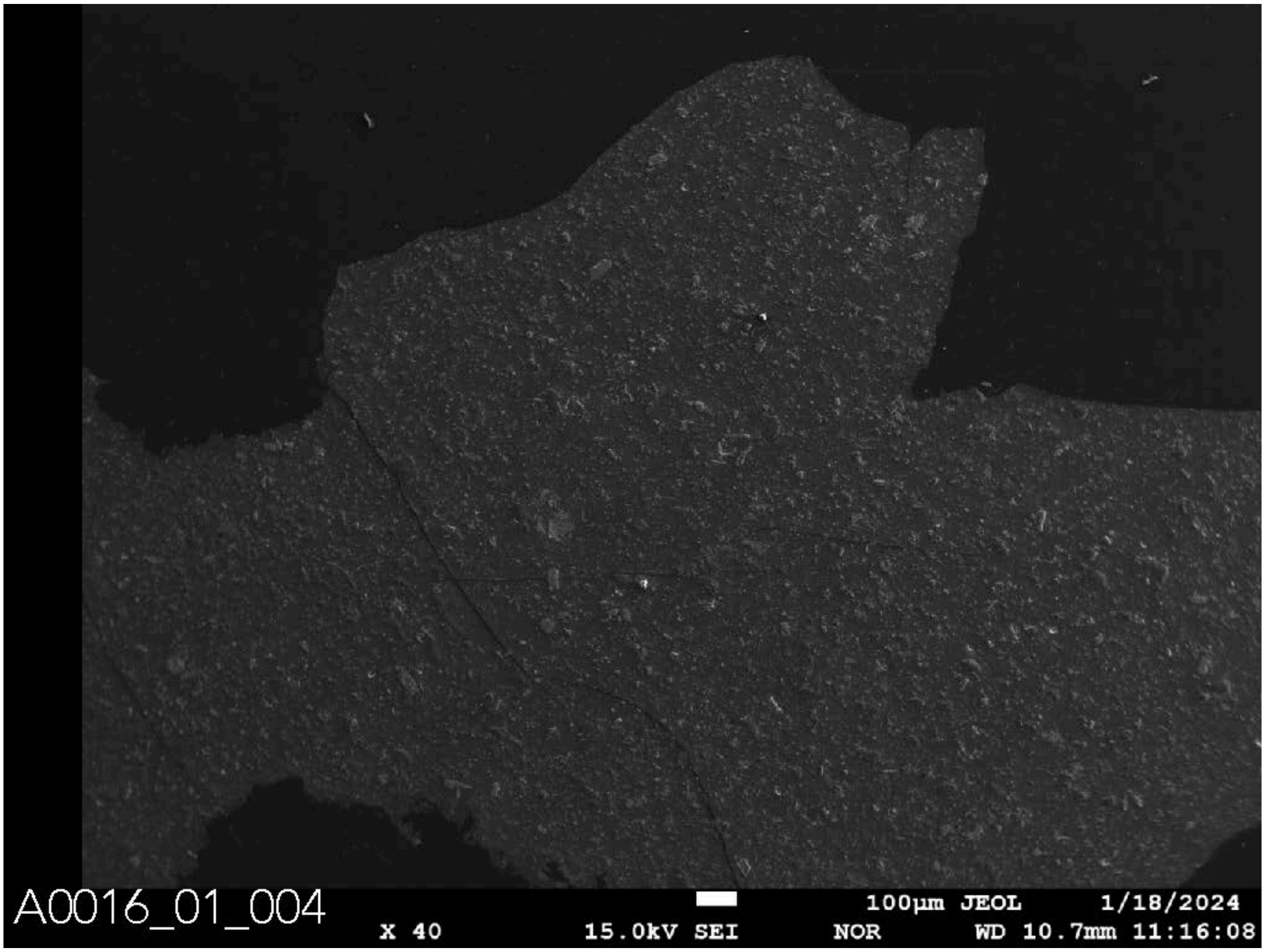

Supplementary Figure 15. Secondary electron (SE) image of region of A0016.

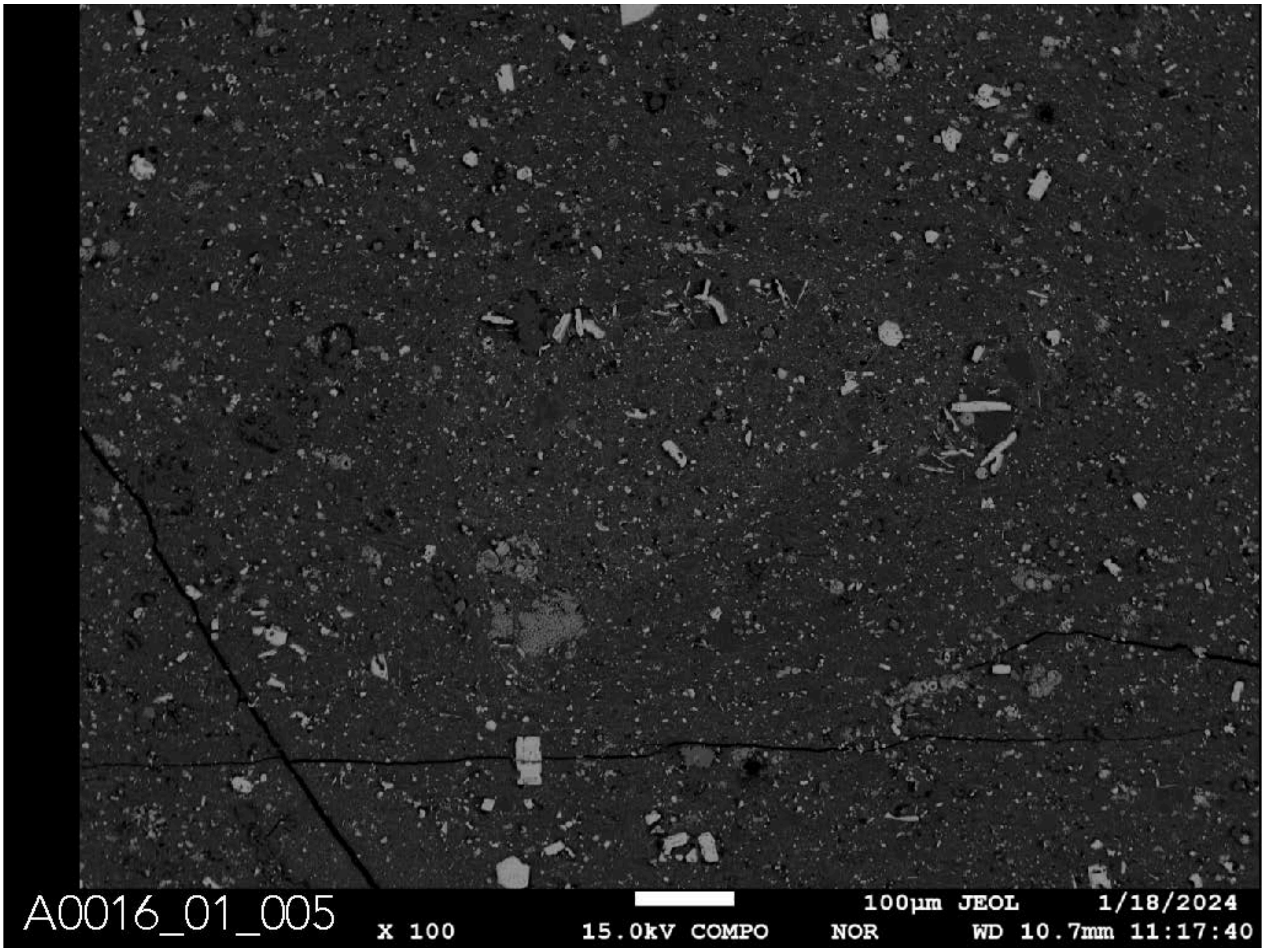

Supplementary Figure 16. Backscattered electron (BSE) image of region of A0016.

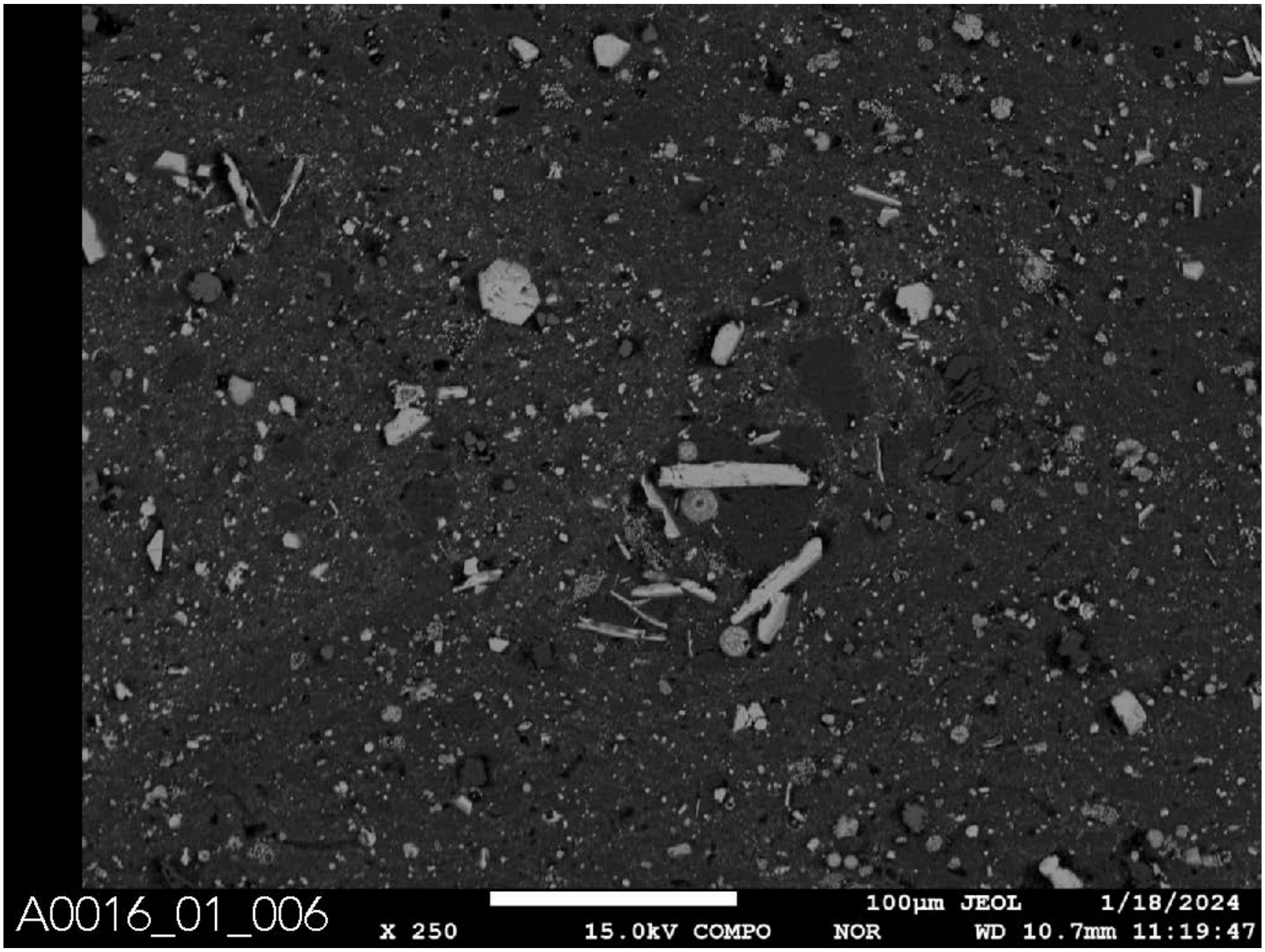

Supplementary Figure 17. Backscattered electron (BSE) image of region of A0016.

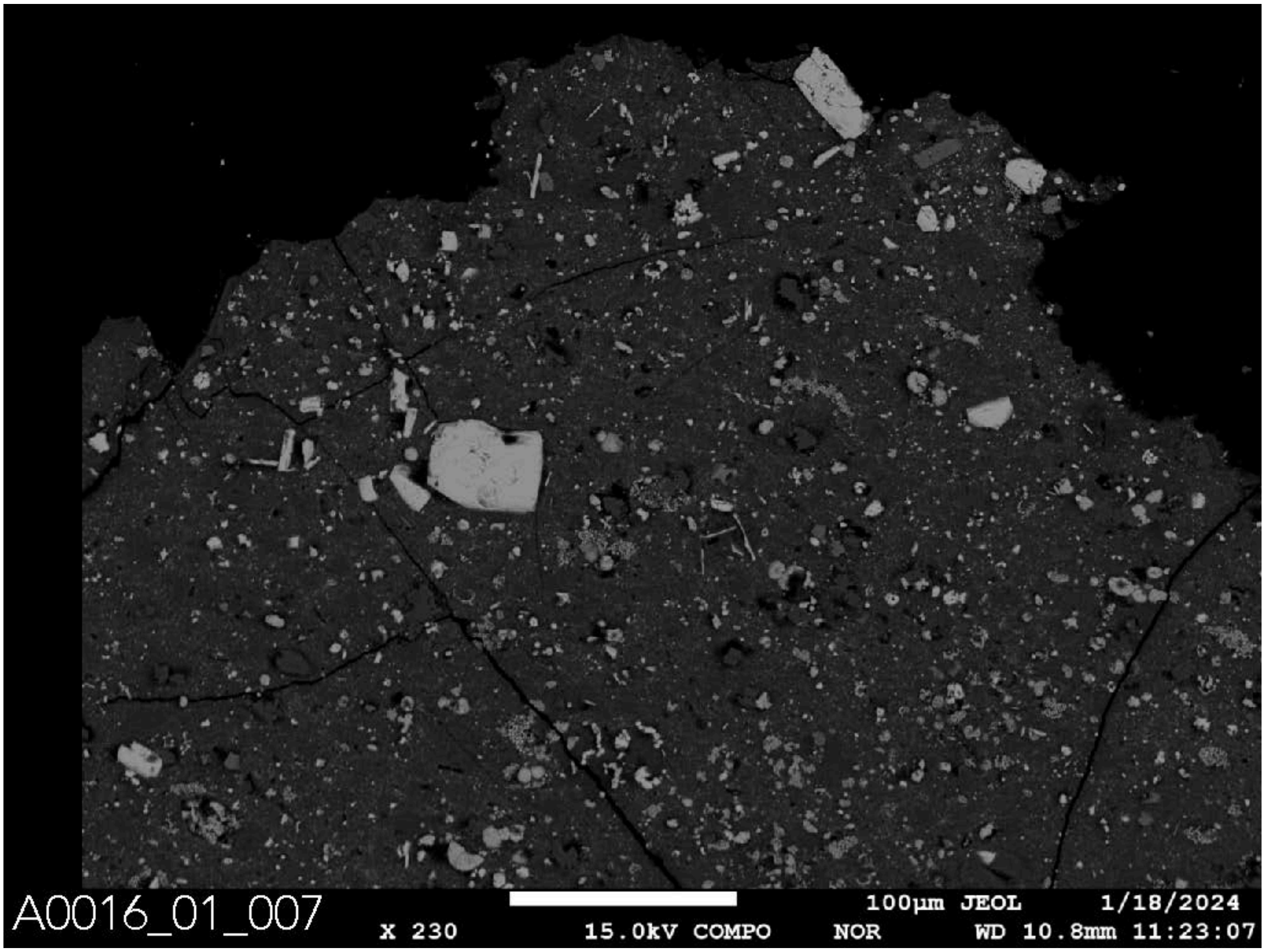

Supplementary Figure 18. Backscattered electron (BSE) image of region of A0016.

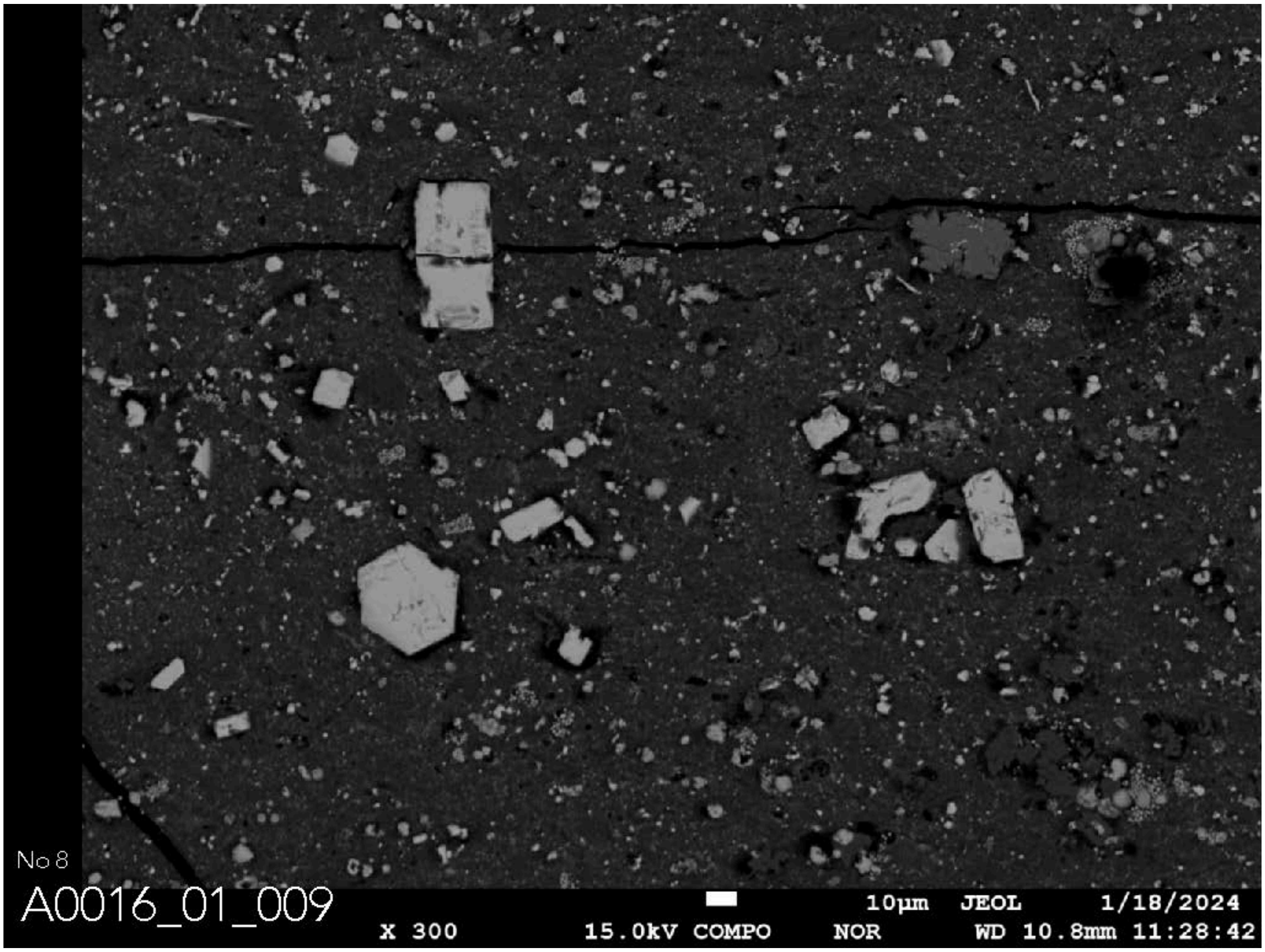

Supplementary Figure 19. Backscattered electron (BSE) image of region of A0016.

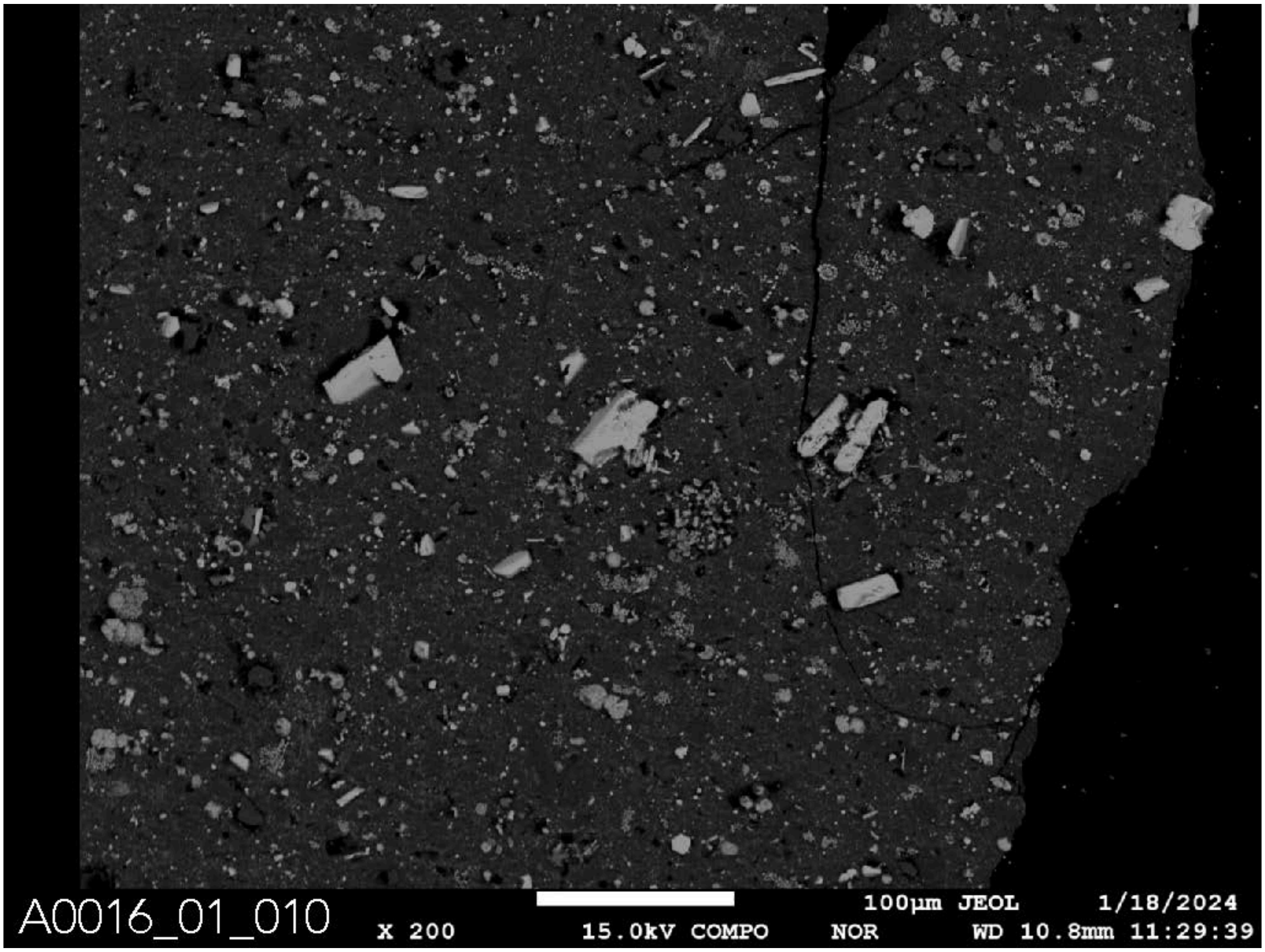

Supplementary Figure 20. Backscattered electron (BSE) image of region of A0016.

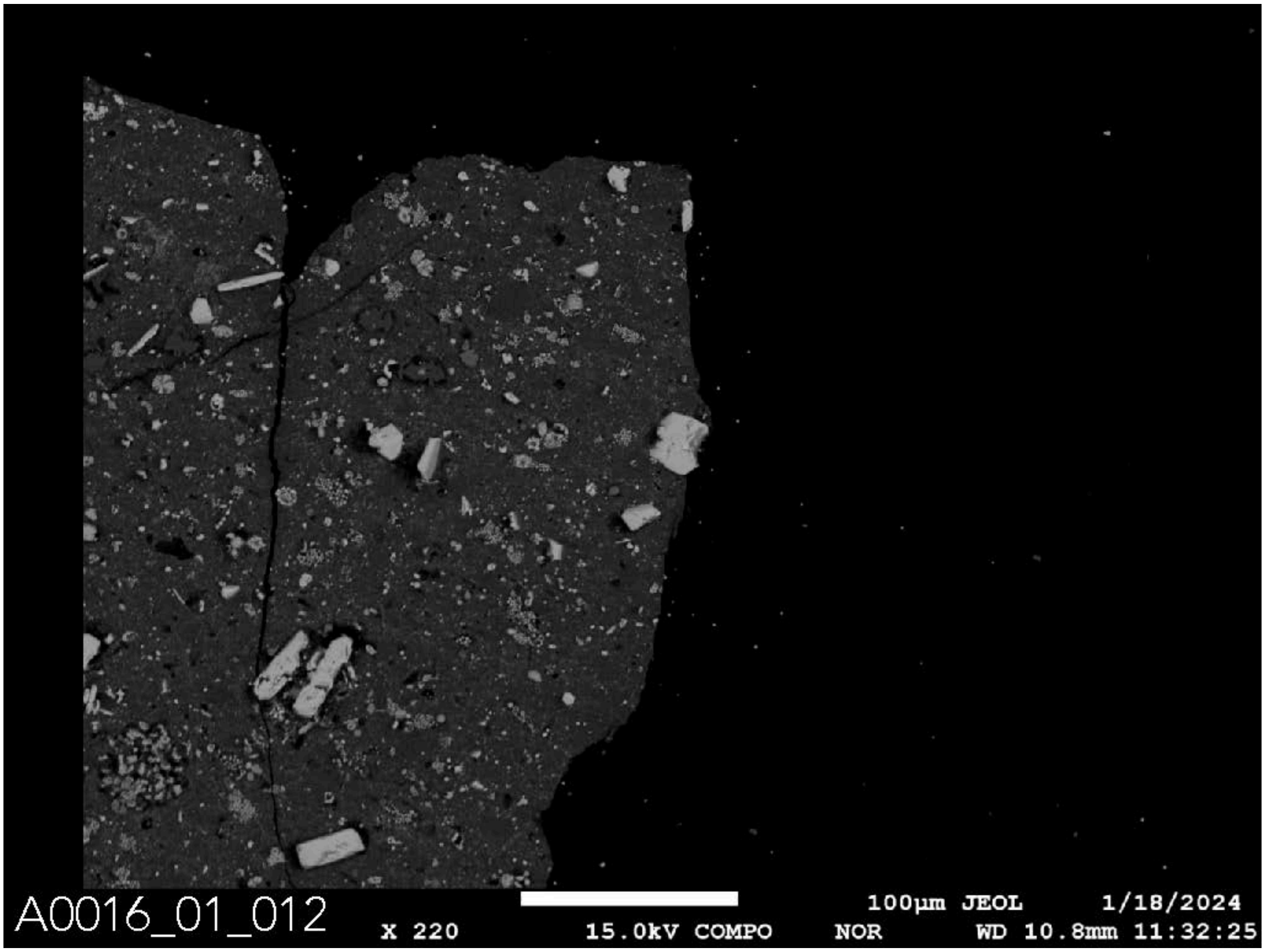

Supplementary Figure 21. Backscattered electron (BSE) image of region of A0016.

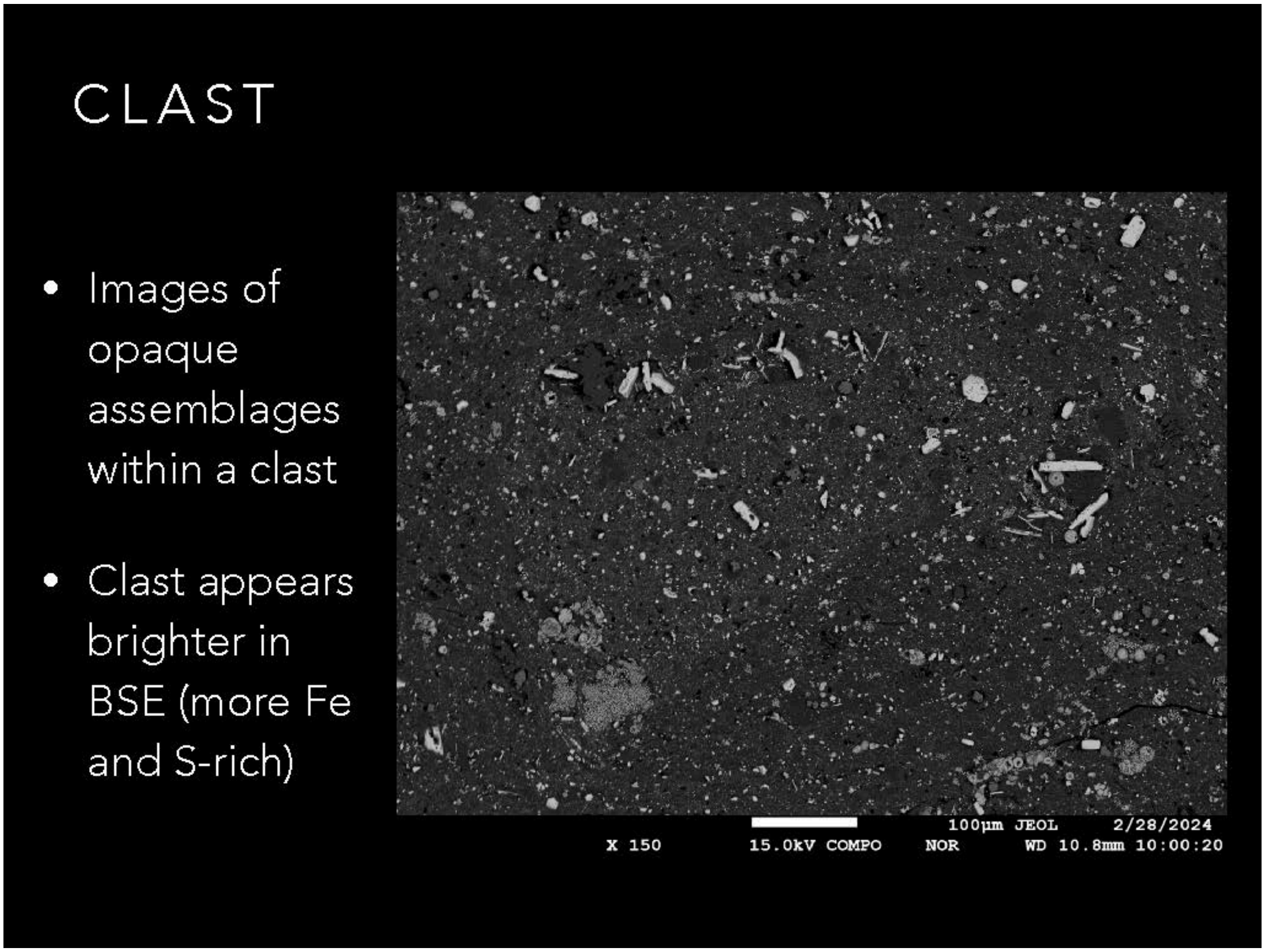

Supplementary Figure 22. Title slide for clast in A0016.

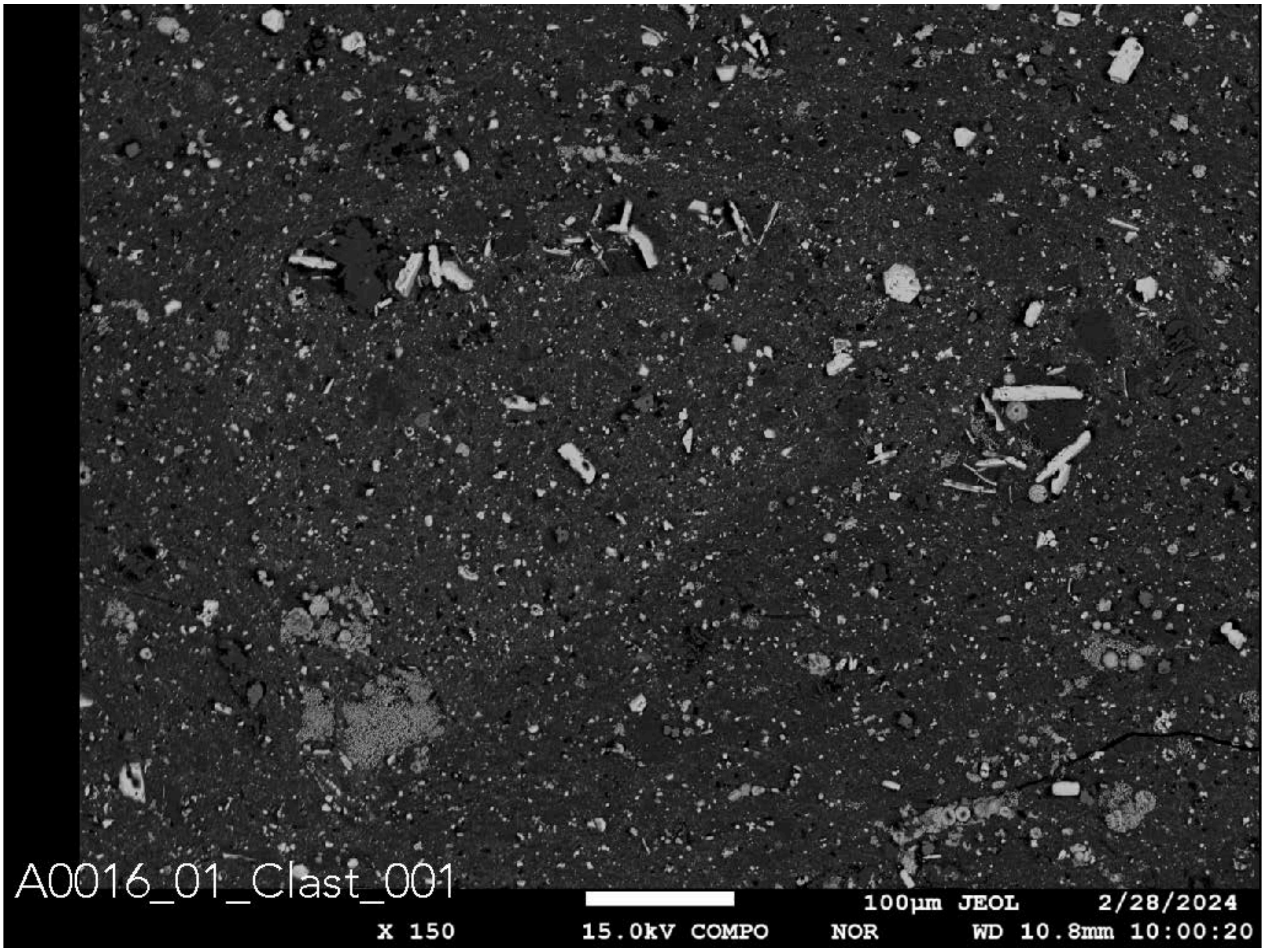

Supplementary Figure 23. Backscattered electron (BSE) image of clast region in A0016.

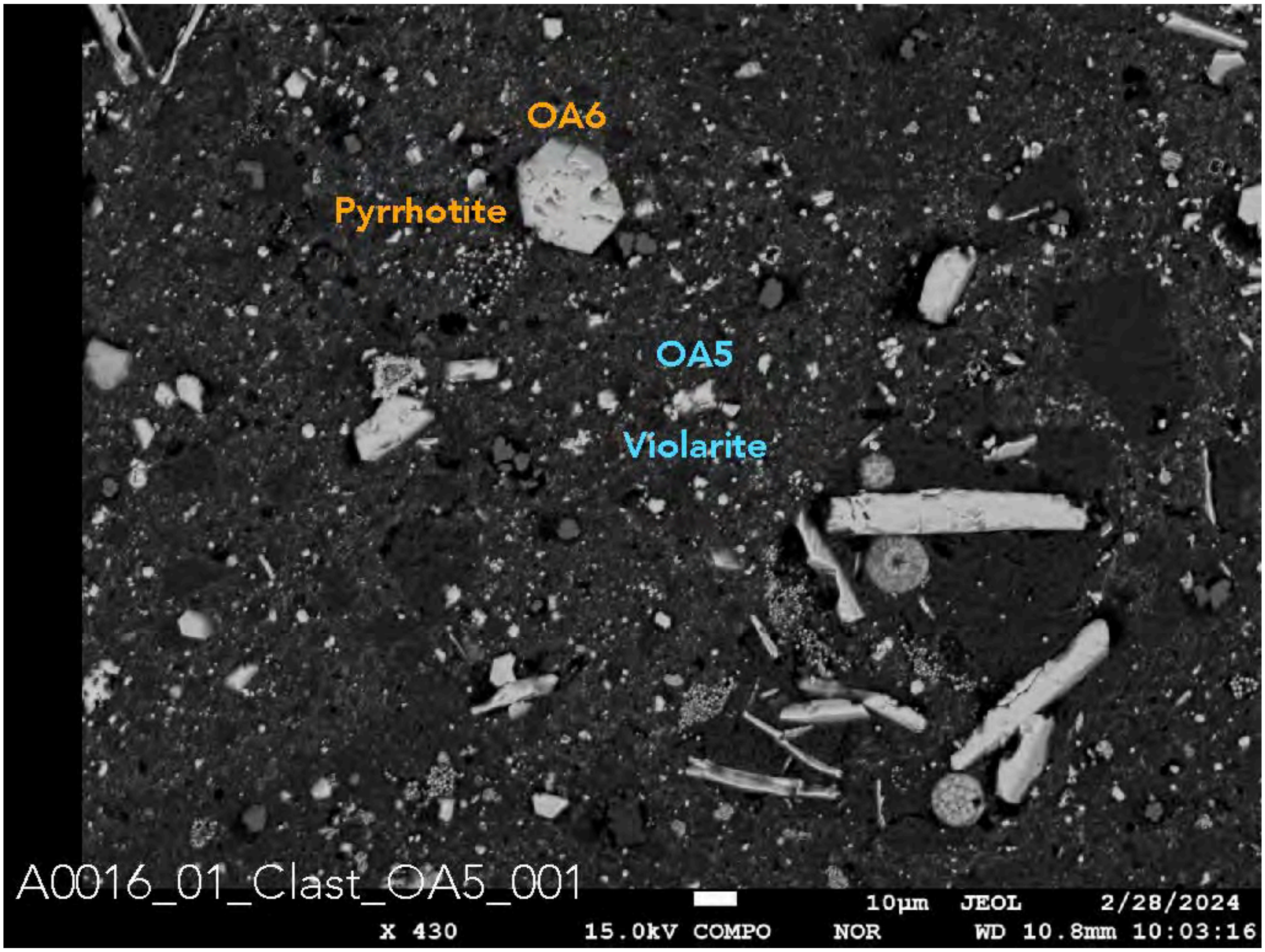

Supplementary Figure 24. Backscattered electron (BSE) image of clast region in A0016 showing OA5 and OA6. OA = opaque assemblage.

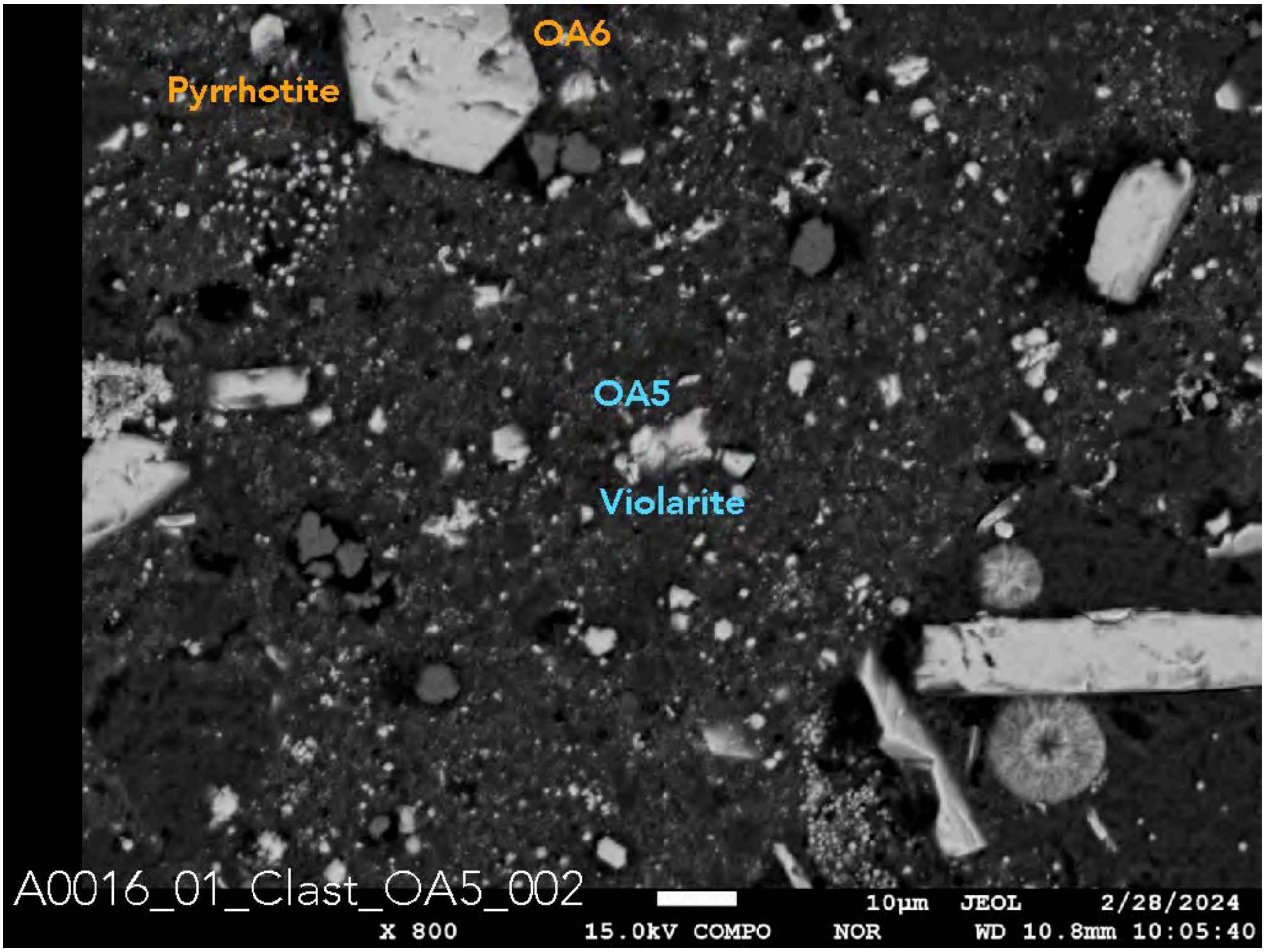

Supplementary Figure 25. Backscattered electron (BSE) image of clast region in A0016 showing OA5 and OA6. OA = opaque assemblage.

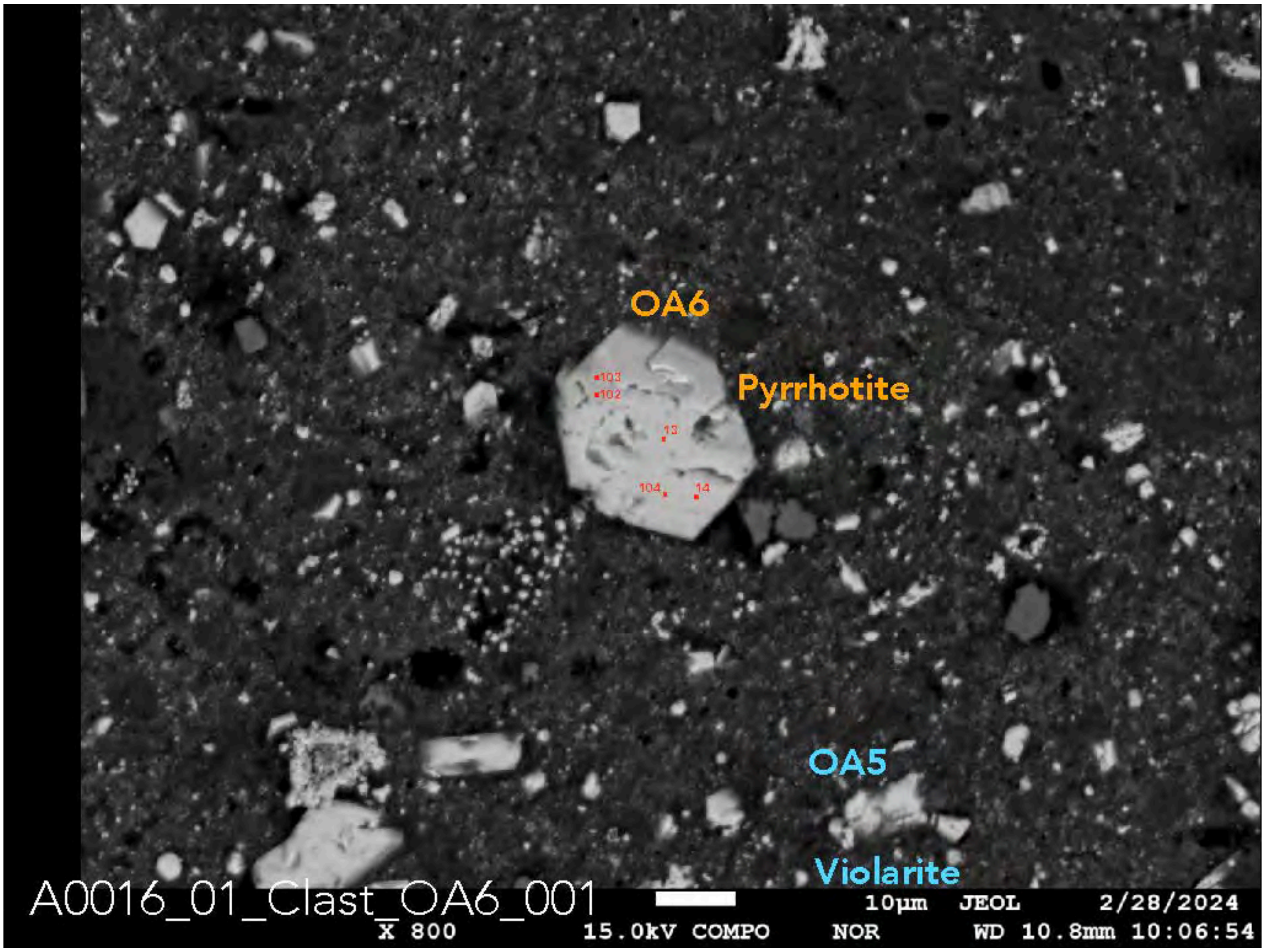

Supplementary Figure 26. Backscattered electron (BSE) image of clast region in A0016 showing OA6, with spot analyses marked. OA = opaque assemblage.

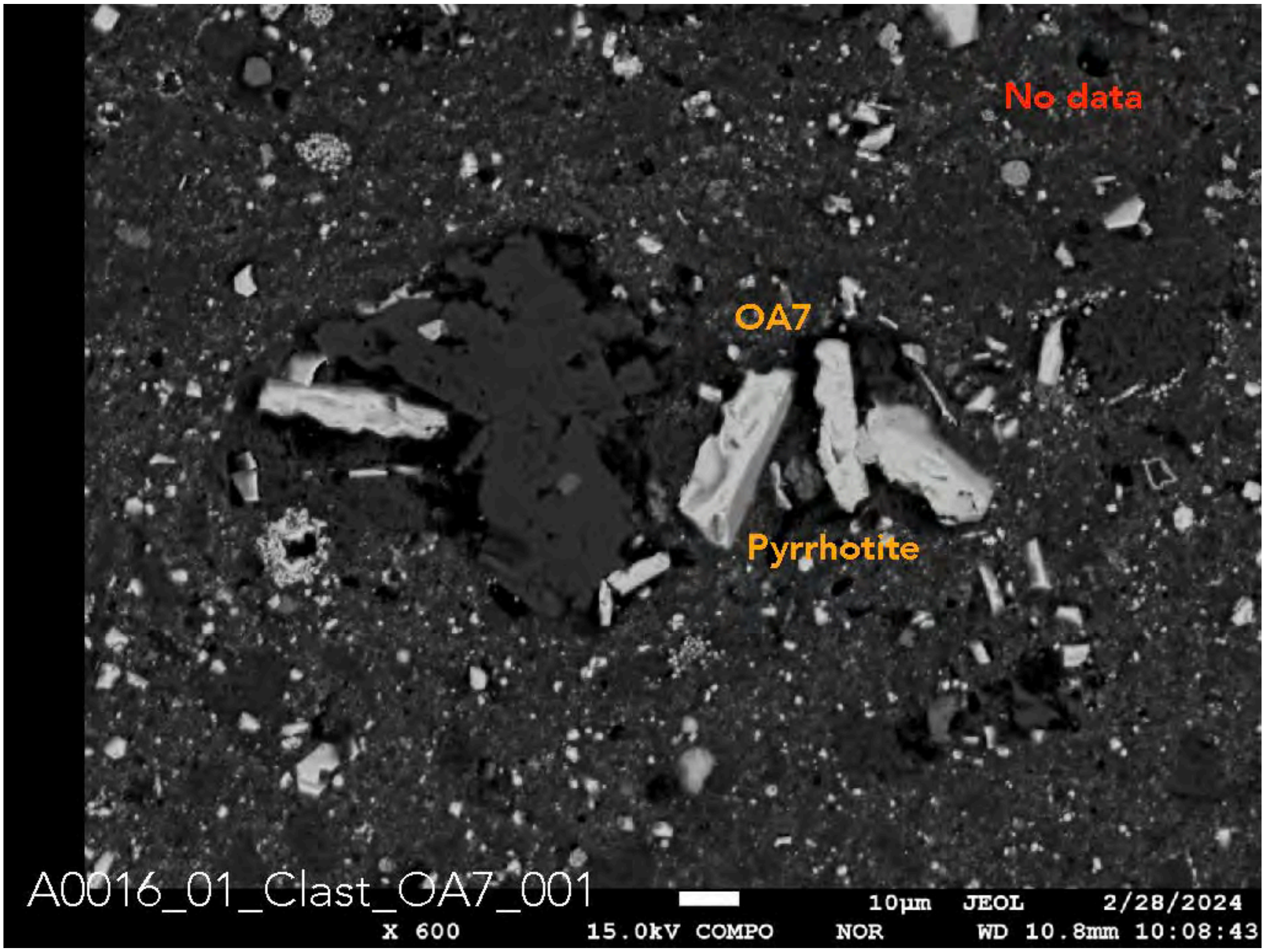

Supplementary Figure 27. Backscattered electron (BSE) image of clast region in A0016, showing OA7. OA = opaque assemblage.

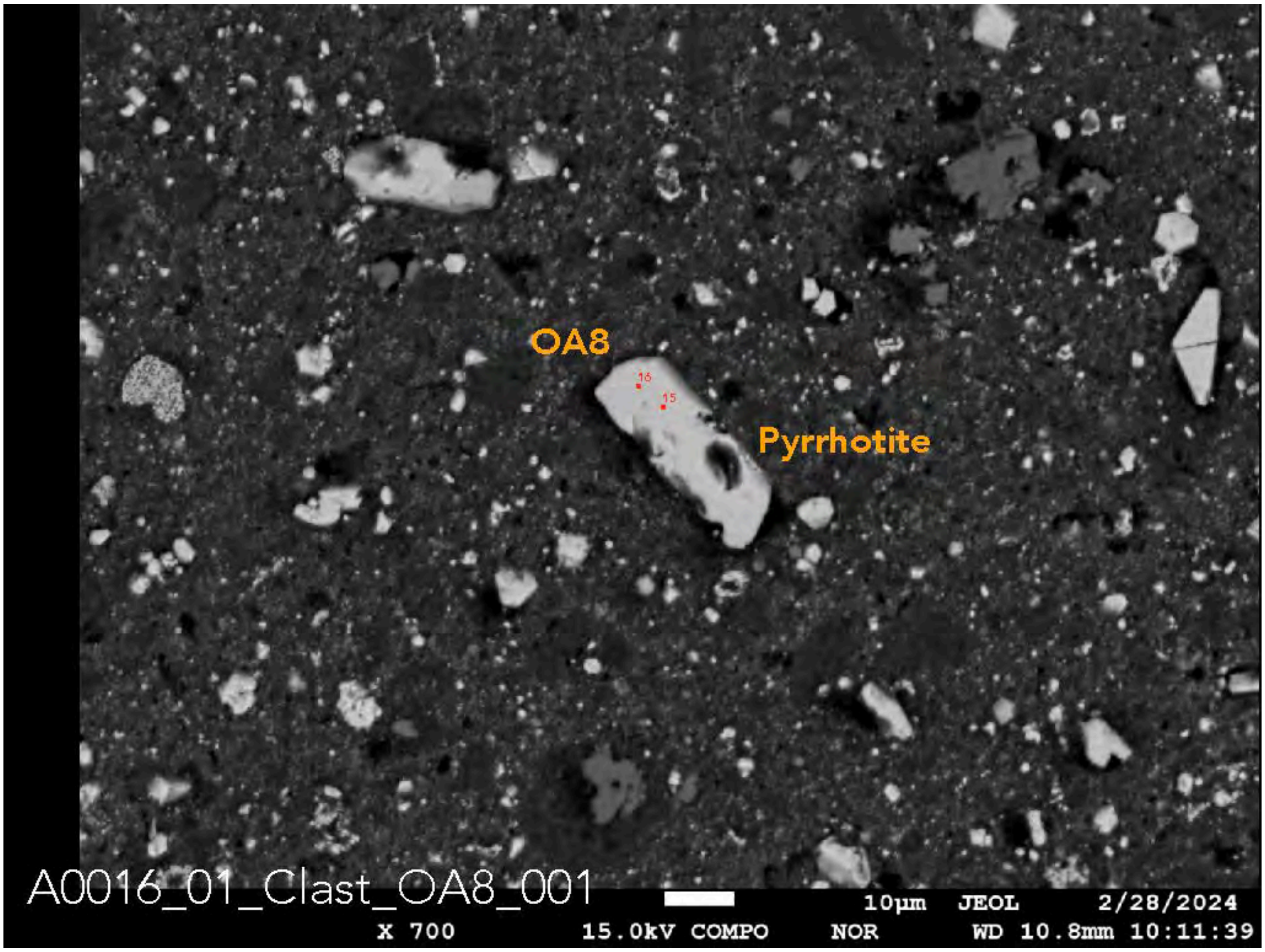

Supplementary Figure 28. Backscattered electron (BSE) image of clast region in A0016 showing OA8, with spot analyses marked. OA = opaque assemblage.

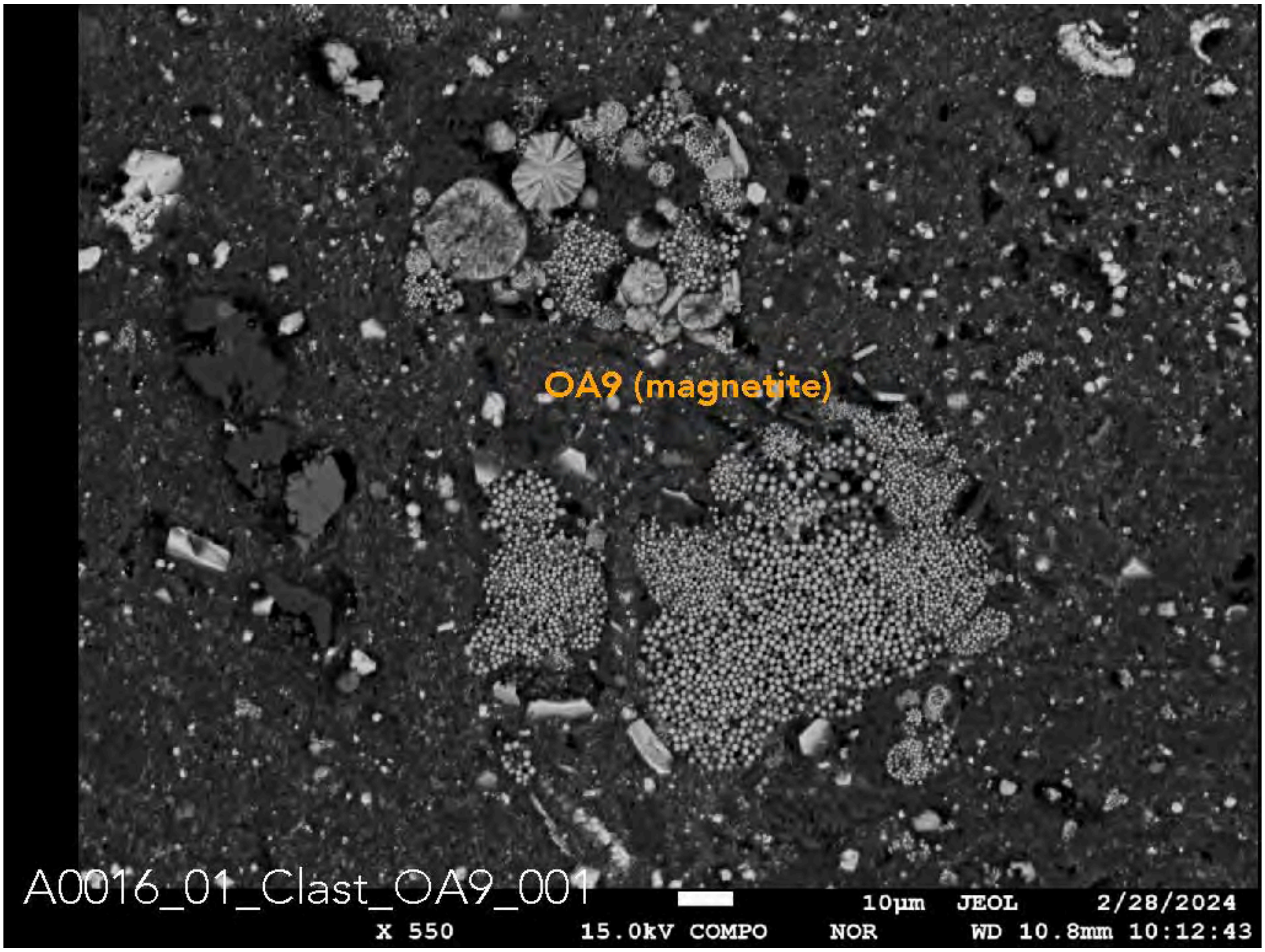

Supplementary Figure 29. Backscattered electron (BSE) image of clast region in A0016 showing OA9. OA = opaque assemblage.

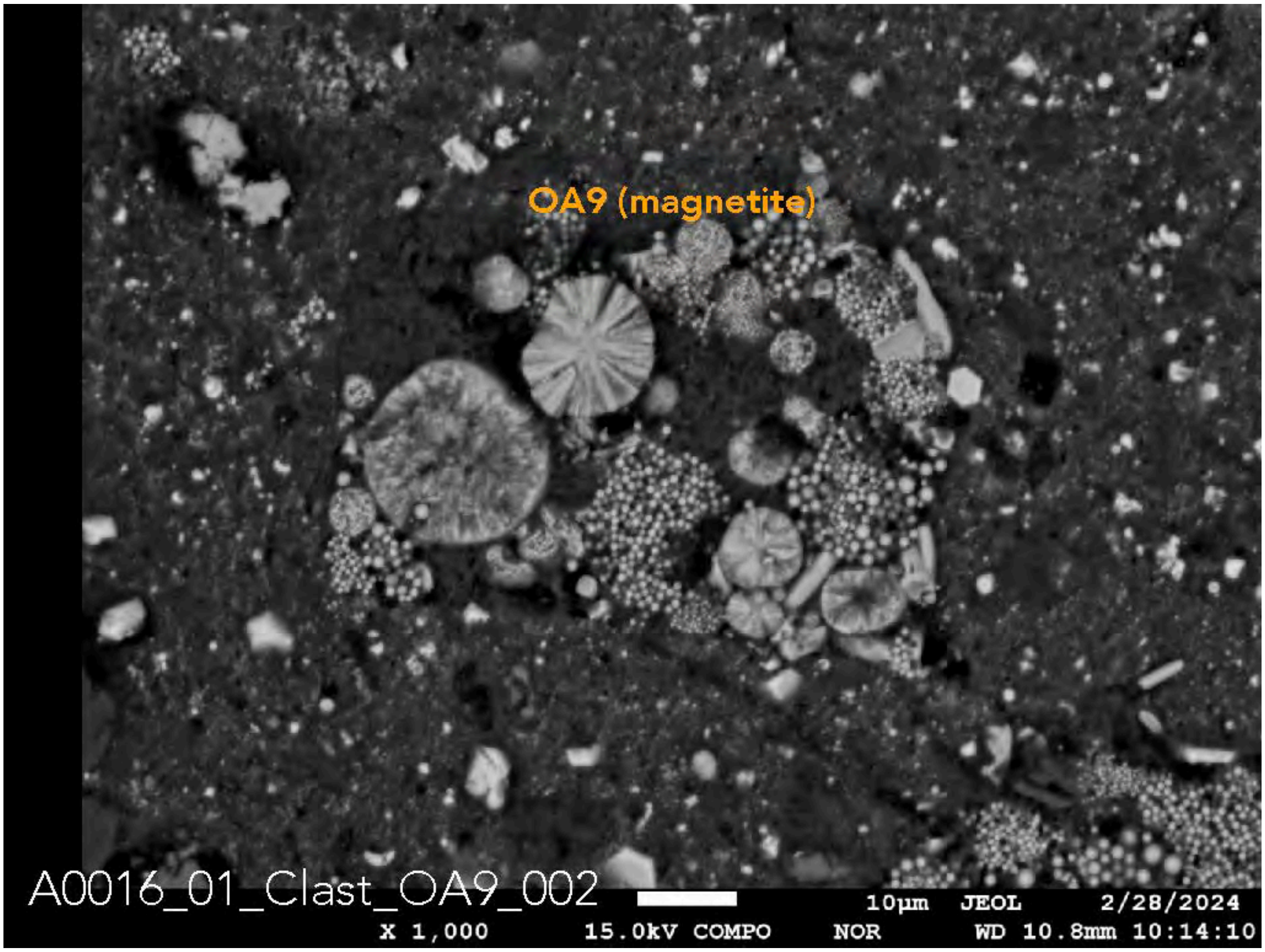

Supplementary Figure 30. Backscattered electron (BSE) image of clast region in A0016 showing OA9. OA = opaque assemblage.

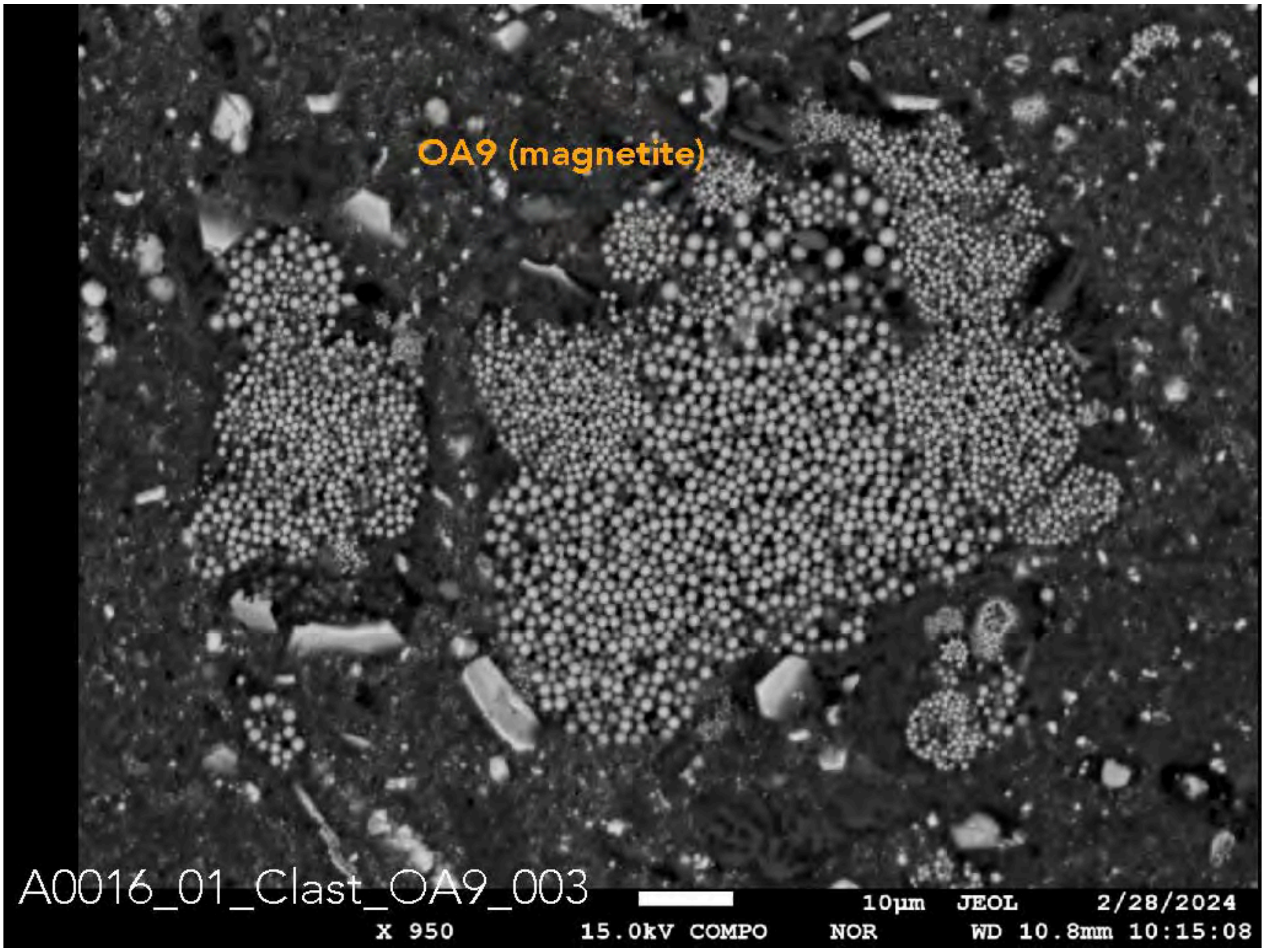

Supplementary Figure 31. Backscattered electron (BSE) image of clast region in A0016 showing OA9. OA = opaque assemblage.

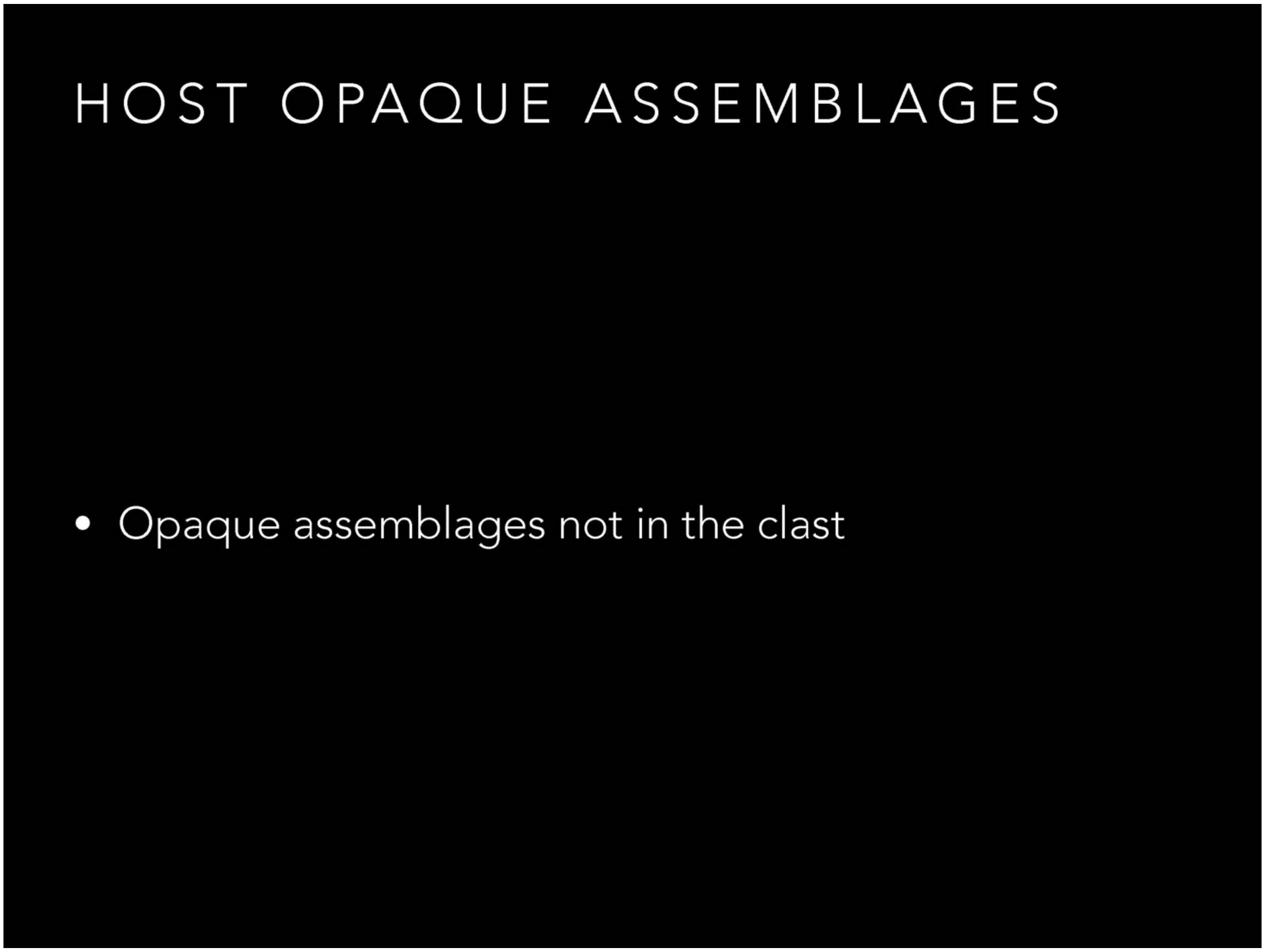

Supplementary Figure 32. Title slide for opaque assemblages outside of the clast in A0016.

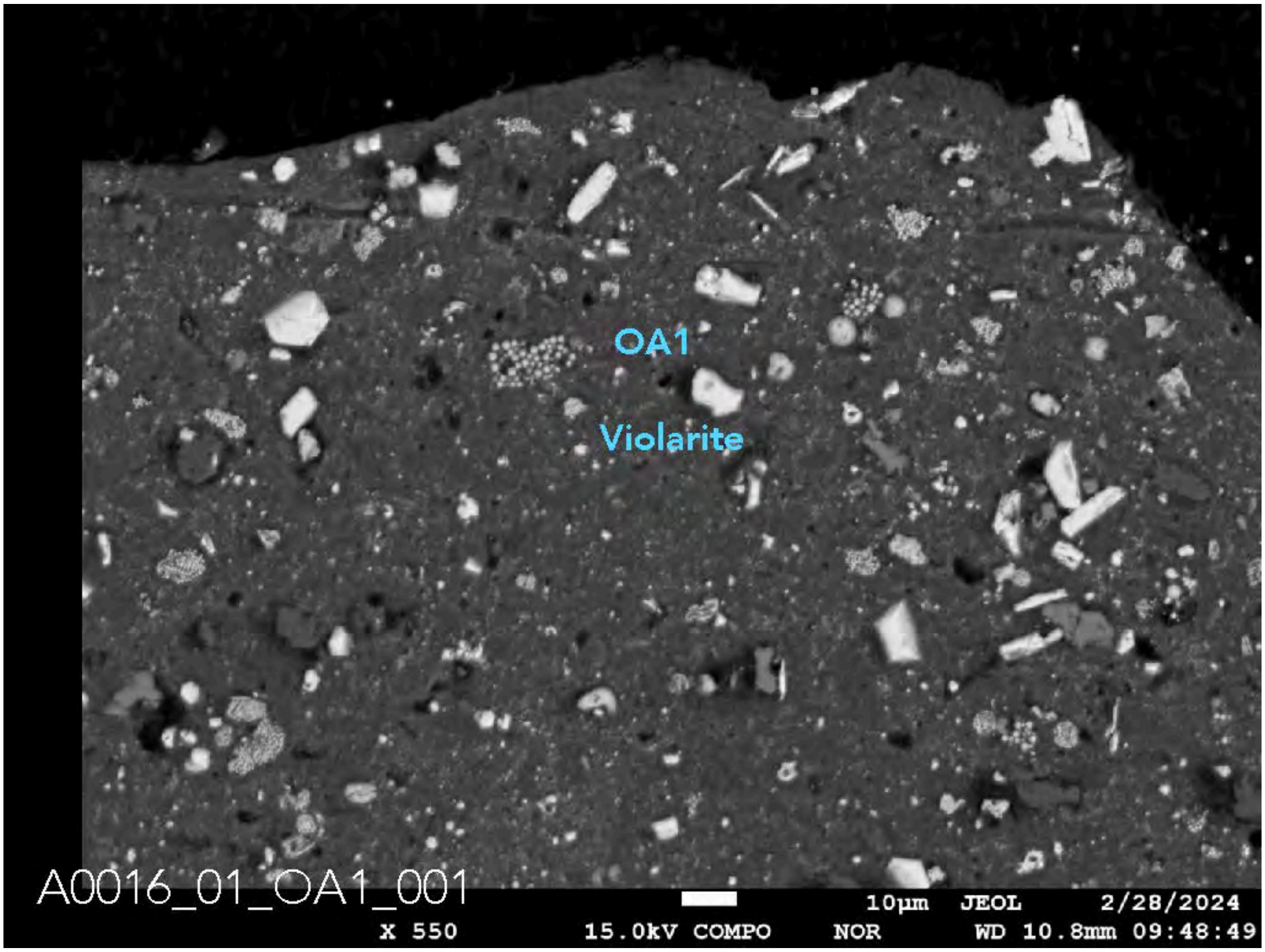

Supplementary Figure 33. Backscattered electron (BSE) image of OA1 in A0016. OA = opaque assemblage.

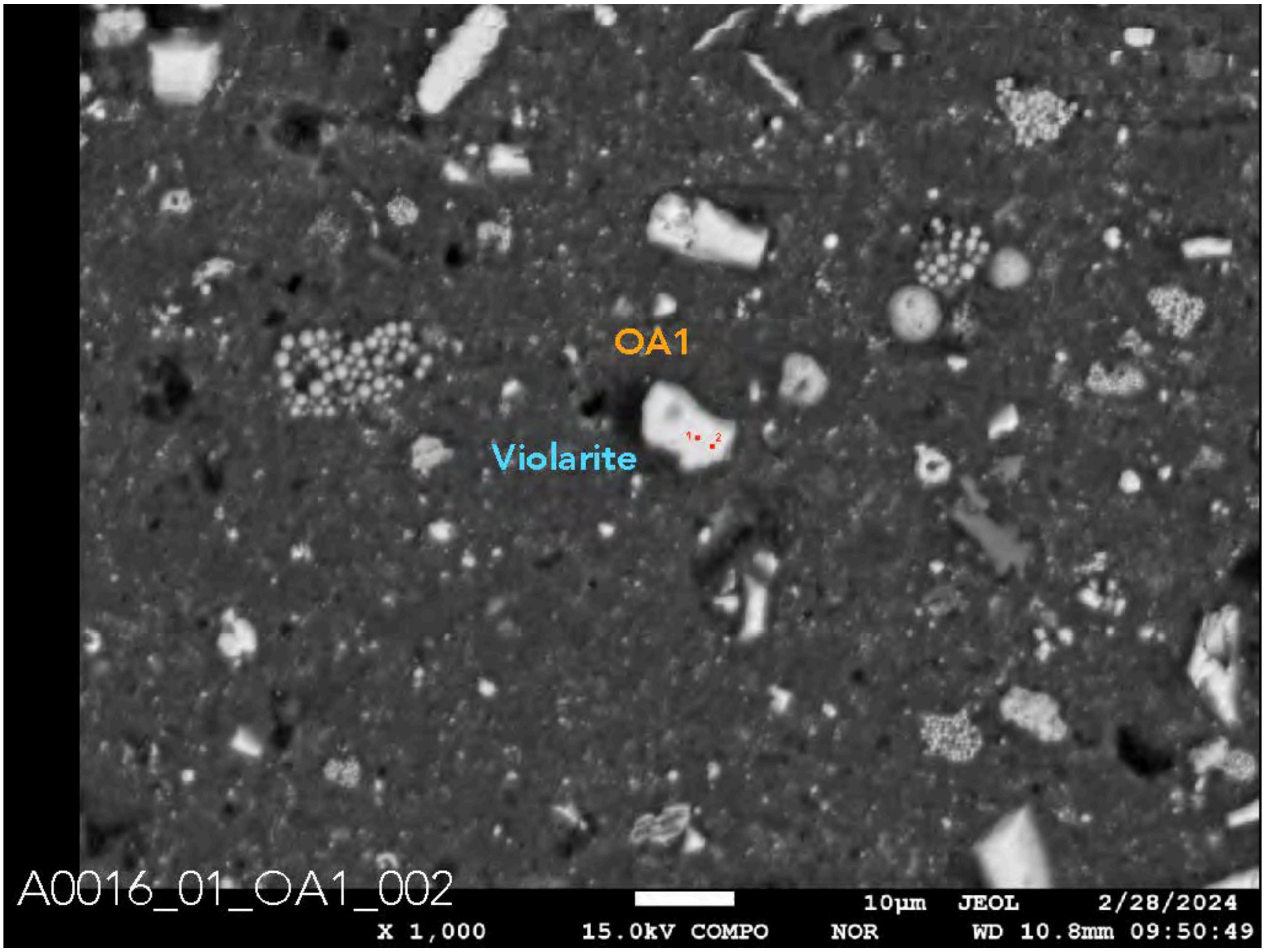

Supplementary Figure 34. Backscattered electron (BSE) image of OA1 in A0016, with spot analyses marked. OA = opaque assemblage.

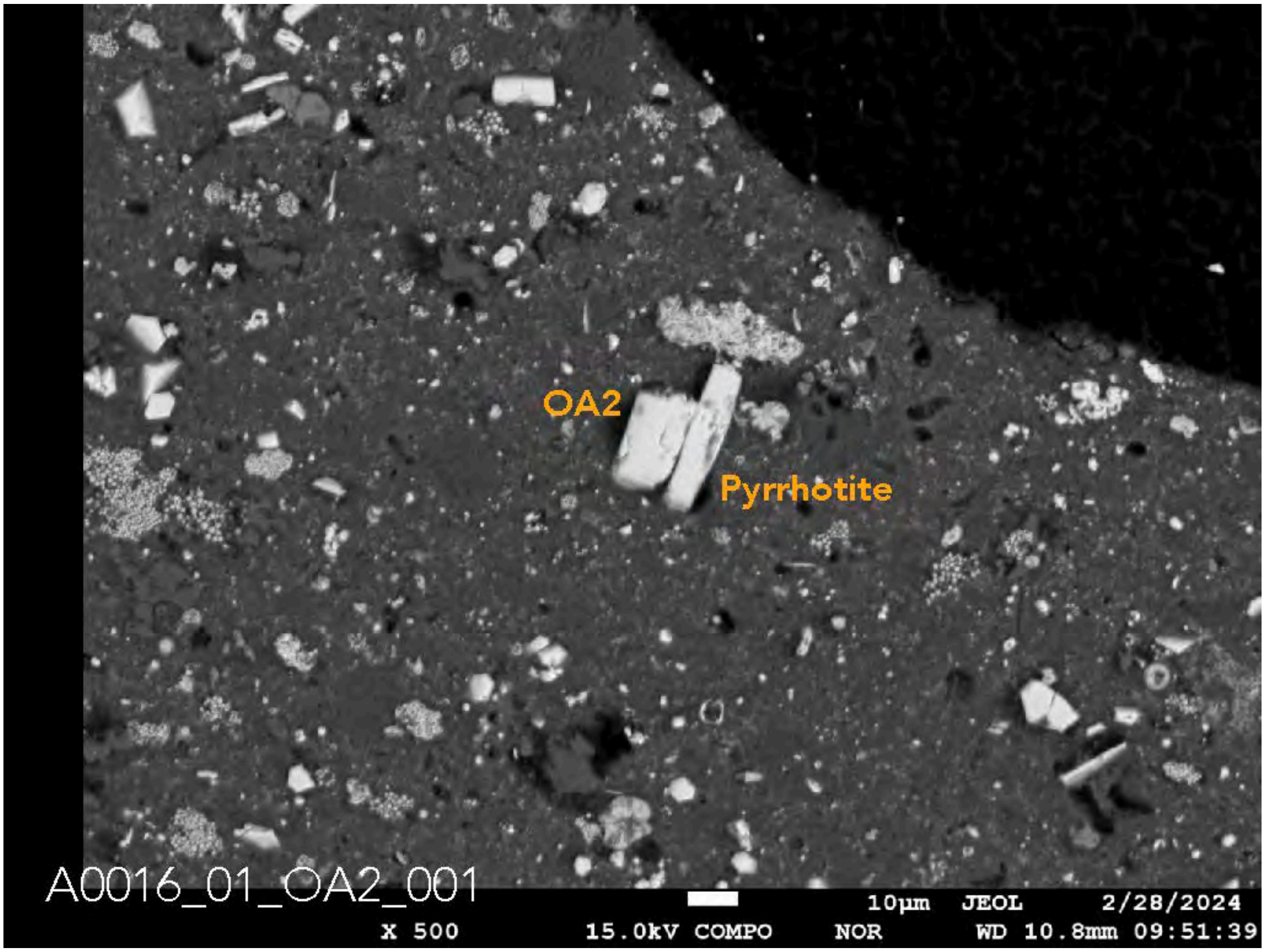

Supplementary Figure 35. Backscattered electron (BSE) image of OA2 in A0016. OA = opaque assemblage.

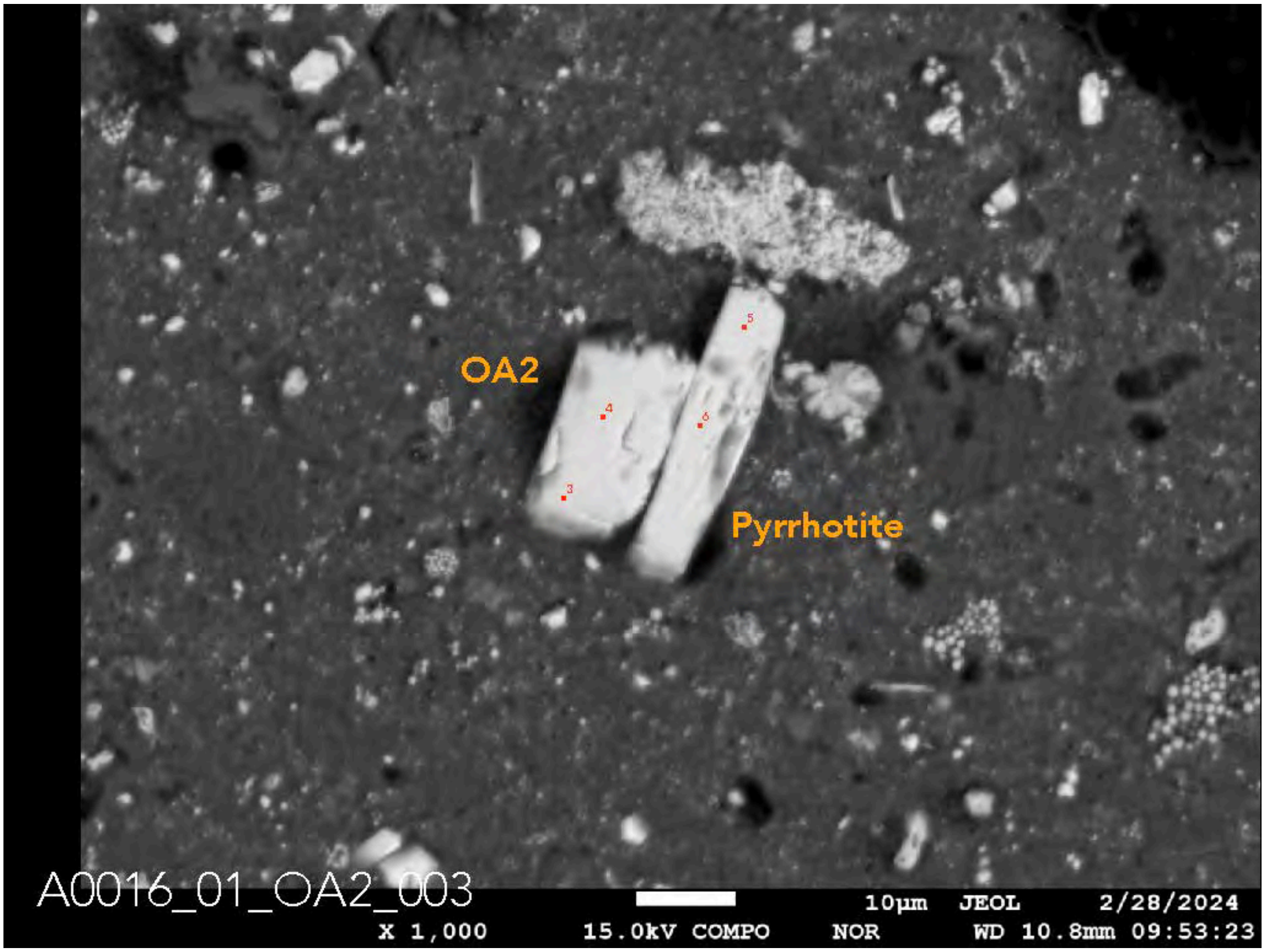

Supplementary Figure 36. Backscattered electron (BSE) image of OA2 in A0016, with spot analyses marked. OA = opaque assemblage.

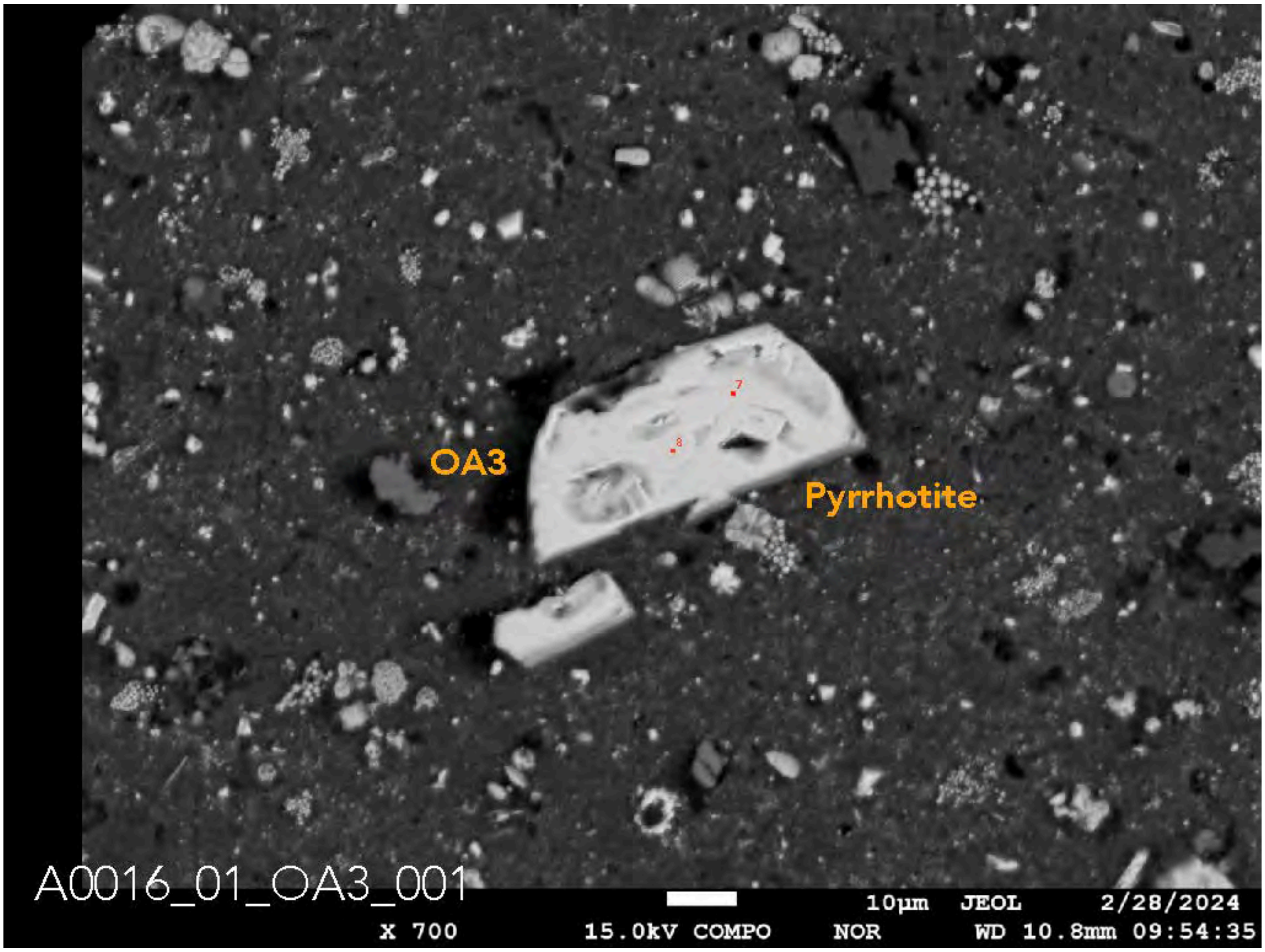

Supplementary Figure 37. Backscattered electron (BSE) image of OA3 in A0016, with spot analyses marked. OA = opaque assemblage.

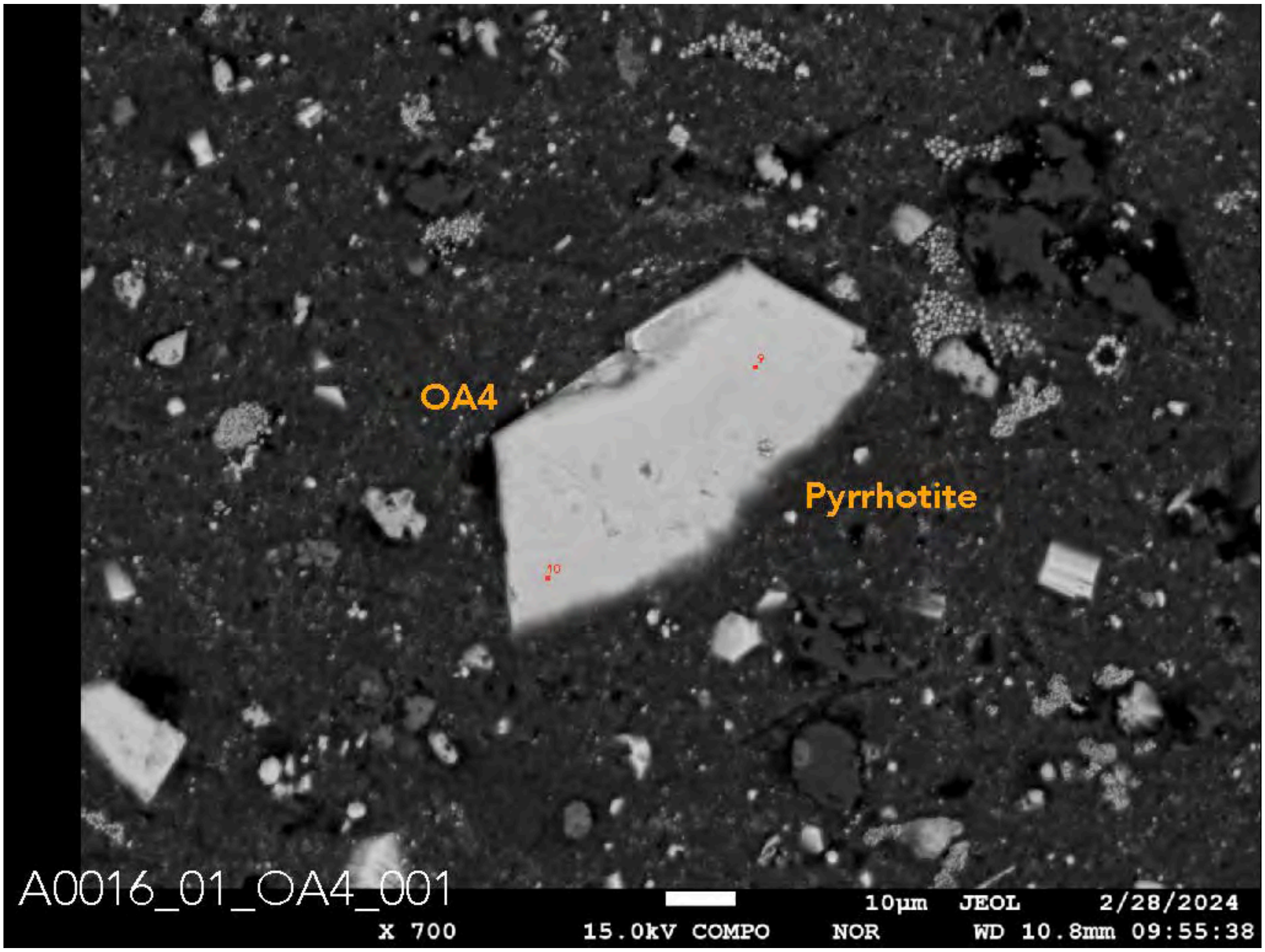

Supplementary Figure 38. Backscattered electron (BSE) image of OA4 in A0016, with spot analyses marked. OA = opaque assemblage.

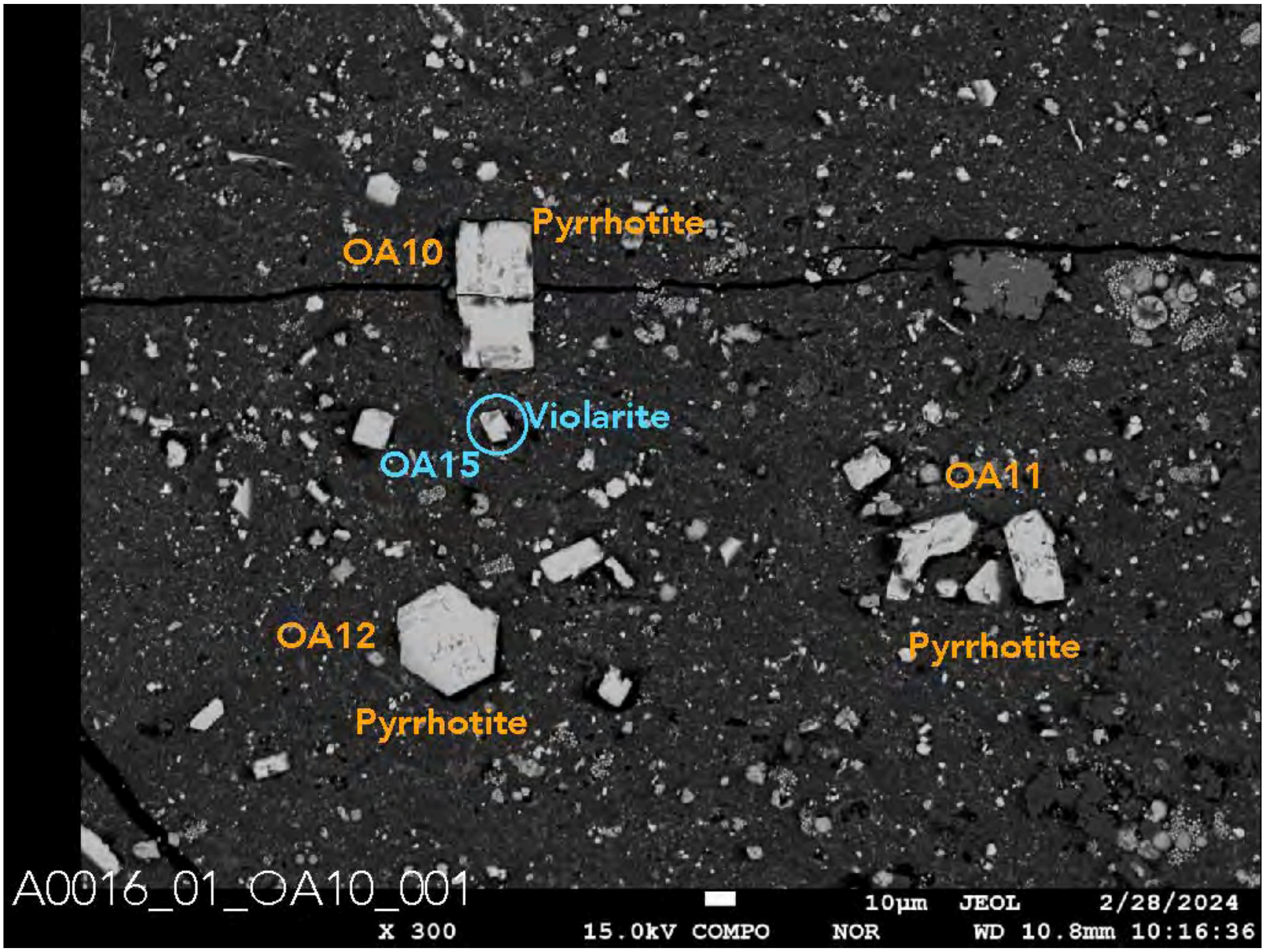

Supplementary Figure 39. Backscattered electron (BSE) image of OA10, OA11, OA12, and OA15 in A0016. OA = opaque assemblage.

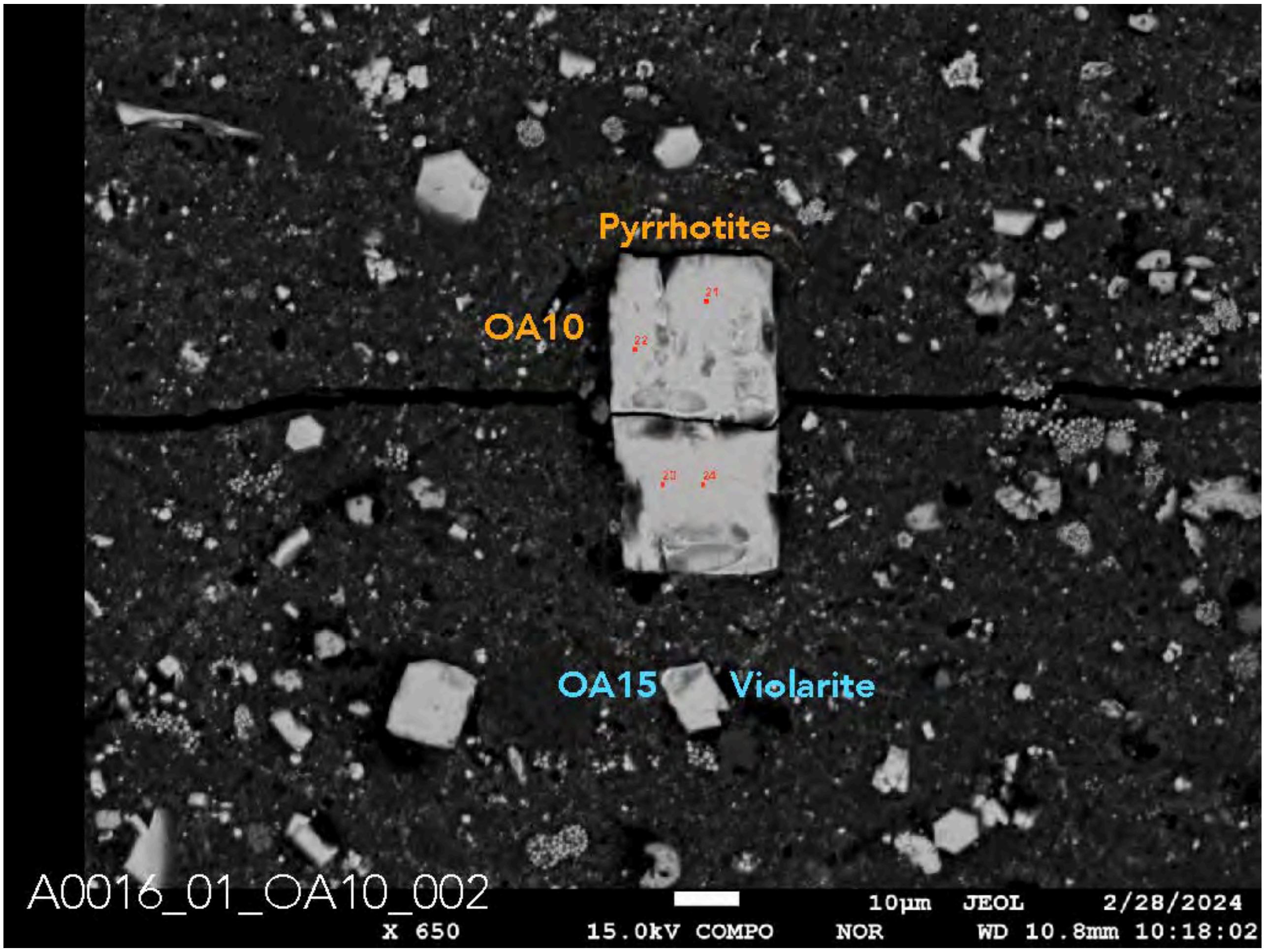

Supplementary Figure 40. Backscattered electron (BSE) image of OA10 and OA15 in A0016, with spot analyses marked. OA = opaque assemblage.

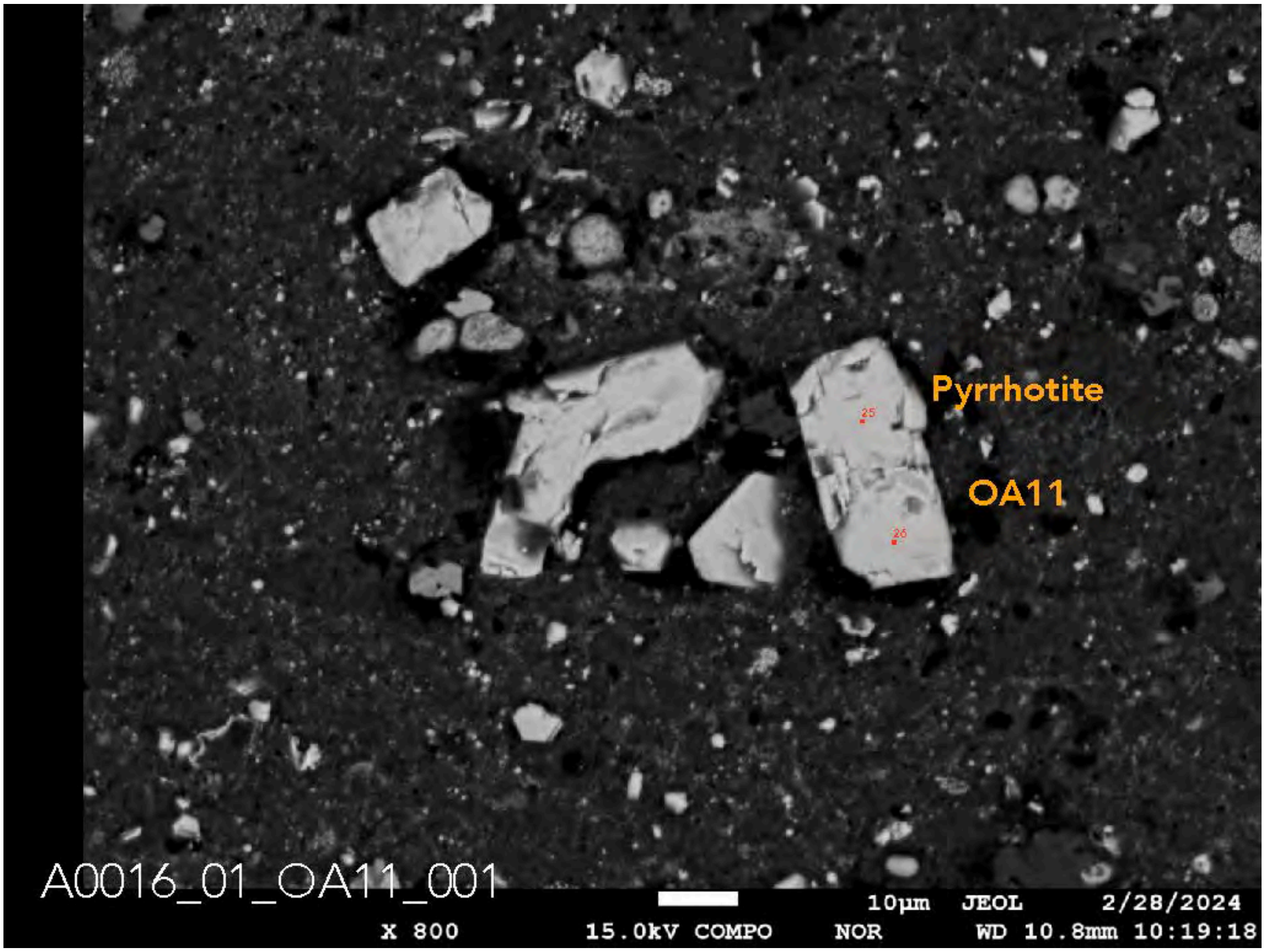

Supplementary Figure 41. Backscattered electron (BSE) image of OA11 in A0016, with spot analyses marked. OA = opaque assemblage.

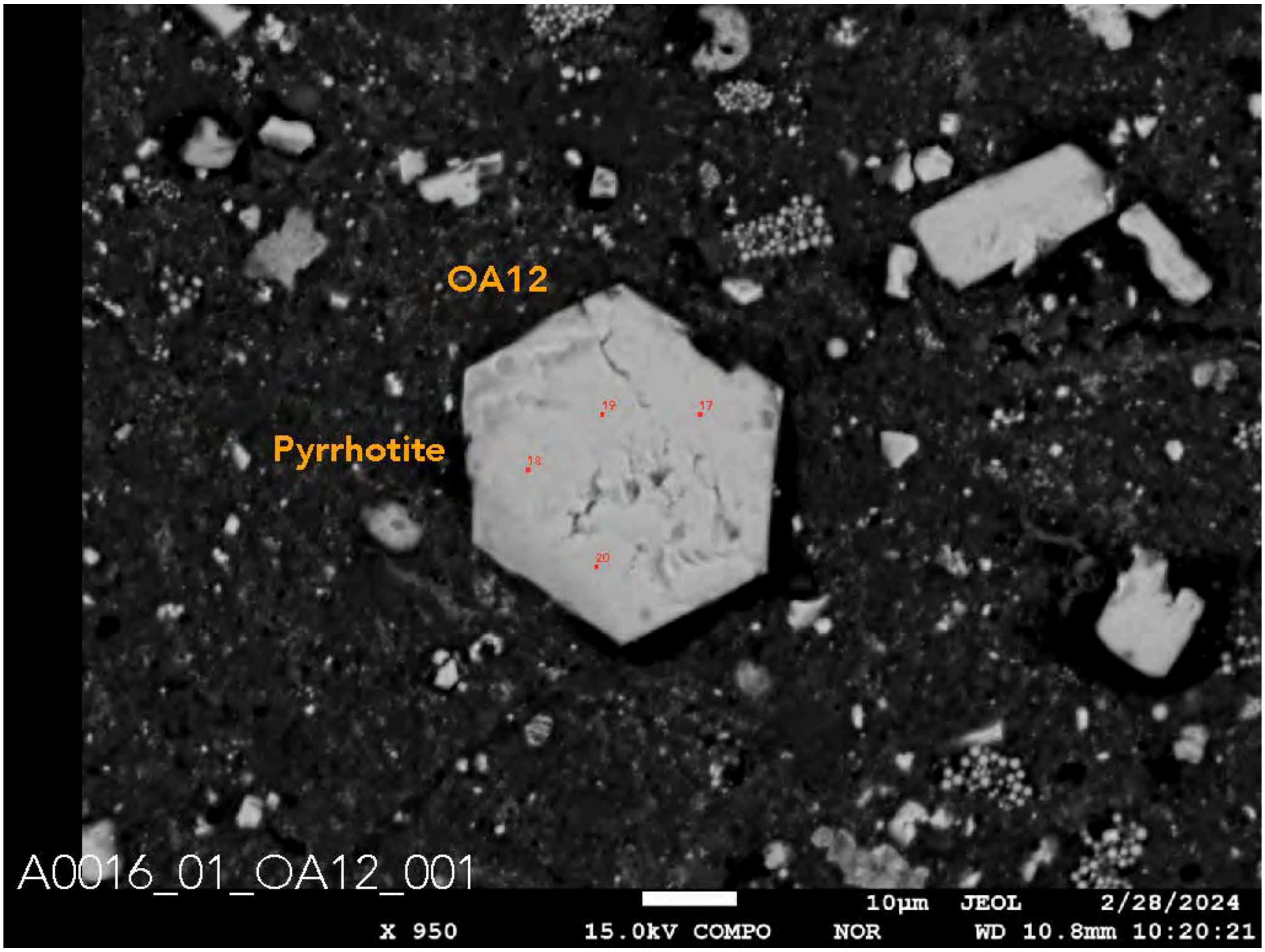

Supplementary Figure 42. Backscattered electron (BSE) image of OA12 in A0016, with spot analyses marked. OA = opaque assemblage.

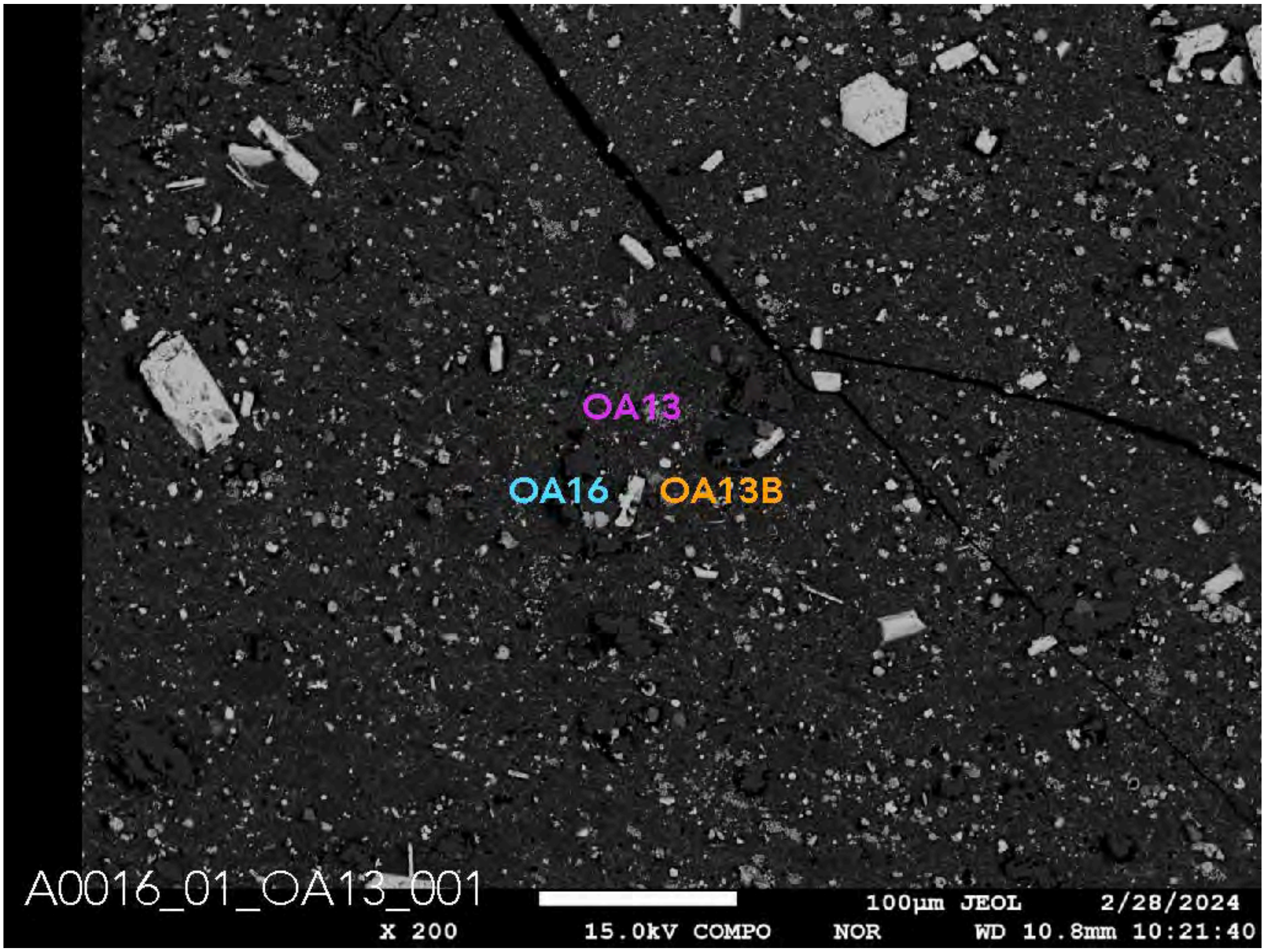

Supplementary Figure 43. Backscattered electron (BSE) image of OA13, OA13B, and OA16 in A0016. OA = opaque assemblage.

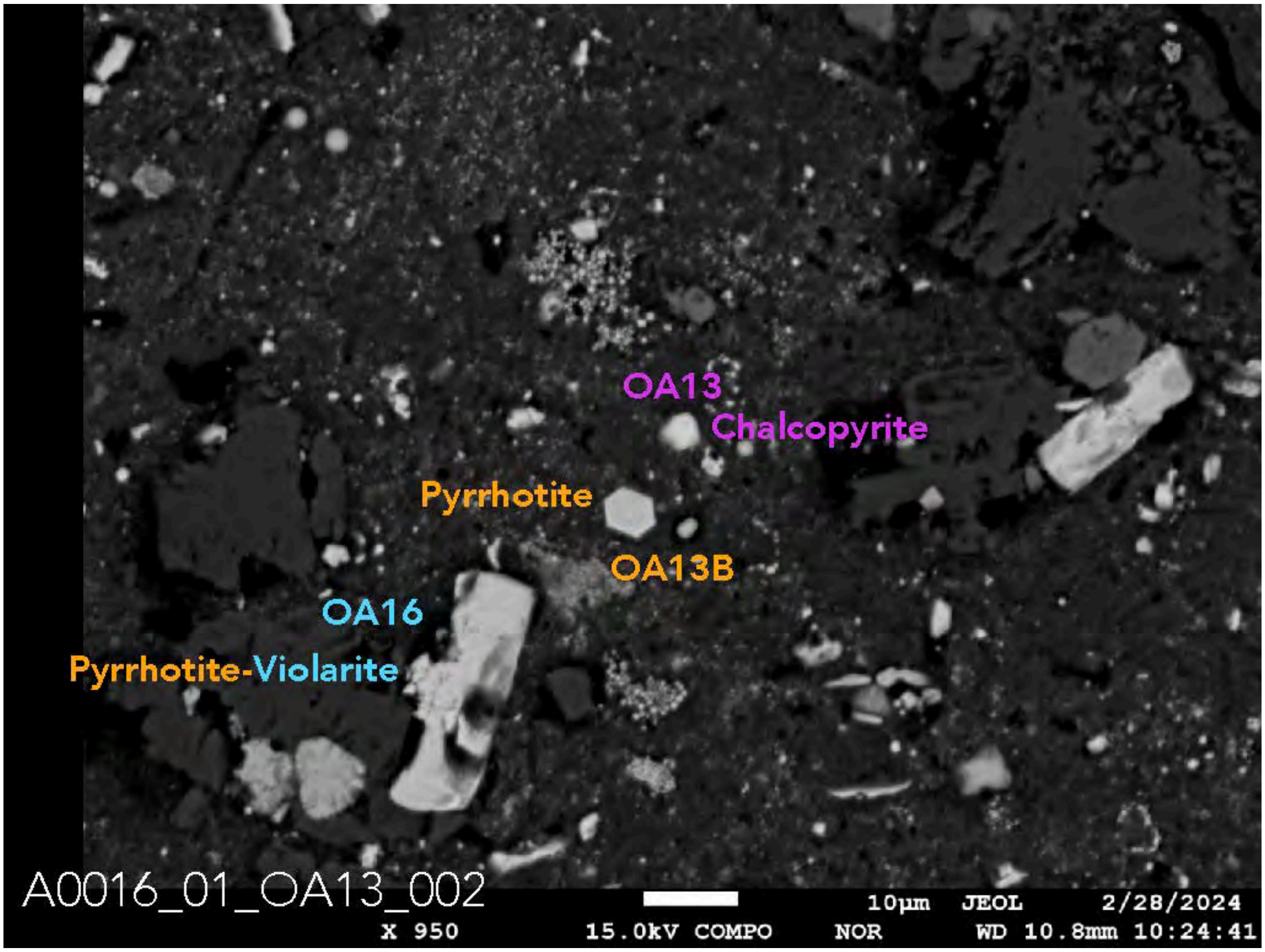

Supplementary Figure 44. Backscattered electron (BSE) image of OA13, OA13B, and OA16 in A0016. OA = opaque assemblage.

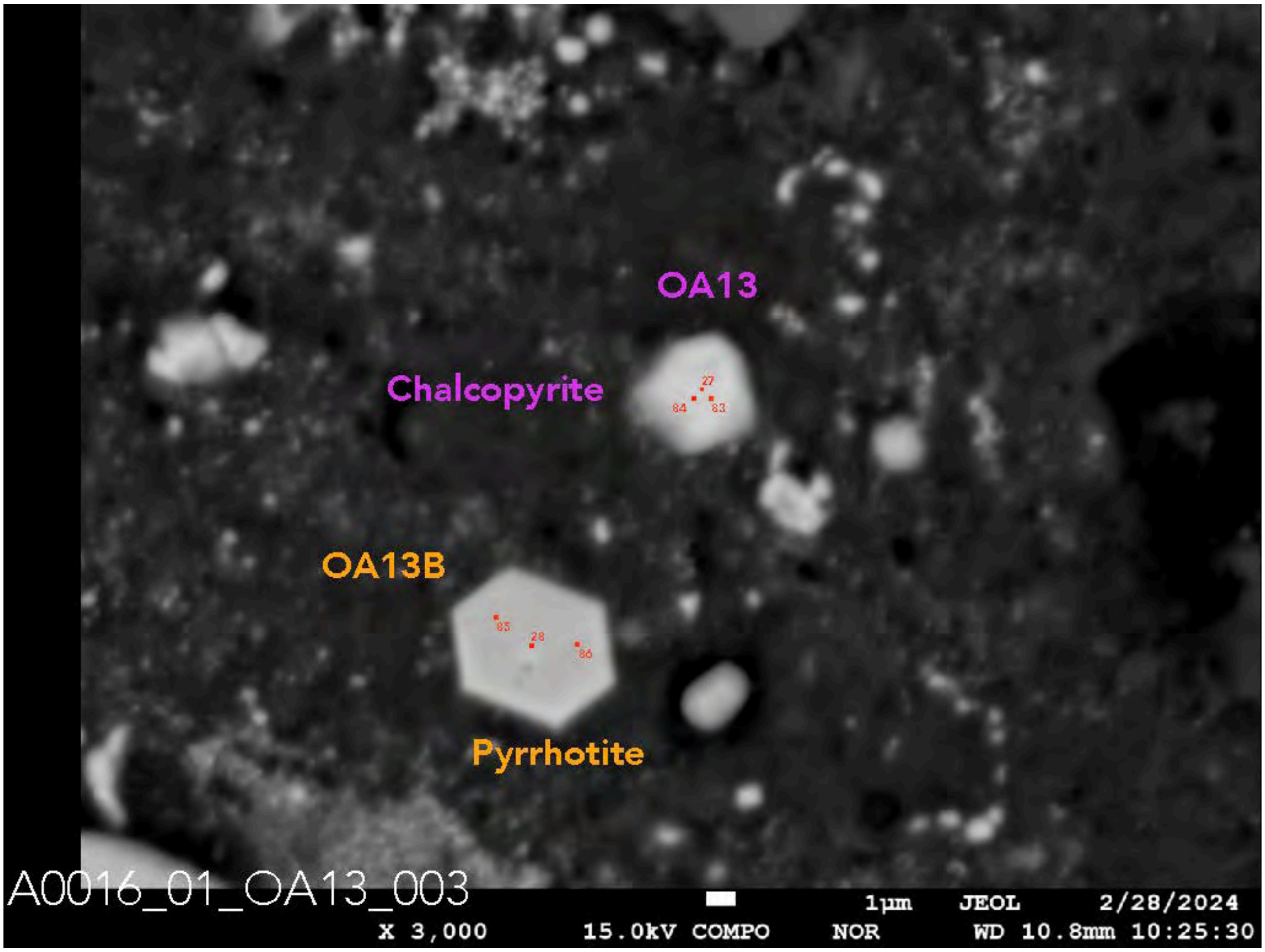

Supplementary Figure 45. Backscattered electron (BSE) image of OA13 and OA13B in A0016, with spot analyses marked. OA = opaque assemblage.

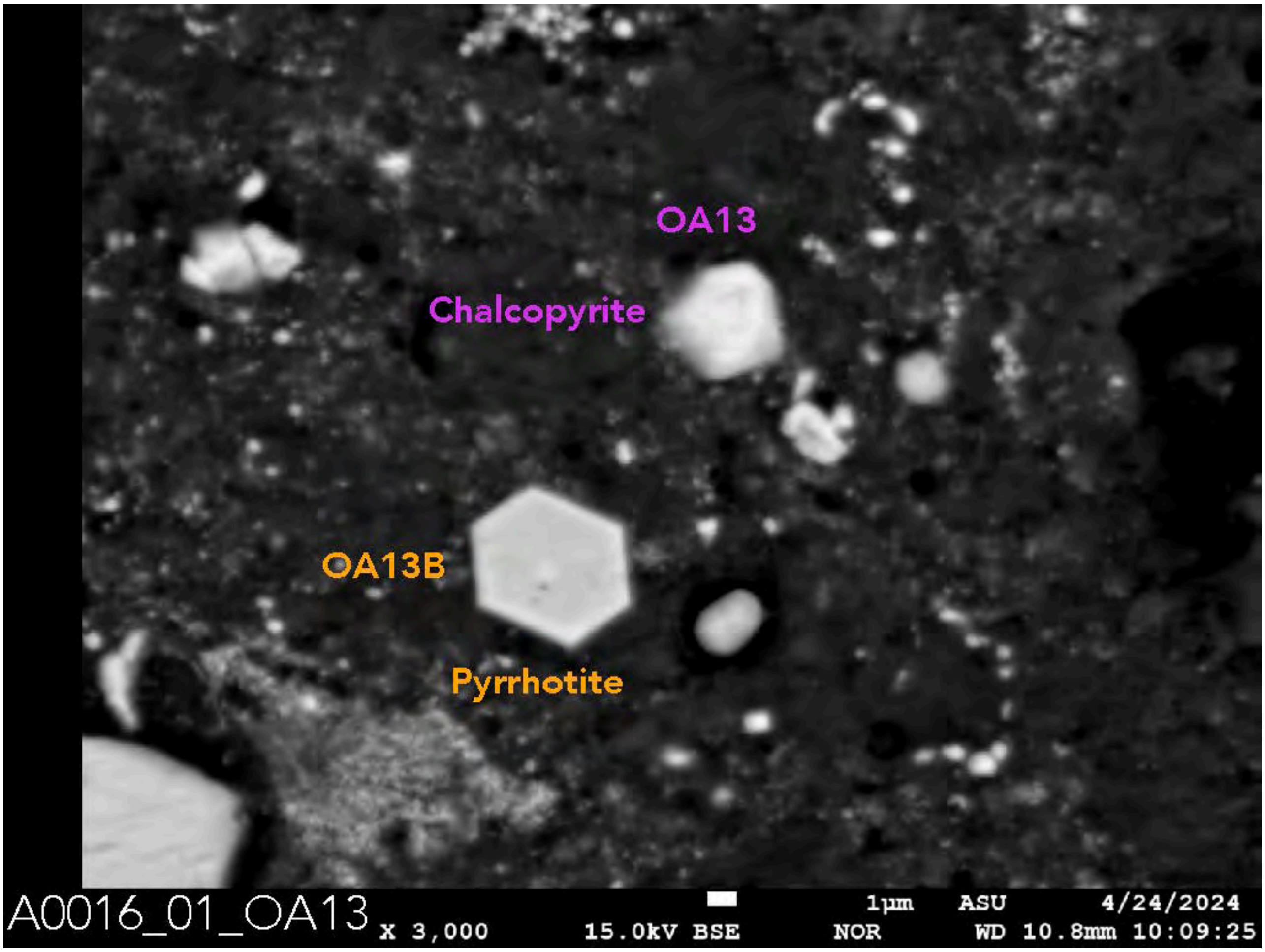

Supplementary Figure 46. Backscattered electron (BSE) image of OA13 and OA13B in A0016. OA = opaque assemblage.

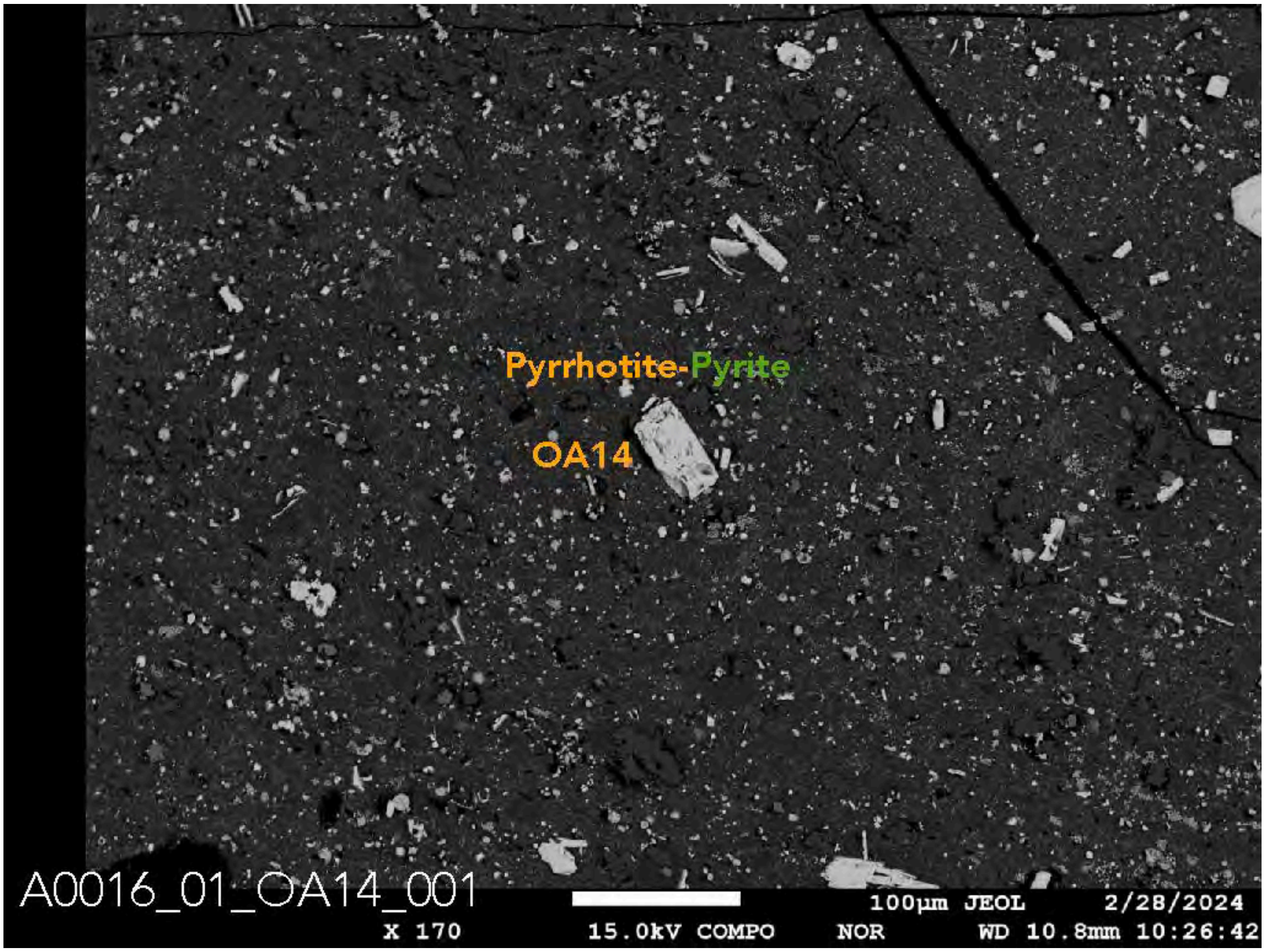

Supplementary Figure 47. Backscattered electron (BSE) image of OA14 in A0016. OA = opaque assemblage.

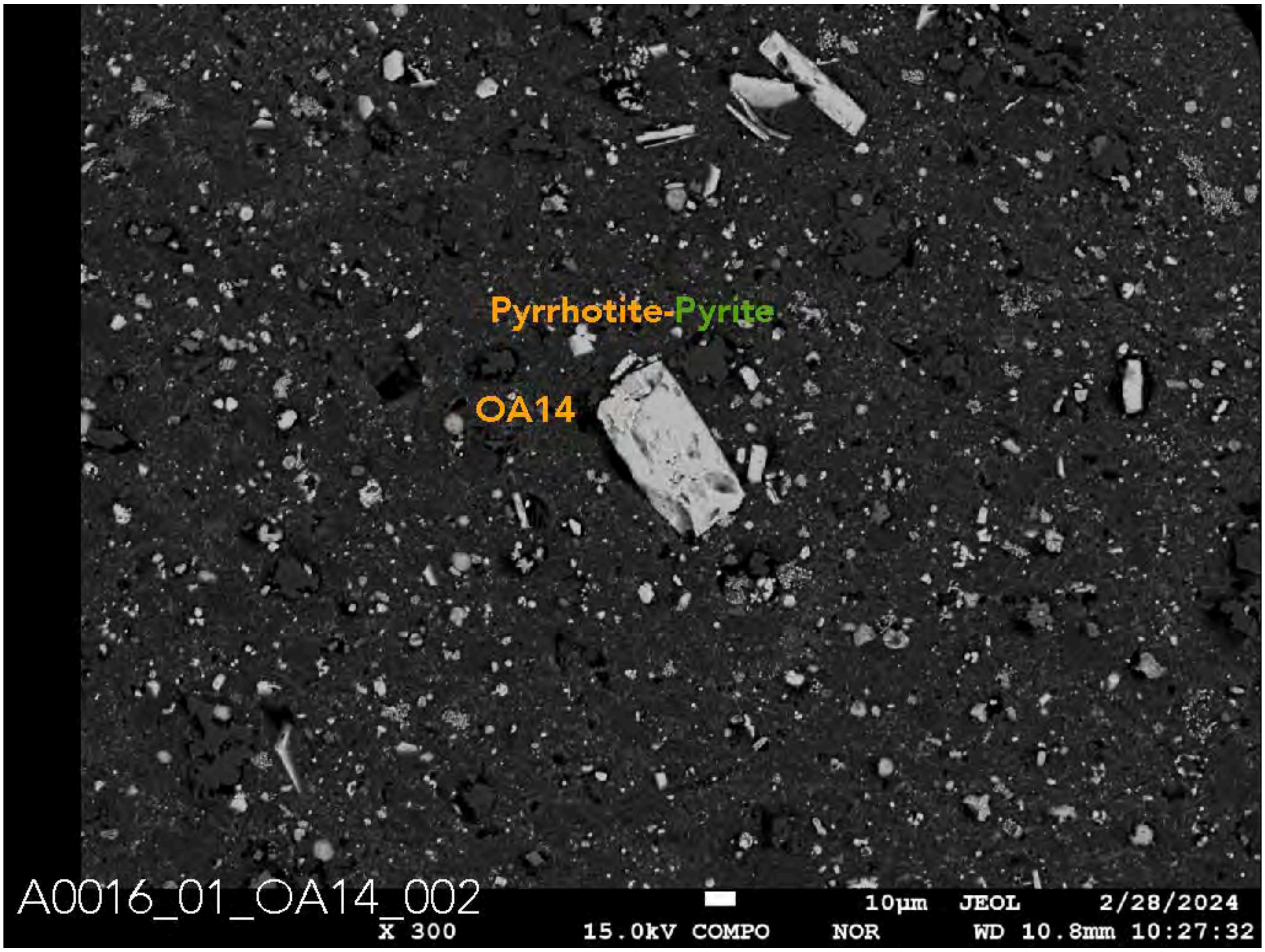

Supplementary Figure 48. Backscattered electron (BSE) image of OA14 in A0016. OA = opaque assemblage.

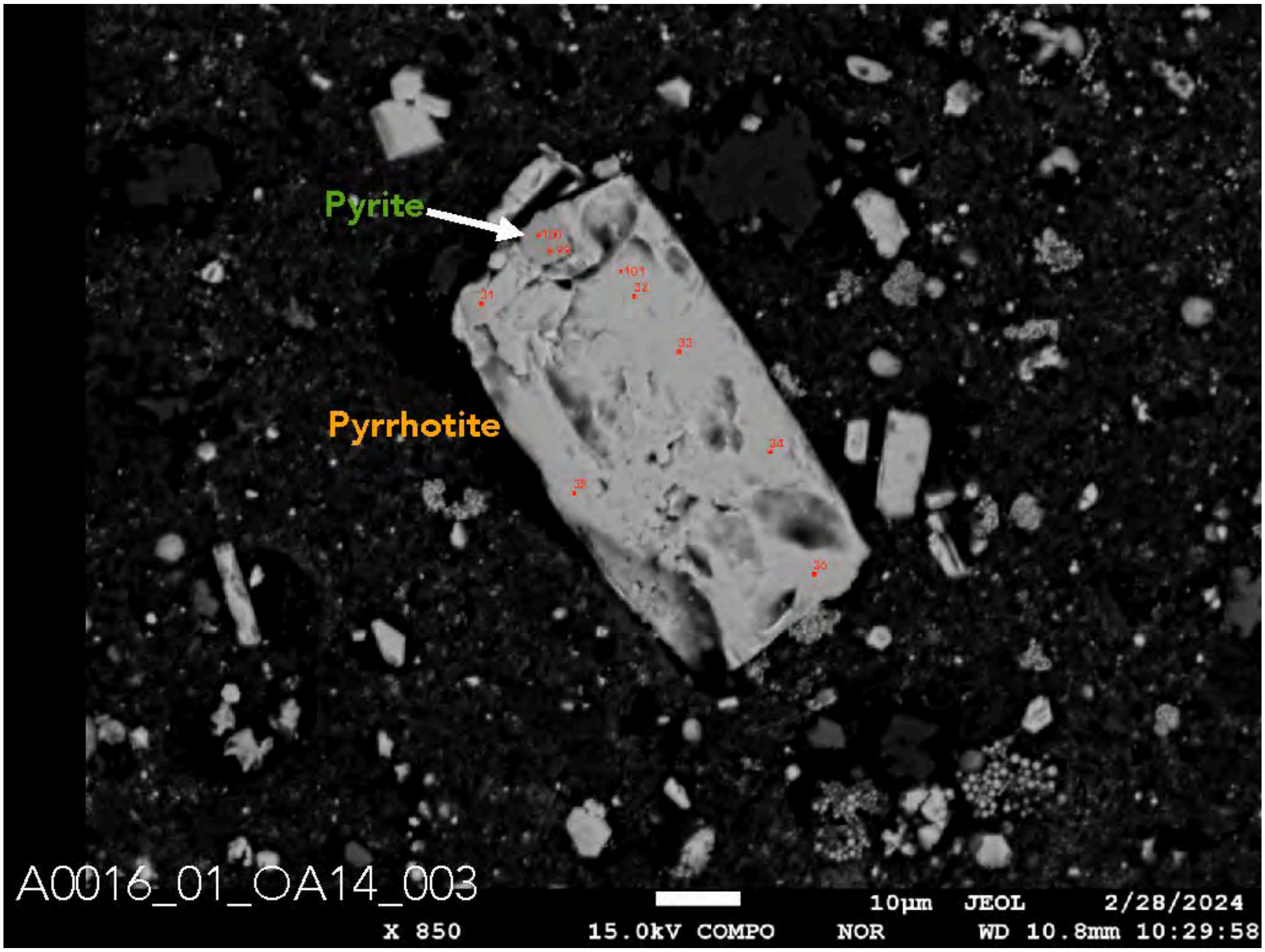

Supplementary Figure 49. Backscattered electron (BSE) image of OA14 in A0016, with spot analyses marked. OA = opaque assemblage.

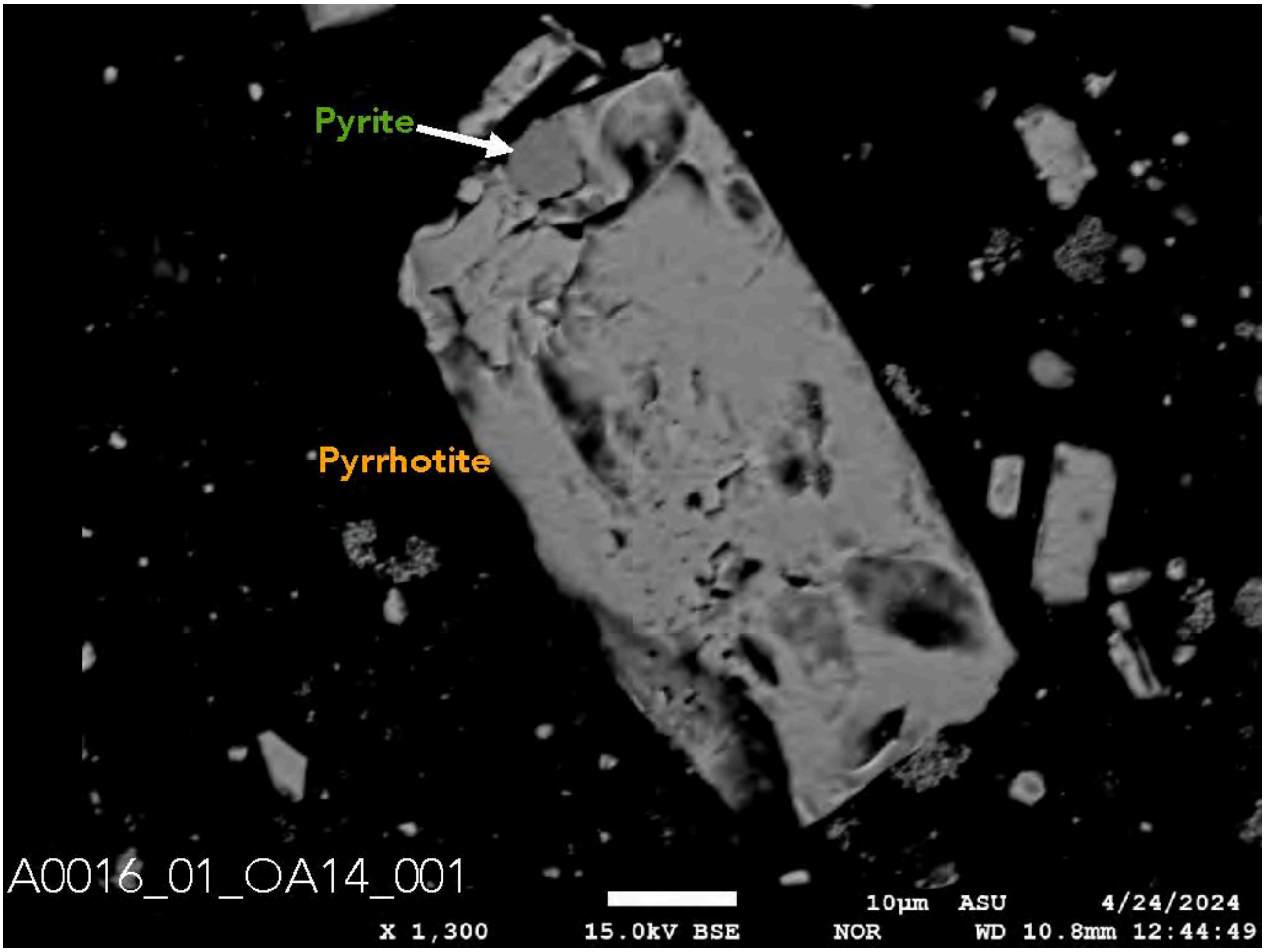

Supplementary Figure 50. Backscattered electron (BSE) image of OA14 in A0016. OA = opaque assemblage.

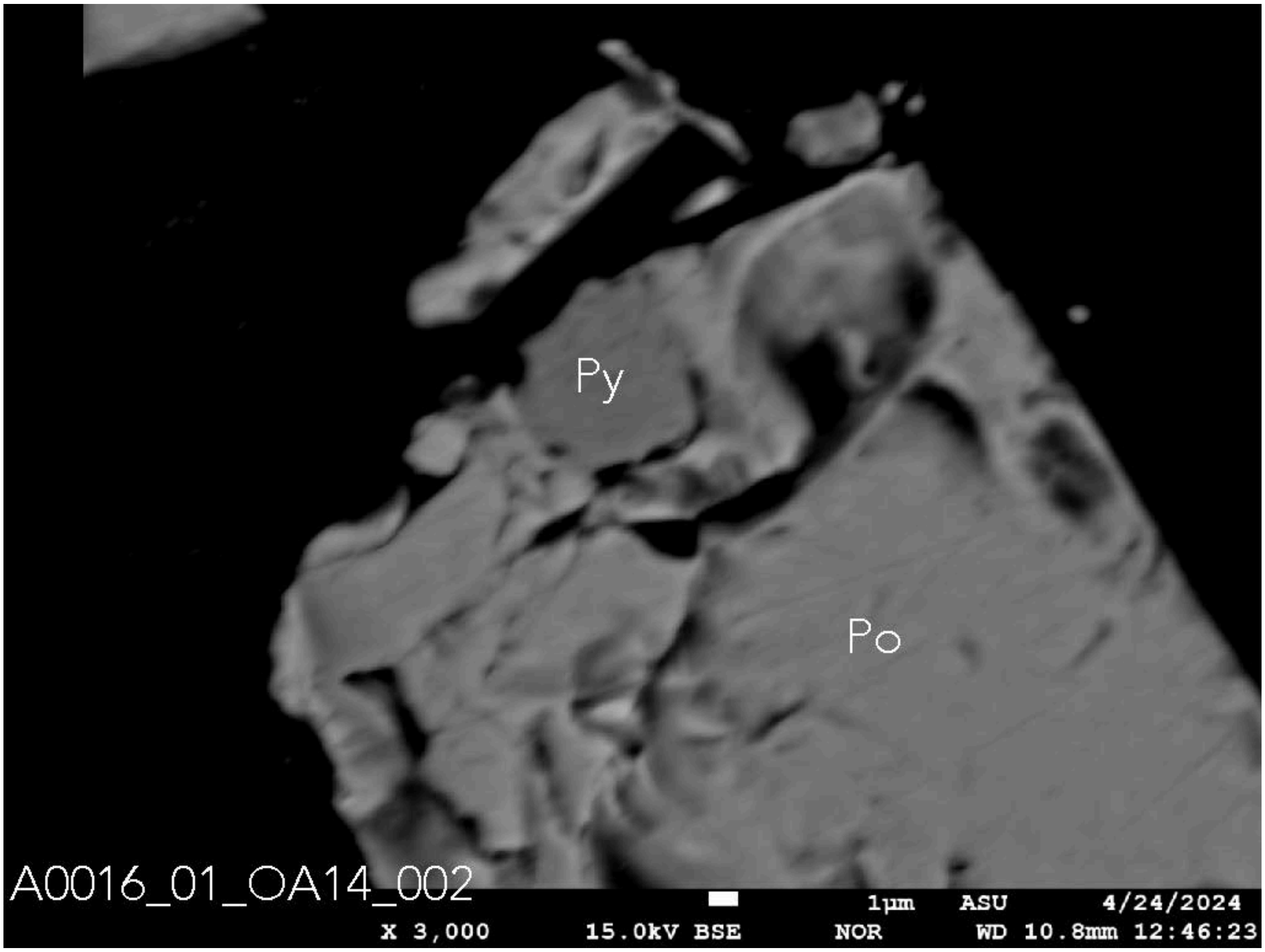

Supplementary Figure 51. Backscattered electron (BSE) image of OA14 in A0016. OA = opaque assemblage. Py = pyrite. Po = pyrrhotite.

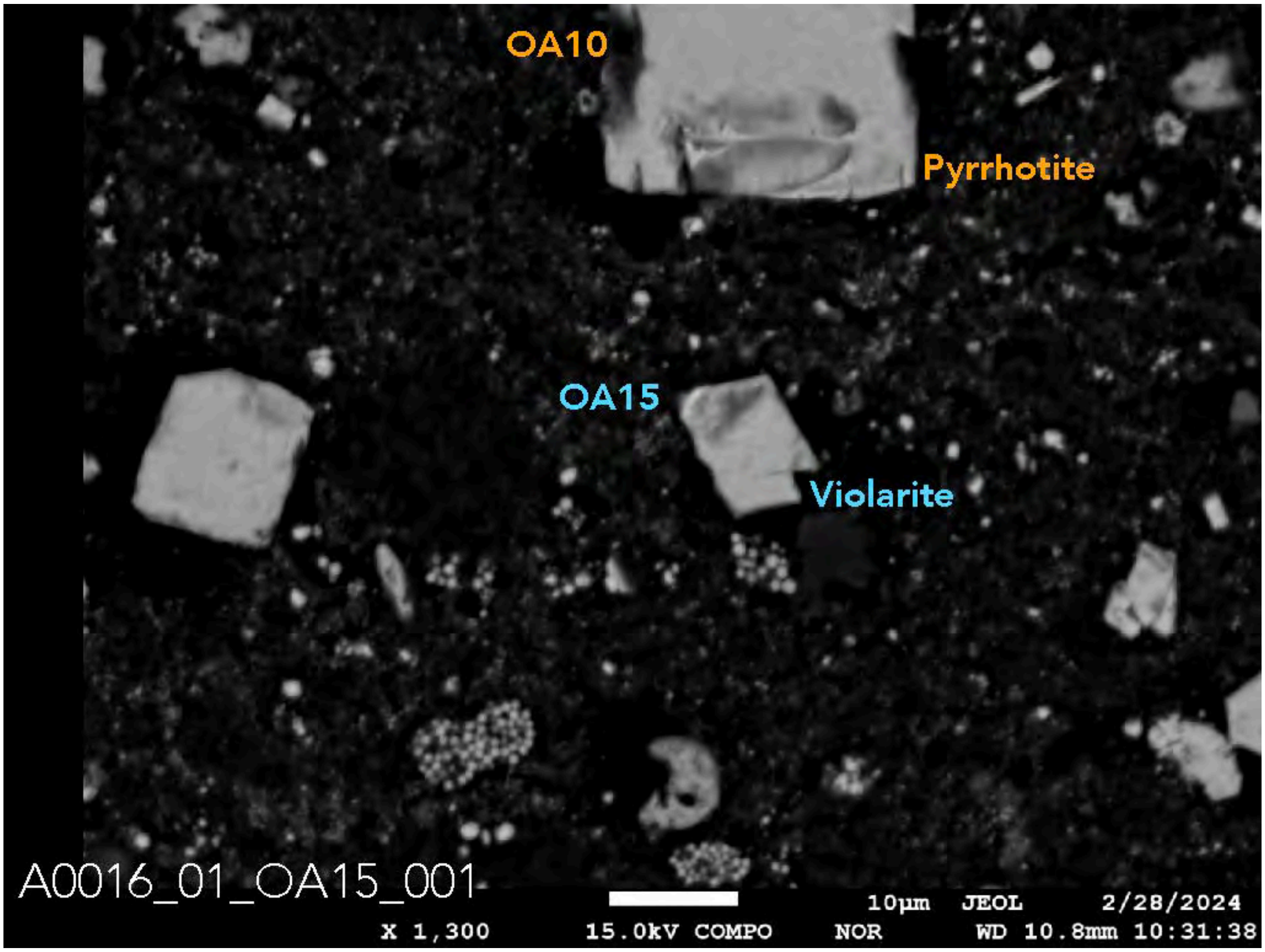

Supplementary Figure 52. Backscattered electron (BSE) image of OA10 and OA15 in A0016. OA = opaque assemblage.

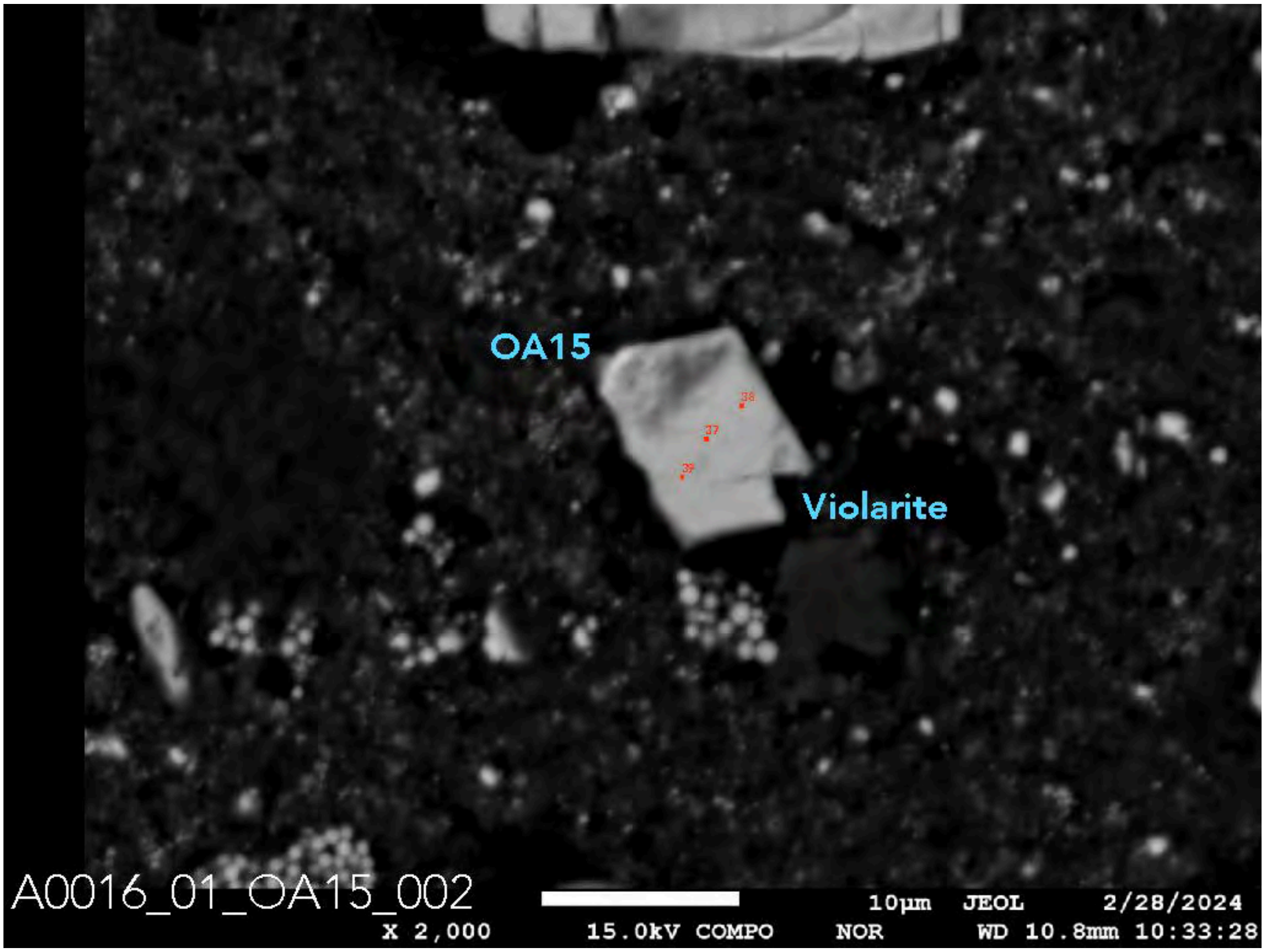

Supplementary Figure 53. Backscattered electron (BSE) image of OA15 in A0016, with spot analyses marked. OA = opaque assemblage.

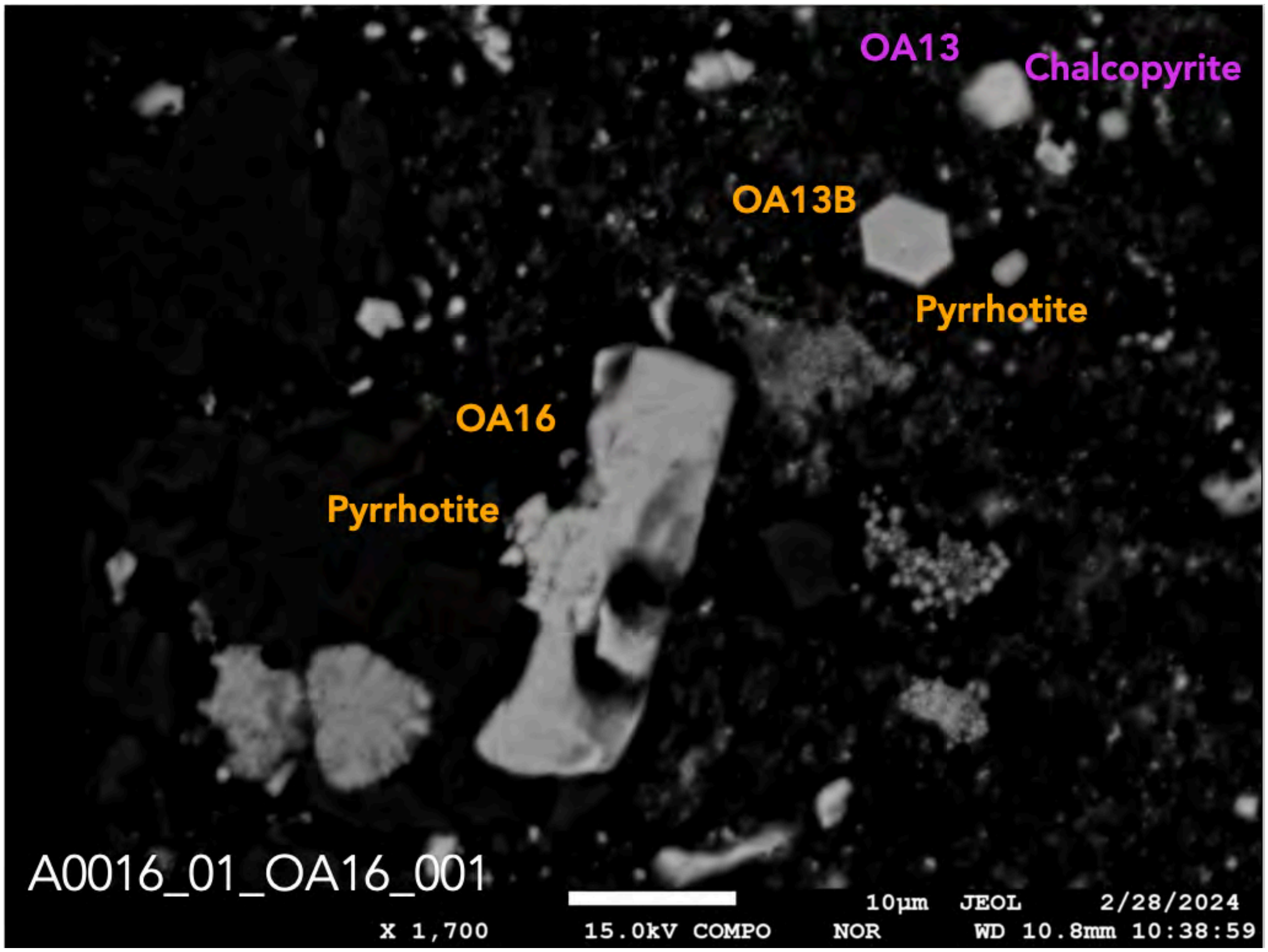

Supplementary Figure 54. Backscattered electron (BSE) image of OA13, OA13B, and OA16 in A0016. OA = opaque assemblage.

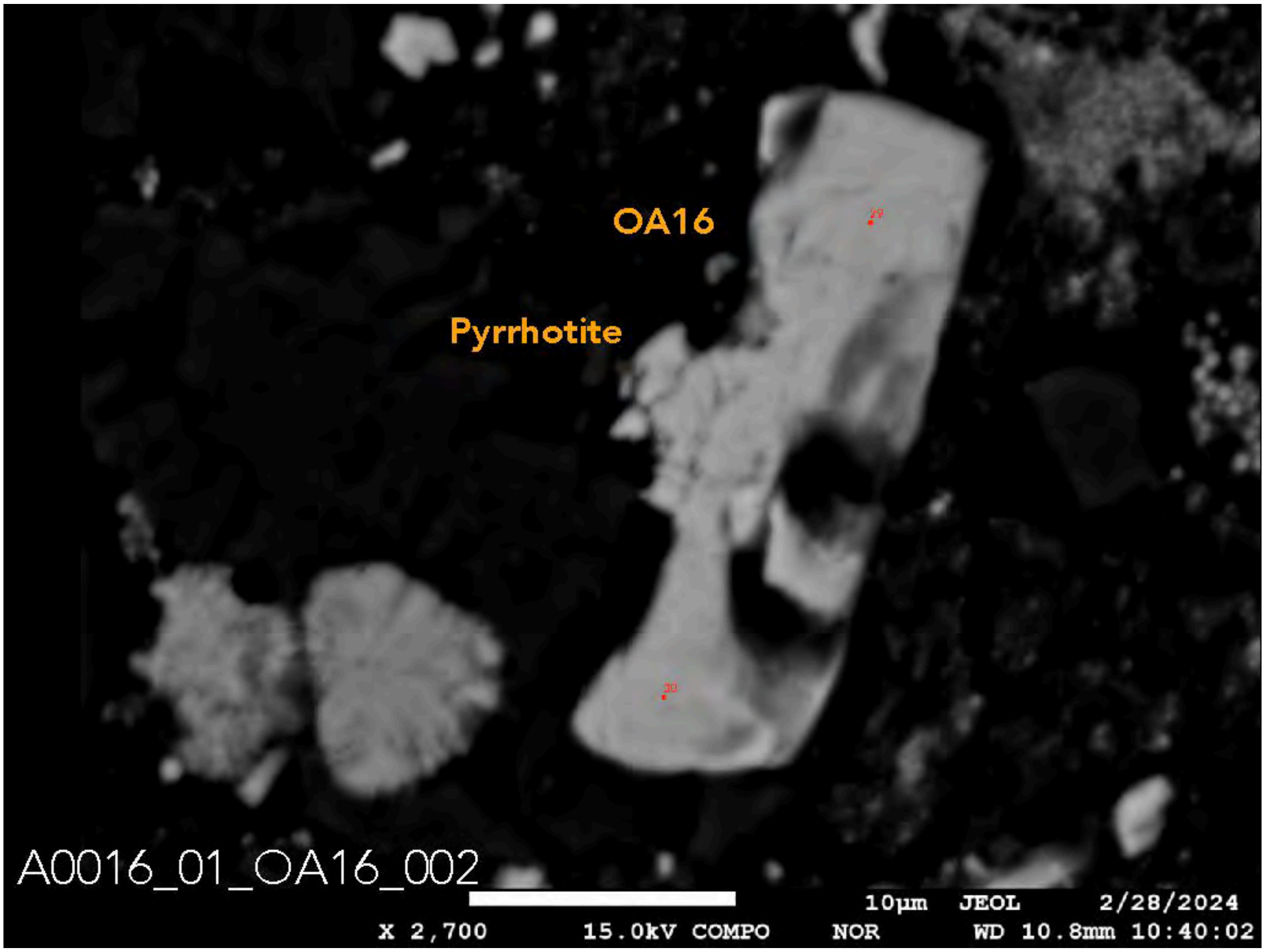

Supplementary Figure 55. Backscattered electron (BSE) image of OA16 in A0016, with spot analyses marked. OA = opaque assemblage.

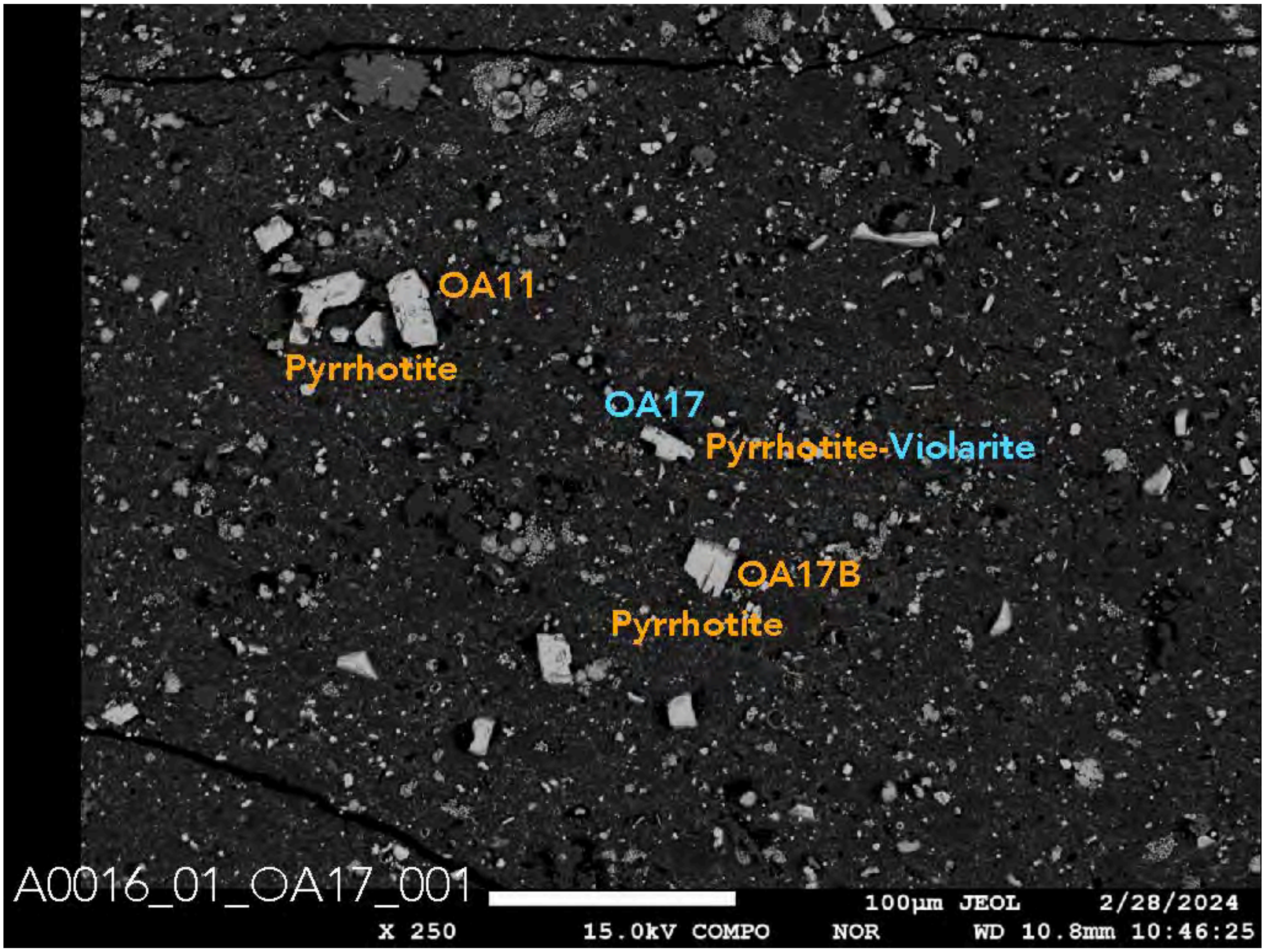

Supplementary Figure 56. Backscattered electron (BSE) image of OA11, OA17, and OA17B in A0016. OA = opaque assemblage.

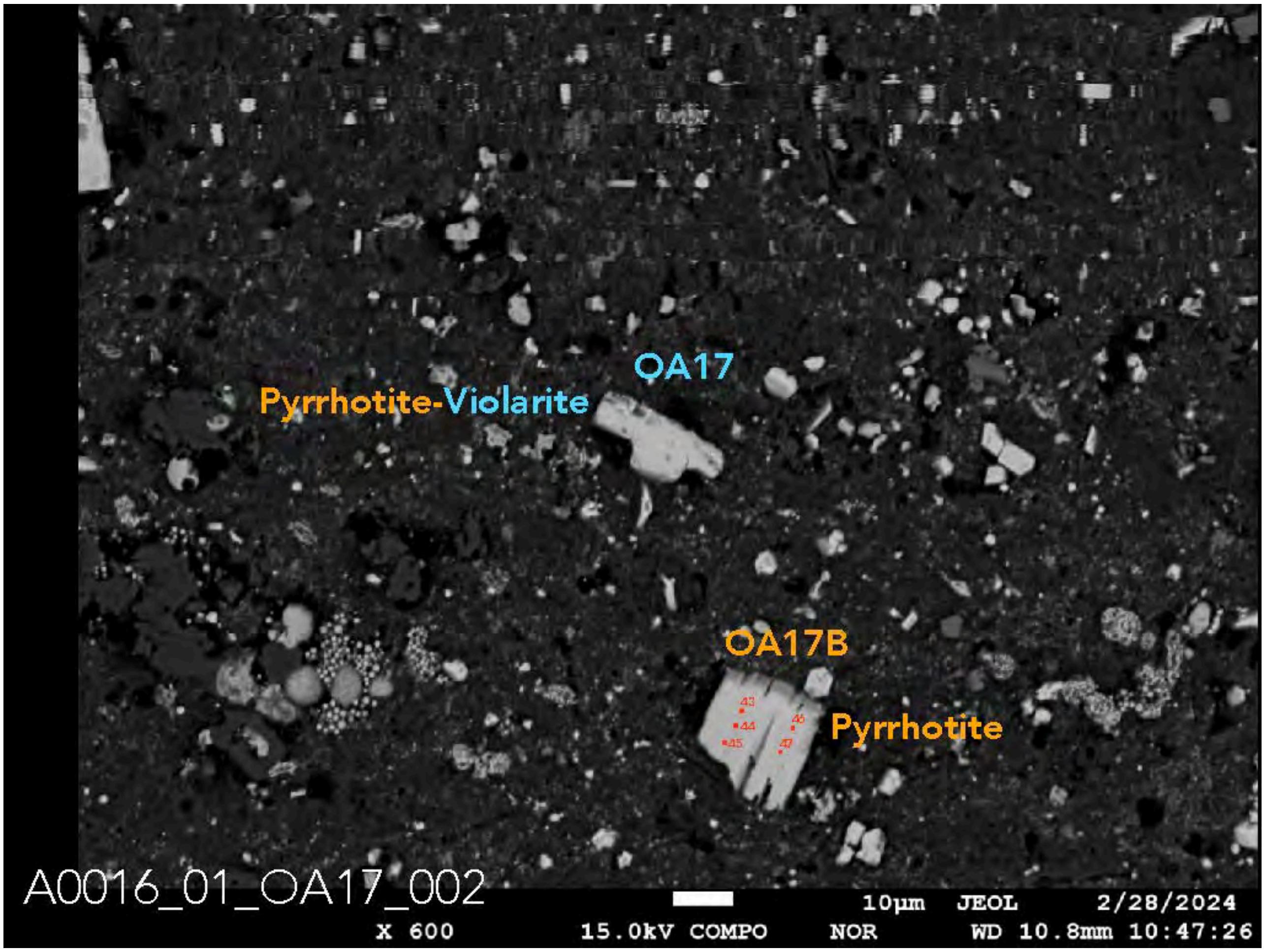

Supplementary Figure 57. Backscattered electron (BSE) image of OA17 and OA17B in A0016, with spot analyses marked. OA = opaque assemblage.

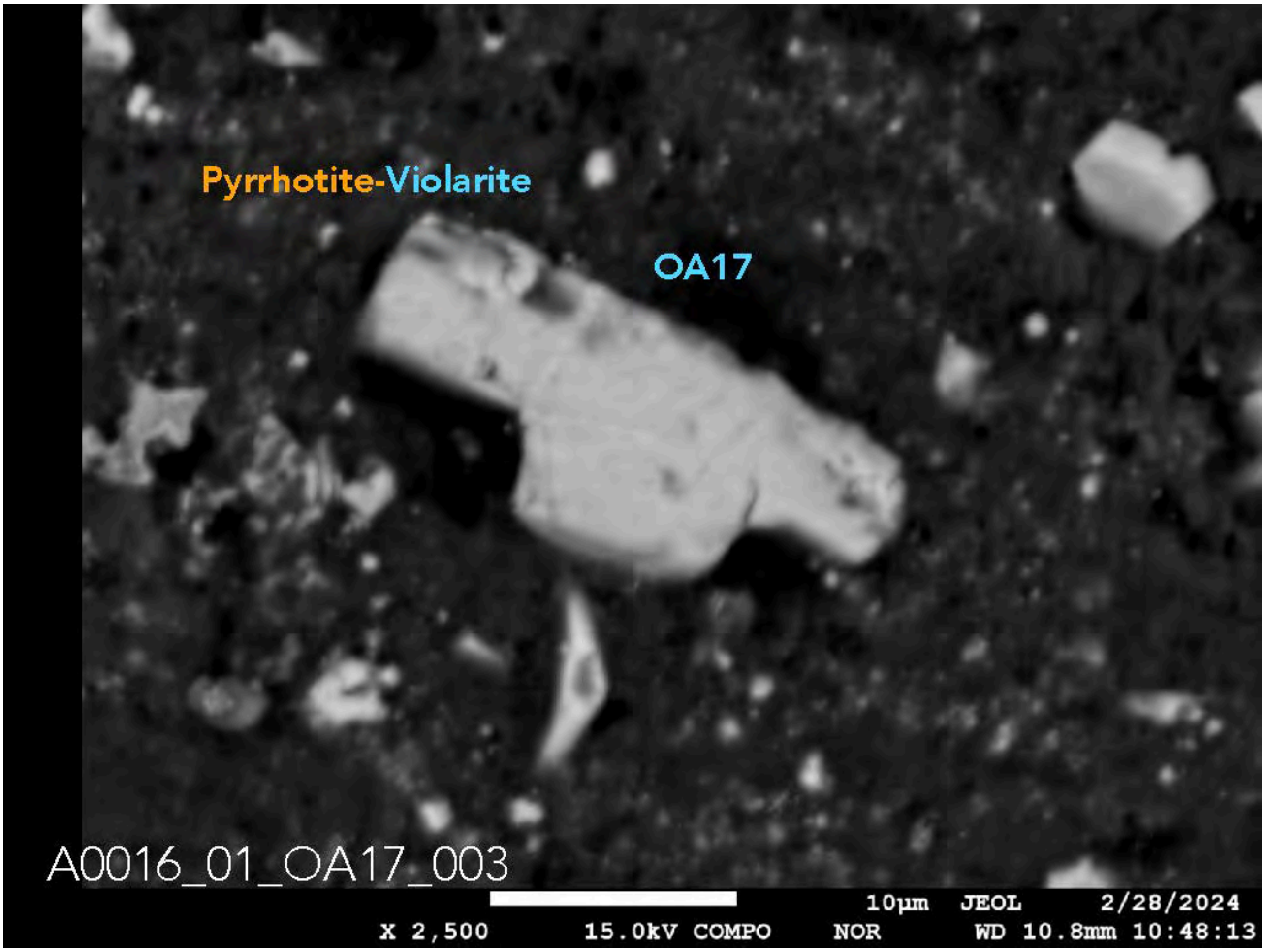

Supplementary Figure 58. Backscattered electron (BSE) image of OA17. OA = opaque assemblage.

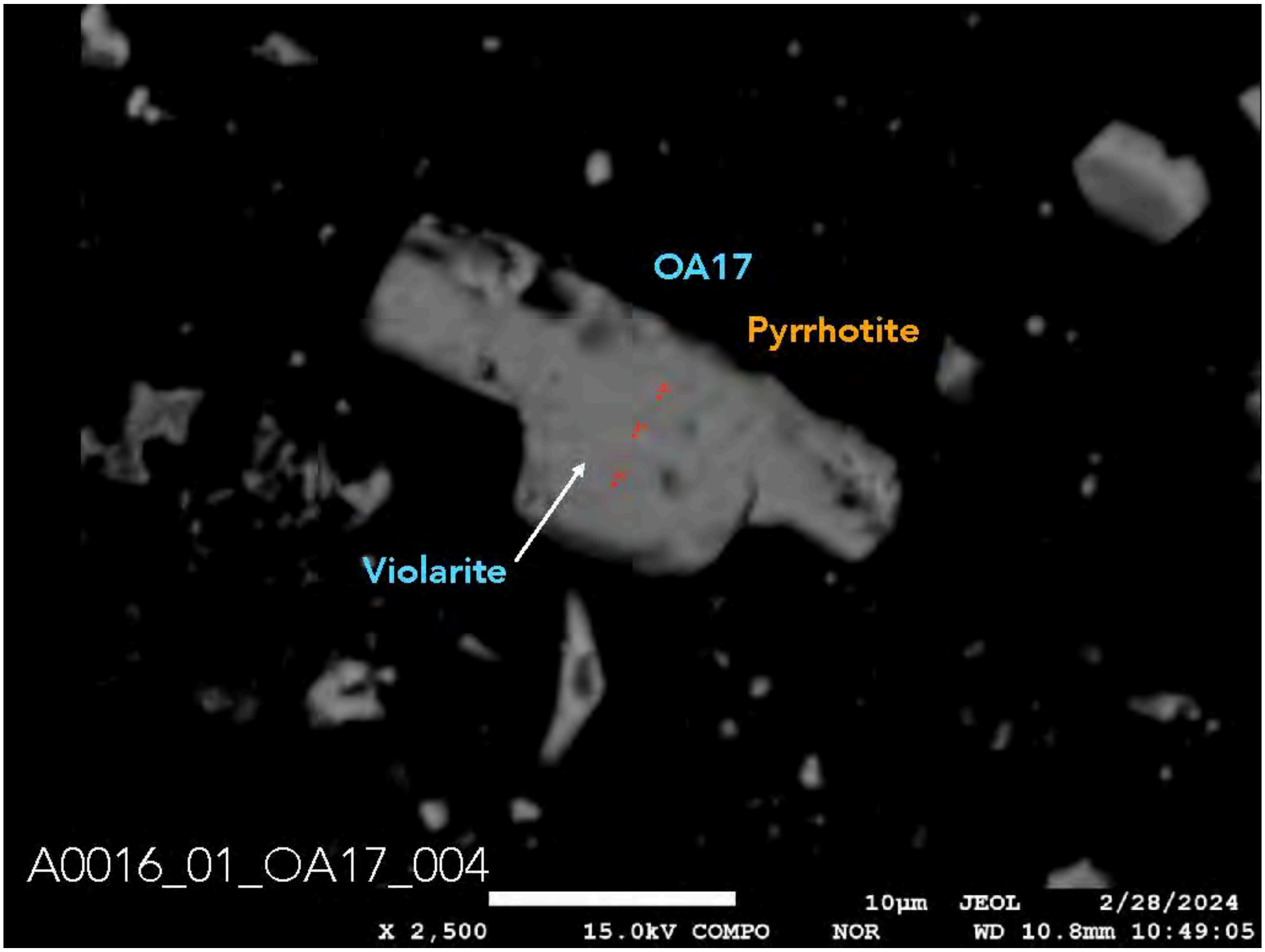

Supplementary Figure 59. Backscattered electron (BSE) image of OA17 in A0016, with spot analyses marked. OA = opaque assemblage.

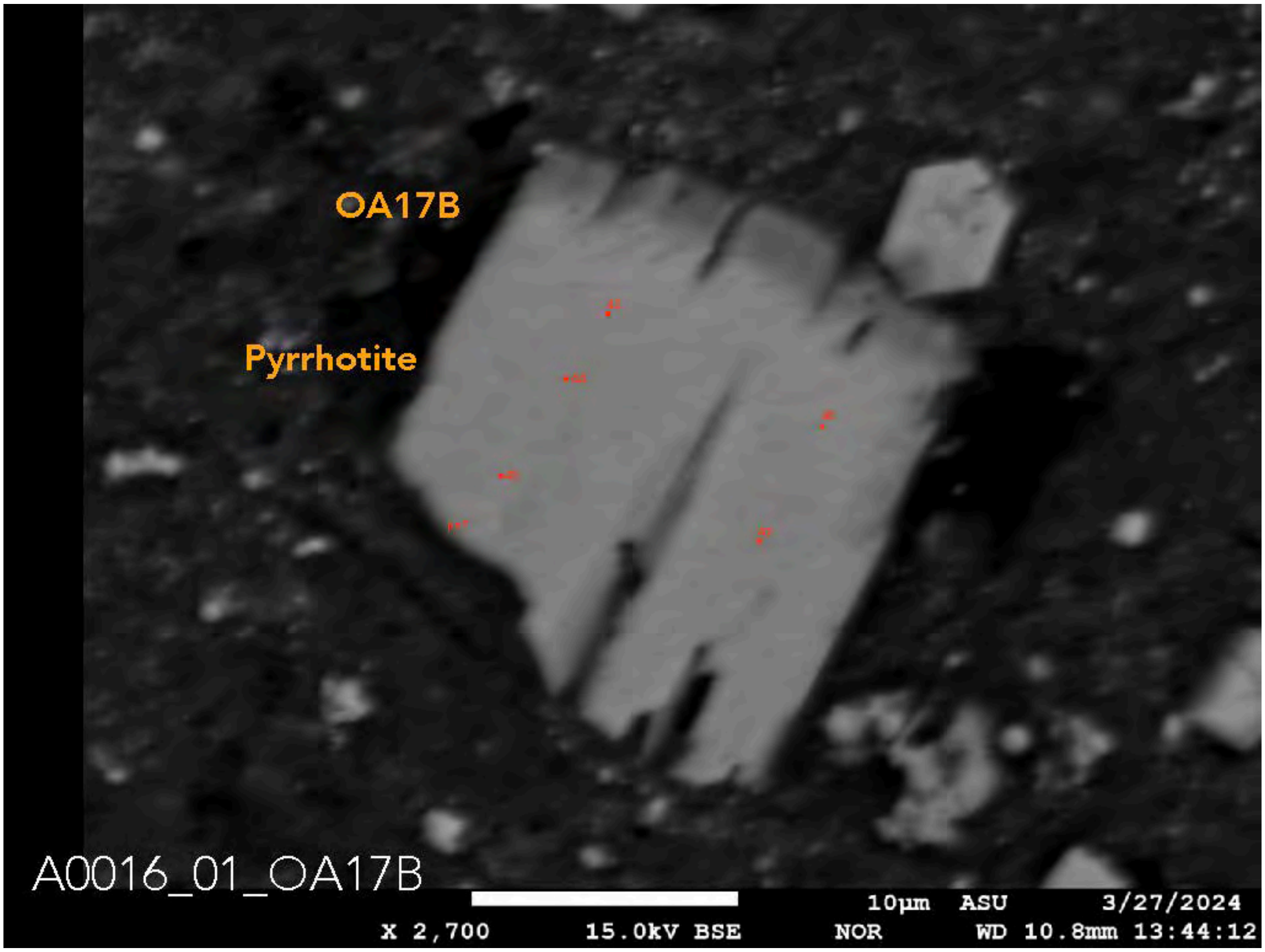

Supplementary Figure 60. Backscattered electron (BSE) image of OA17B in A0016, with spot analyses marked. OA = opaque assemblage.

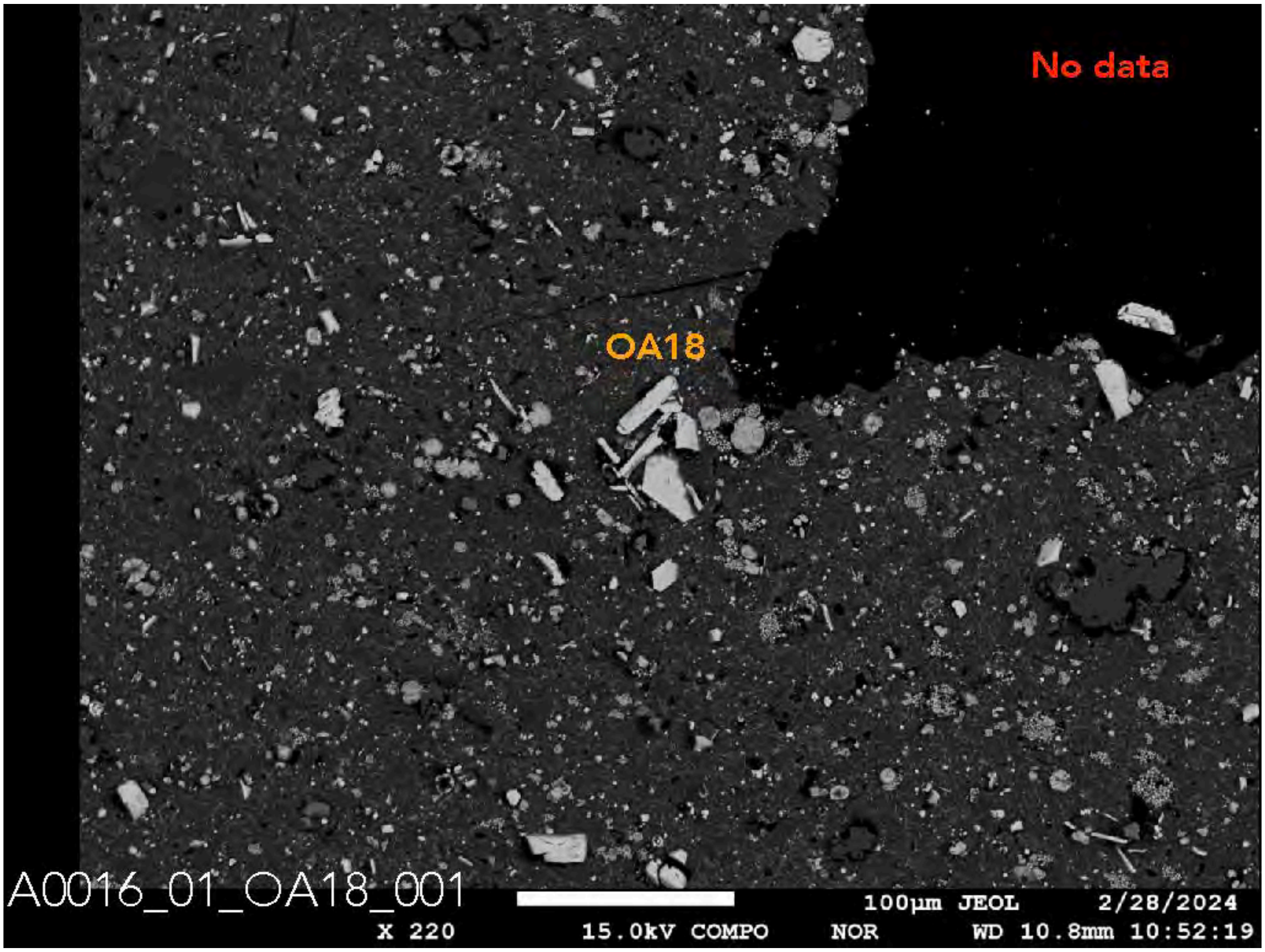

Supplementary Figure 61. Backscattered electron (BSE) image of OA18 in A0016. OA = opaque assemblage.

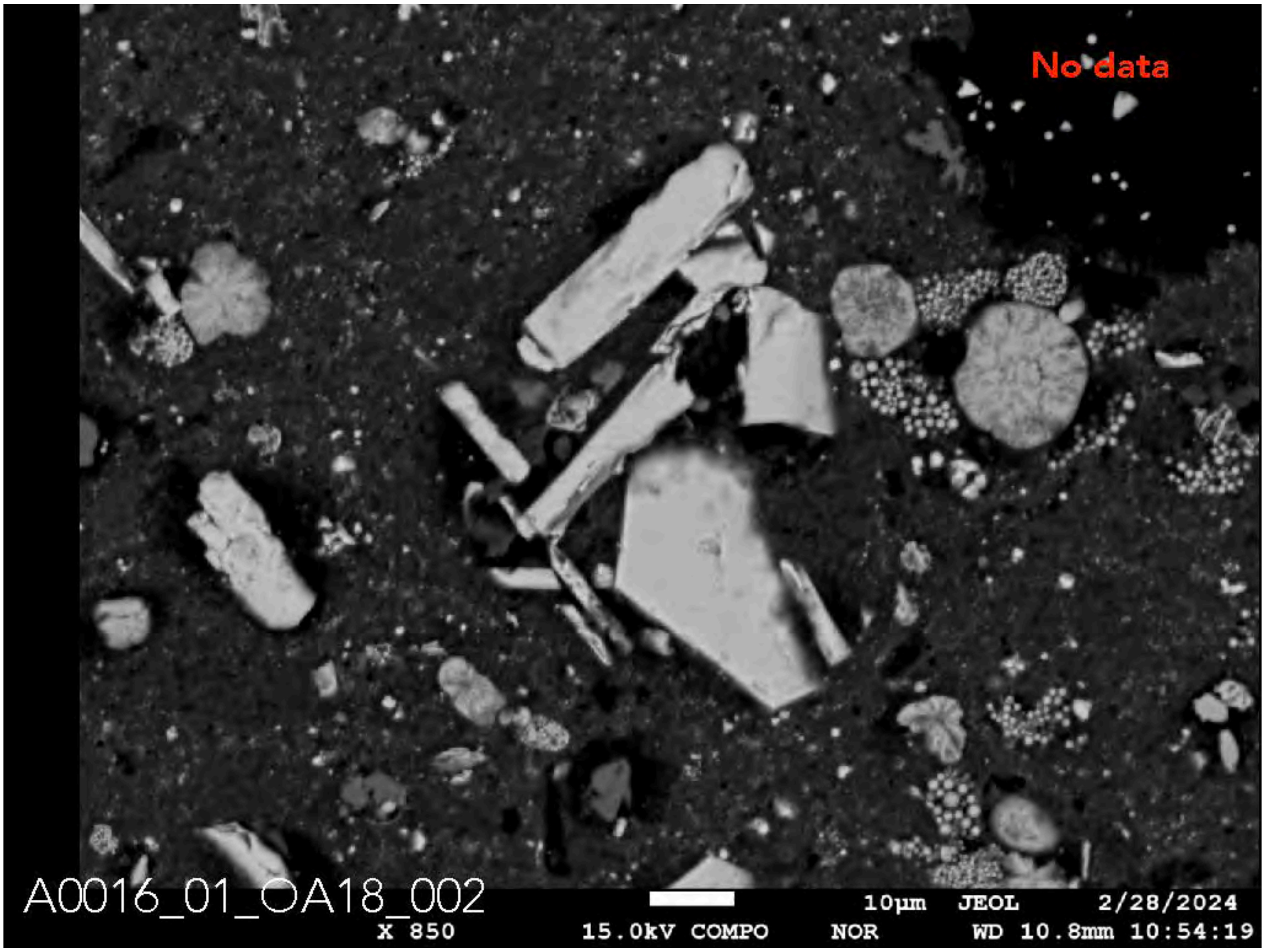

Supplementary Figure 62. Backscattered electron (BSE) image of OA18 in A0016. OA = opaque assemblage.

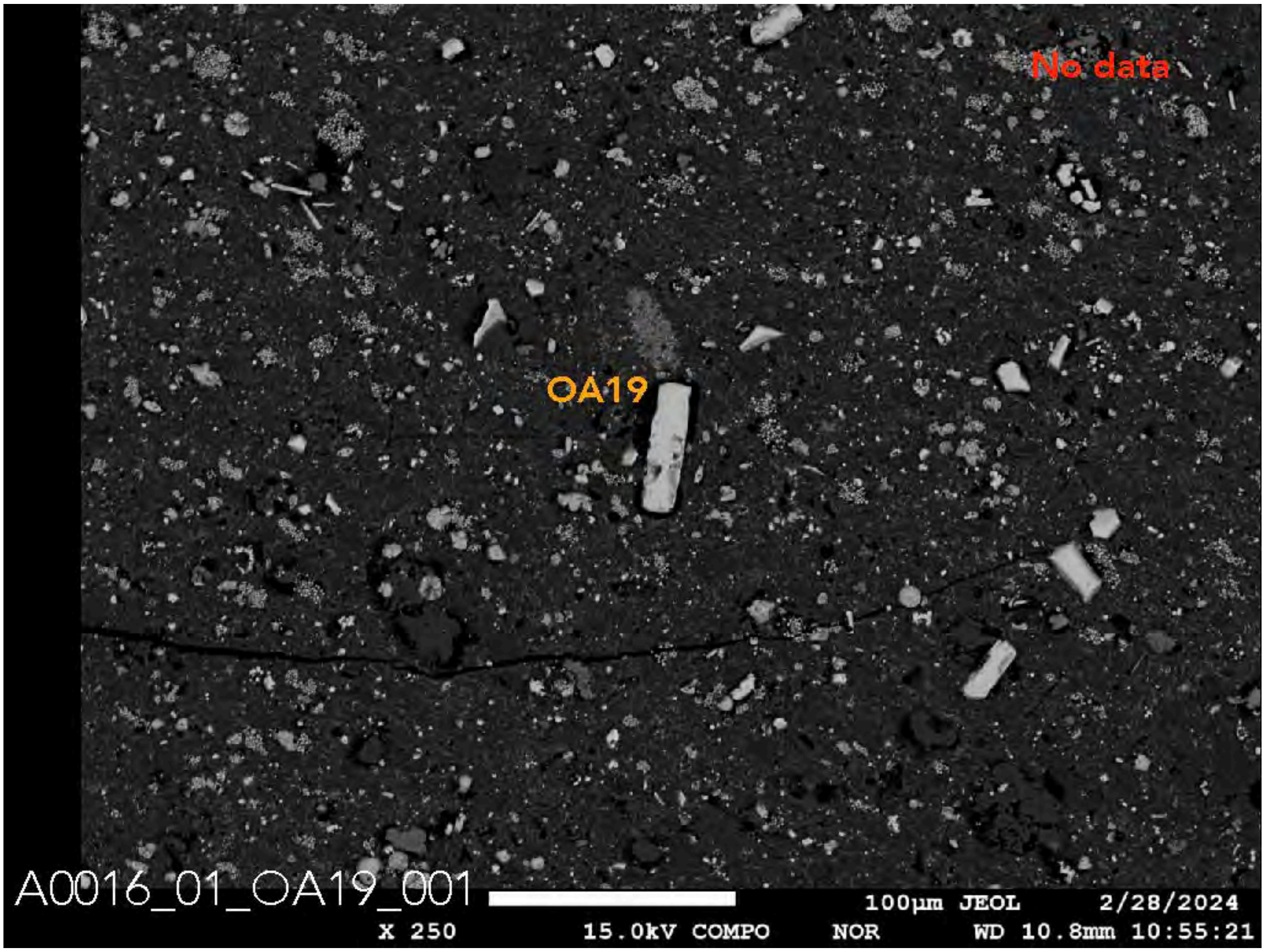

Supplementary Figure 63. Backscattered electron (BSE) image of OA19 in A0016. OA = opaque assemblage.

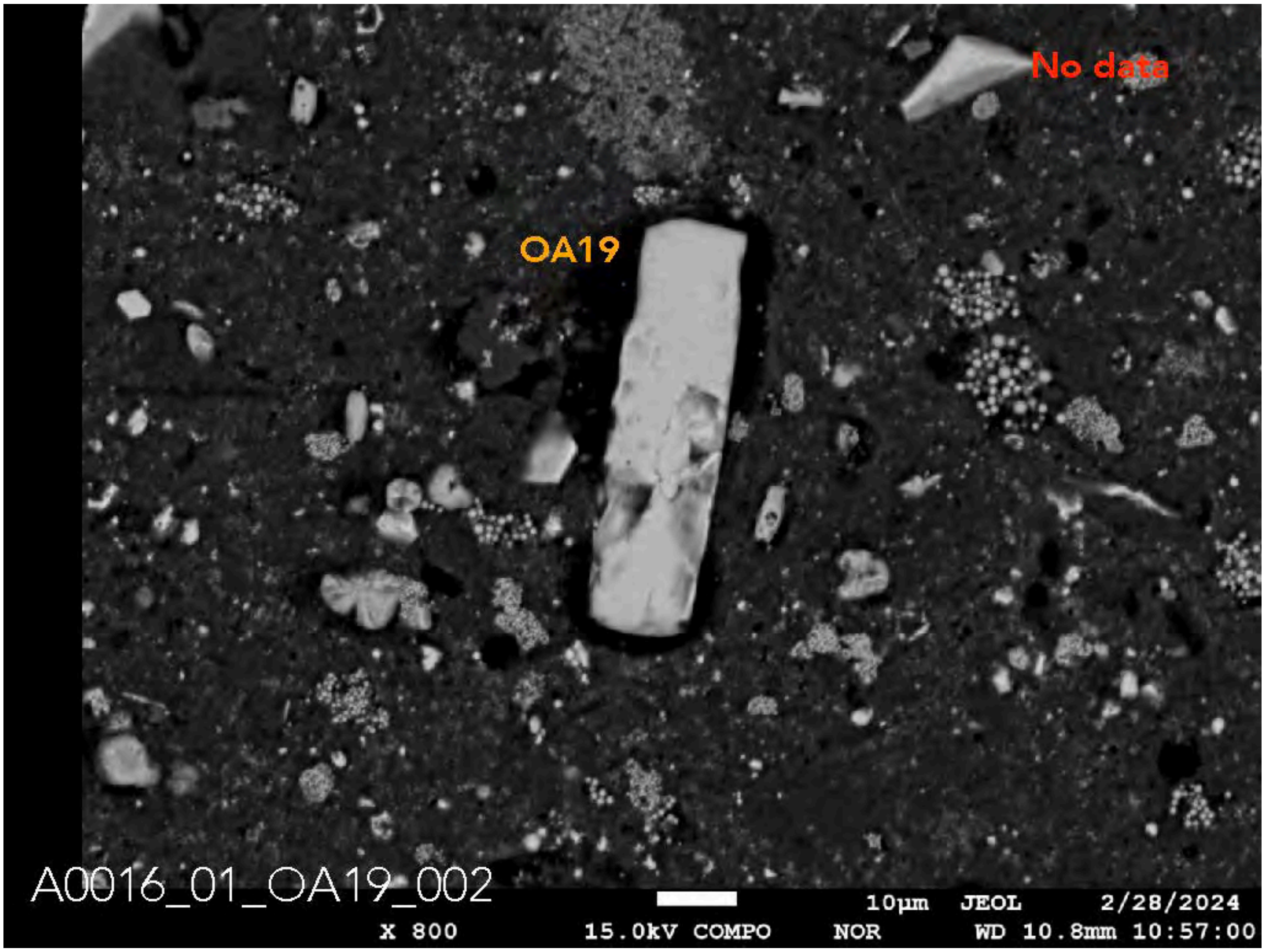

Supplementary Figure 64. Backscattered electron (BSE) image of OA19 in A0016. OA = opaque assemblage.

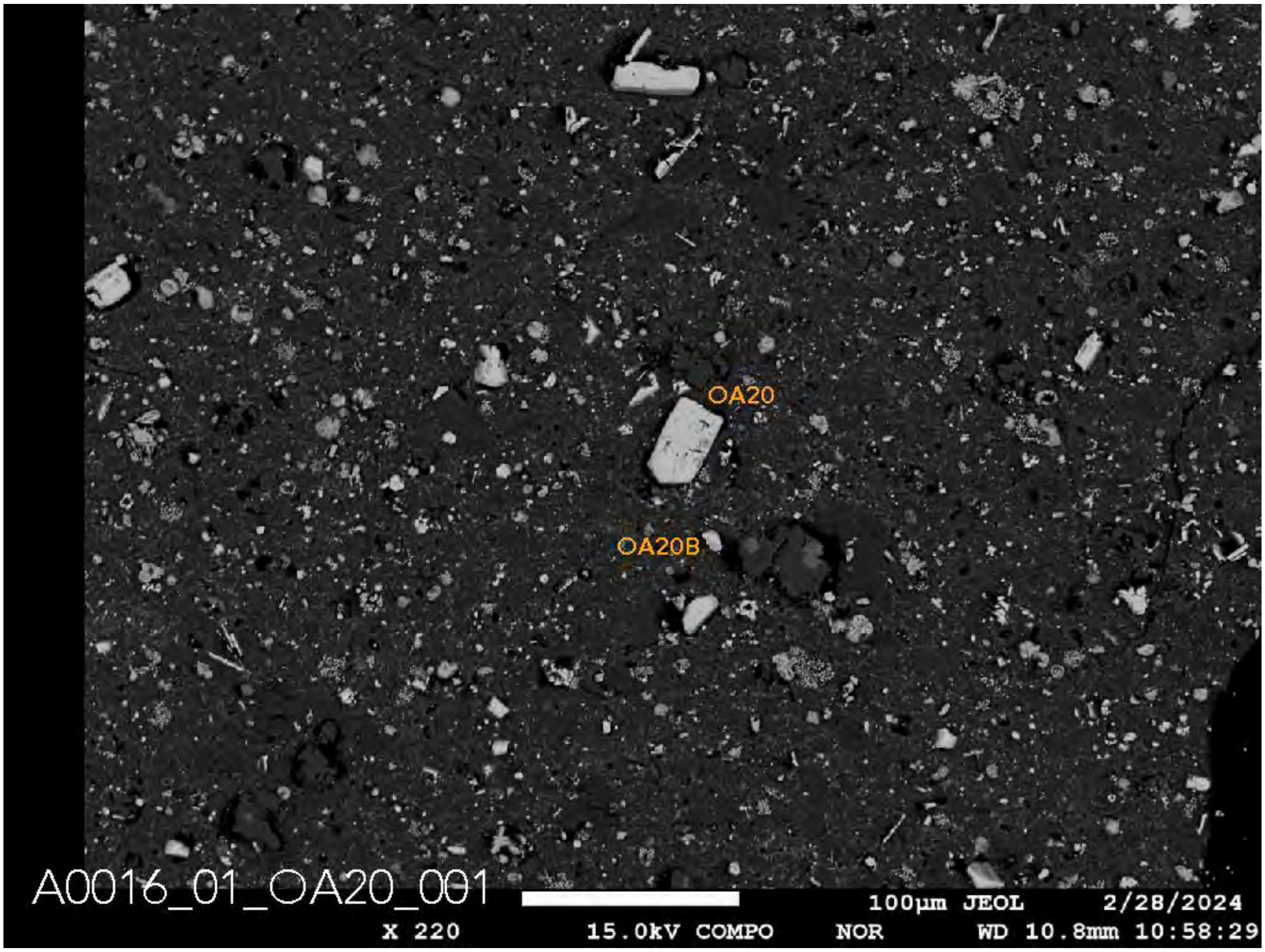

Supplementary Figure 65. Backscattered electron (BSE) image of OA20 and OA20B in A0016. OA = opaque assemblage.

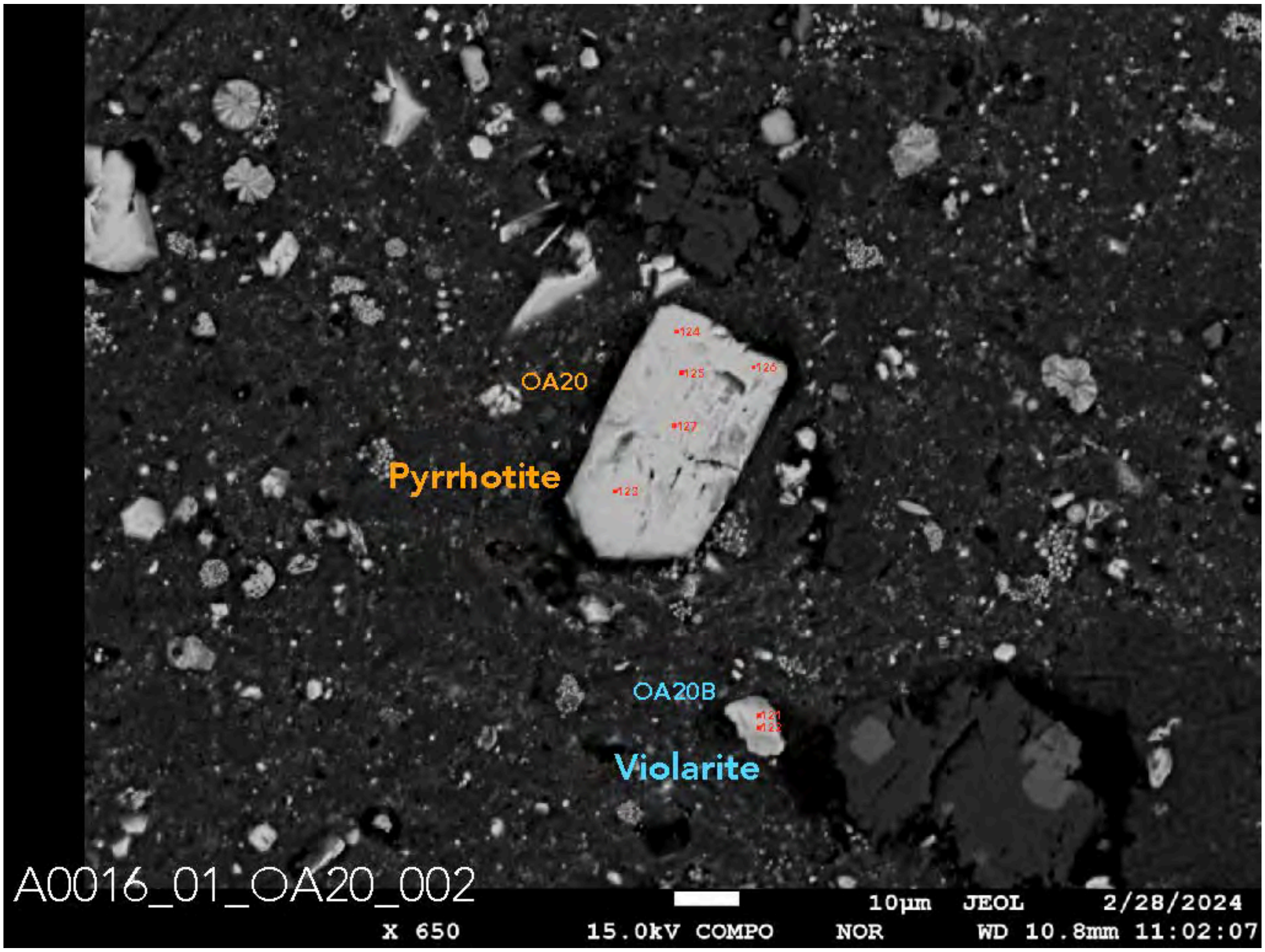

Supplementary Figure 66. Backscattered electron (BSE) image of OA20 and OA20B in A0016, with spot analyses marked. OA = opaque assemblage.

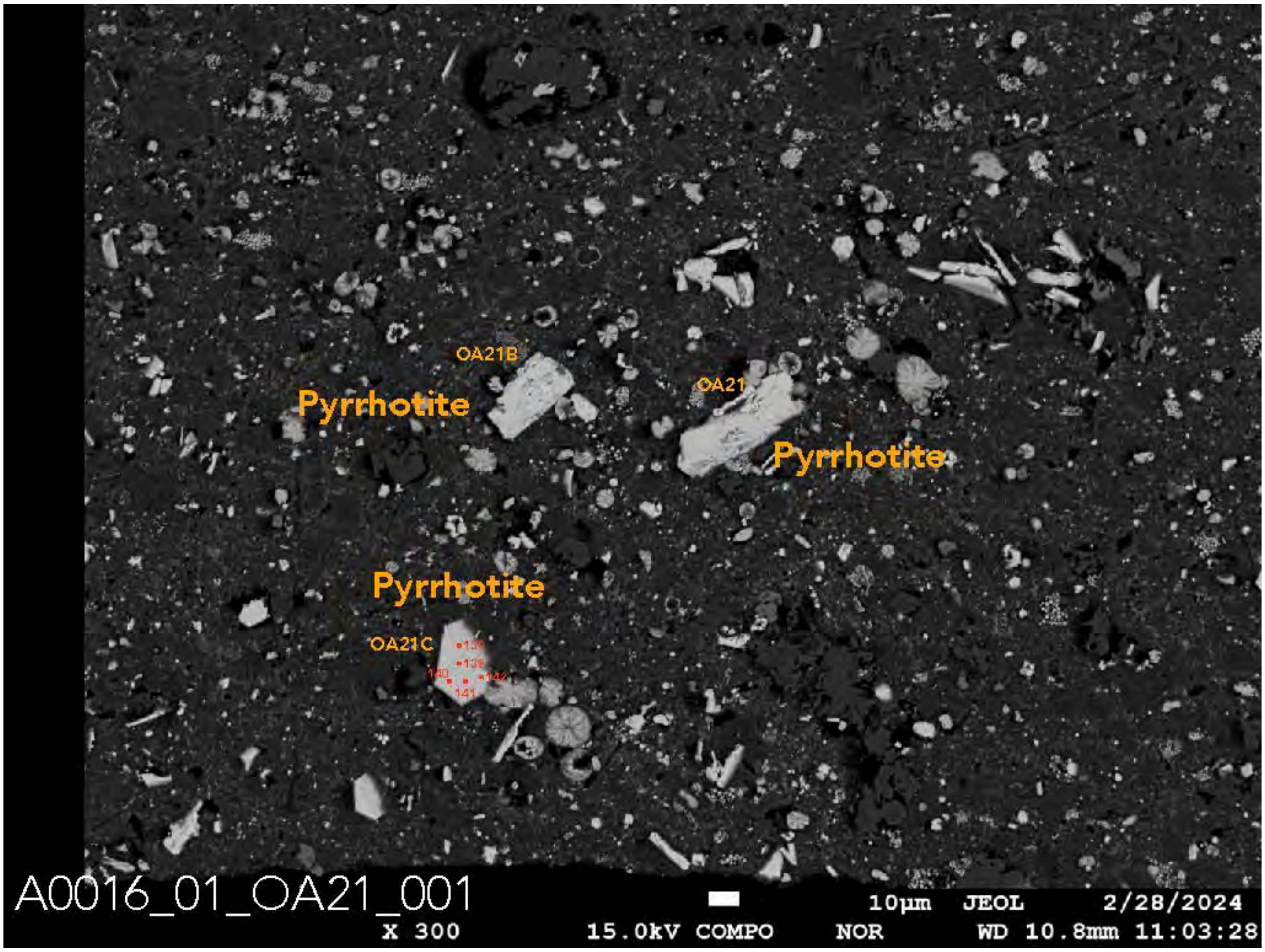

Supplementary Figure 67. Backscattered electron (BSE) image of OA21, OA21B, and OA21C in A0016, with spot analyses marked. OA = opaque assemblage.

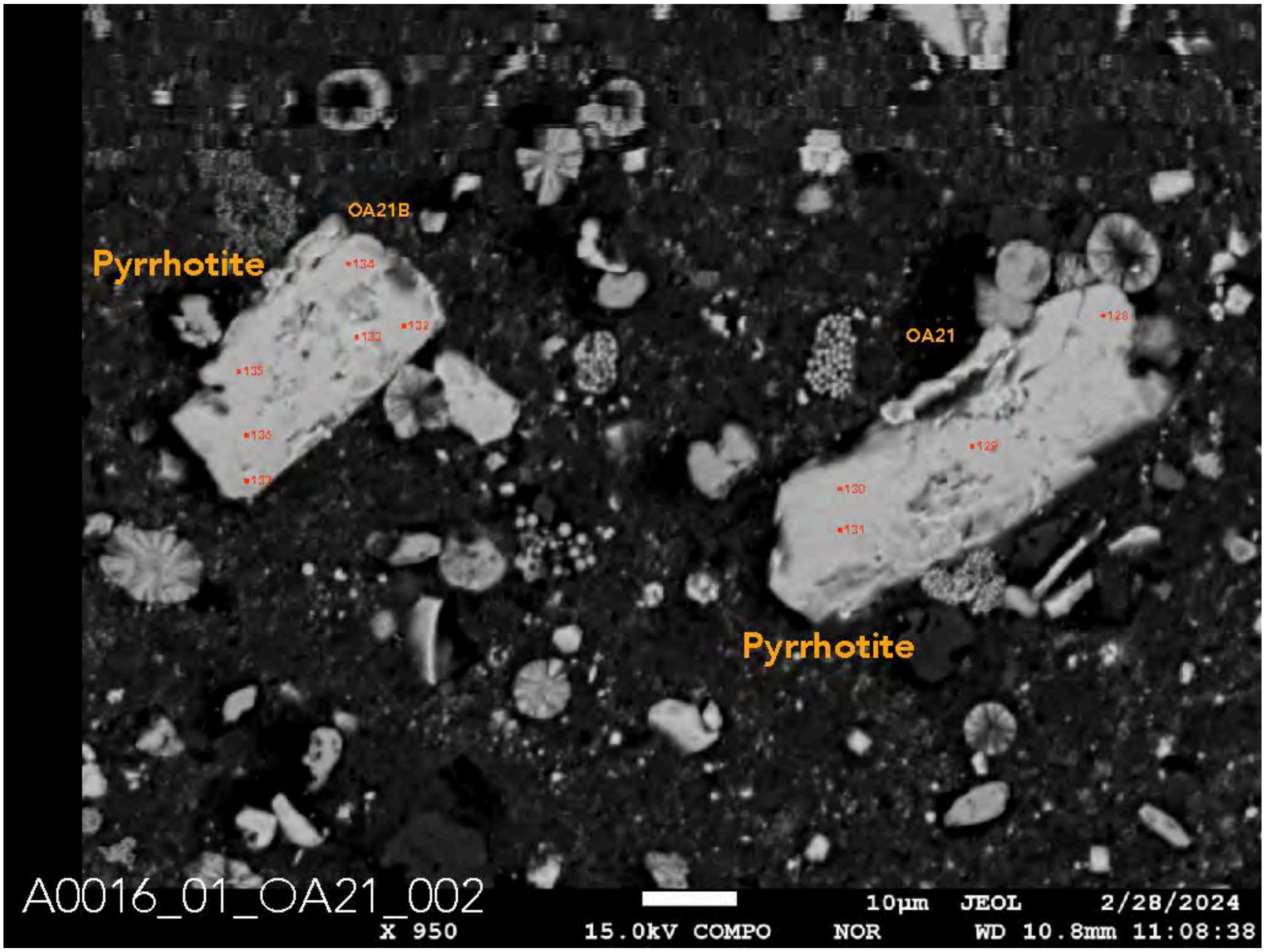

Supplementary Figure 68. Backscattered electron (BSE) image of OA21 and OA21B in A0016, with spot analyses marked. OA = opaque assemblage.

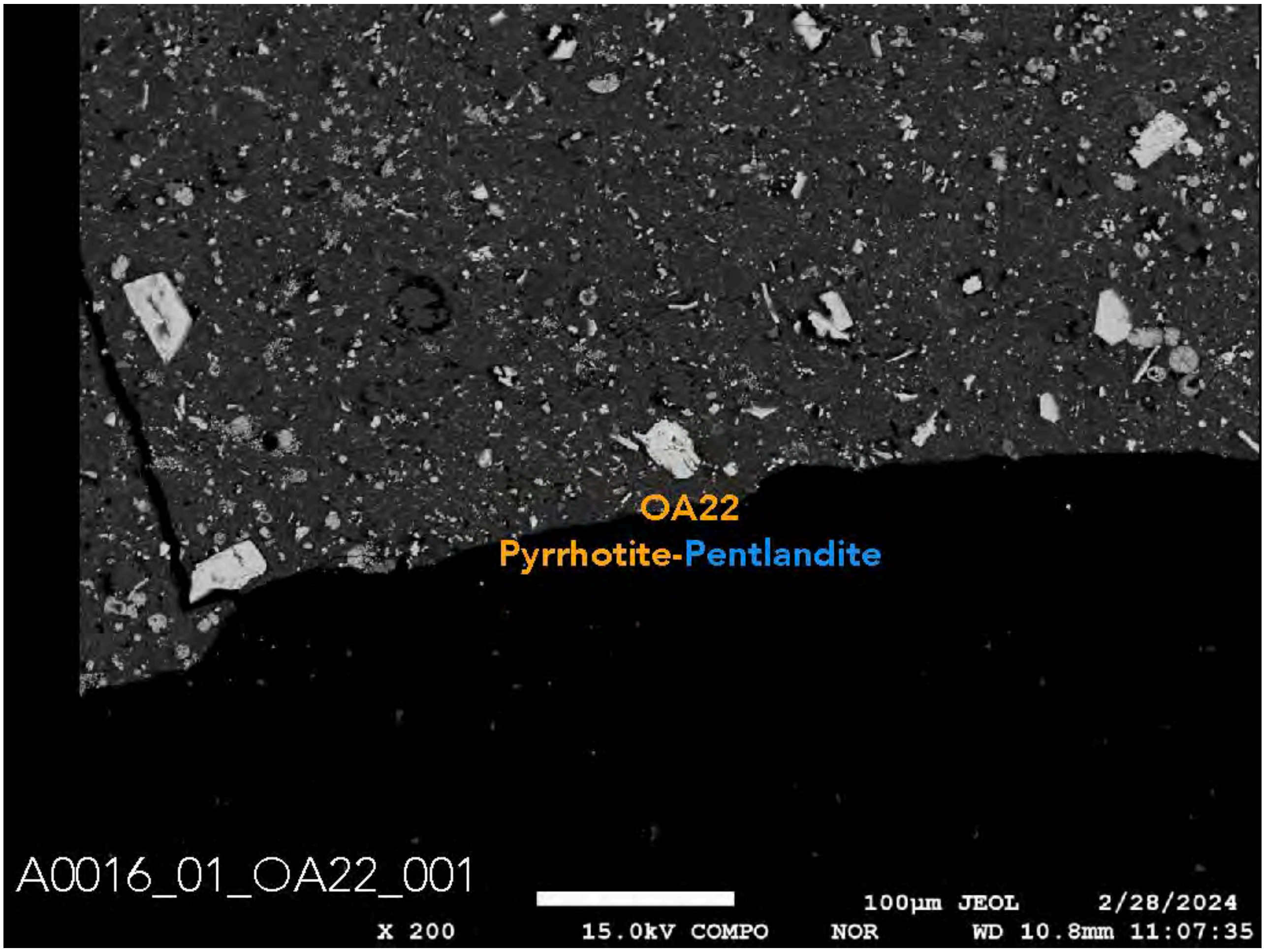

Supplementary Figure 69. Backscattered electron (BSE) image of OA22 in A0016. OA = opaque assemblage.

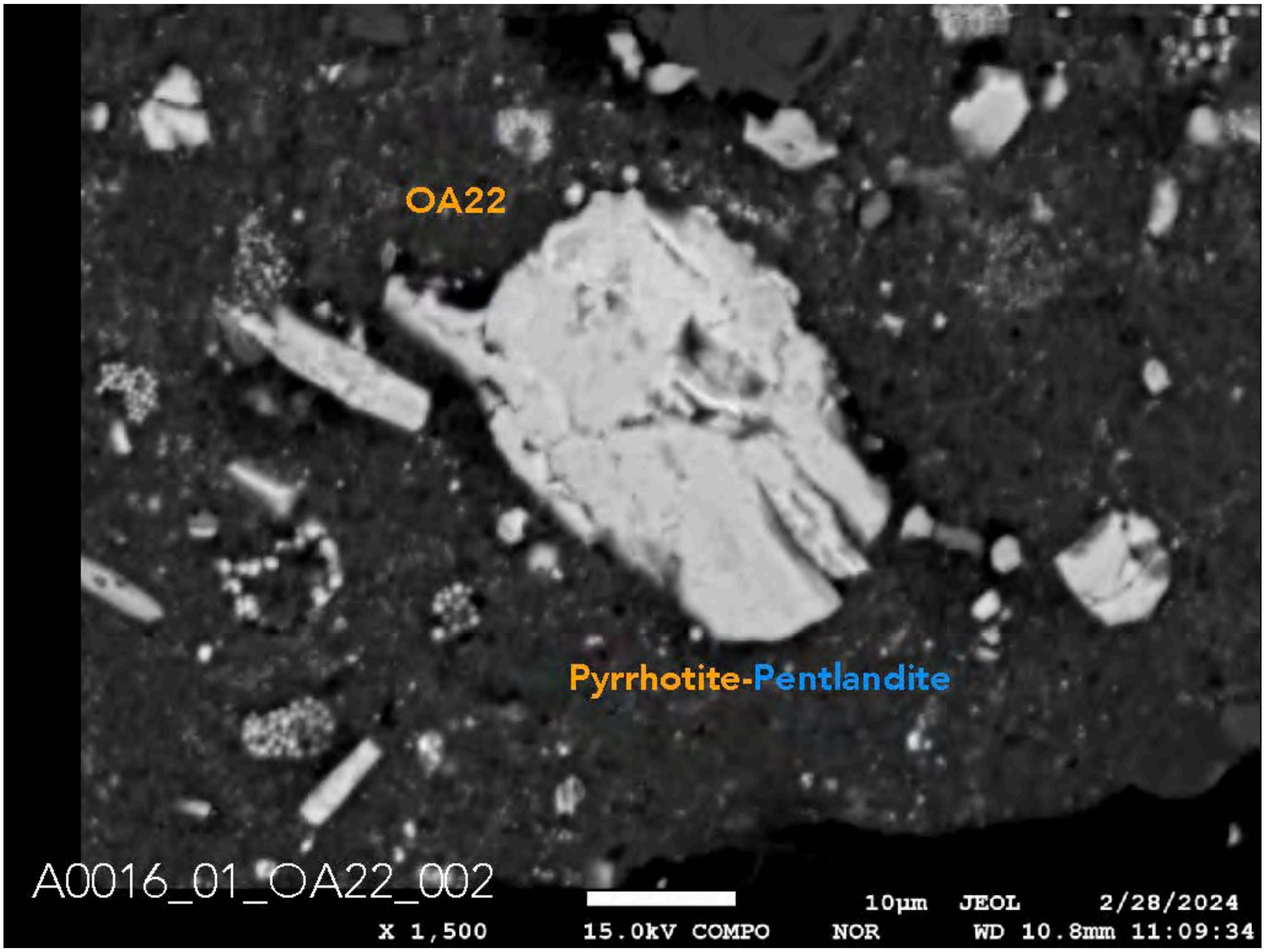

Supplementary Figure 70. Backscattered electron (BSE) image of OA22 in A0016. OA = opaque assemblage.

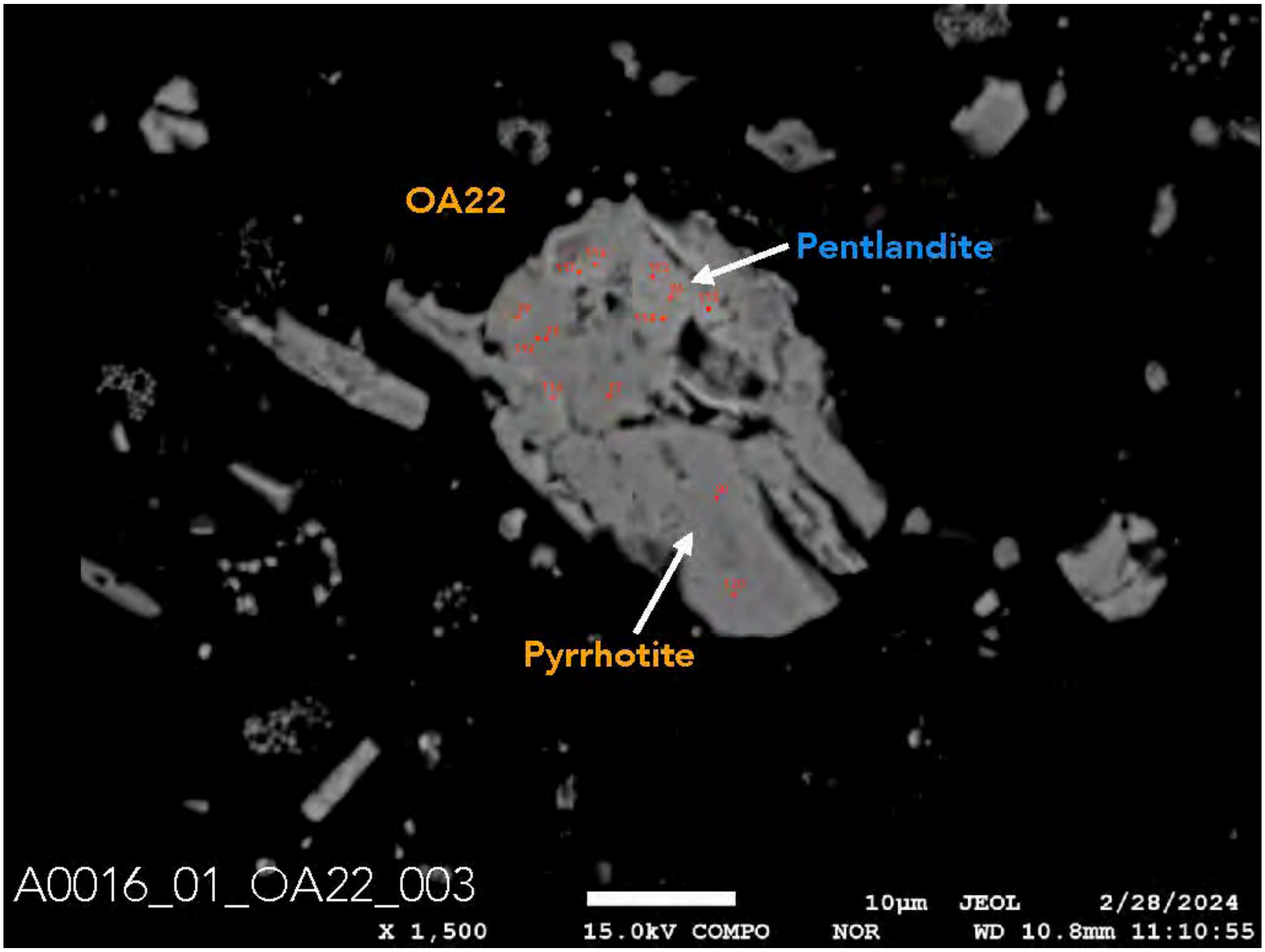

Supplementary Figure 71. Backscattered electron (BSE) image of OA22 in A0016, with spot analyses marked. OA = opaque assemblage.

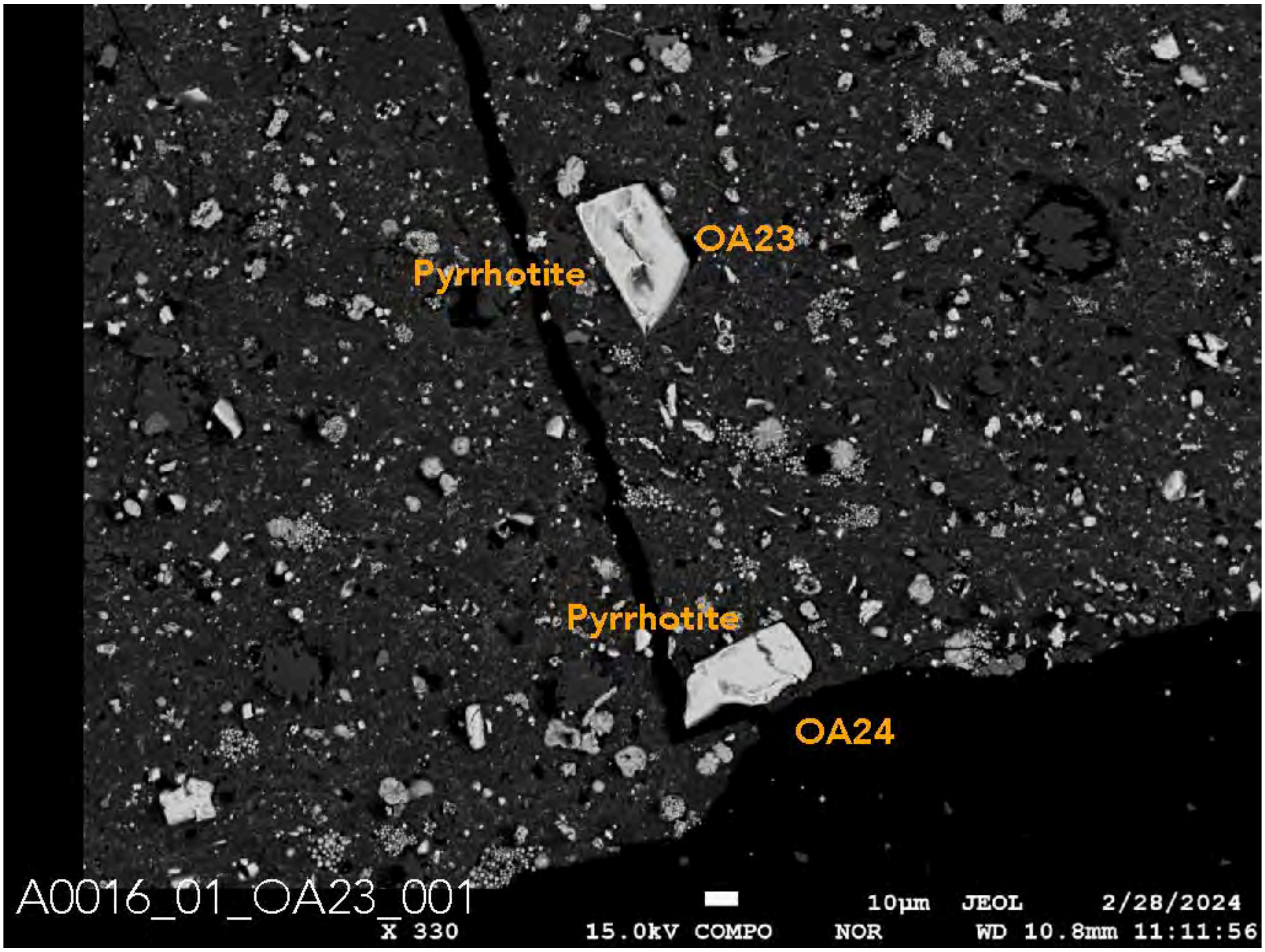

Supplementary Figure 72. Backscattered electron (BSE) image of OA23 and OA24 in A0016. OA = opaque assemblage.

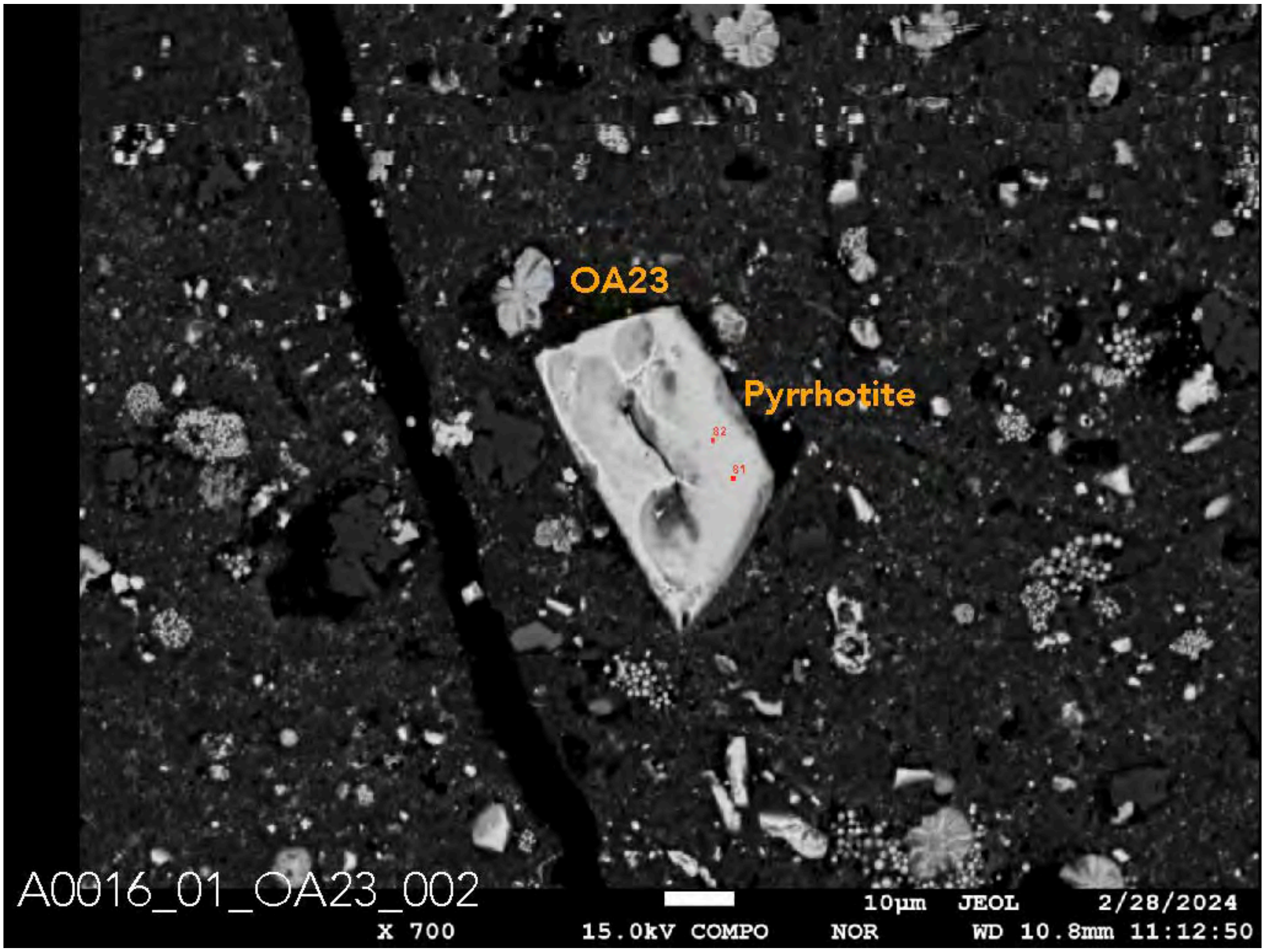

Supplementary Figure 73. Backscattered electron (BSE) image of OA23 in A0016, with spot analyses marked. OA = opaque assemblage.

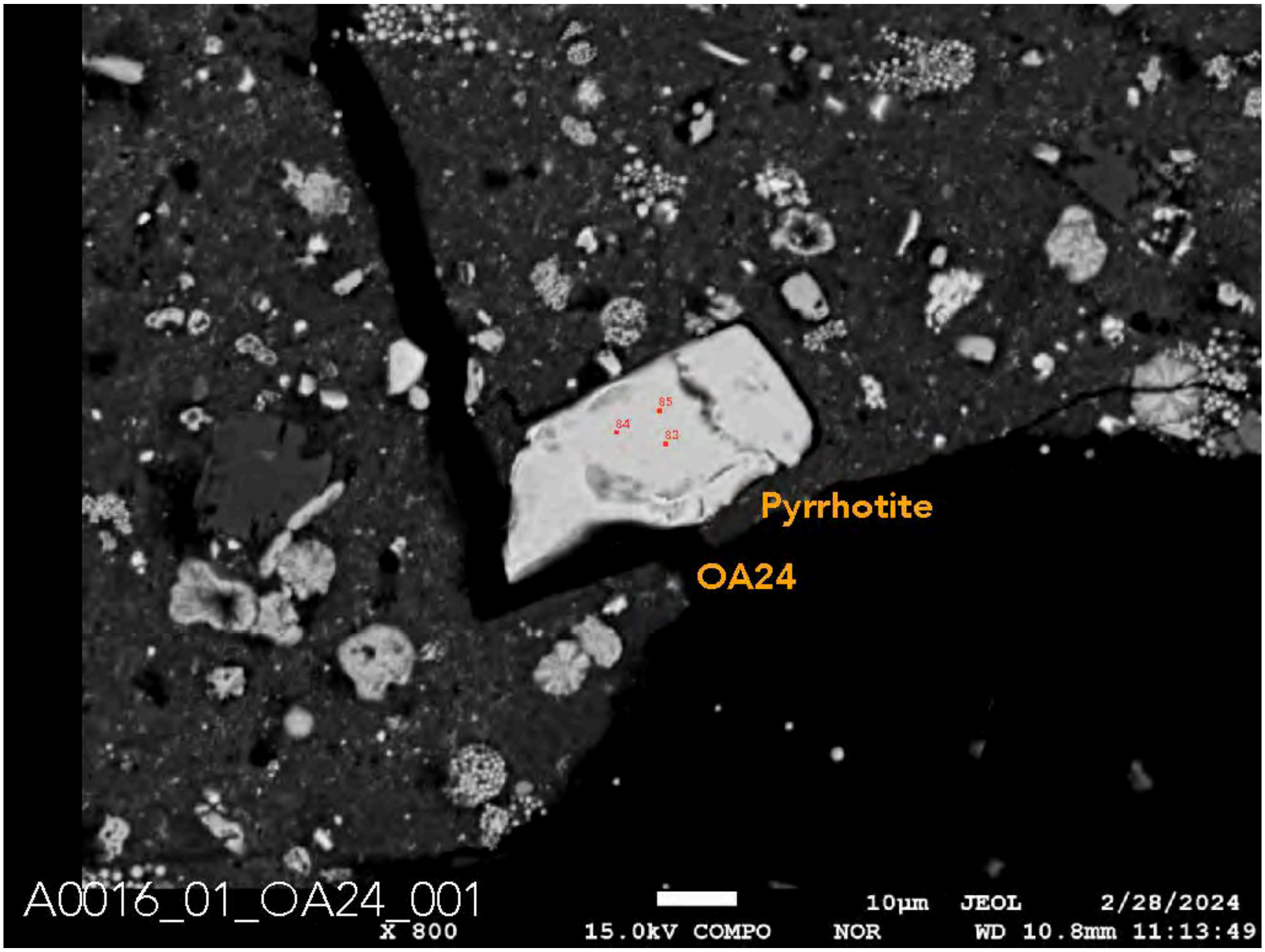

Supplementary Figure 74. Backscattered electron (BSE) image of OA24 in A0016, with spot analyses marked. OA = opaque assemblage.

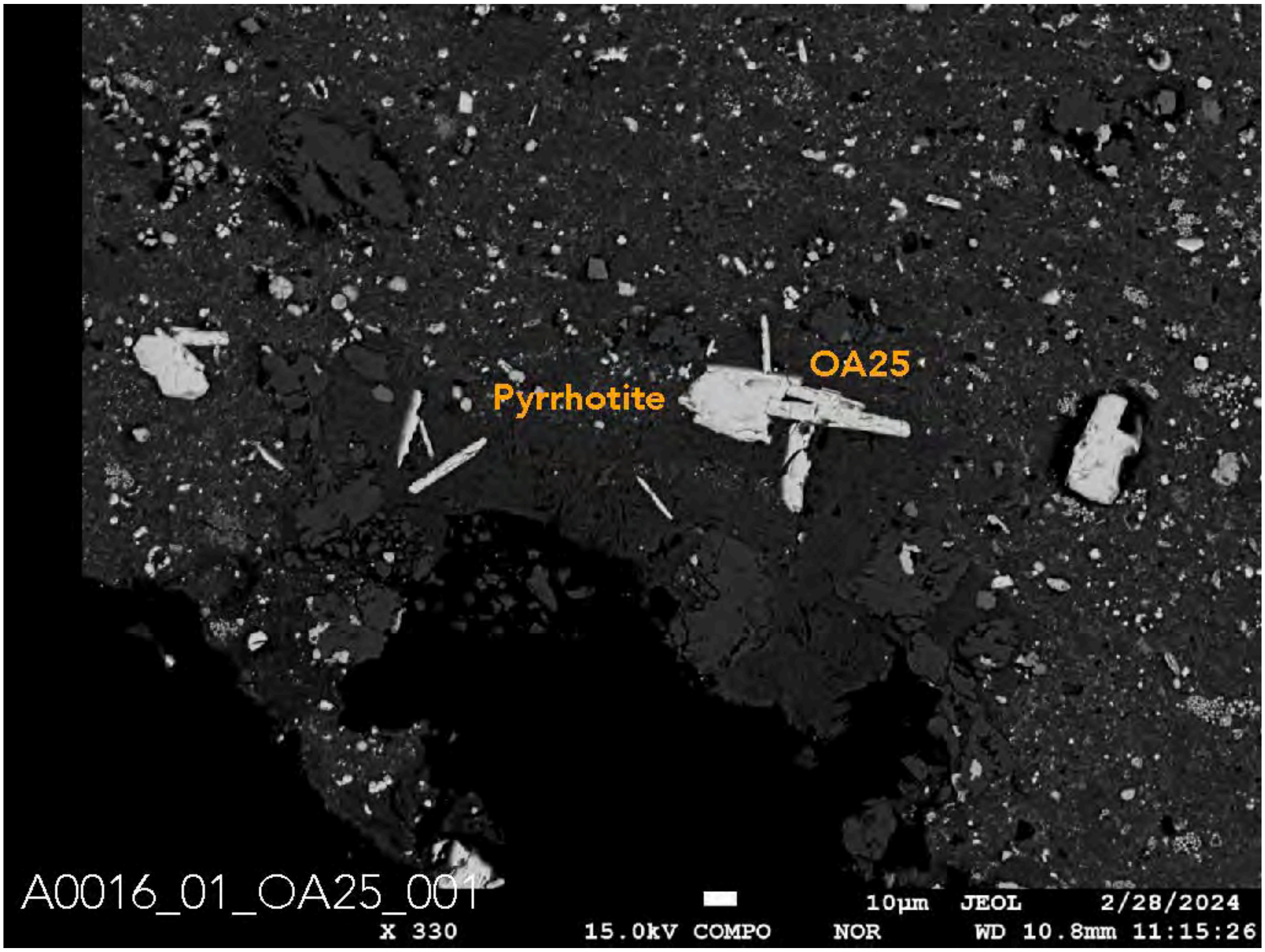

Supplementary Figure 75. Backscattered electron (BSE) image of OA25 in A0016. OA = opaque assemblage.

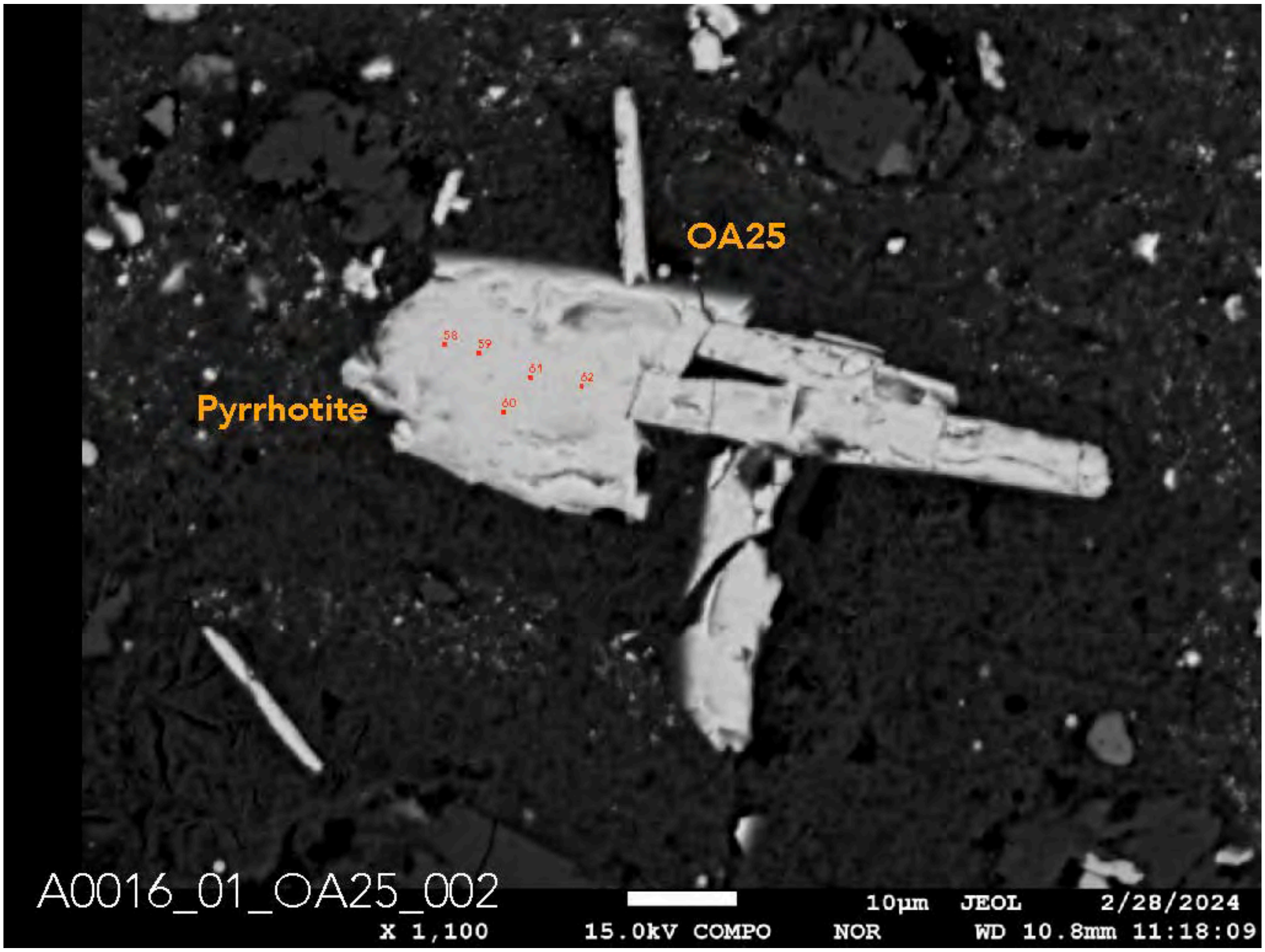

Supplementary Figure 76. Backscattered electron (BSE) image of OA25 in A0016, with spot analyses marked. OA = opaque assemblage.

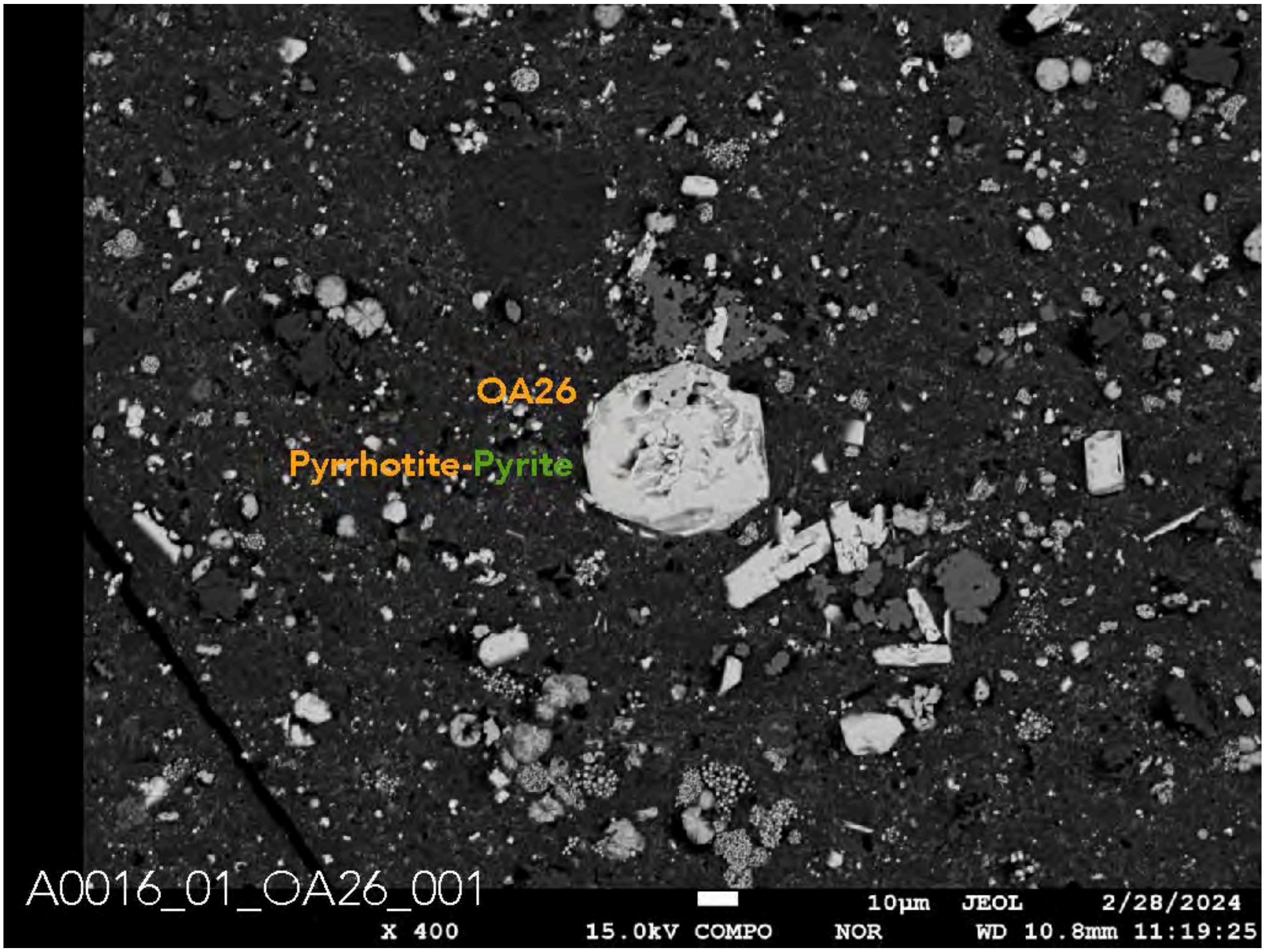

Supplementary Figure 77. Backscattered electron (BSE) image of OA26 in A0016. OA = opaque assemblage.

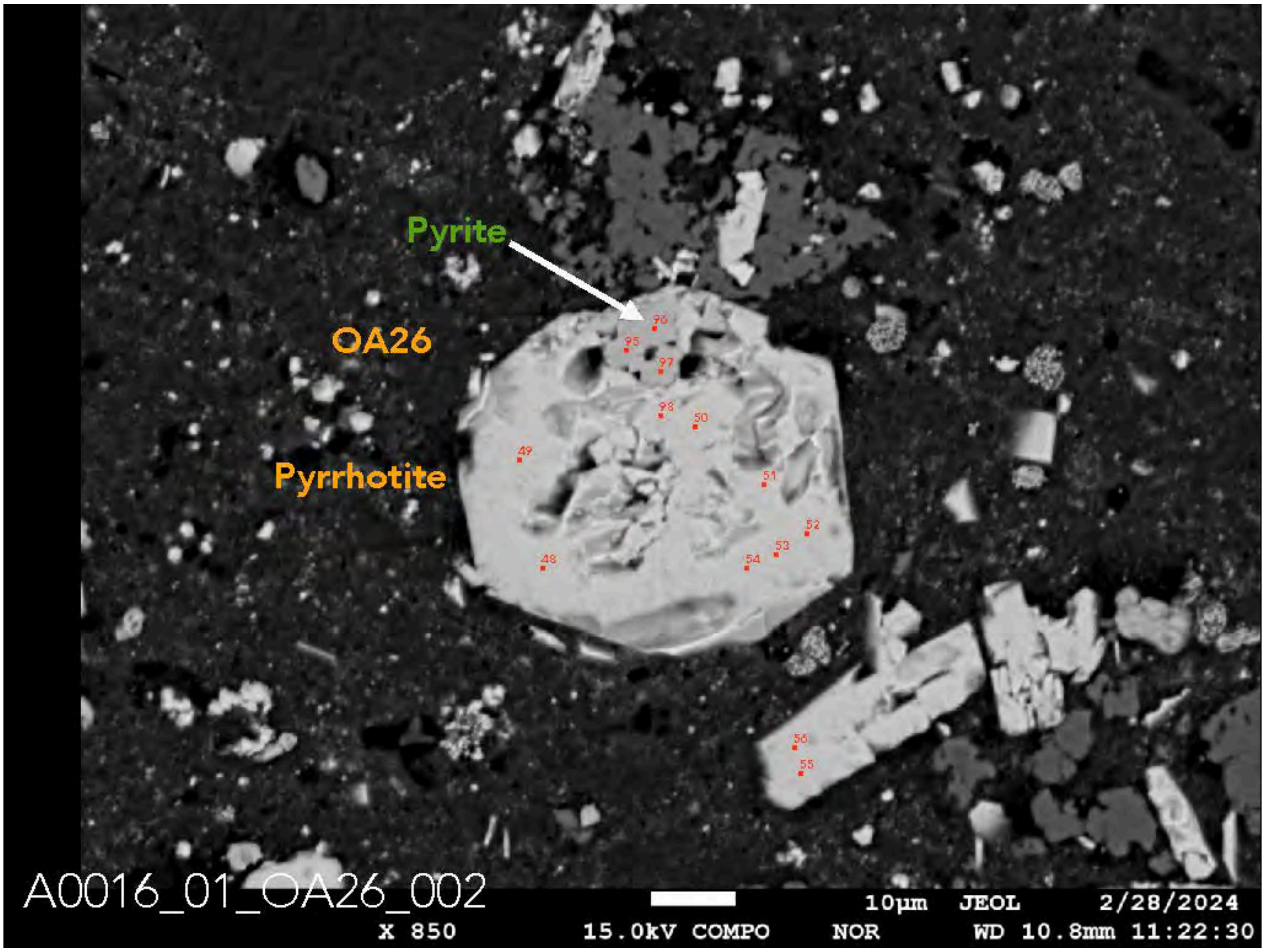

Supplementary Figure 78. Backscattered electron (BSE) image of OA26 in A0016, with spot analyses marked. OA = opaque assemblage.

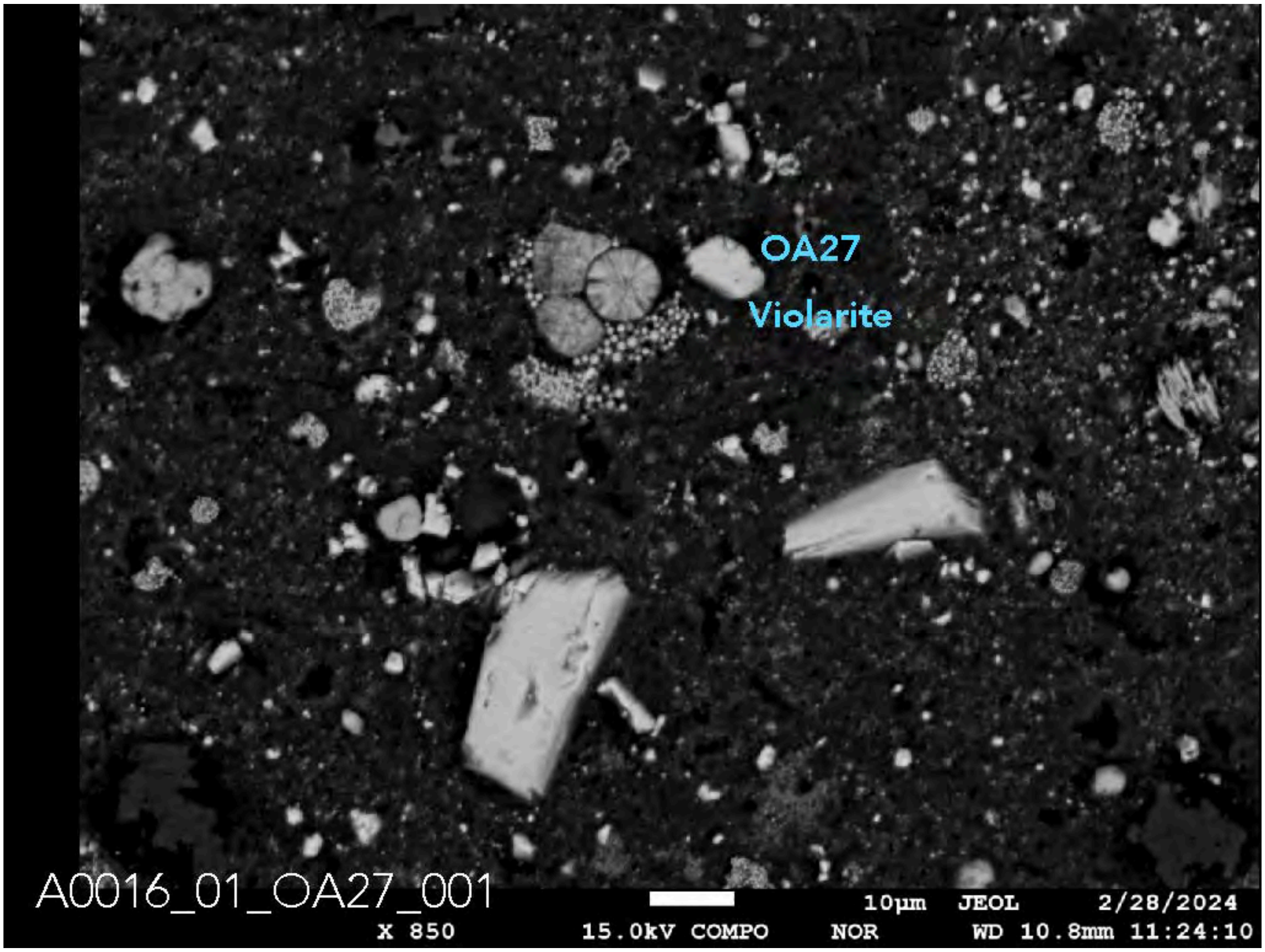

Supplementary Figure 79. Backscattered electron (BSE) image of OA27 in A0016. OA = opaque assemblage.

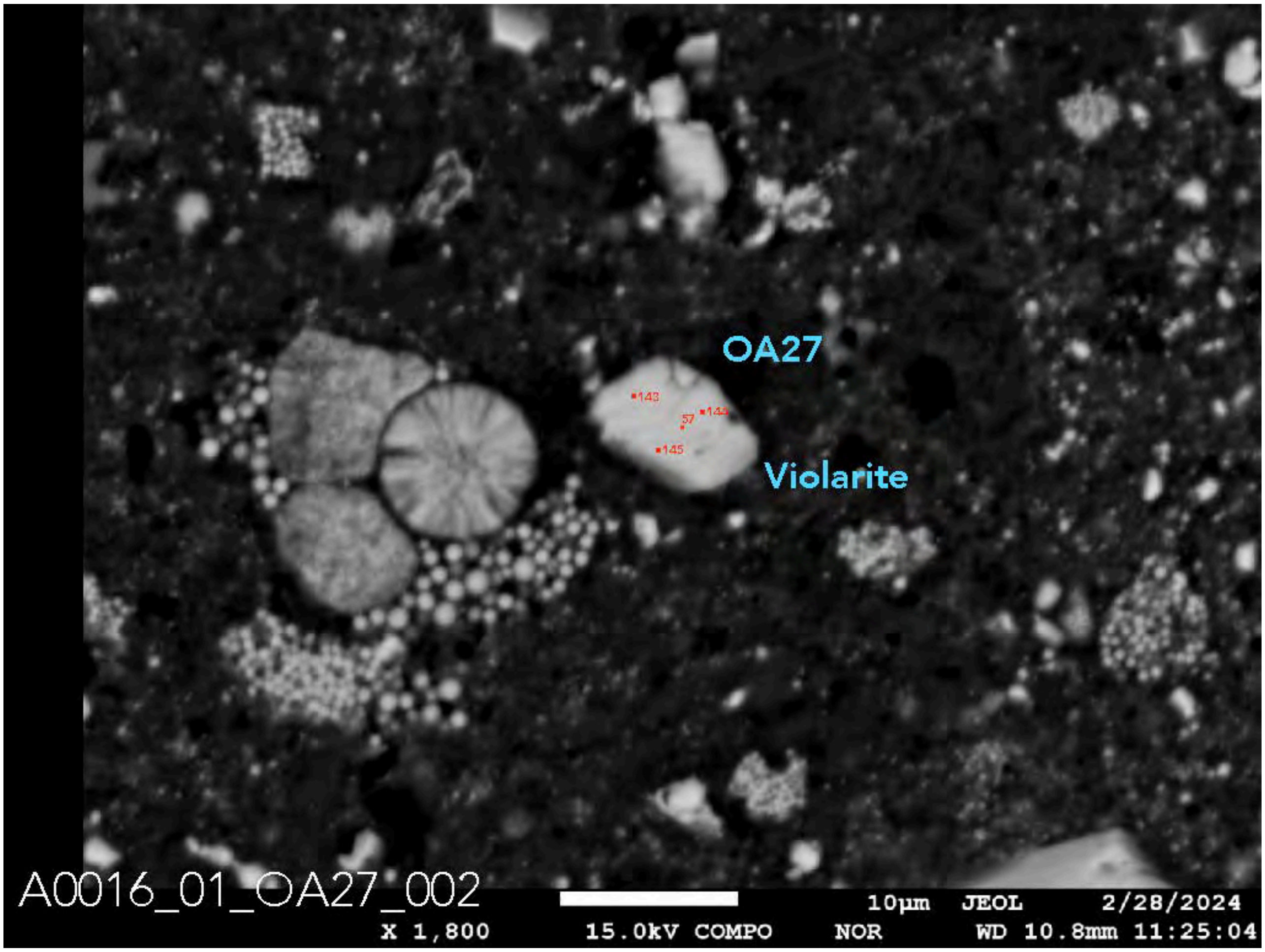

Supplementary Figure 80. Backscattered electron (BSE) image of OA27 in A0016, with spot analyses marked. OA = opaque assemblage.

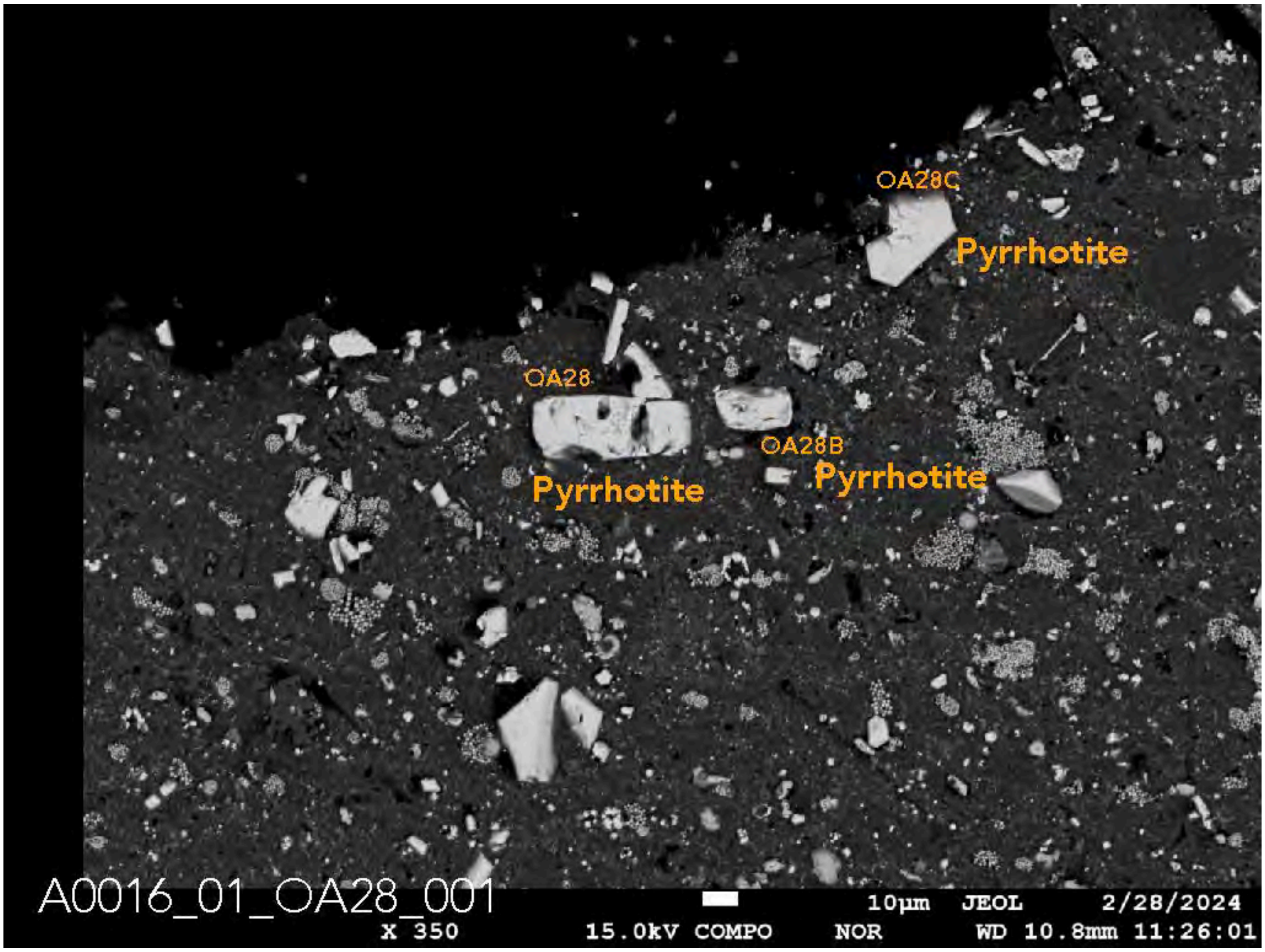

Supplementary Figure 81. Backscattered electron (BSE) image of OA28, OA28B, and OA28C in A0016. OA = opaque assemblage.

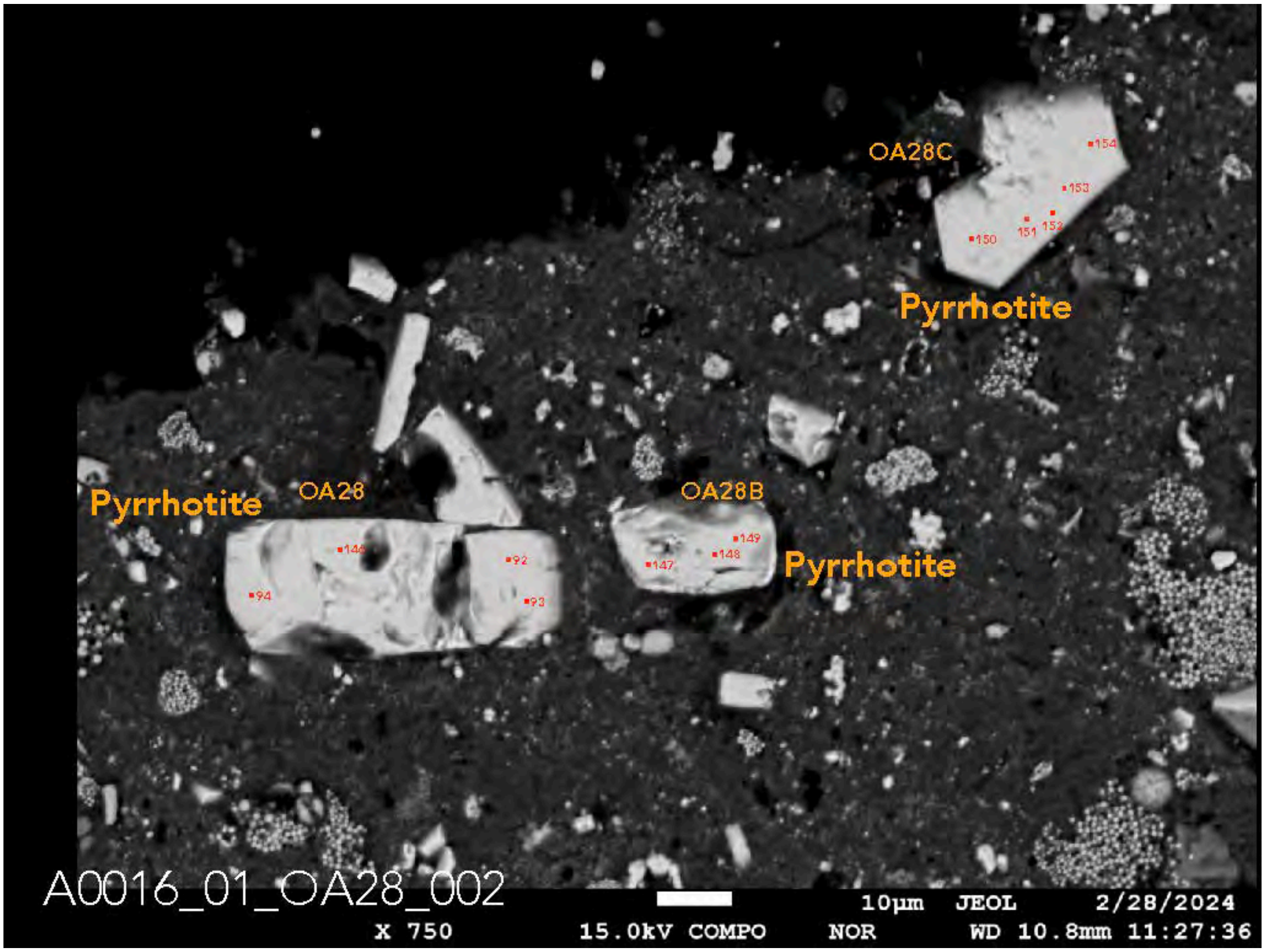

Supplementary Figure 82. Backscattered electron (BSE) image of OA28, OA28B, and OA28C in A0016, with spot analyses marked. OA = opaque assemblage.

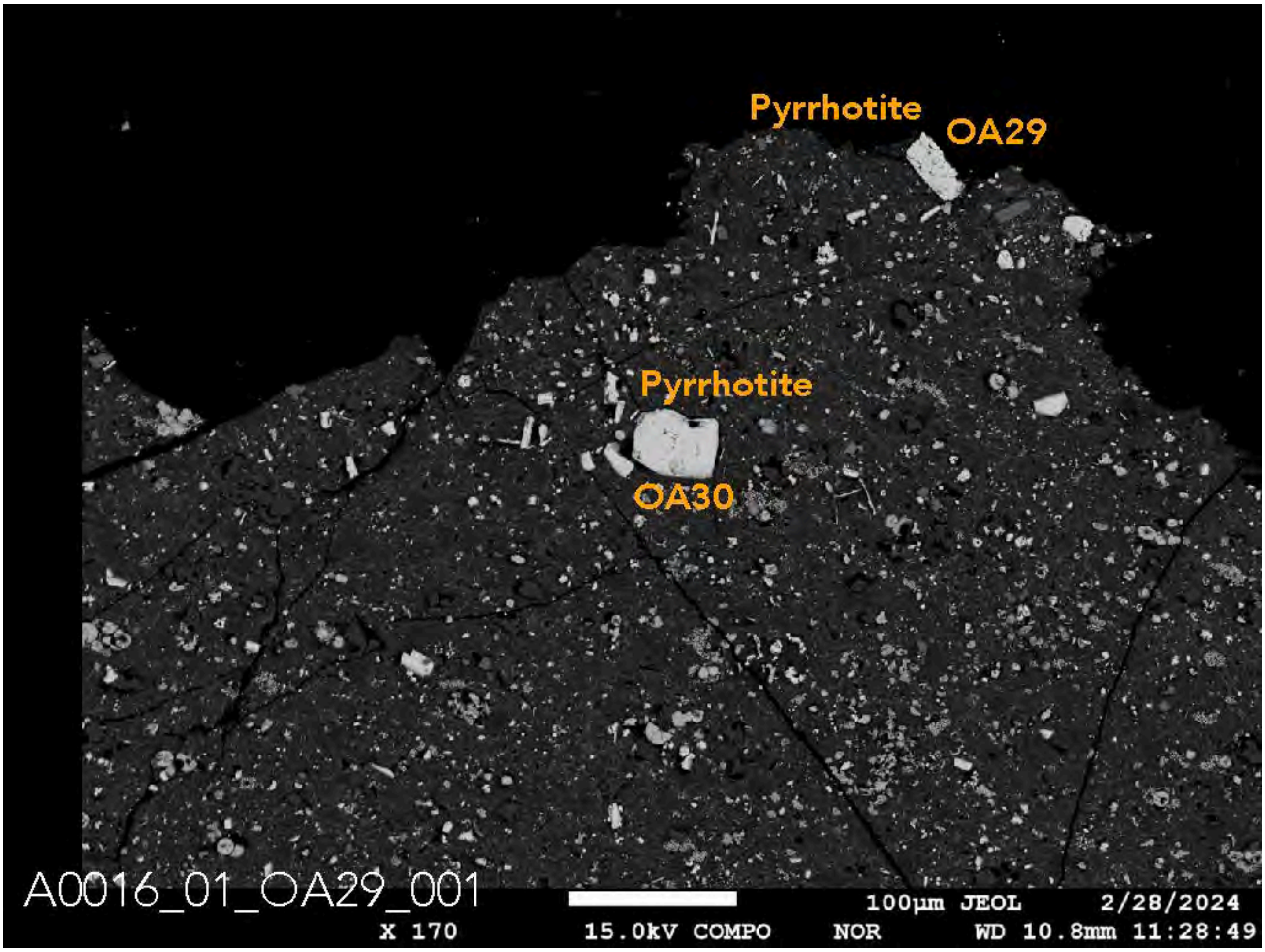

Supplementary Figure 83. Backscattered electron (BSE) image of OA29 and OA30. OA = opaque assemblage.

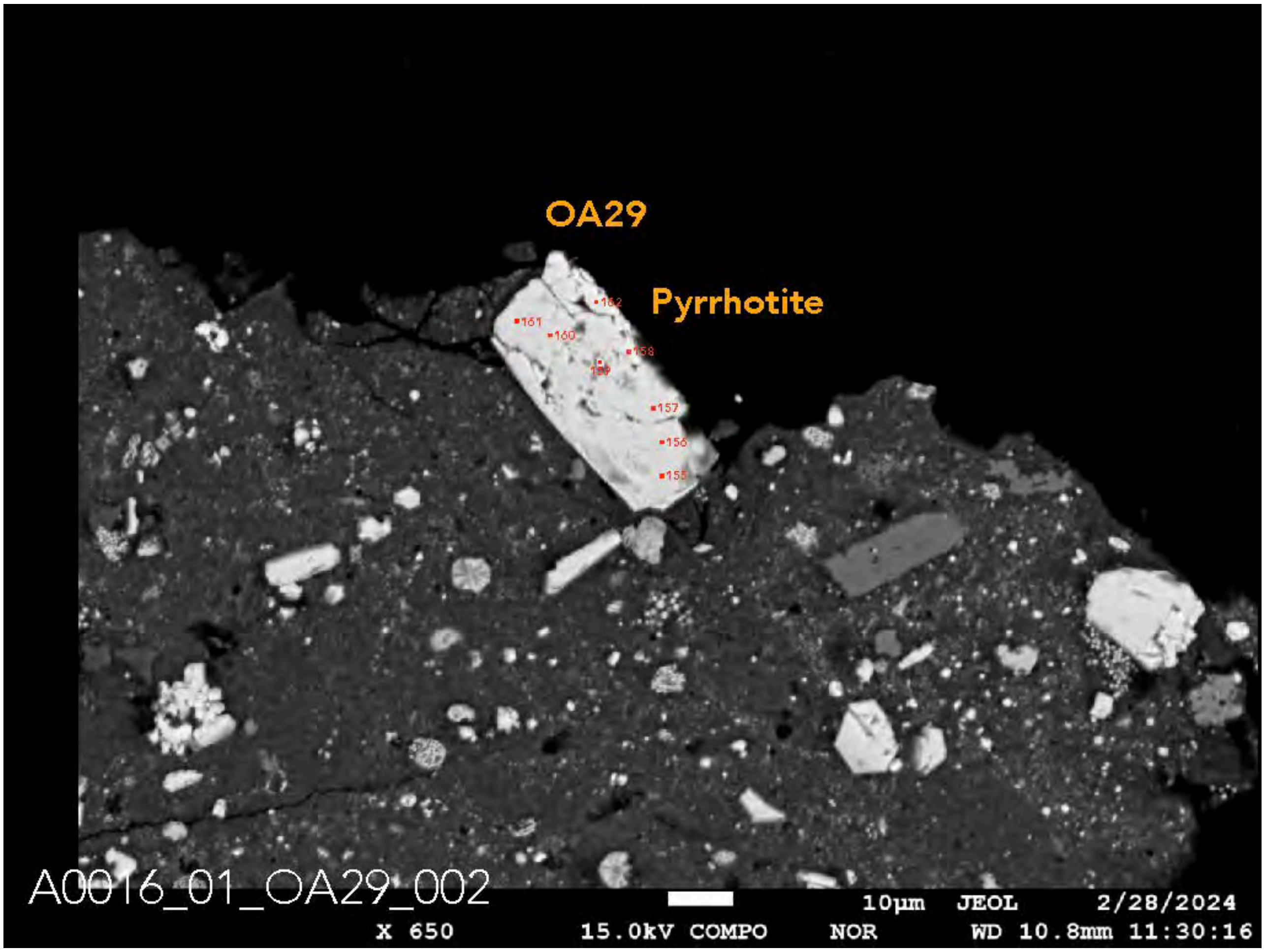

Supplementary Figure 84. Backscattered electron (BSE) image of OA29 in A0016, with spot analyses marked. OA = opaque assemblage.

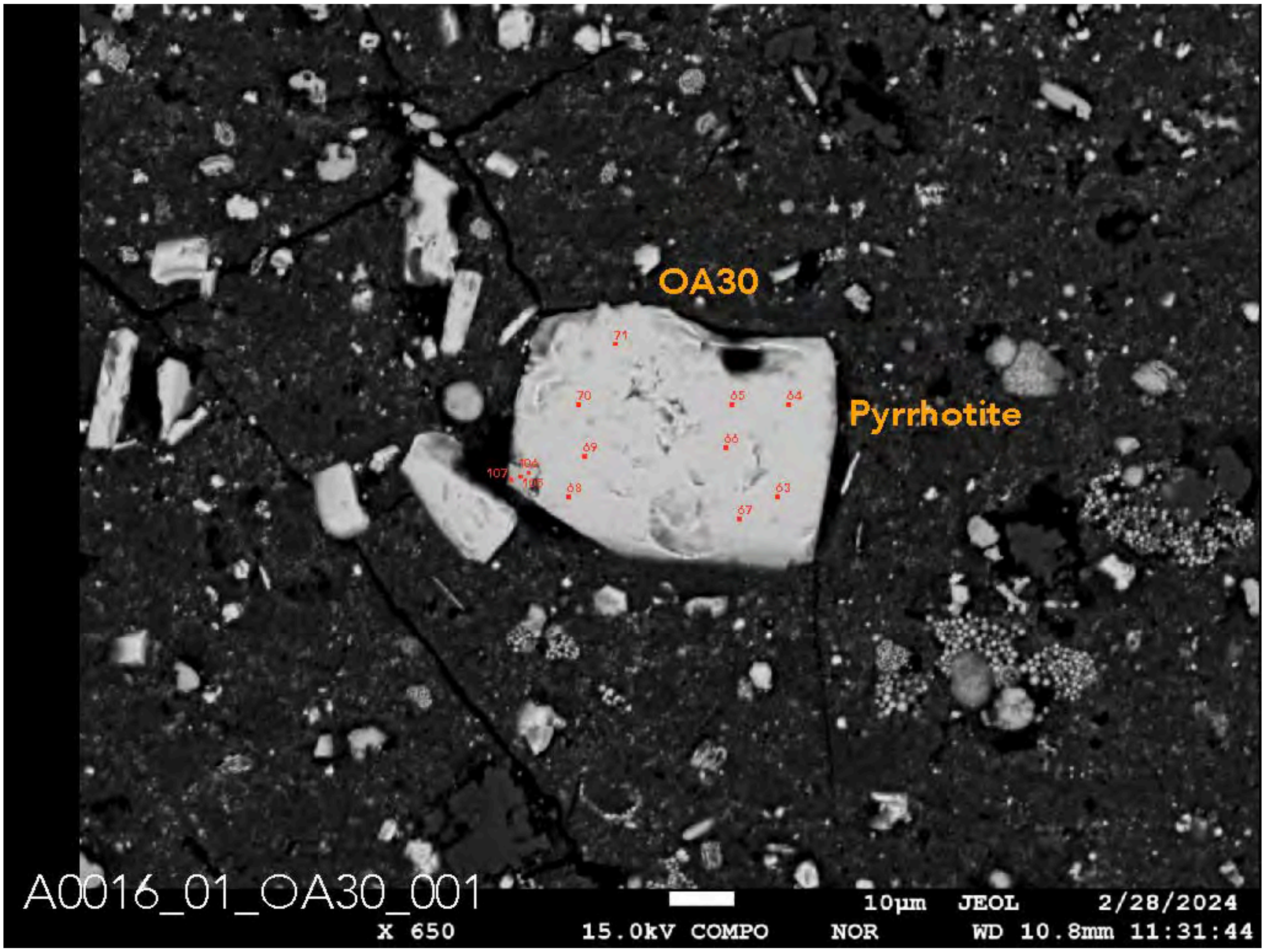

Supplementary Figure 85. Backscattered electron (BSE) image of OA30 in A0016, with spot analyses marked. OA = opaque assemblage.

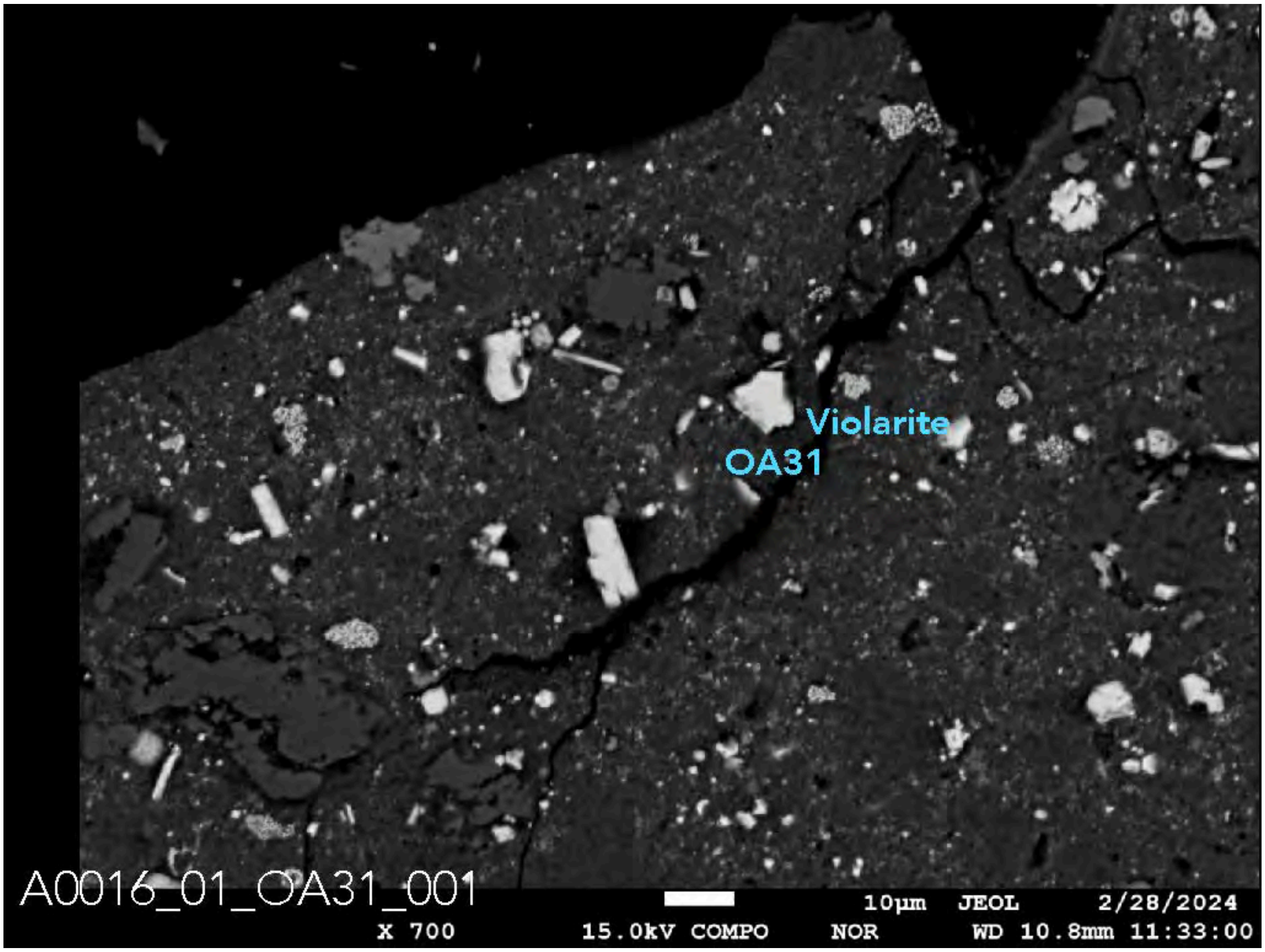

Supplementary Figure 86. Backscattered electron (BSE) image of OA31 in A0016. OA = opaque assemblage.

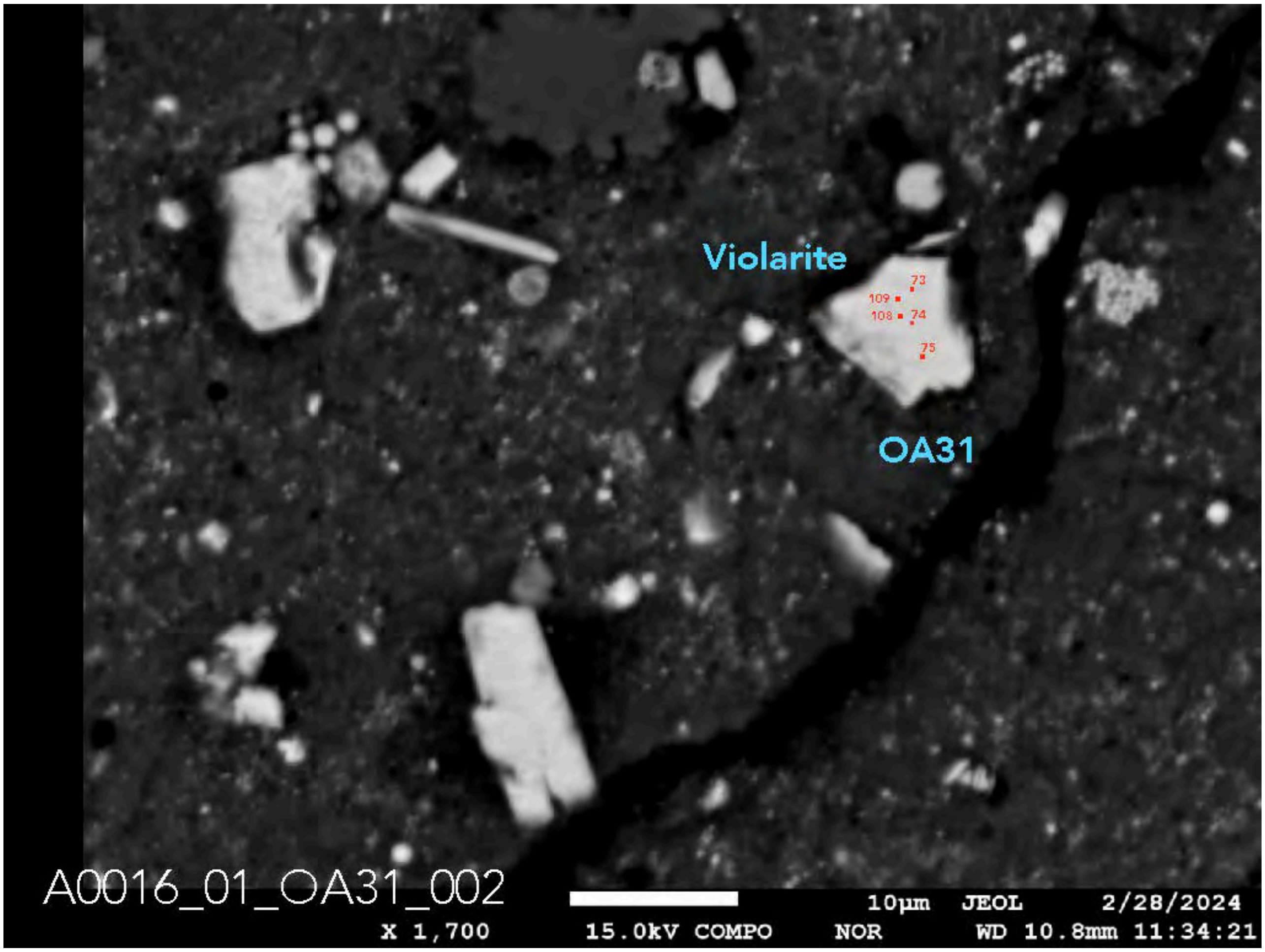

Supplementary Figure 87. Backscattered electron (BSE) image of OA31 in A0016, with spot analyses marked. OA = opaque assemblage.

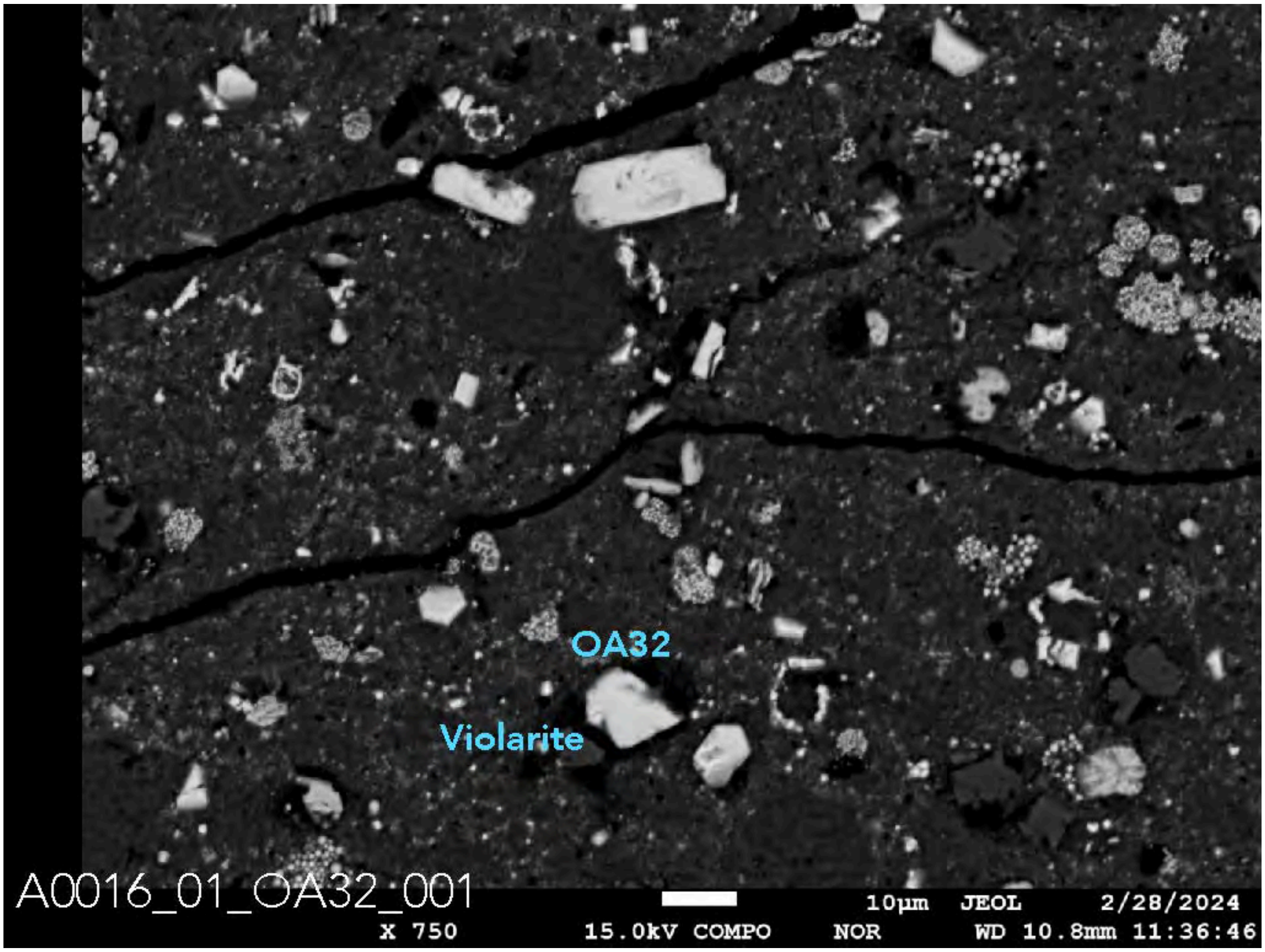

Supplementary Figure 88. Backscattered electron (BSE) image of OA32 in A0016. OA = opaque assemblage.

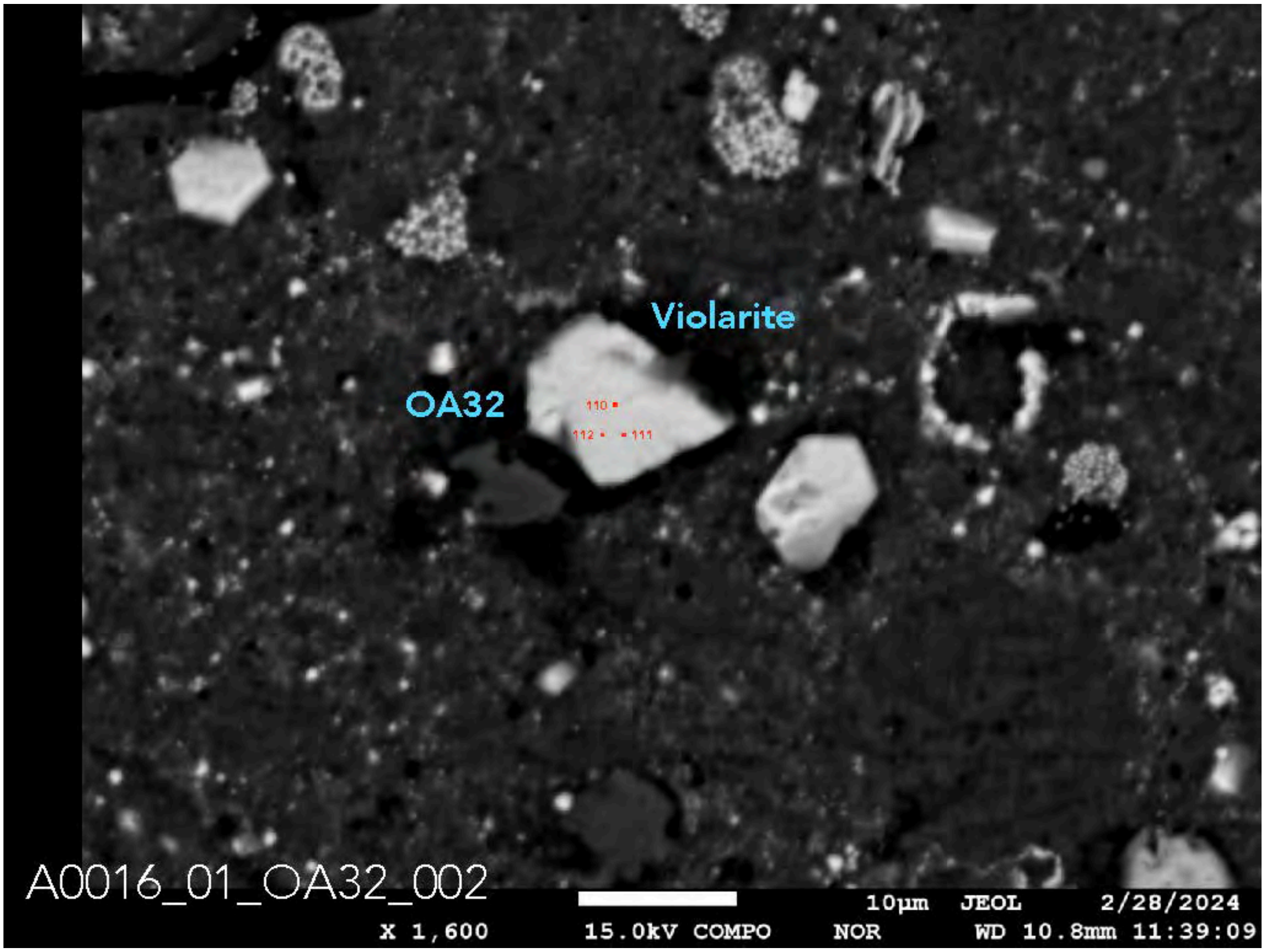

Supplementary Figure 89. Backscattered electron (BSE) image of OA32 in A0016, with spot analyses marked. OA = opaque assemblage.

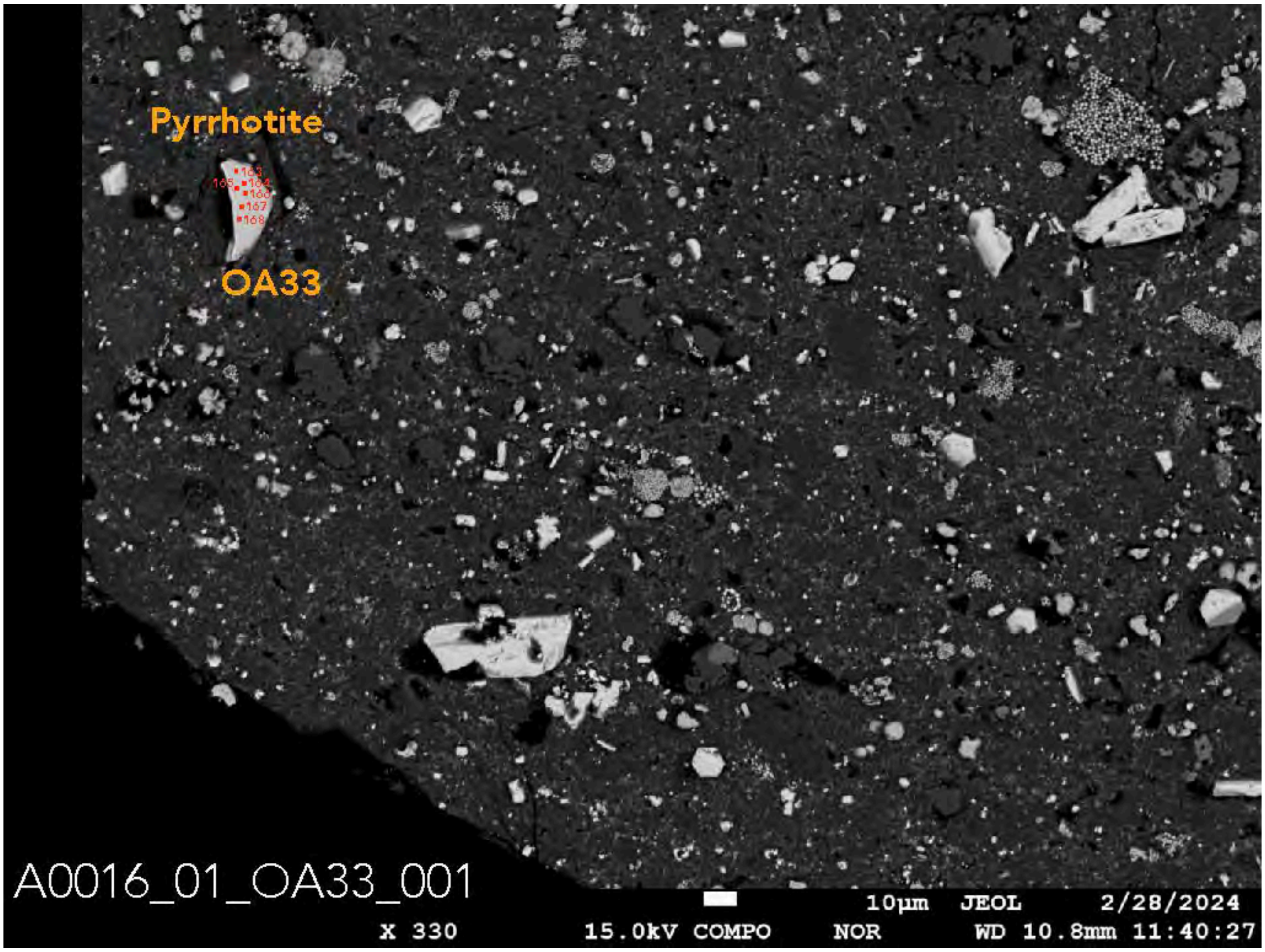

Supplementary Figure 90. Backscattered electron (BSE) image of OA33 in A0016, with spot analyses marked. OA = opaque assemblage.

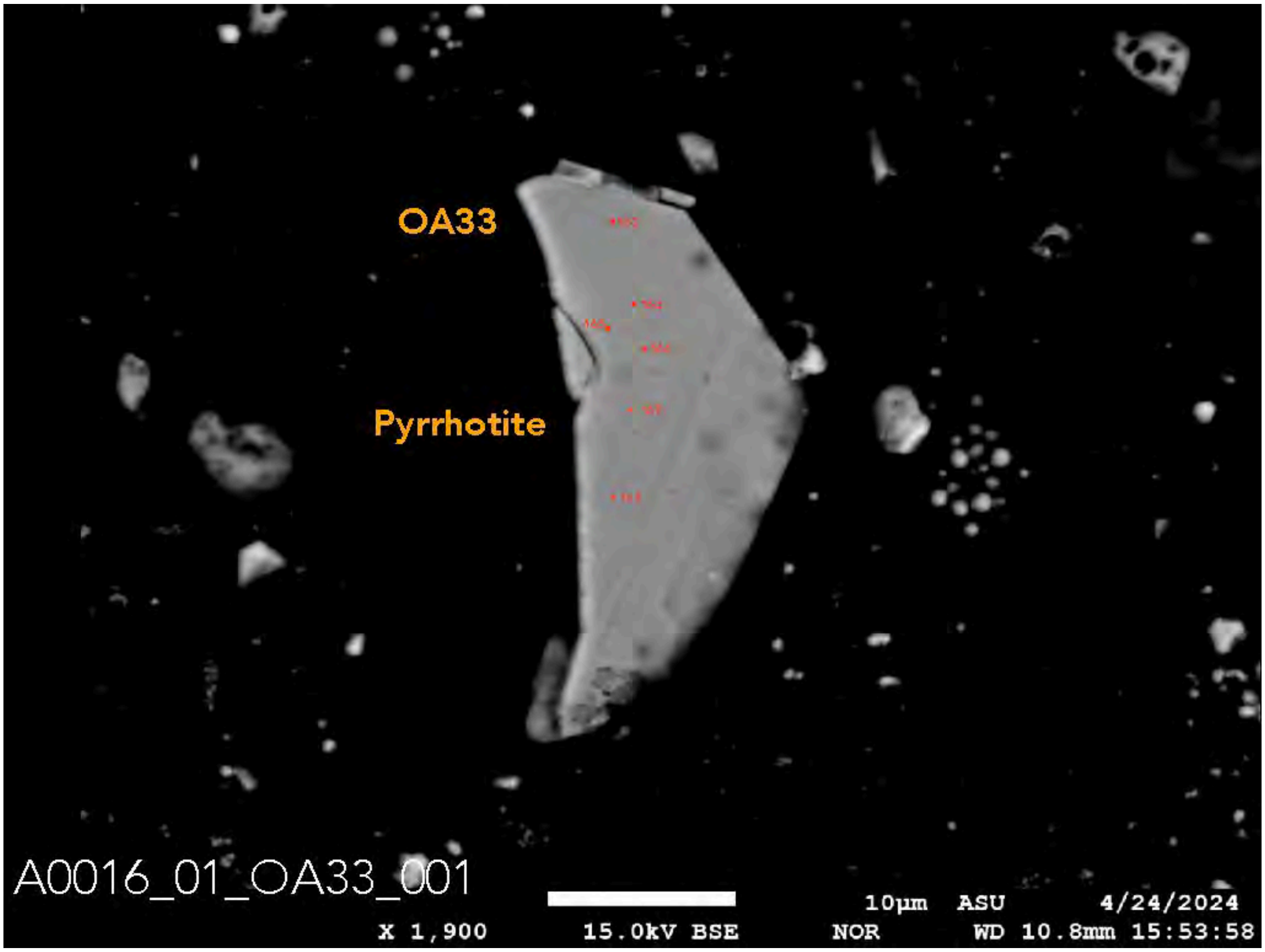

Supplementary Figure 91. Backscattered electron (BSE) image of OA33 in A0016, with spot analyses marked. OA = opaque assemblage.

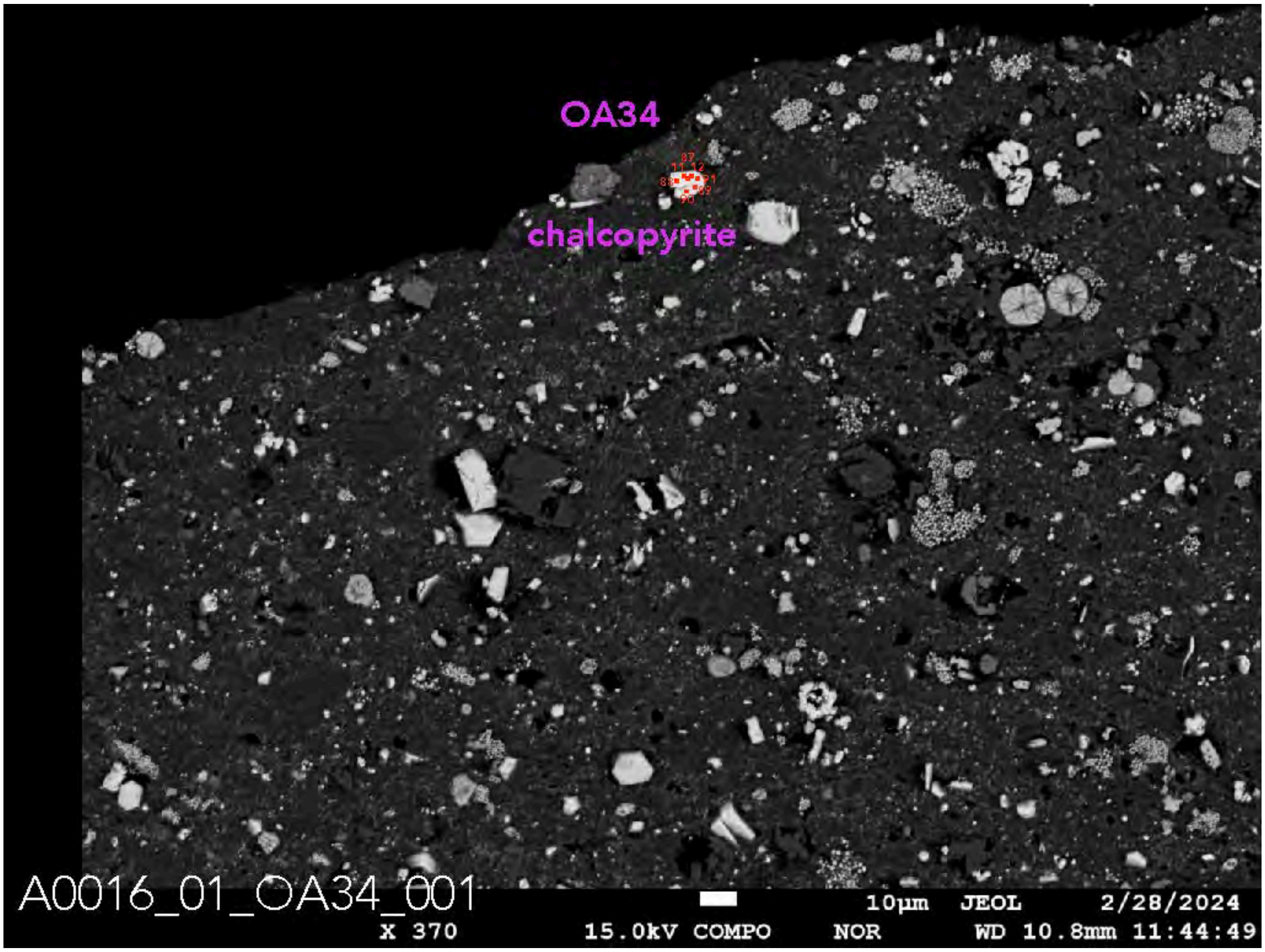

Supplementary Figure 92. Backscattered electron (BSE) image of OA34 in A0016, with spot analyses marked. OA = opaque assemblage.

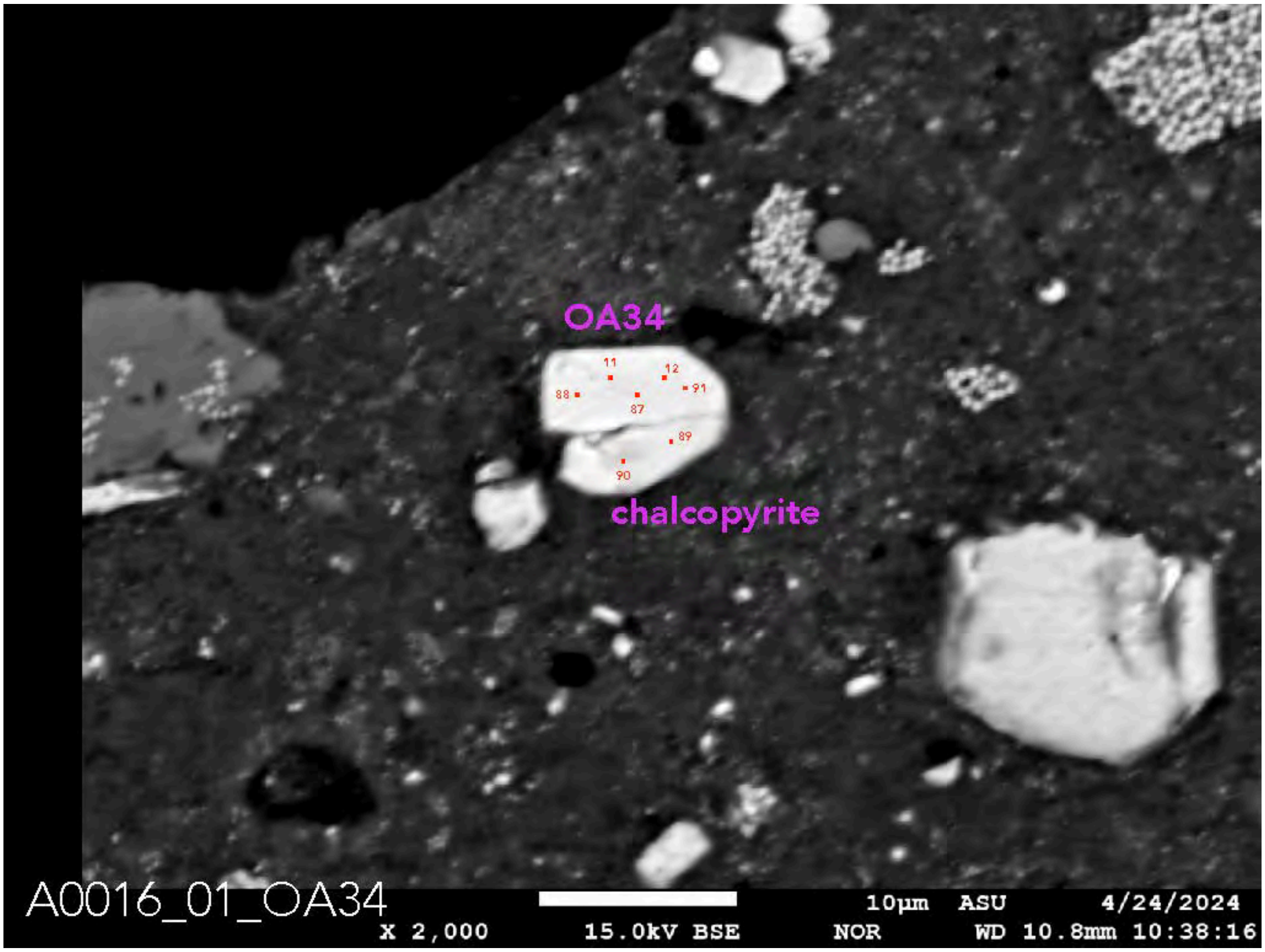

Supplementary Figure 93. Backscattered electron (BSE) image of OA34 in A0016, with spot analyses marked. OA = opaque assemblage.

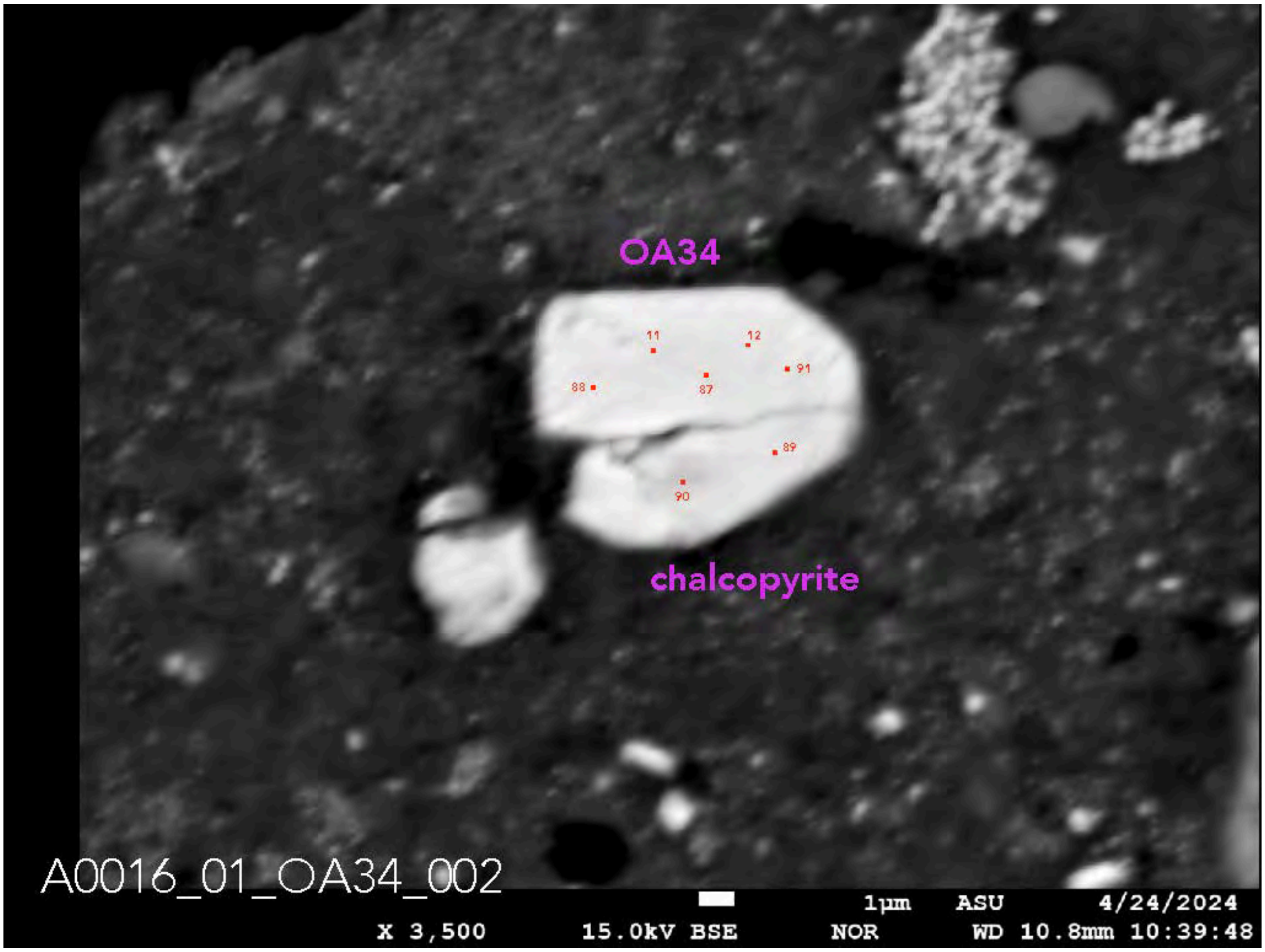

Supplementary Figure 94. Backscattered electron (BSE) image of OA34 in A0016, with spot analyses marked. OA = opaque assemblage.

Part II

Images and data point locations for Ryugu Samples: A0094-01 and C0103-01.

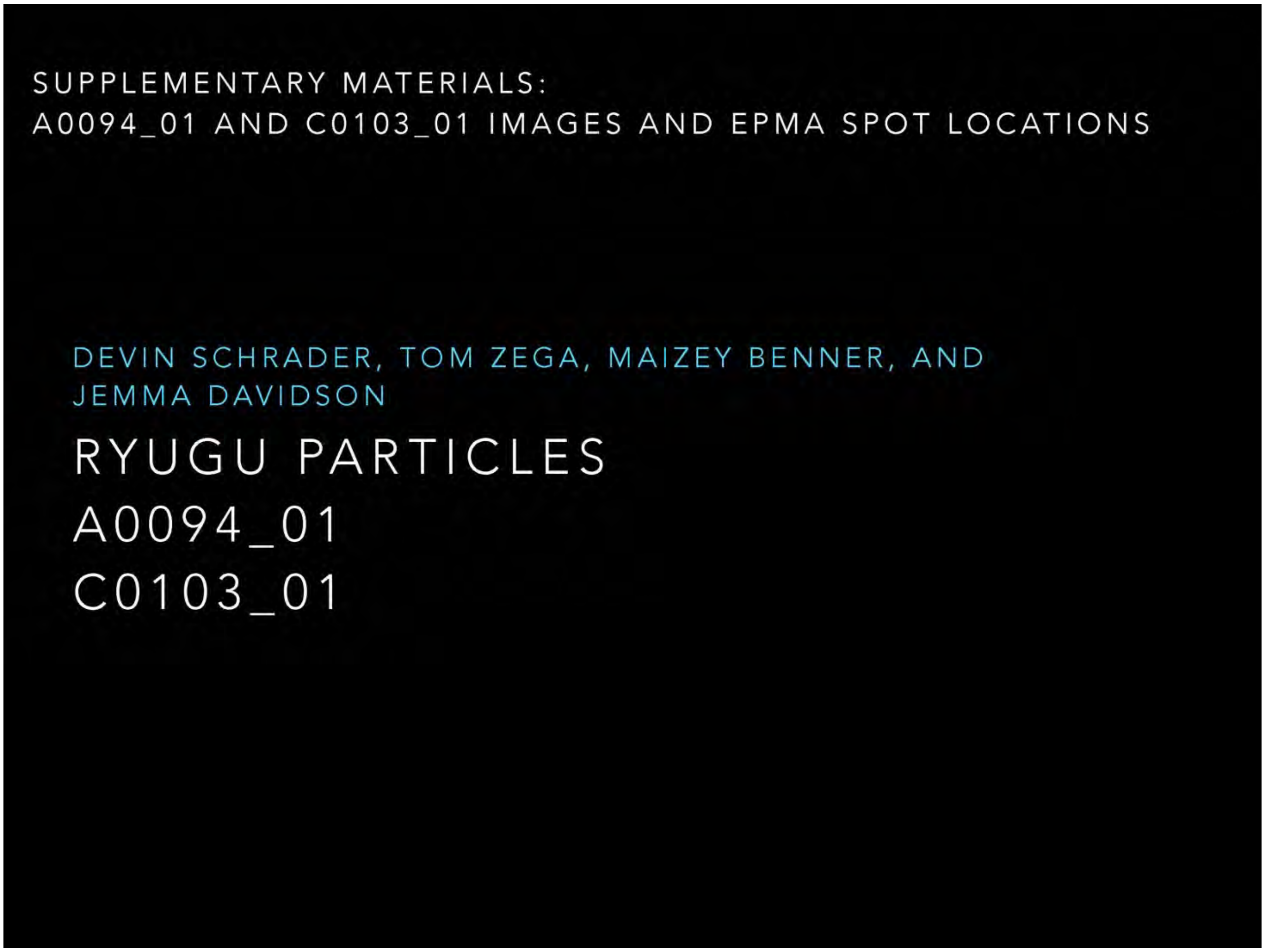

Supplementary Figure 95. Title slide for A0094-01 and C0103-01 images and electron probe microanalyzer (EPMA) spot locations.

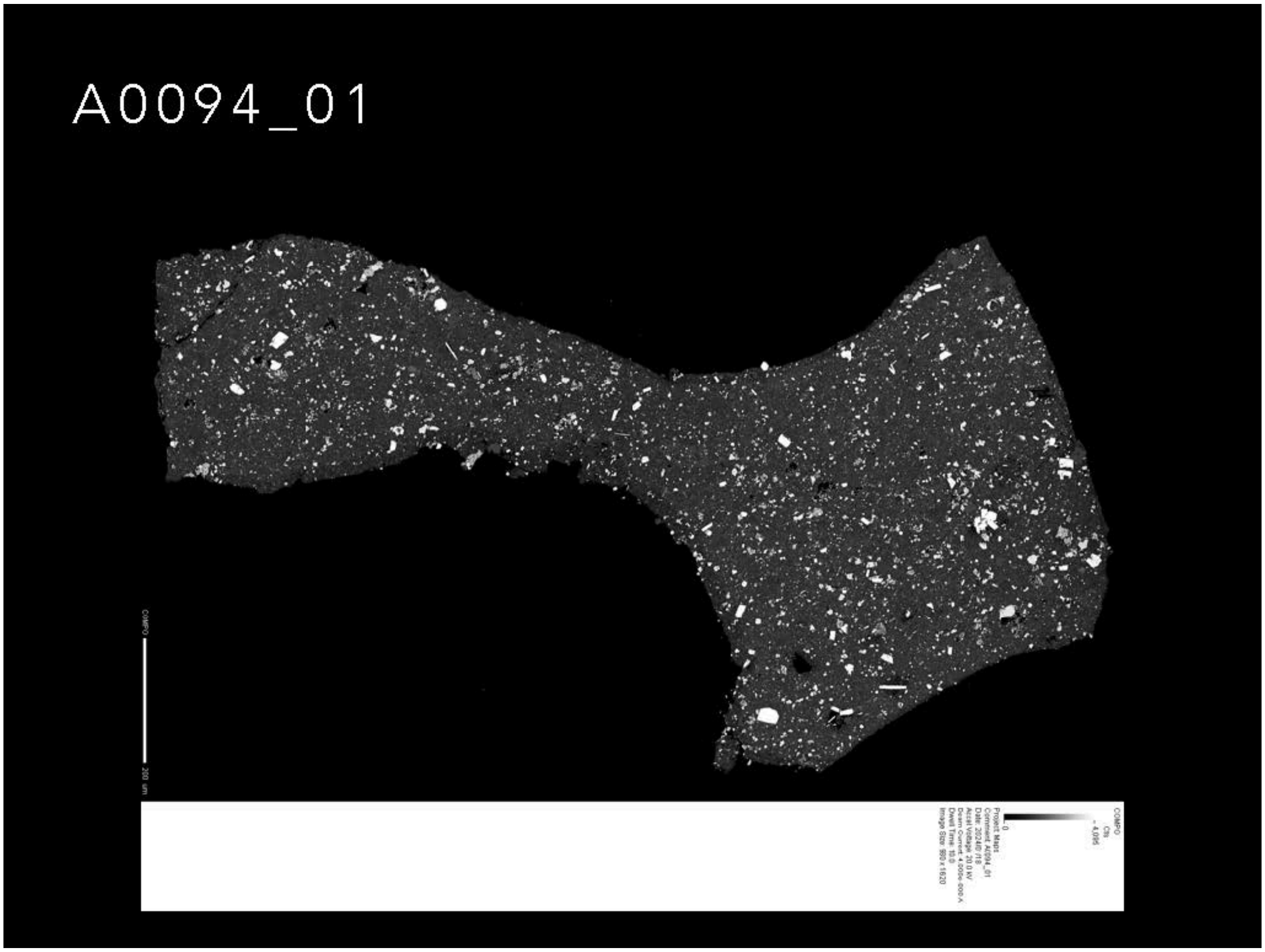

Supplementary Figure 96. Full backscattered electron (BSE) image of A0094-01.

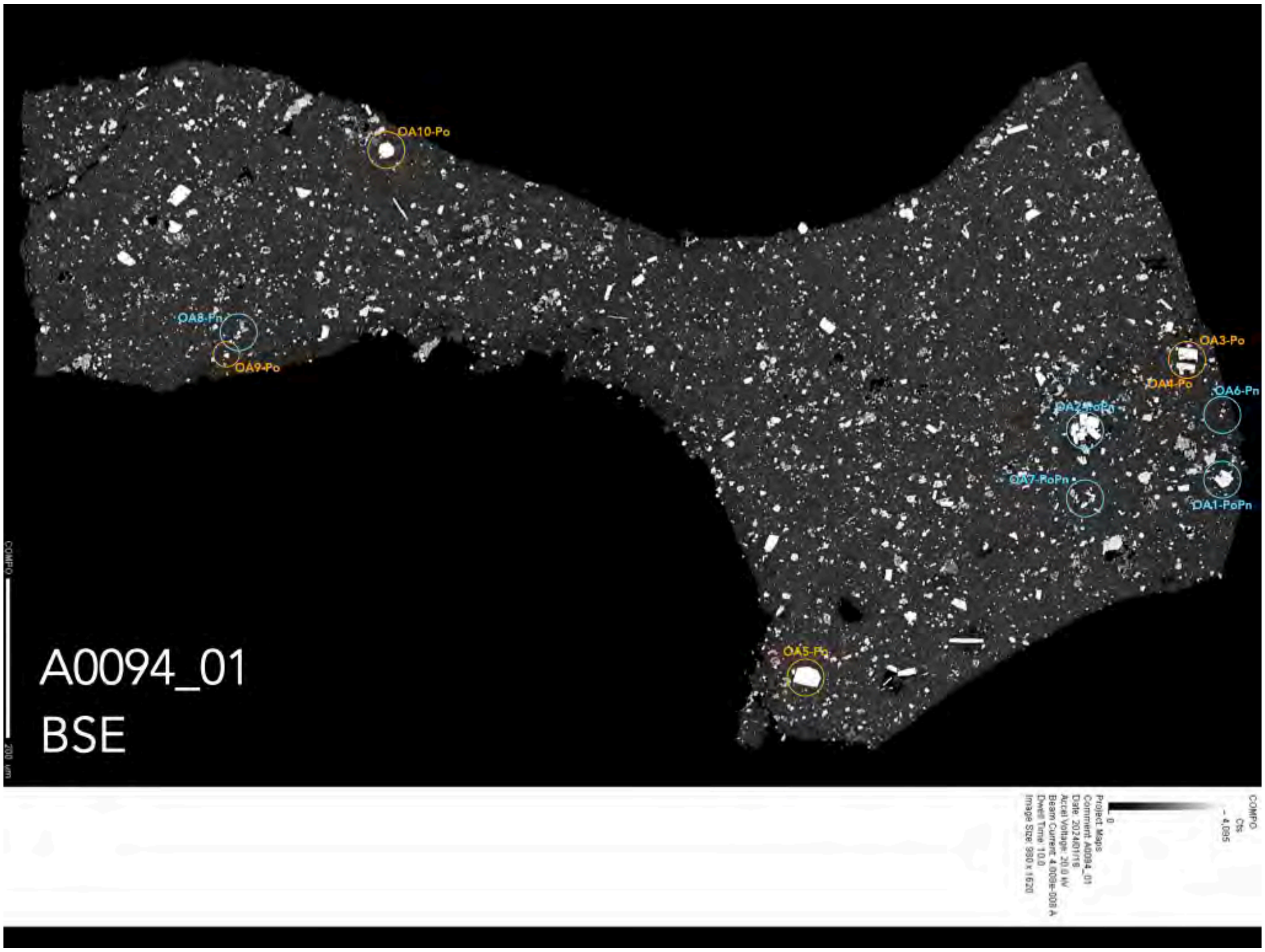

Supplementary Figure 97. Full backscattered electron (BSE) image of A0094-01 with location of all sulfides imaged in detail and analyzed marked. Where OA = opaque assemblage, Po = pyrrhotite, and Pn = pentlandite.

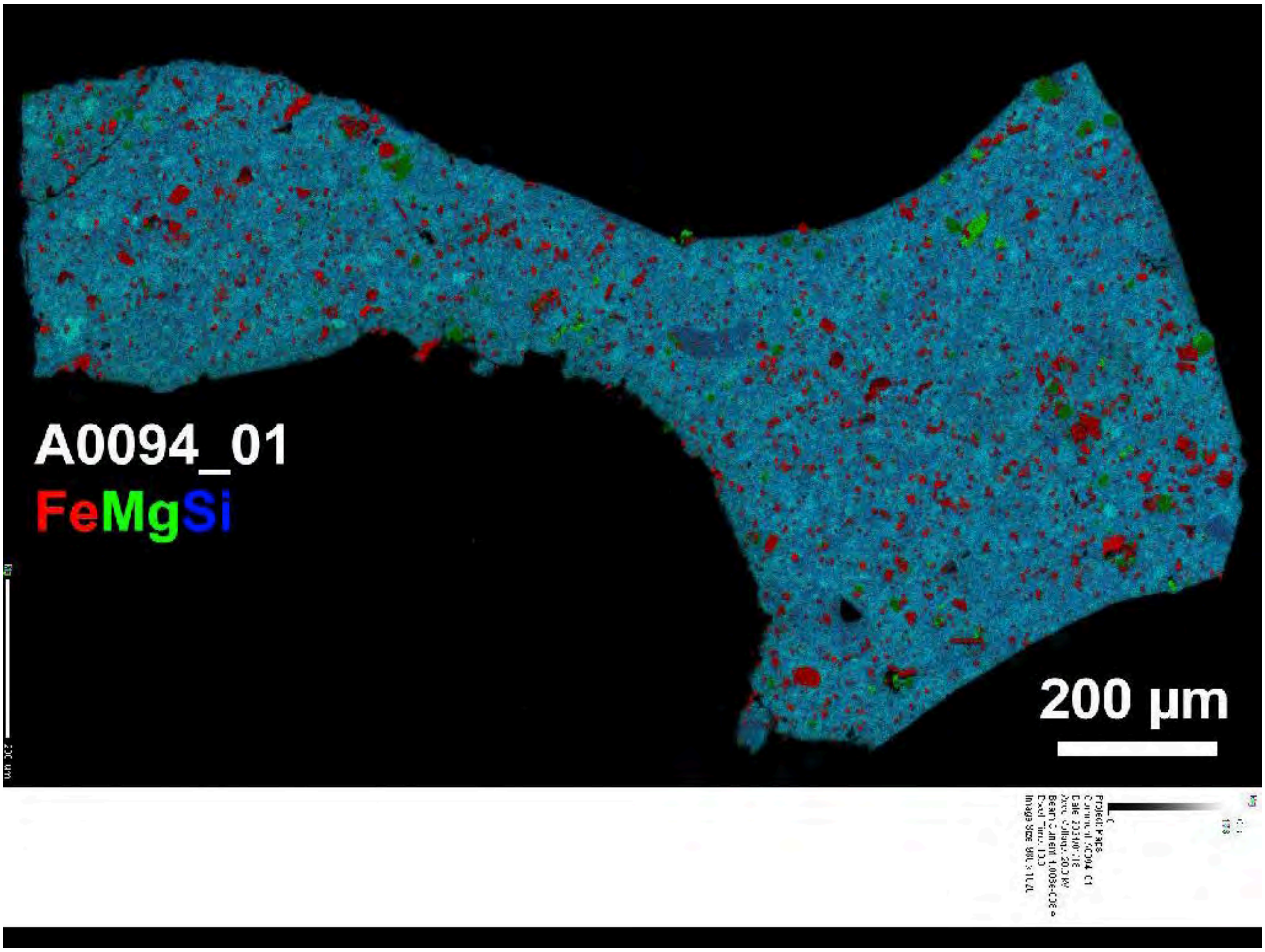

Supplementary Figure 98. Full composite X-ray element RGB image (Fe = red, Mg = green, and Si = blue) of A0094-01.

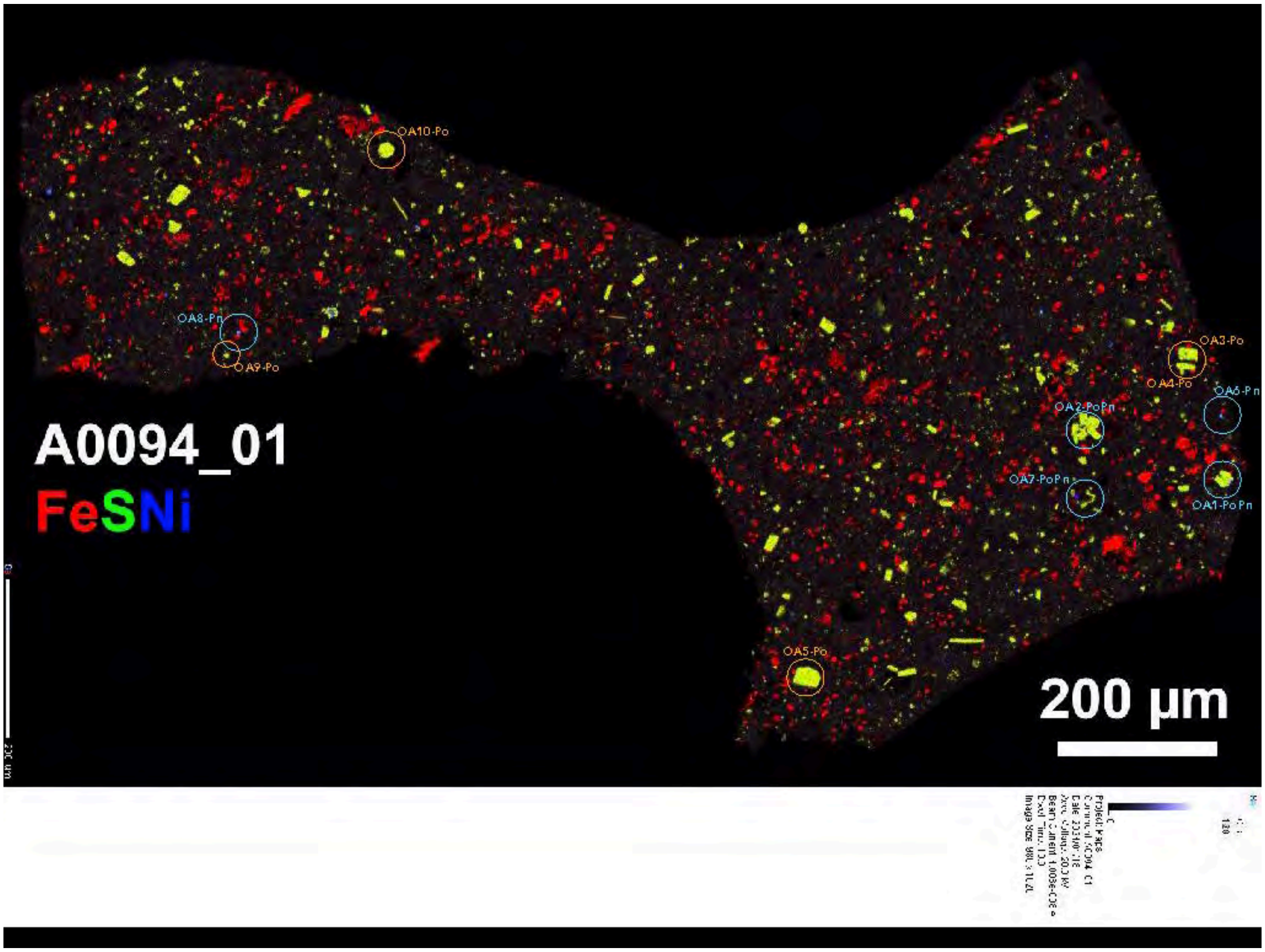

Supplementary Figure 99. Full composite X-ray element RGB image (Fe = red, S = green, and Ni = blue) of A0094-01, with location of all sulfides imaged in detail and analyzed marked. Where OA = opaque assemblage, Po = pyrrhotite, and Pn = pentlandite.

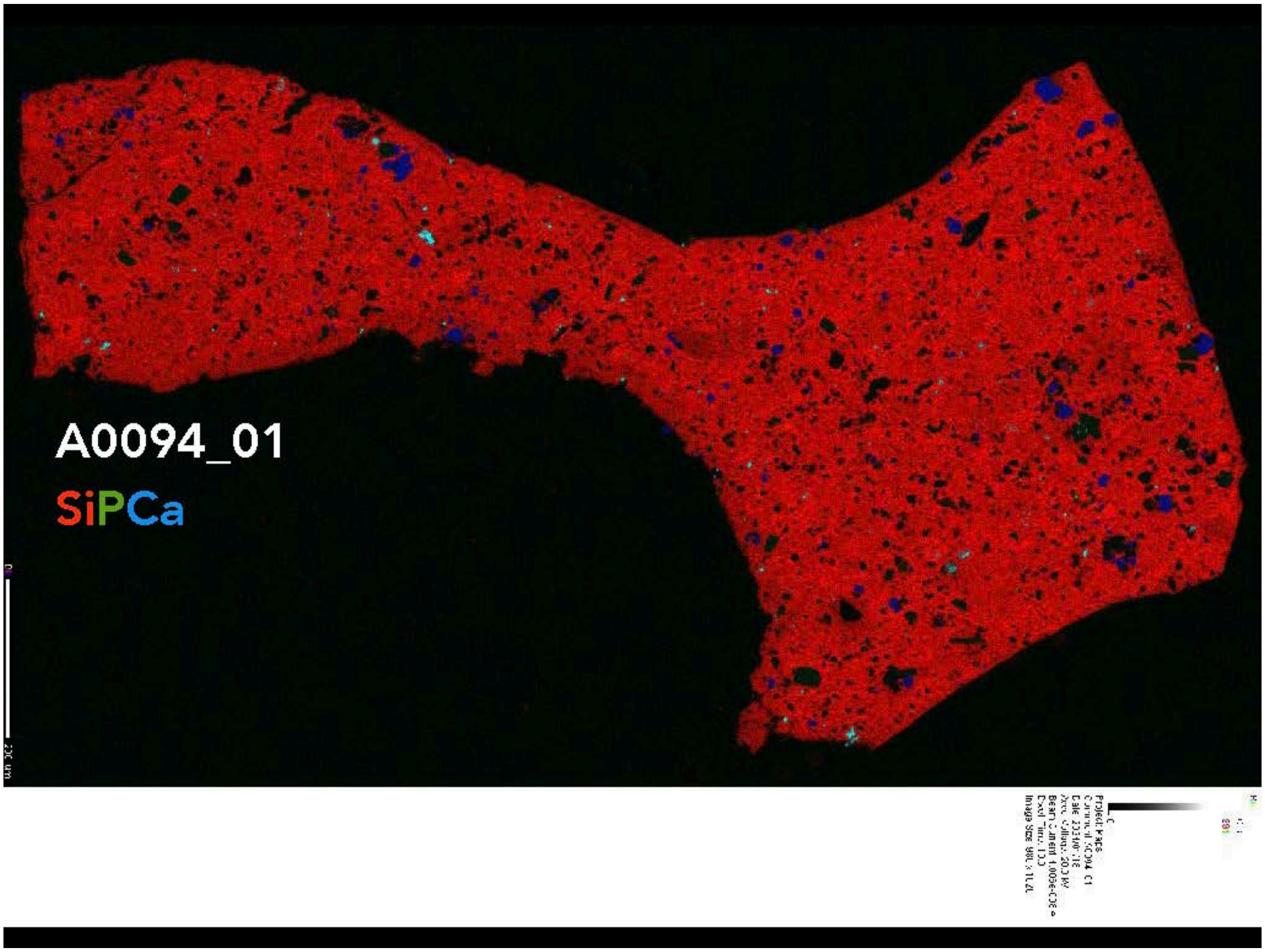

Supplementary Figure 100. Full composite X-ray element RGB image (Si = red, P = green, and Ca = blue) of A0094-01.

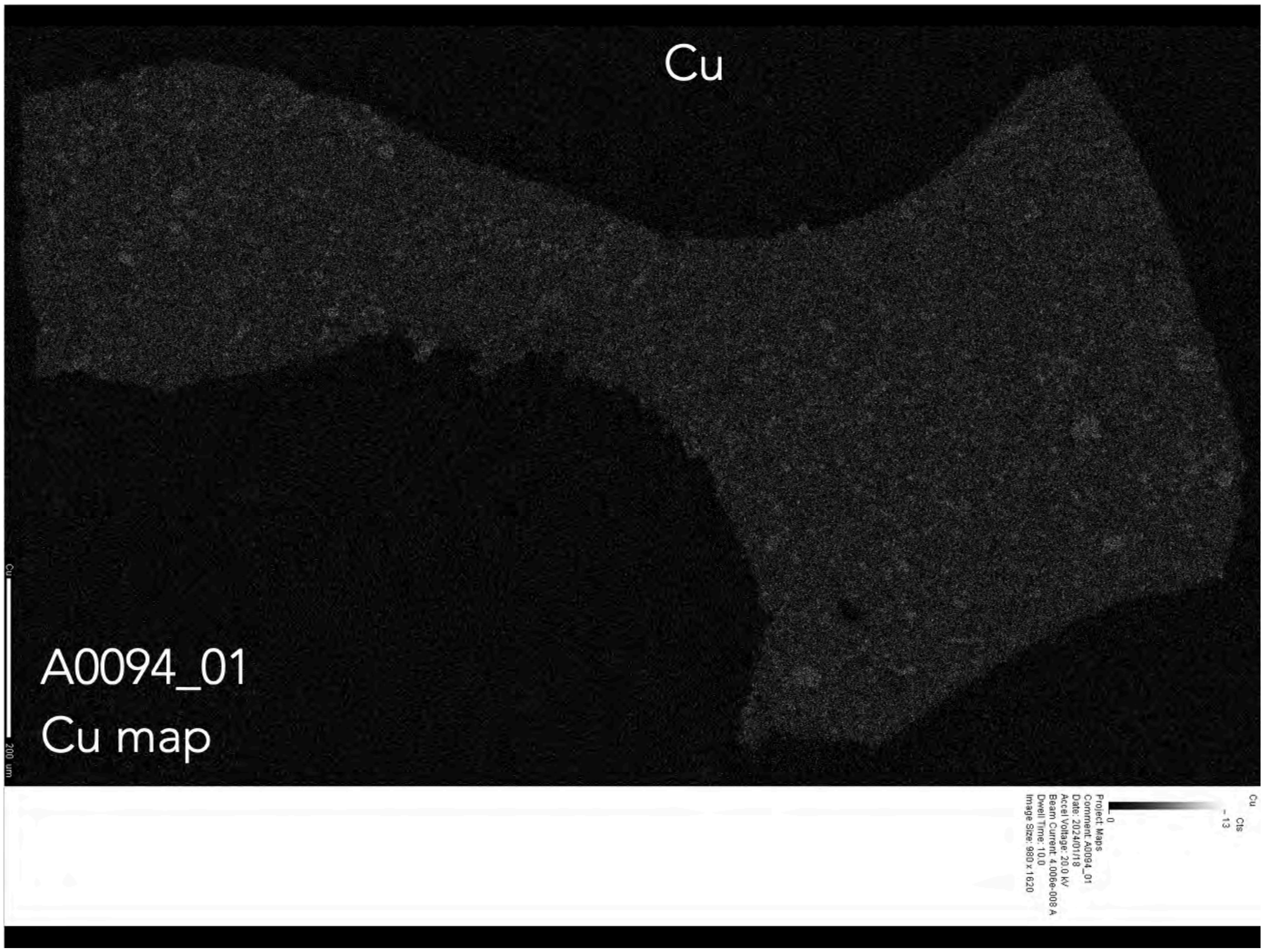

Supplementary Figure 101. Cu X-ray element map of A0094-01, showing no Cu-sulfides present.

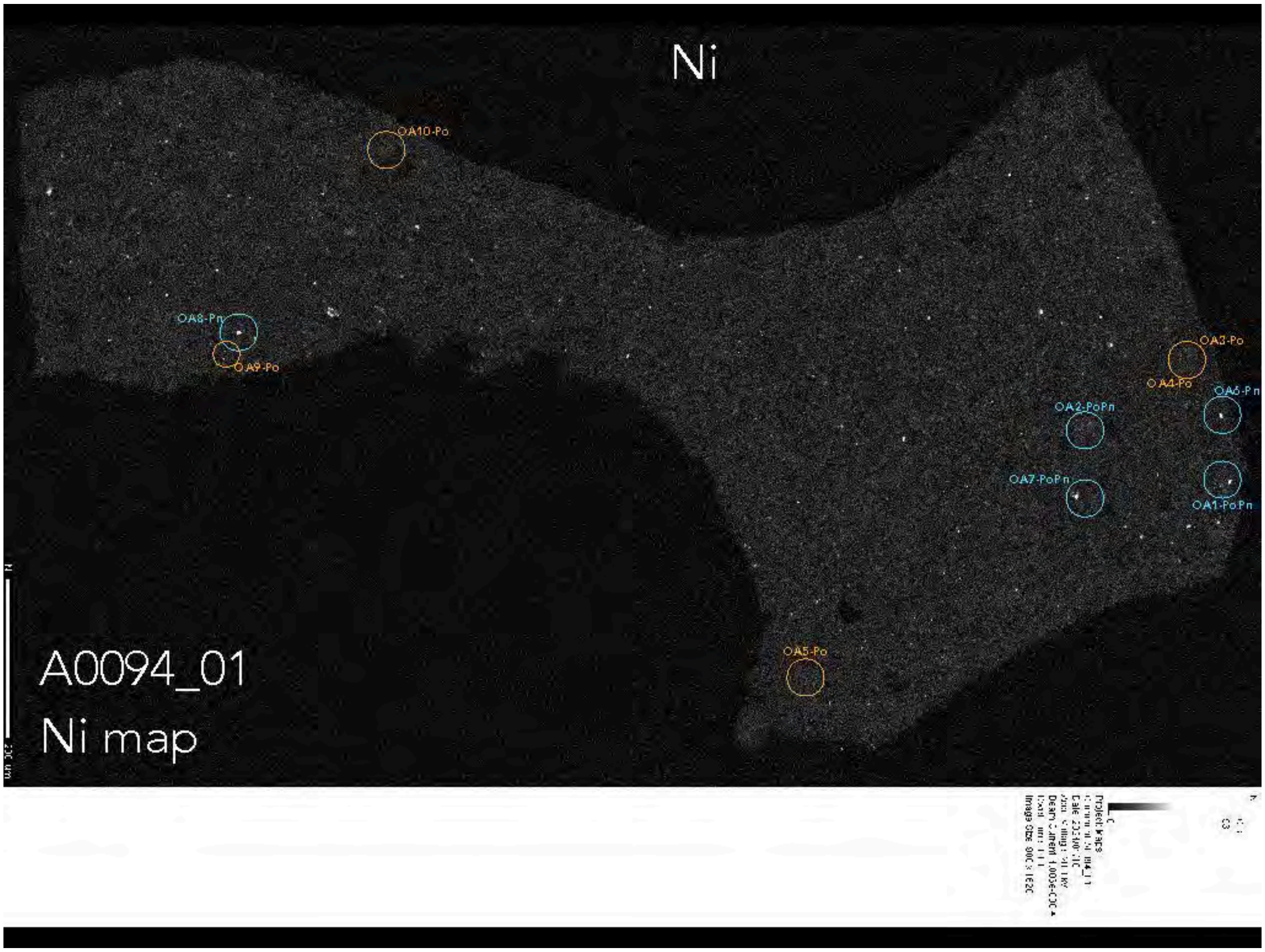

Supplementary Figure 102. Ni X-ray element map of A0094-01, with Ni-bearing sulfides analyzed marked. Where OA = opaque assemblage, Po = pyrrhotite and Pn = pentlandite.

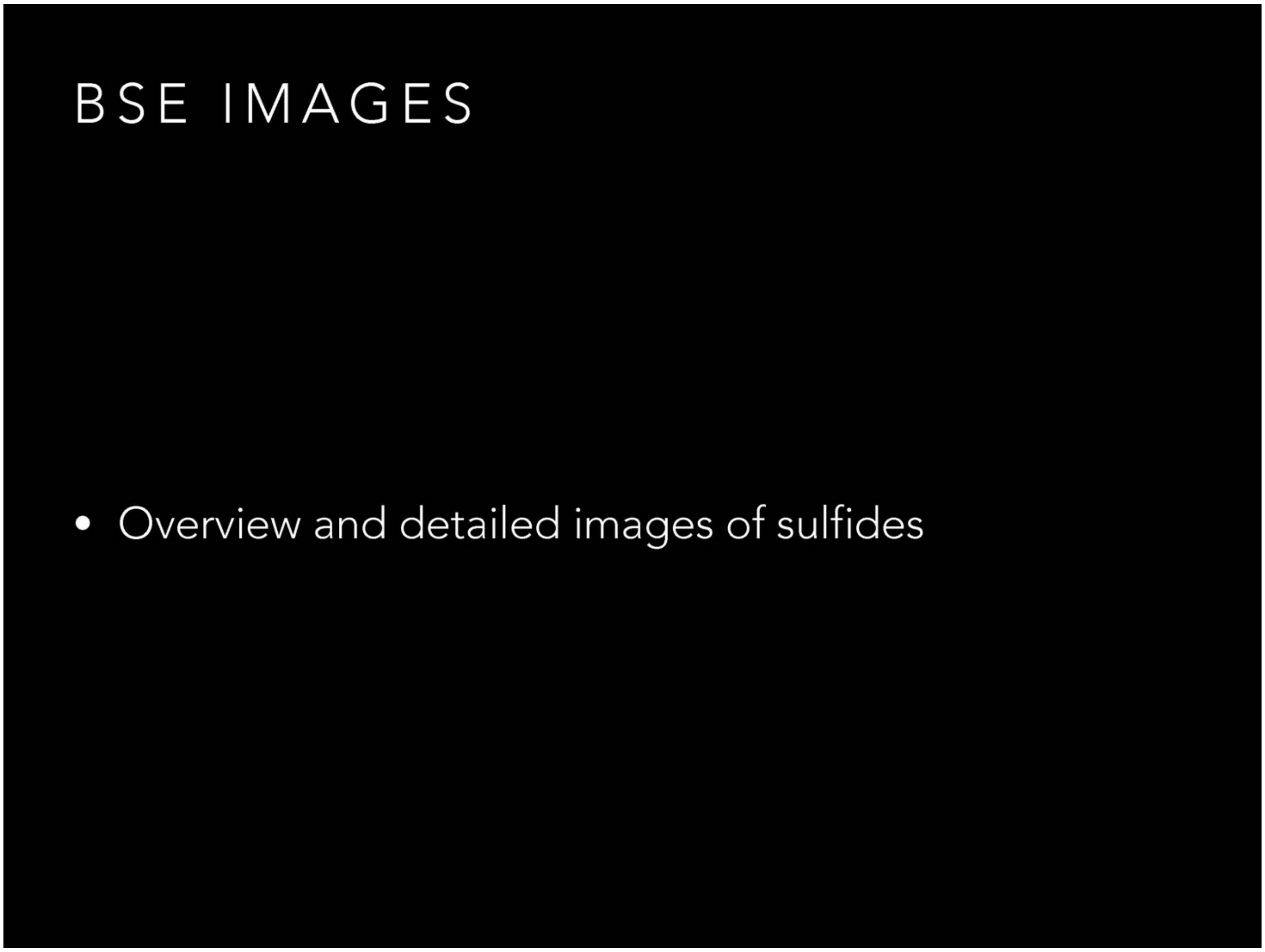

Supplementary Figure 103. Title slide for opaque assemblages in A0094-01.

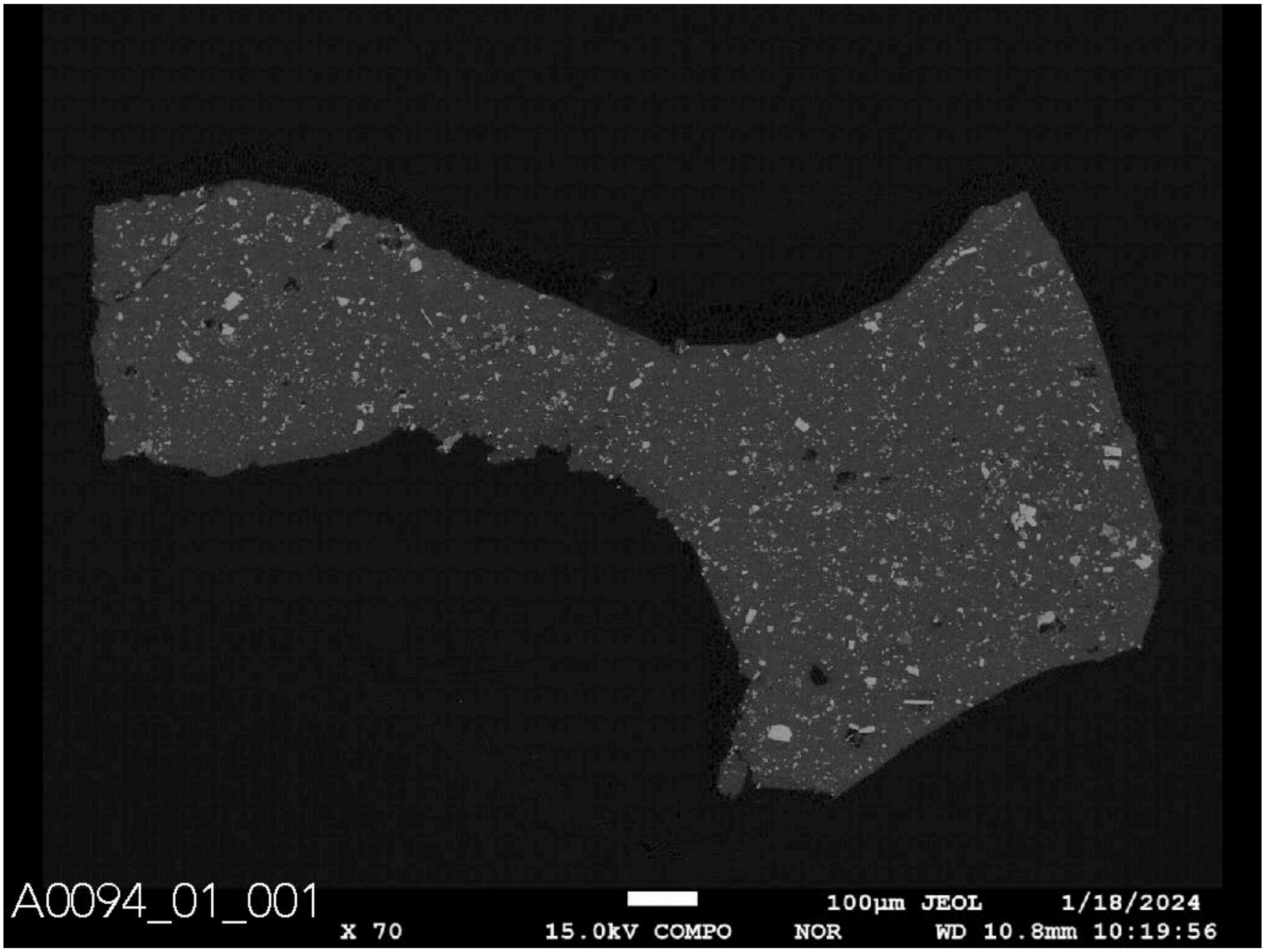

Supplementary Figure 104. Full backscattered electron (BSE) image of A0094-01.

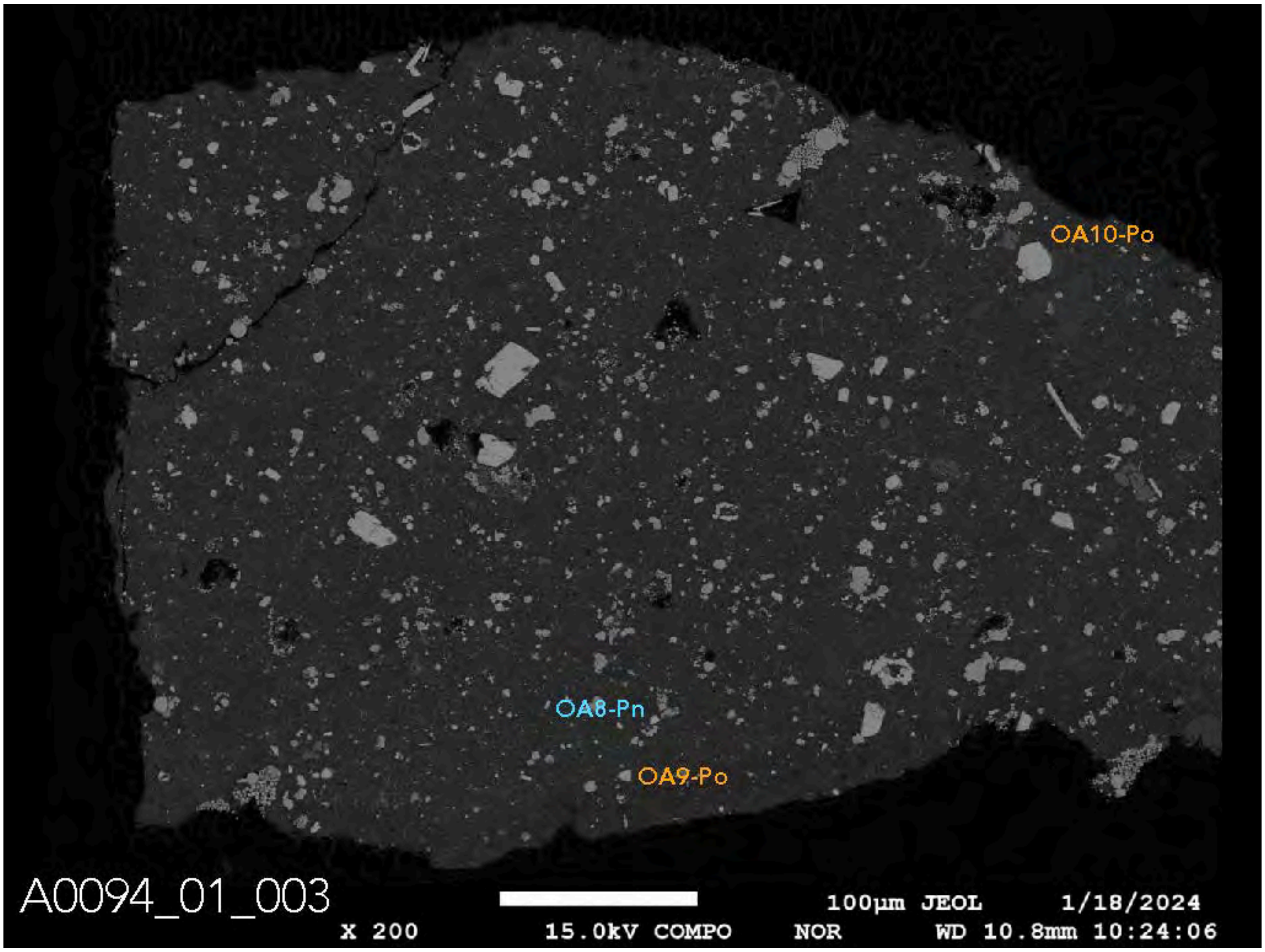

Supplementary Figure 105. Backscattered electron (BSE) image of region in A0094-01 showing OA8, OA9, and OA10. OA = opaque assemblage, Po = pyrrhotite, and Pn = pentlandite.

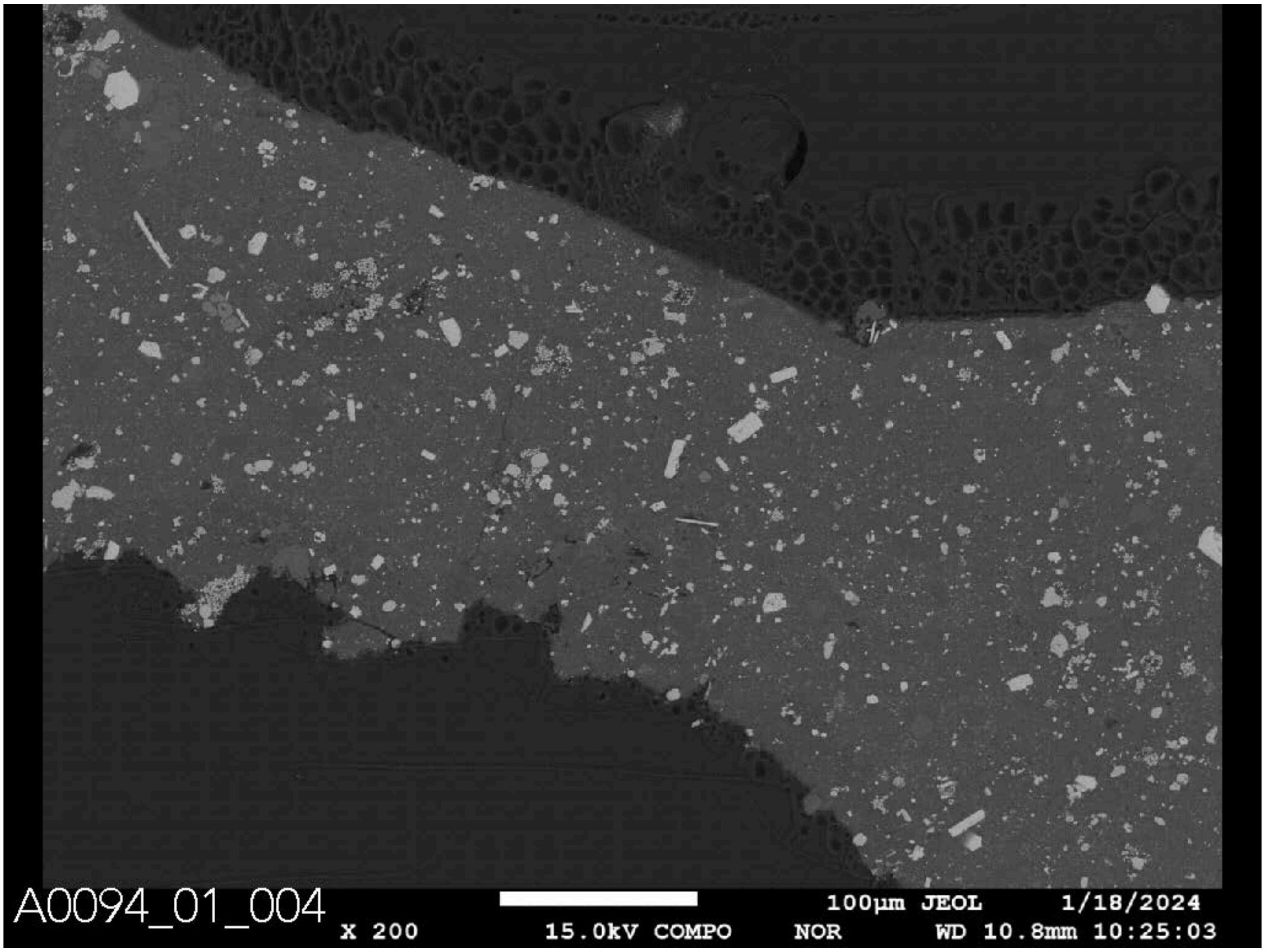

Supplementary Figure 106. Backscattered electron (BSE) image of region in A0094-01.

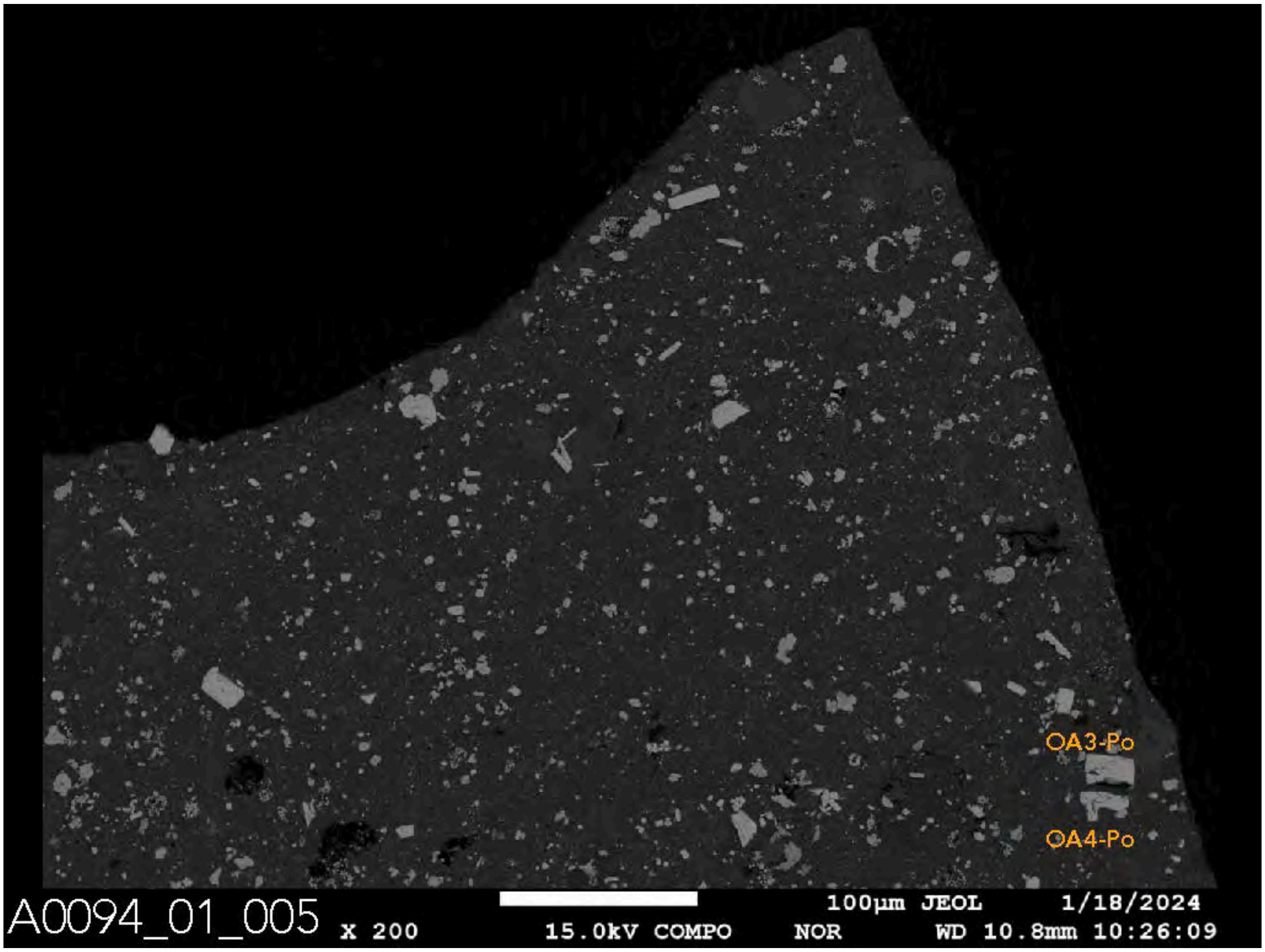

Supplementary Figure 107. Backscattered electron (BSE) image of region in A0094-01 showing OA3 and OA4. OA = opaque assemblage, and Po = pyrrhotite.

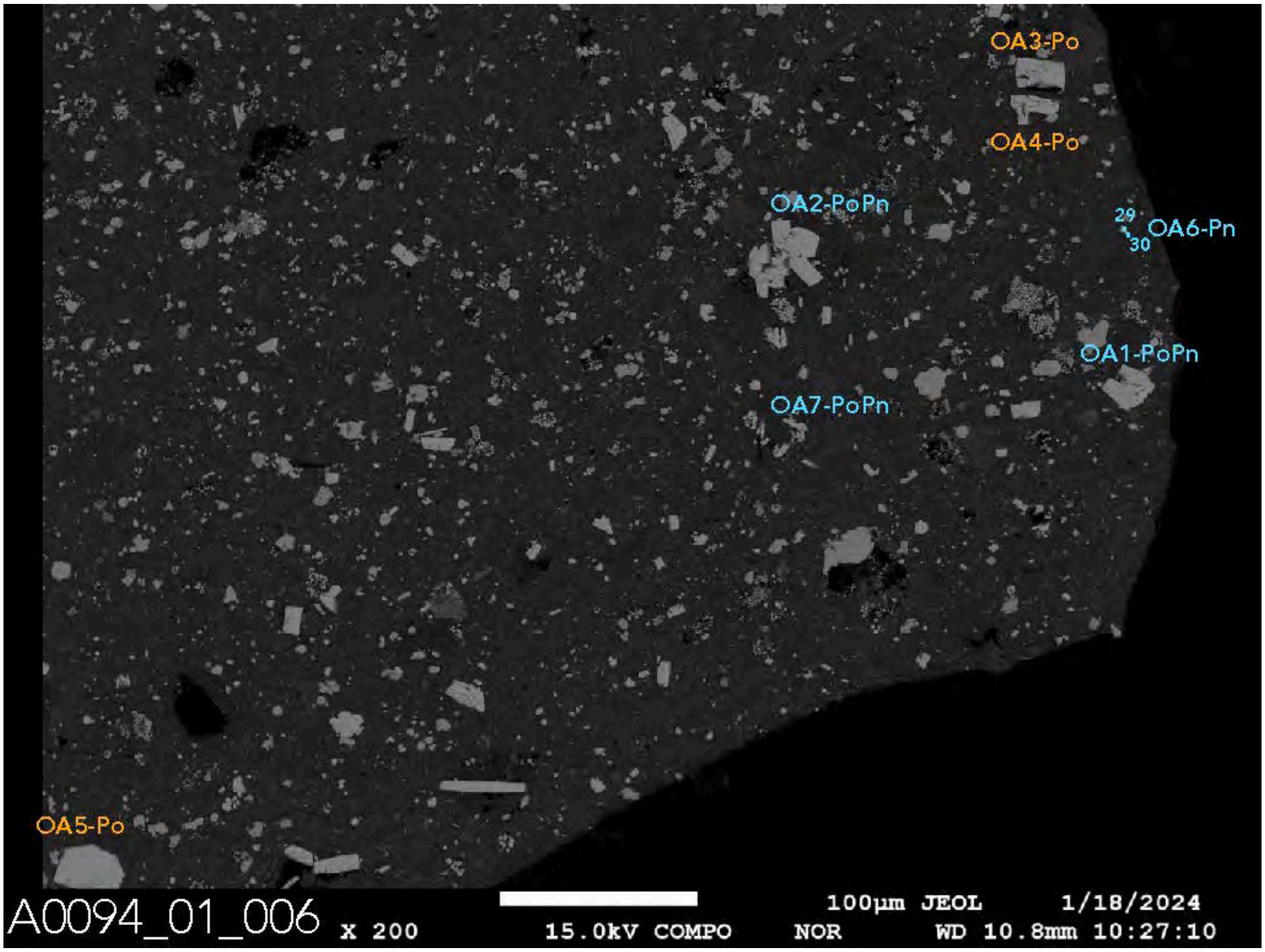

Supplementary Figure 108. Backscattered electron (BSE) image of region in A0094-01 showing OA1, OA2, OA3, OA4, OA6, and OA7. Analysis spots on OA6 shown. OA = opaque assemblage, Po = pyrrhotite, and Pn = pentlandite.

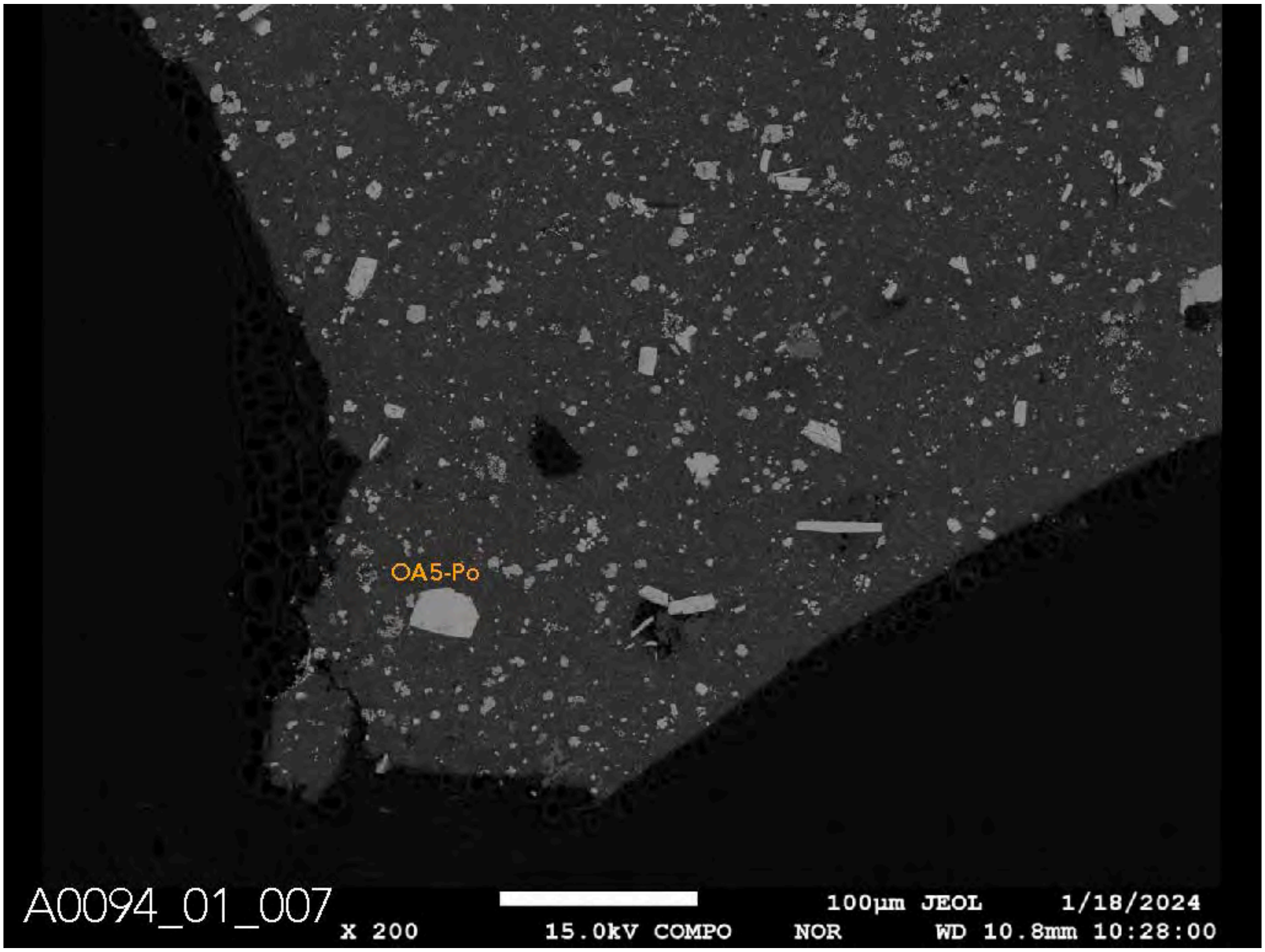

Supplementary Figure 109. Backscattered electron (BSE) image of region in A0094-01 showing OA5. OA = opaque assemblage, and Po = pyrrhotite.

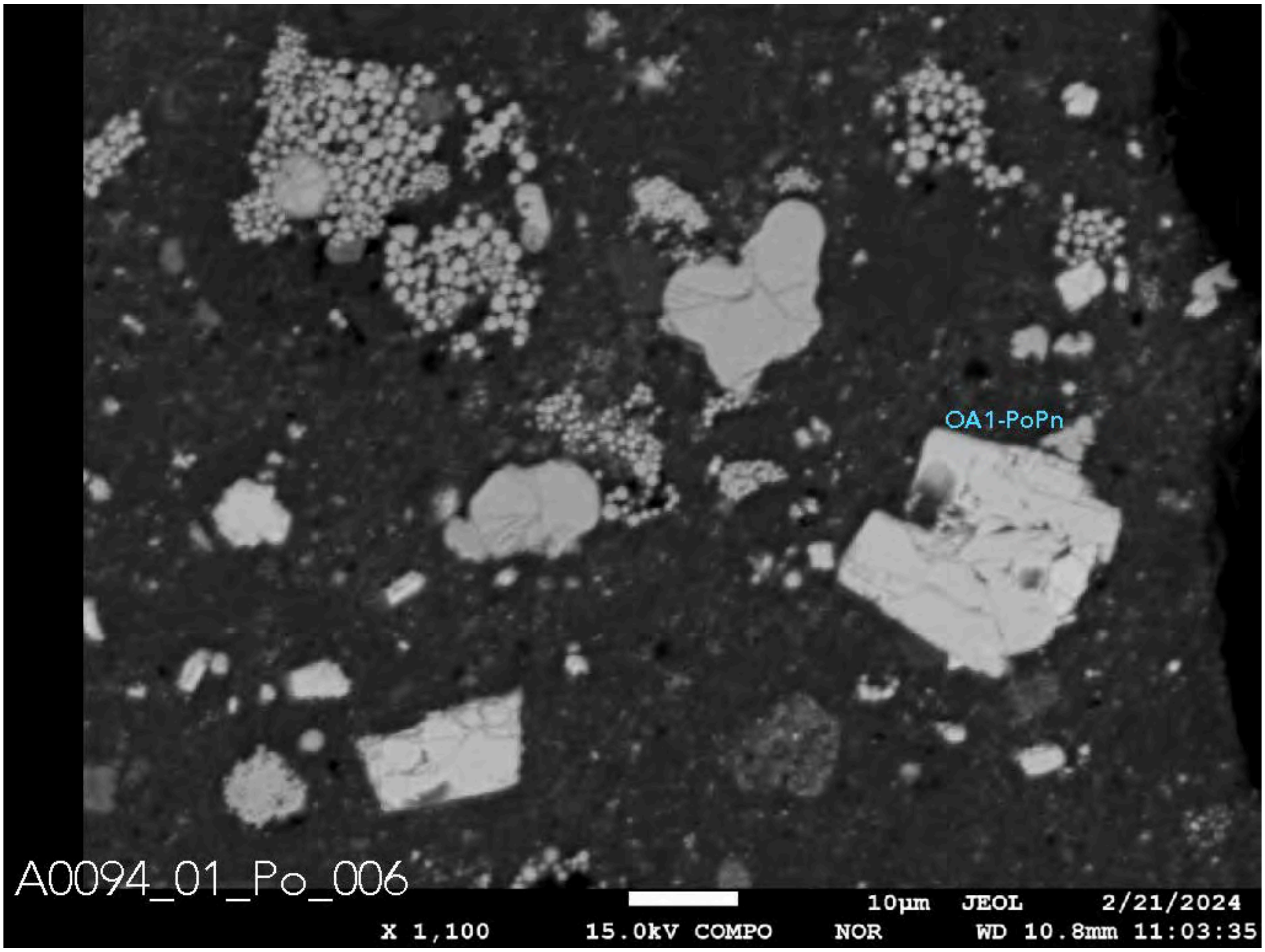

Supplementary Figure 110. Backscattered electron (BSE) image of OA1 in A0094-01. OA = opaque assemblage, Po = pyrrhotite, and Pn = pentlandite.

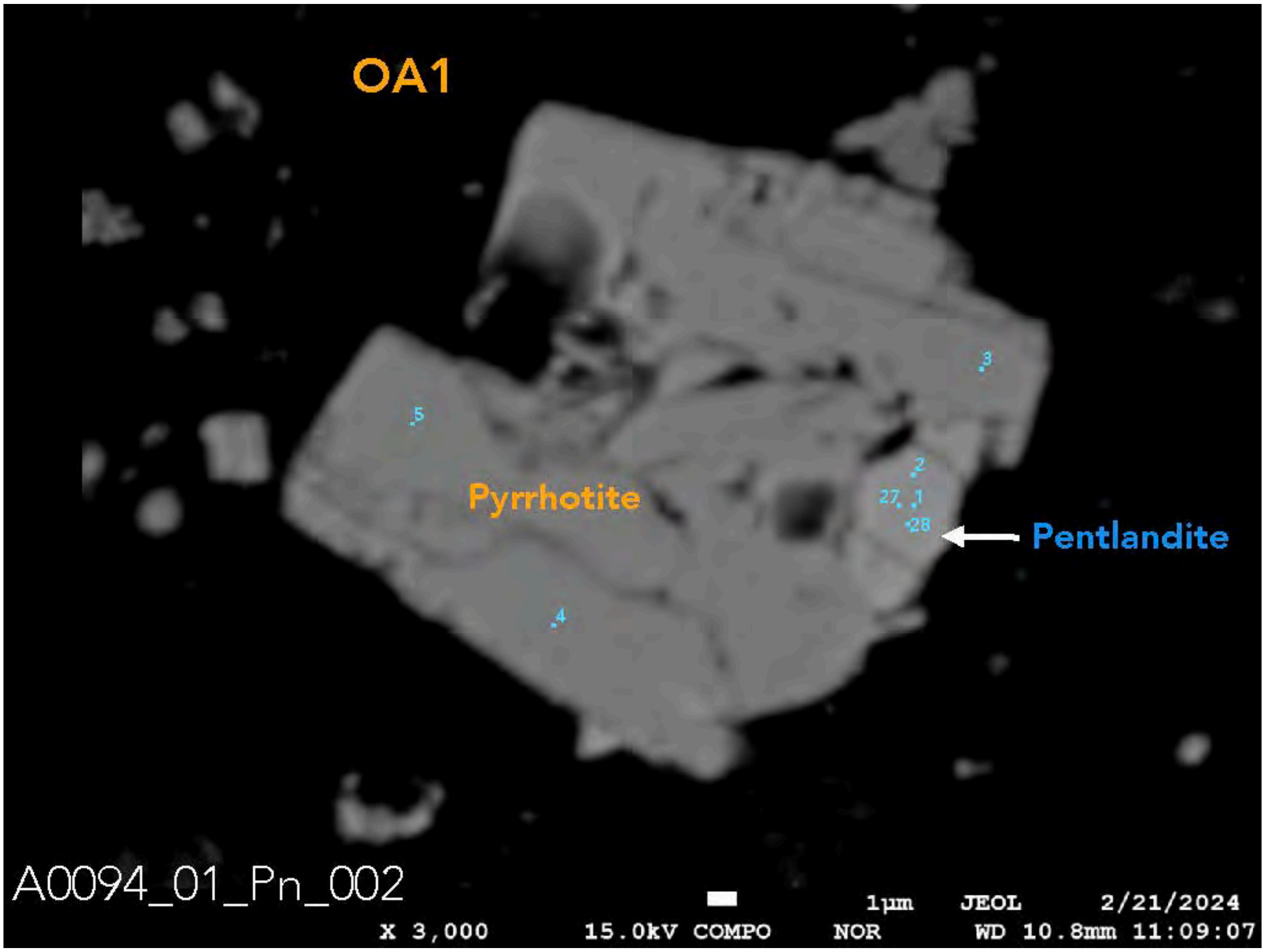

Supplementary Figure 111. Backscattered electron (BSE) image of OA1 in A0094-01, with spot analyses marked. OA = opaque assemblage.

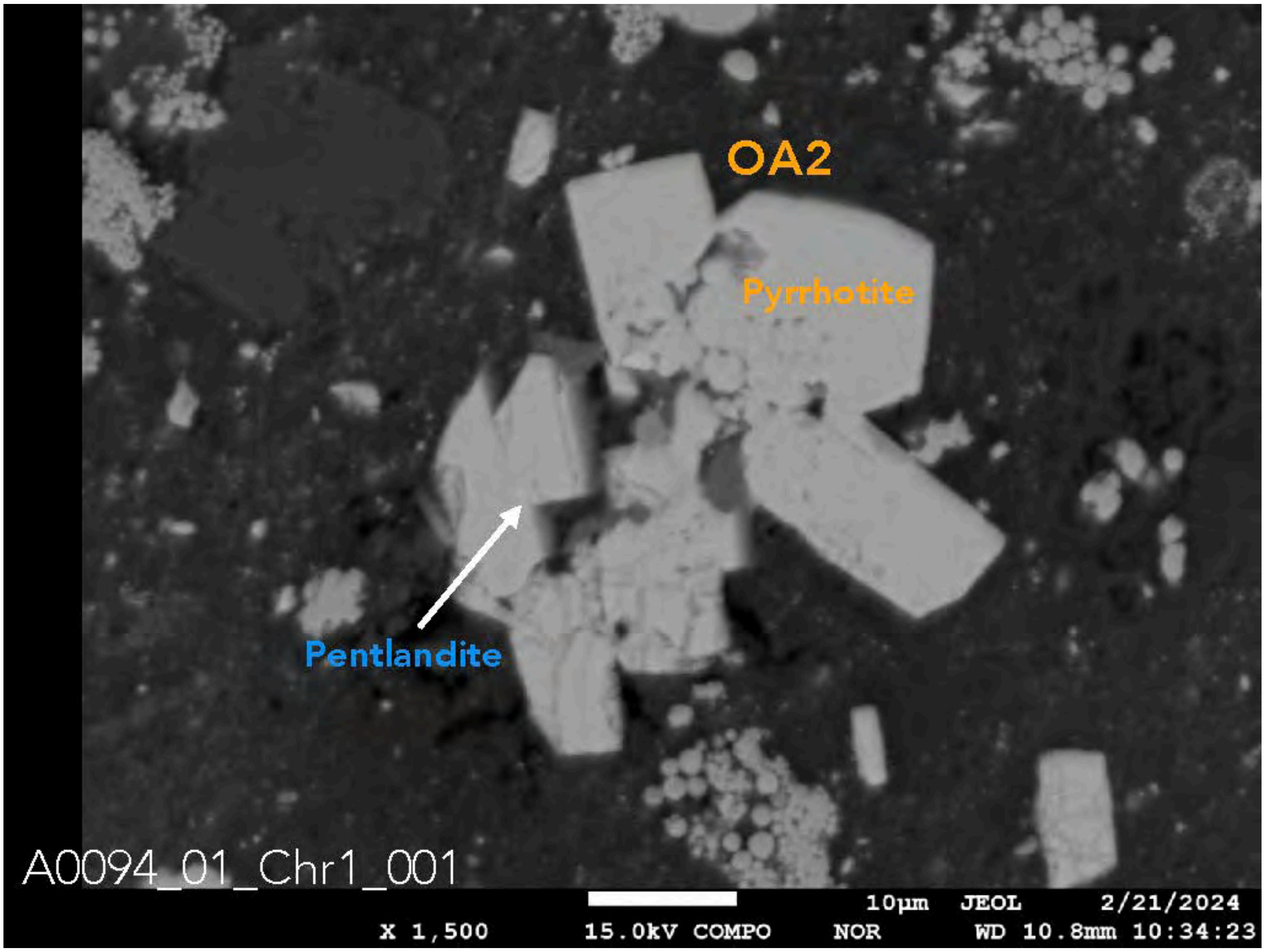

Supplementary Figure 112. Backscattered electron (BSE) image of OA2 in A0094-01. OA = opaque assemblage.

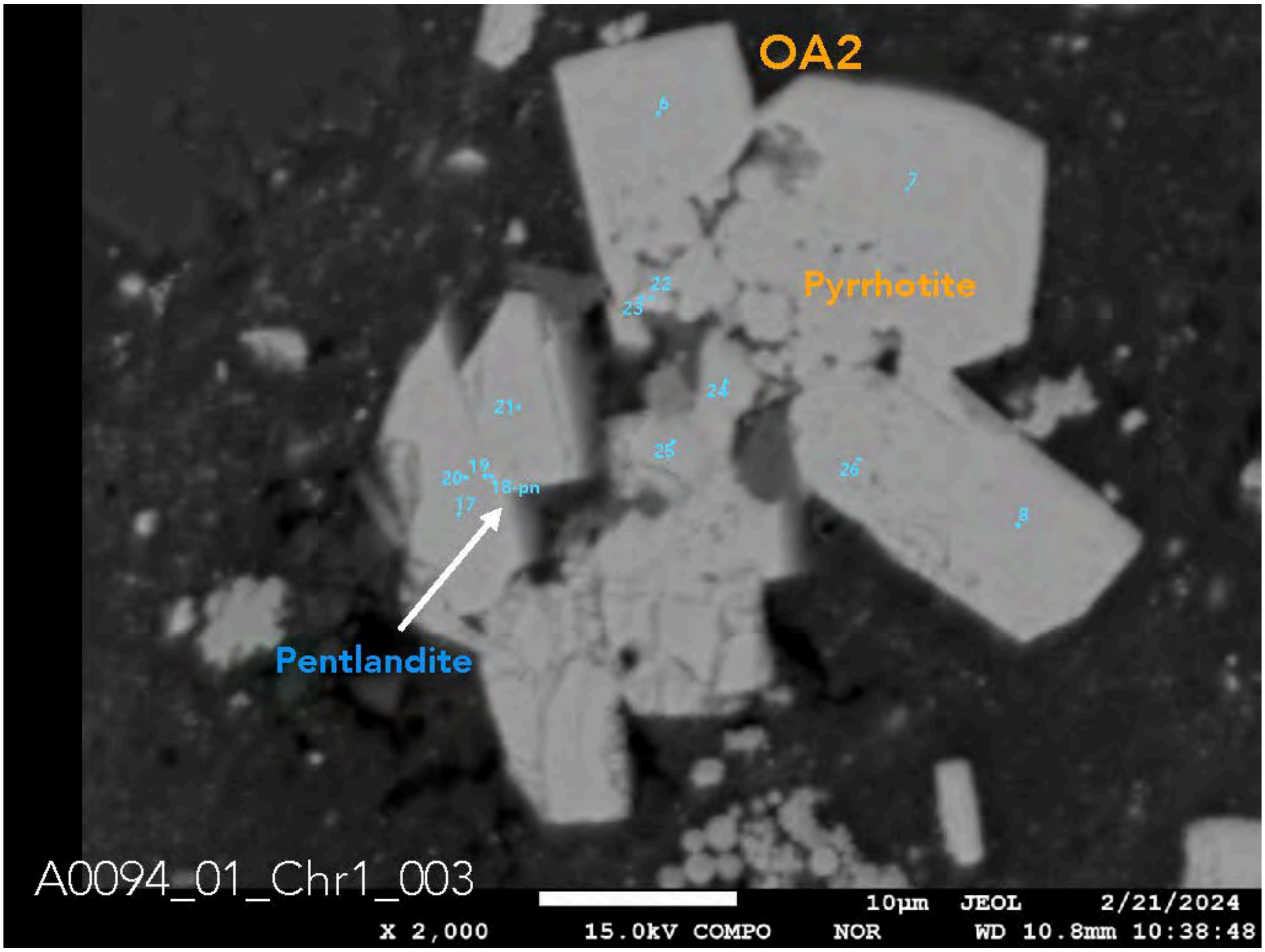

Supplementary Figure 113. Backscattered electron (BSE) image of OA2 in A0094-01, with spot analyses marked. OA = opaque assemblage.

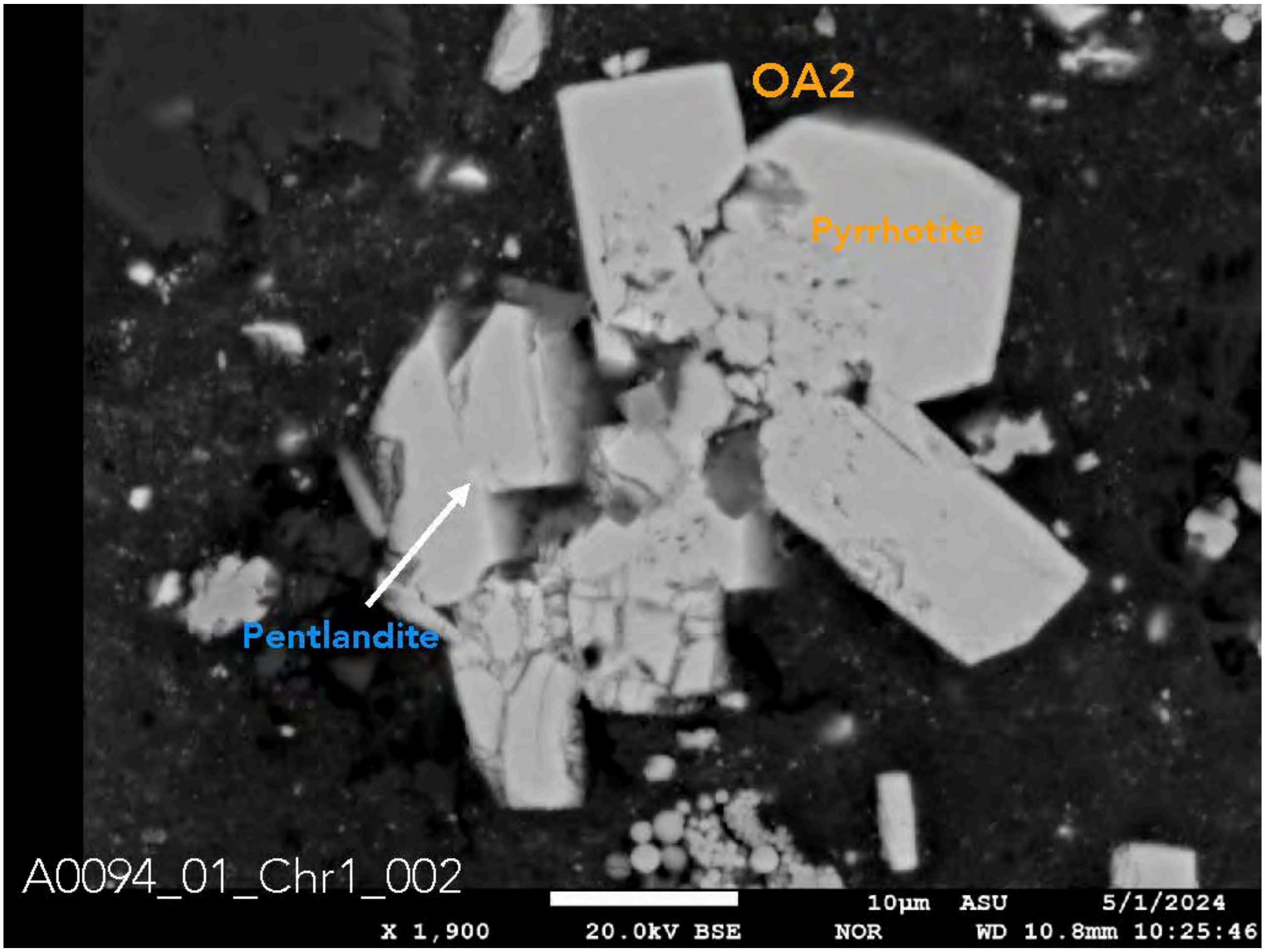

Supplementary Figure 114. Backscattered electron (BSE) image of OA2 in A0094-01. OA = opaque assemblage.

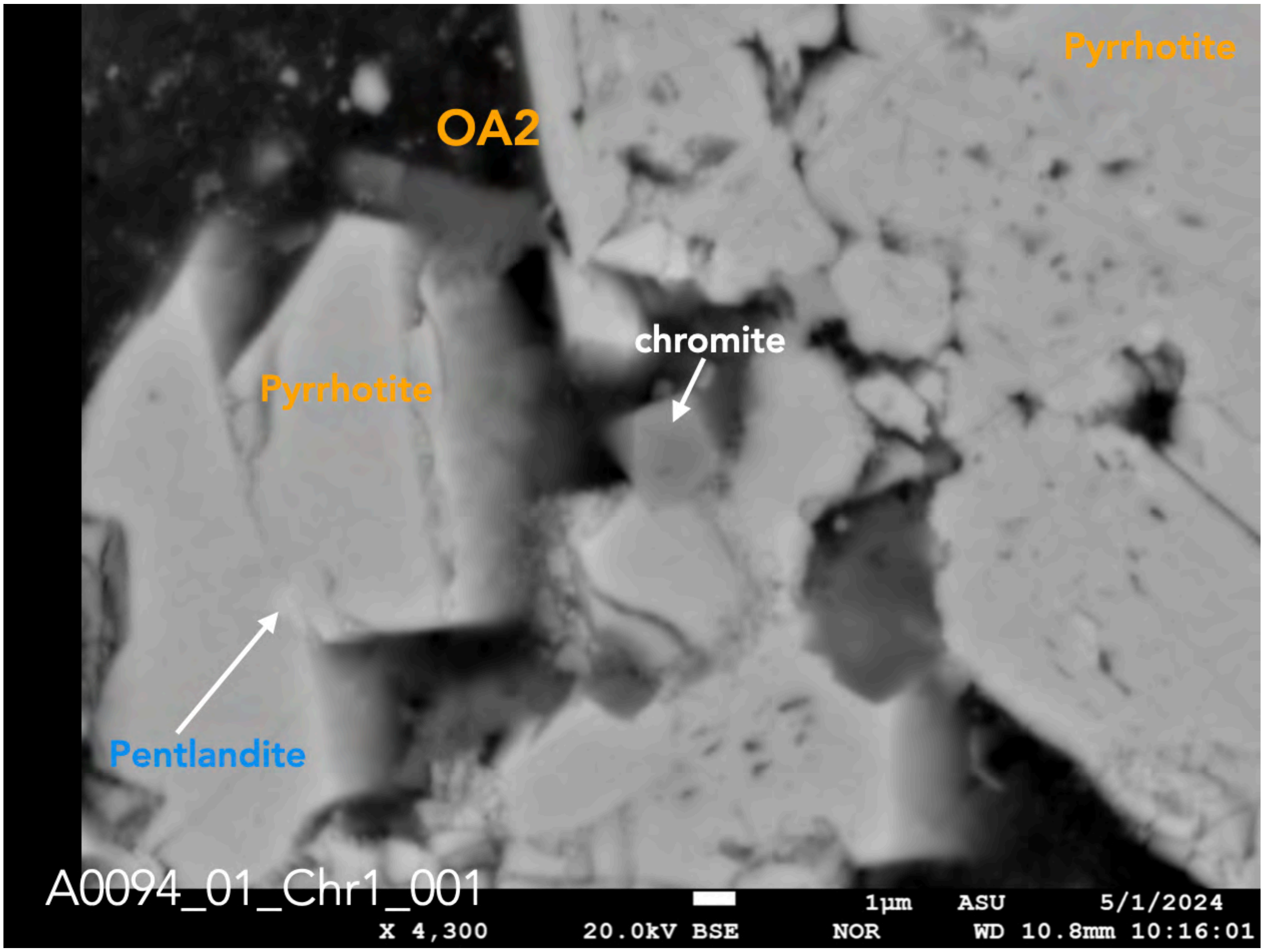

Supplementary Figure 115. Backscattered electron (BSE) image of OA2 in A0094-01. OA = opaque assemblage.

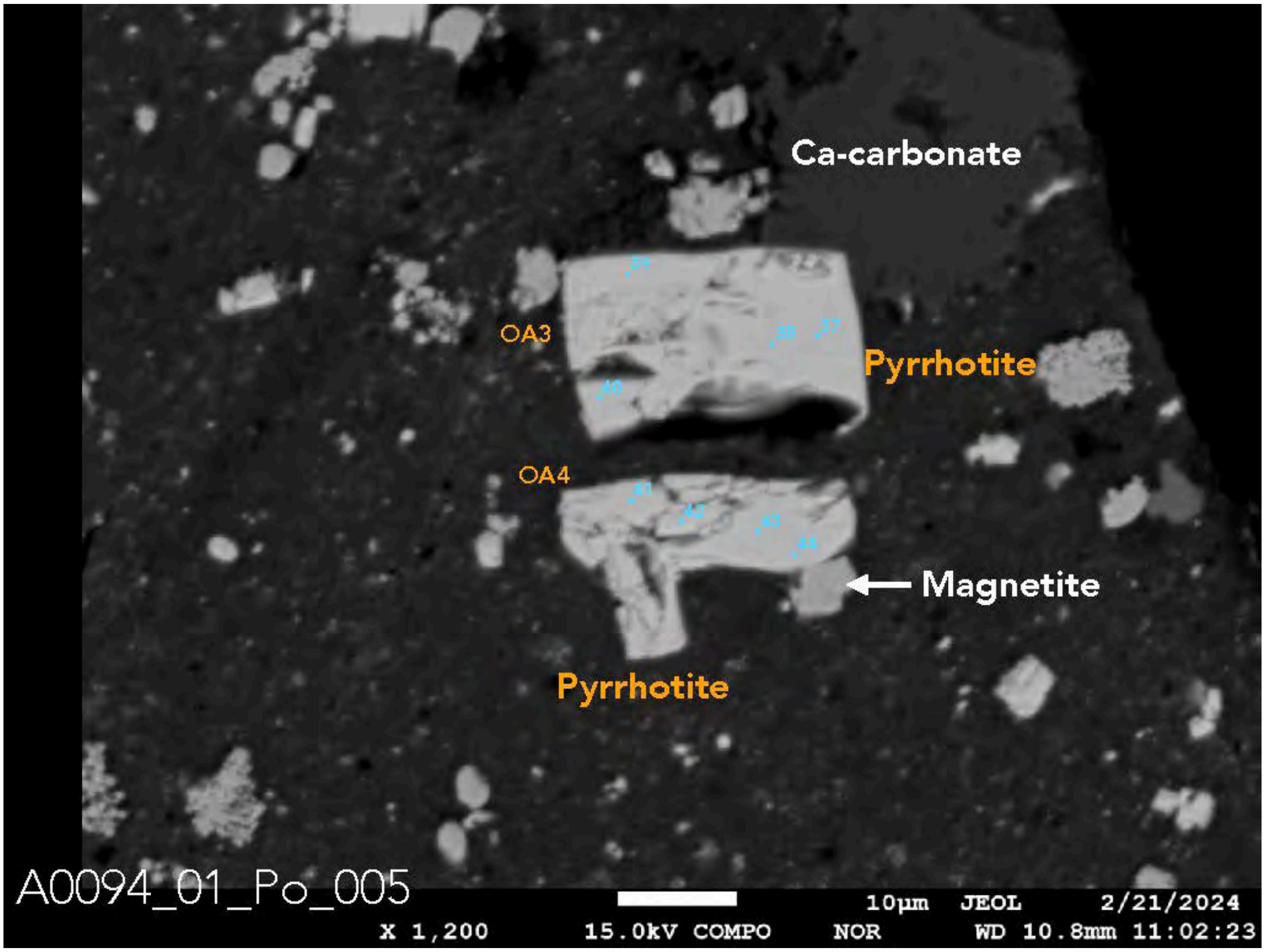

Supplementary Figure 116. Backscattered electron (BSE) image of OA3 and OA4 in A0094-01, with spot analyses marked. OA = opaque assemblage.

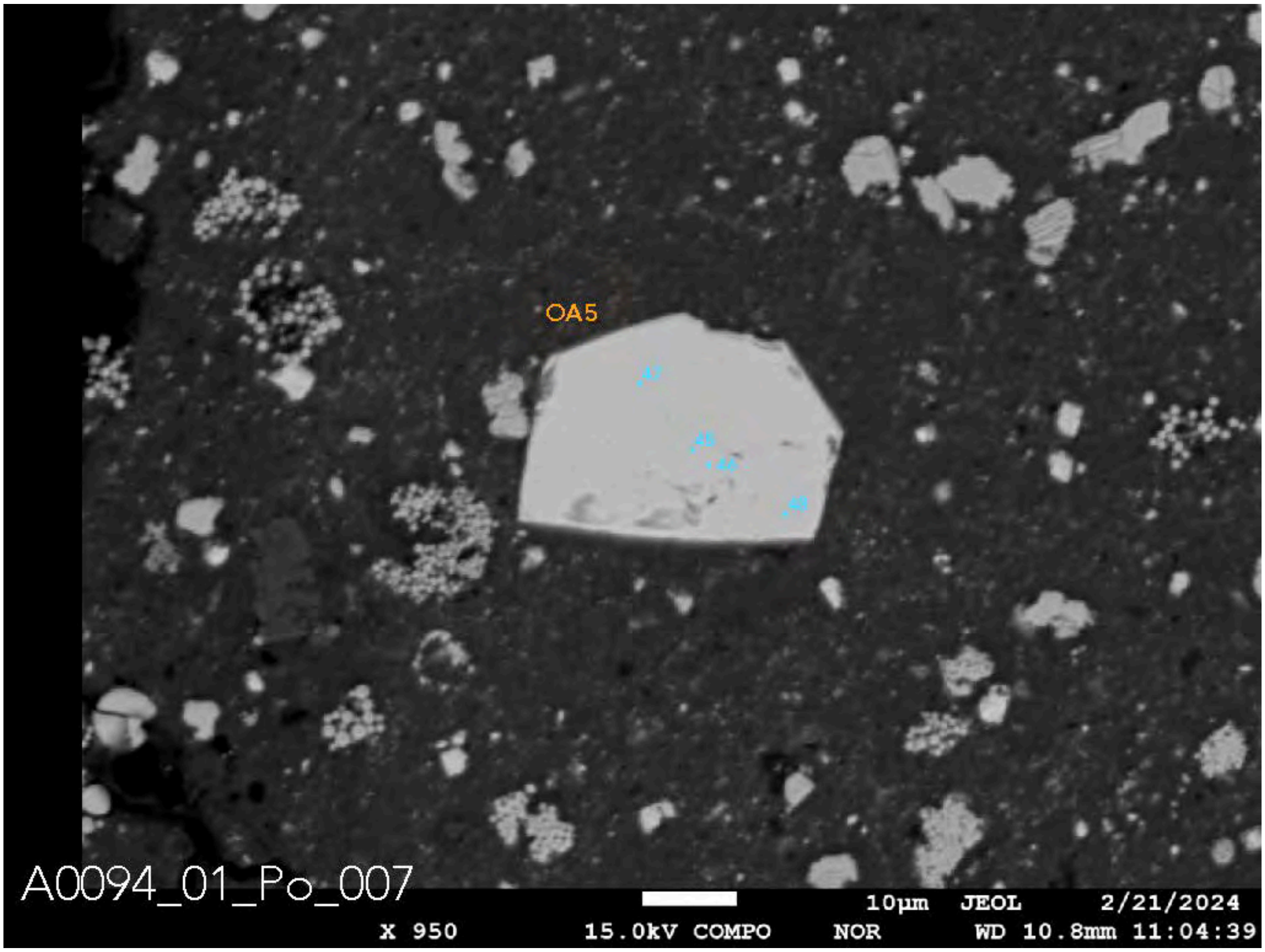

Supplementary Figure 117. Backscattered electron (BSE) image of OA5 in A0094-01, with spot analyses marked. OA = opaque assemblage.

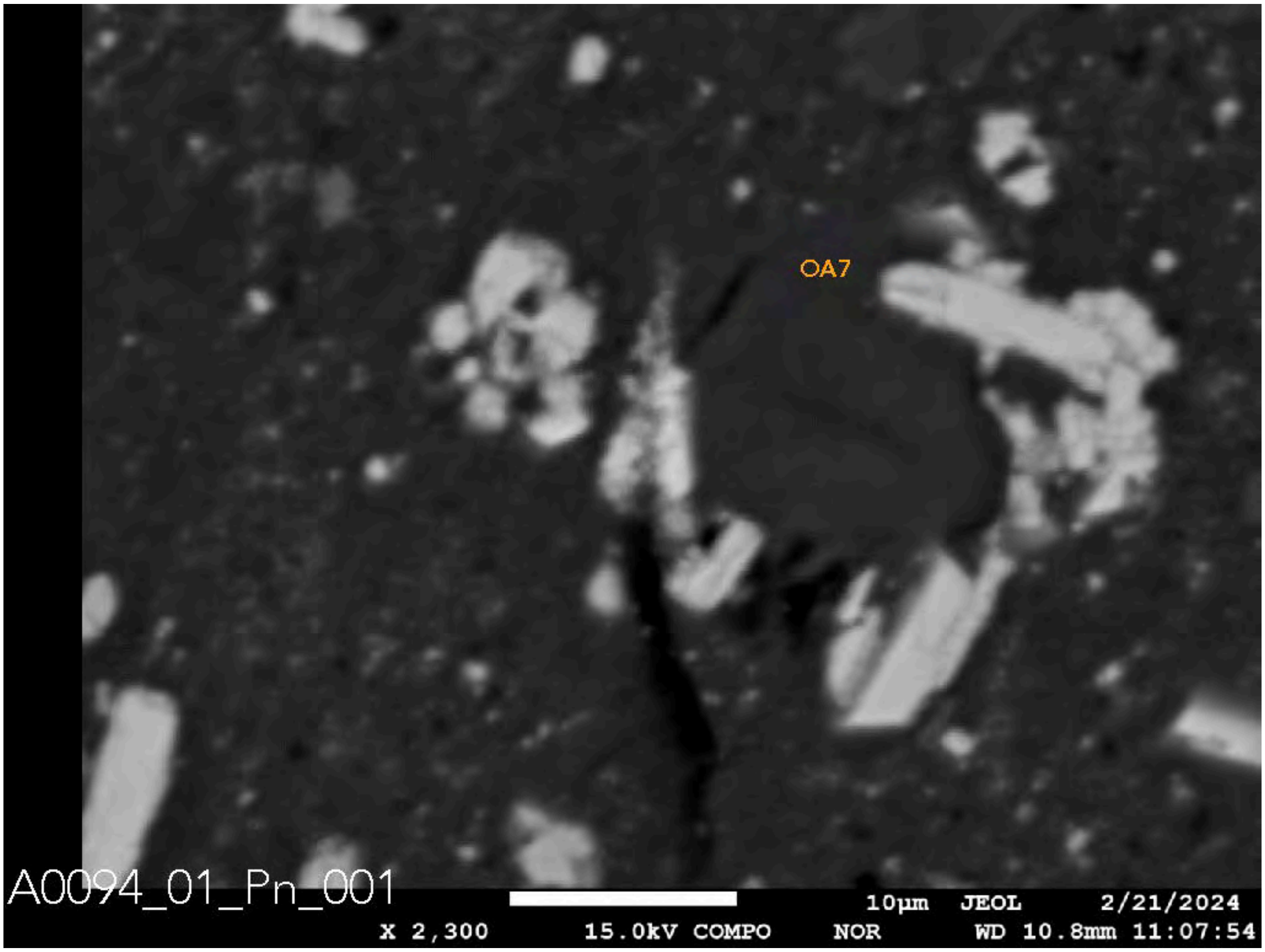

Supplementary Figure 118. Backscattered electron (BSE) image of OA7 in A0094-01. OA = opaque assemblage.

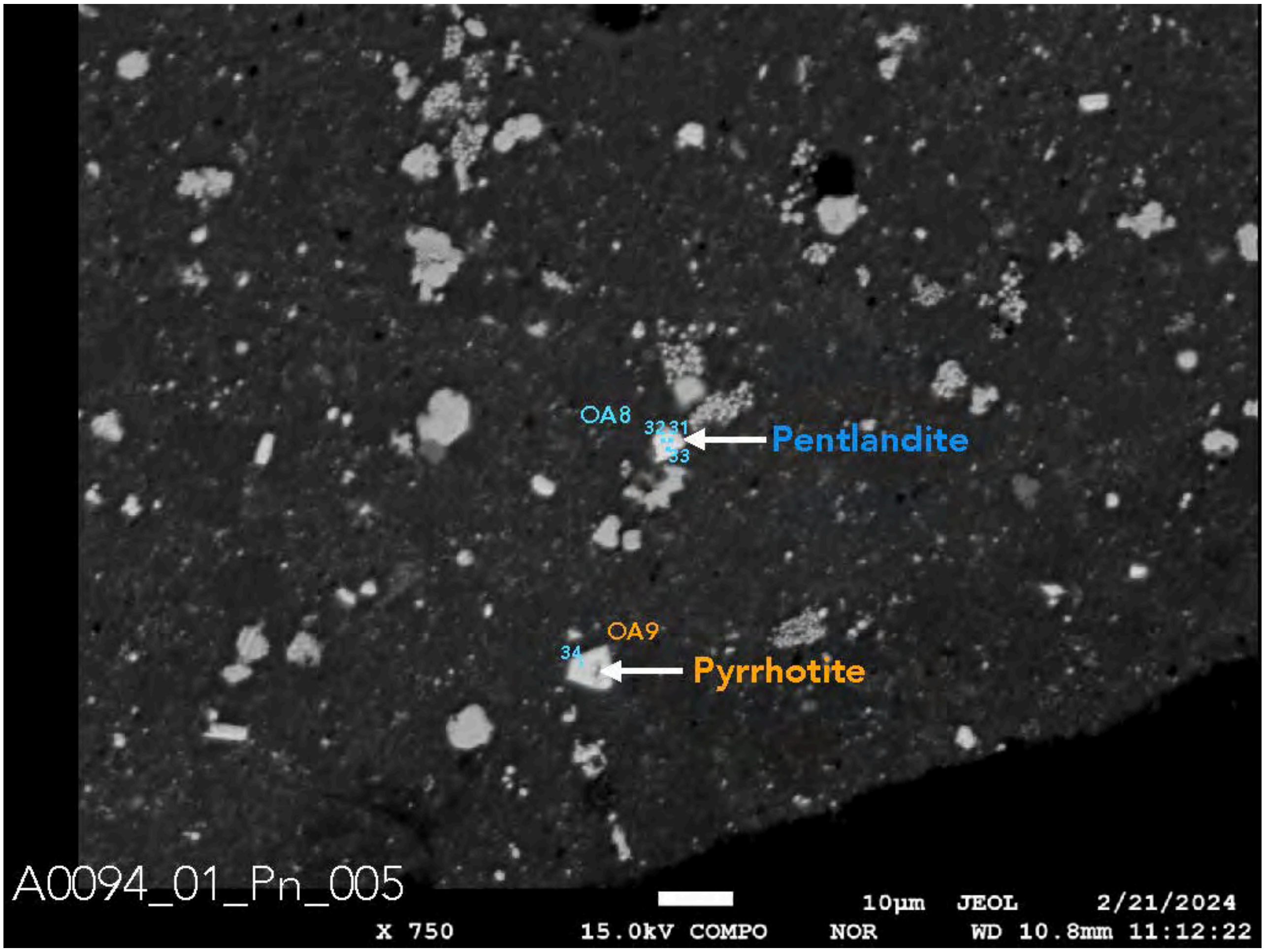

Supplementary Figure 119. Backscattered electron (BSE) image of OA8 and OA9 in A0094-01, with spot analyses marked. OA = opaque assemblage

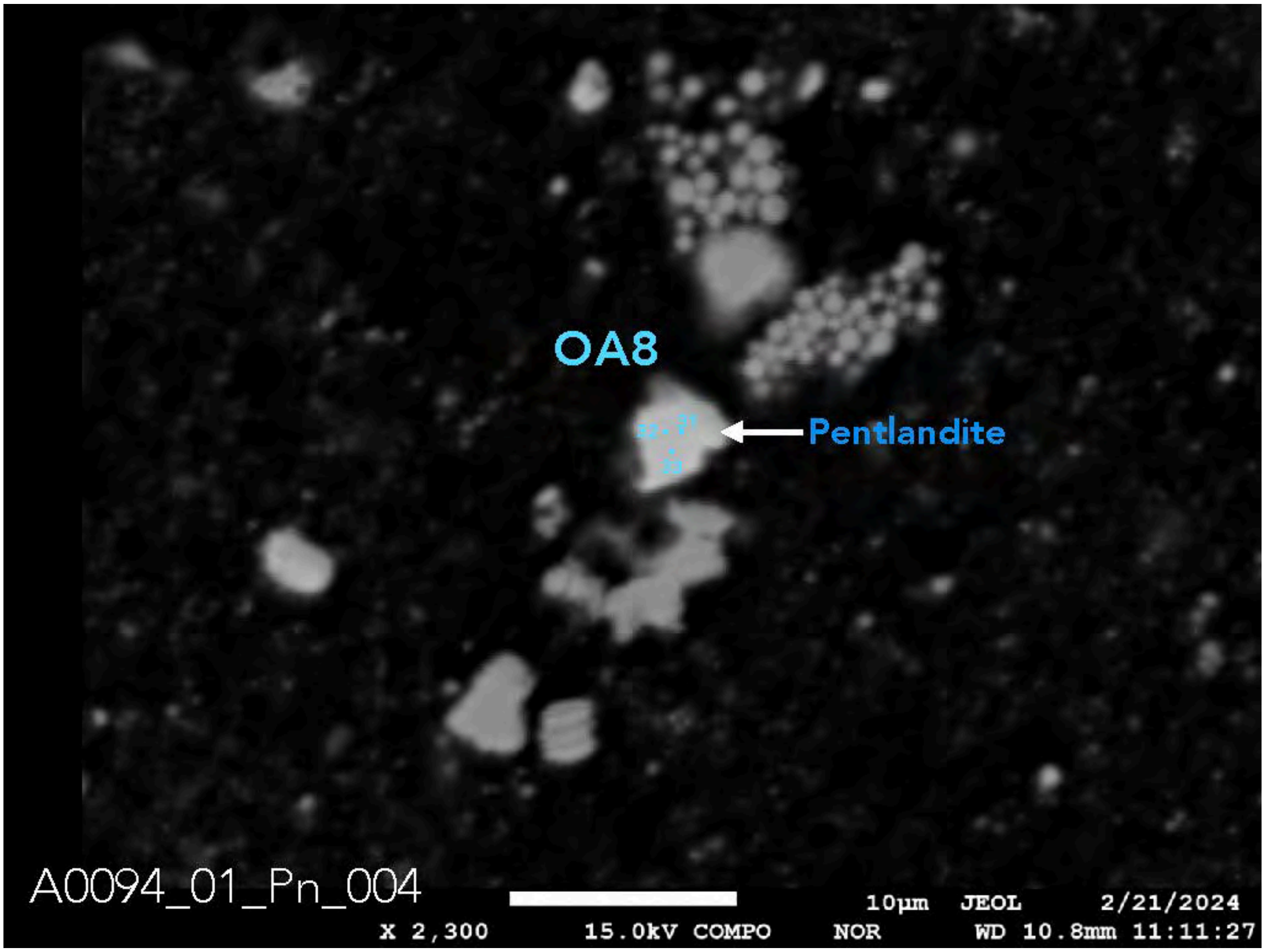

Supplementary Figure 120. Backscattered electron (BSE) image of OA8 in A0094-01, with spot analyses marked. OA = opaque assemblage.

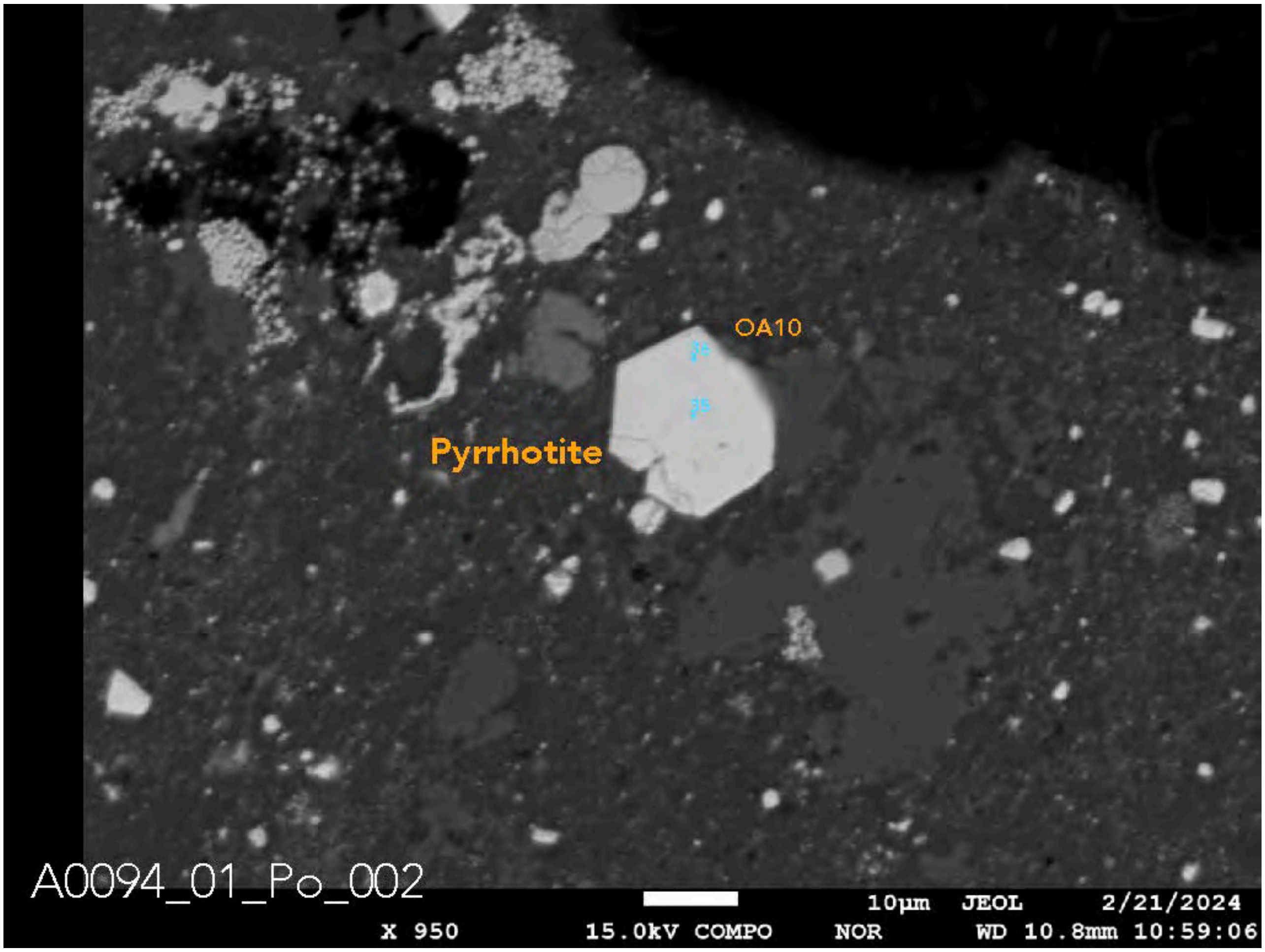

Supplementary Figure 121. Backscattered electron (BSE) image of OA10 in A0094-01, with spot analyses marked. OA = opaque assemblage.

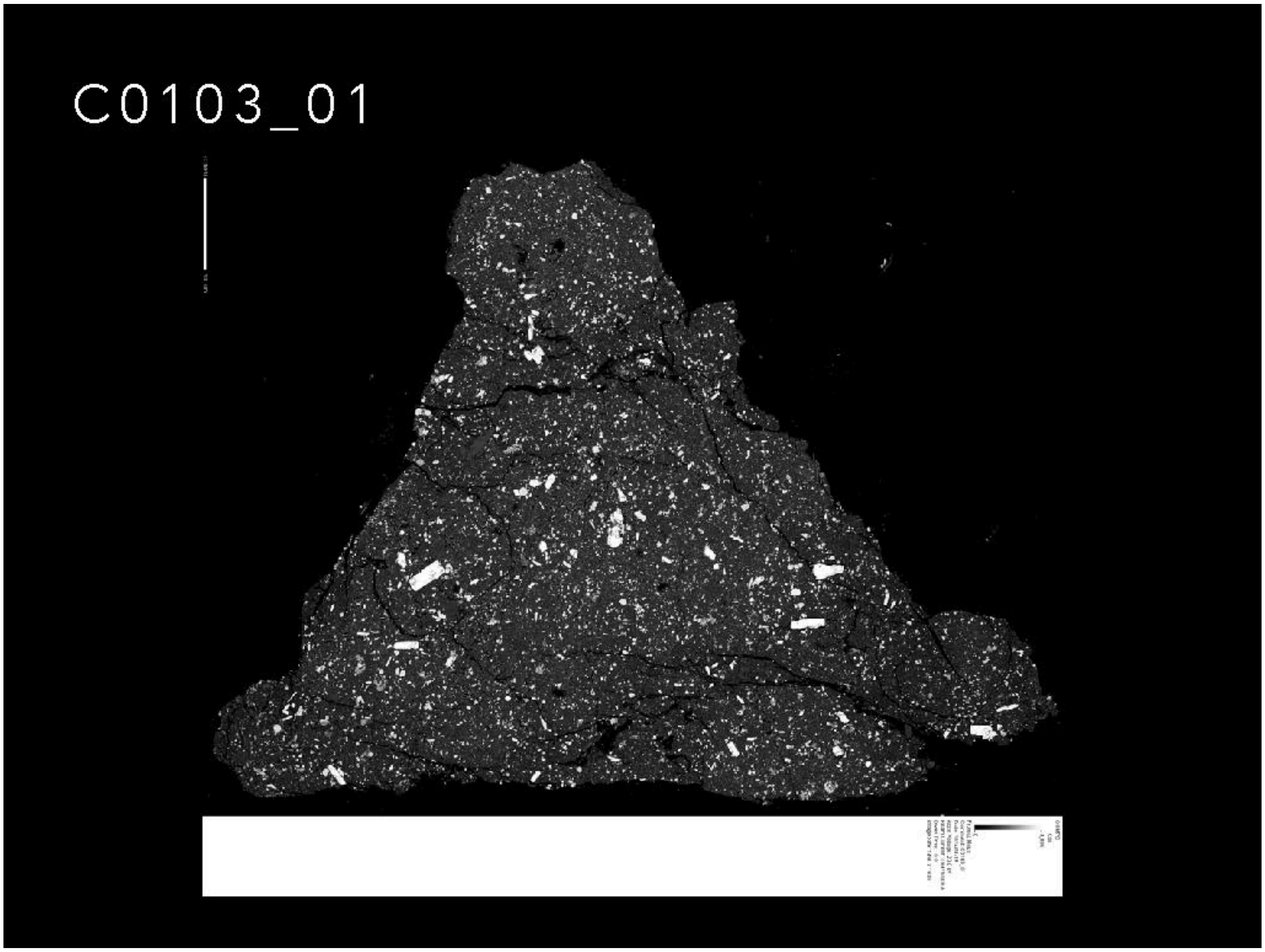

Supplementary Figure 122. Full backscattered electron (BSE) image of C0103-01.

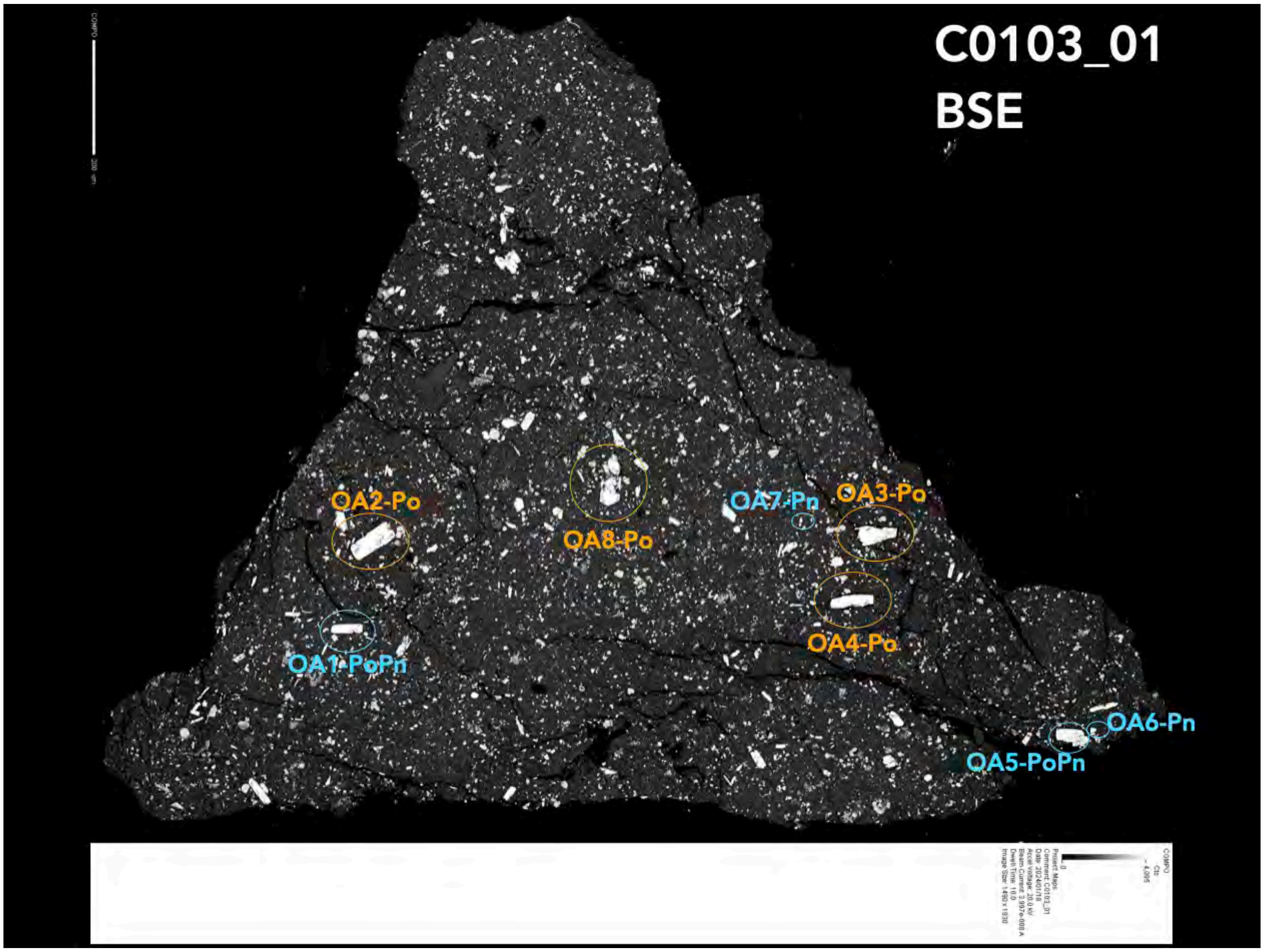

Supplementary Figure 123. Full backscattered electron (BSE) image of C0103-01 with location of all sulfides imaged in detail and analyzed marked. Where OA = opaque assemblage, Po = pyrrhotite, and Pn = pentlandite.

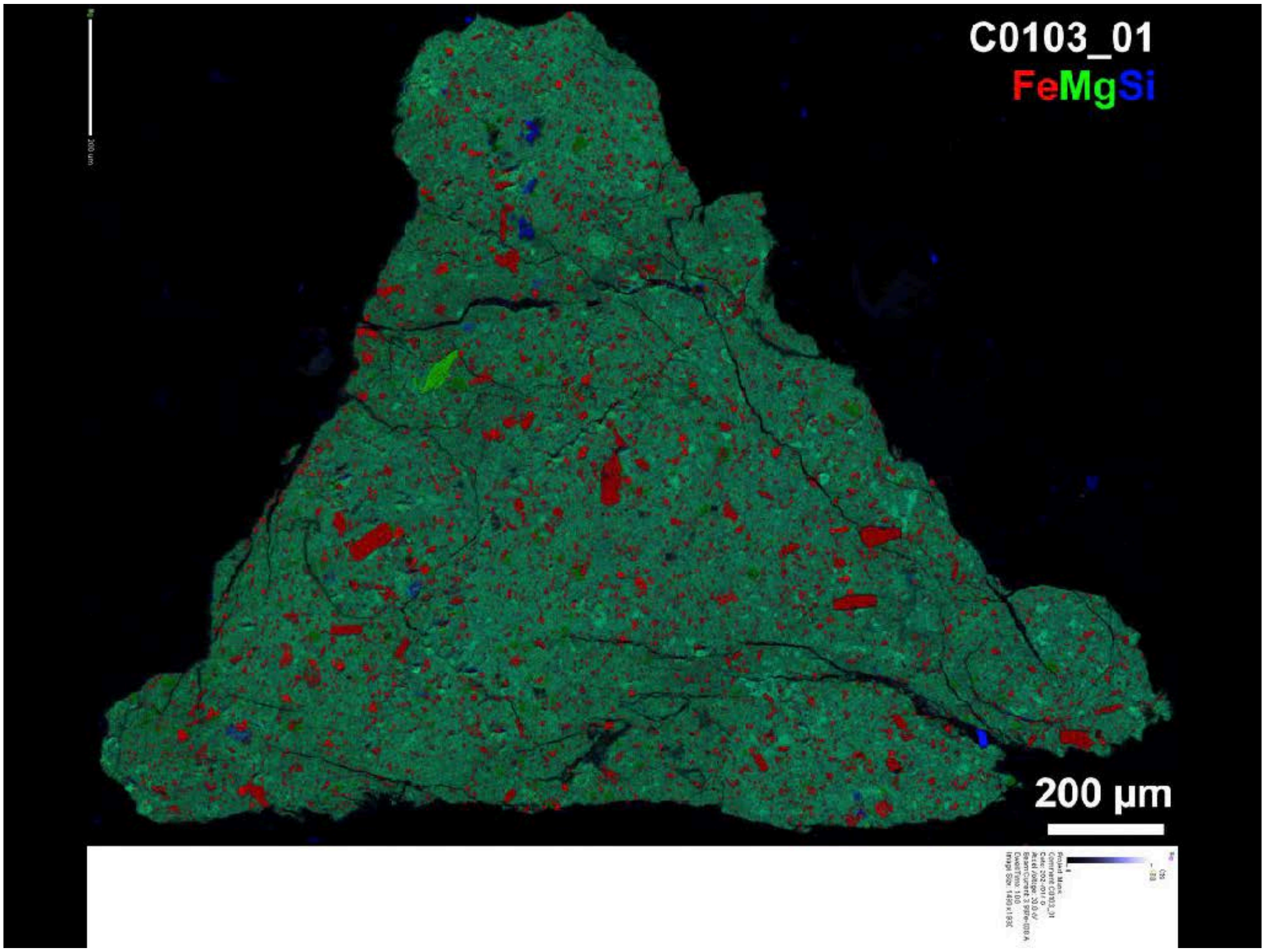

Supplementary Figure 124. Full composite X-ray element RGB image (Fe = red, Mg = green, and Si = blue) of C0103-01.

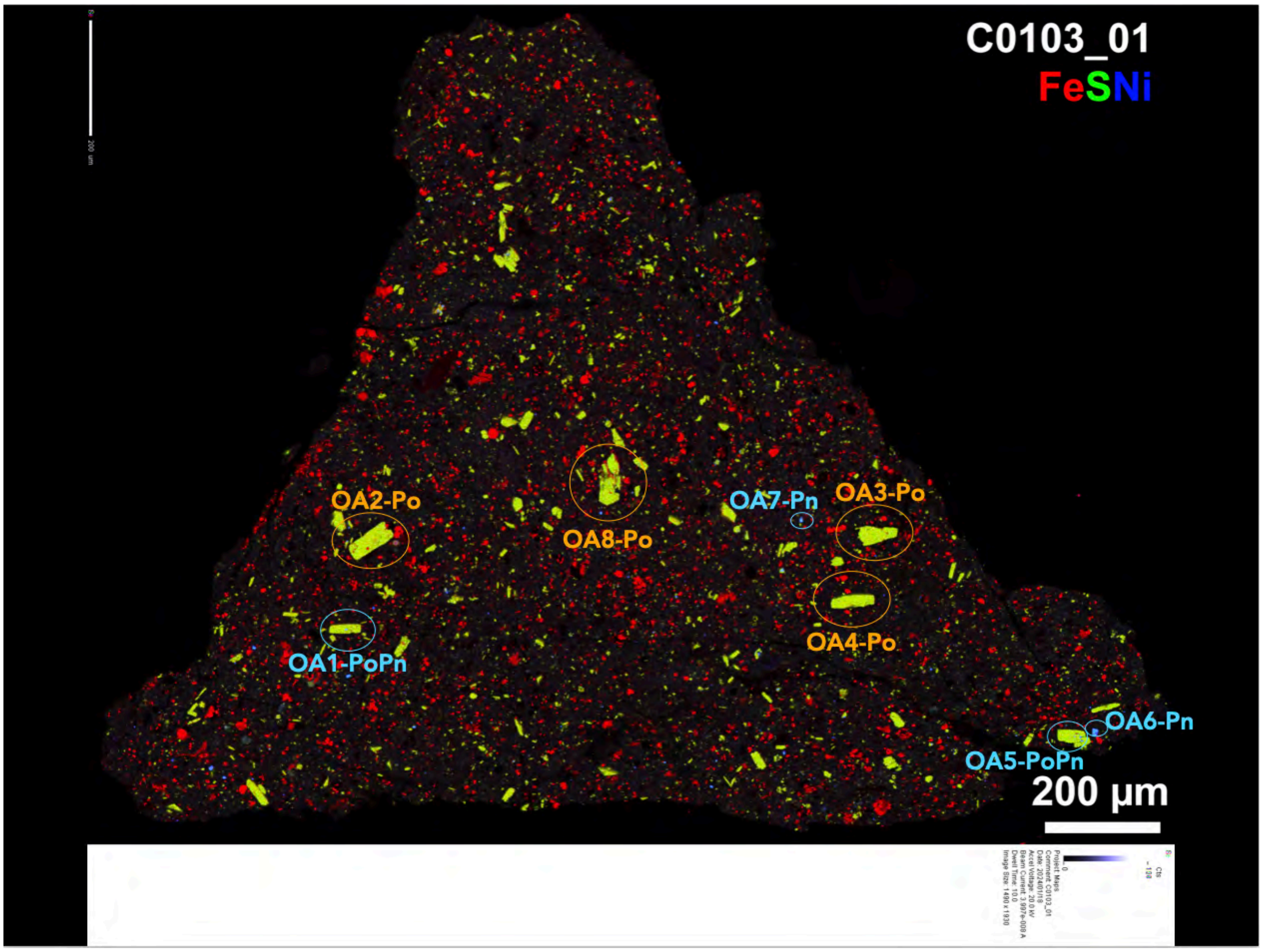

Supplementary Figure 125. Full composite X-ray element RGB image (Fe = red, S = green, and Ni = blue) of C0103-01, with location of all sulfides imaged in detail and analyzed marked. Where OA = opaque assemblage, Po = pyrrhotite, and Pn = pentlandite.

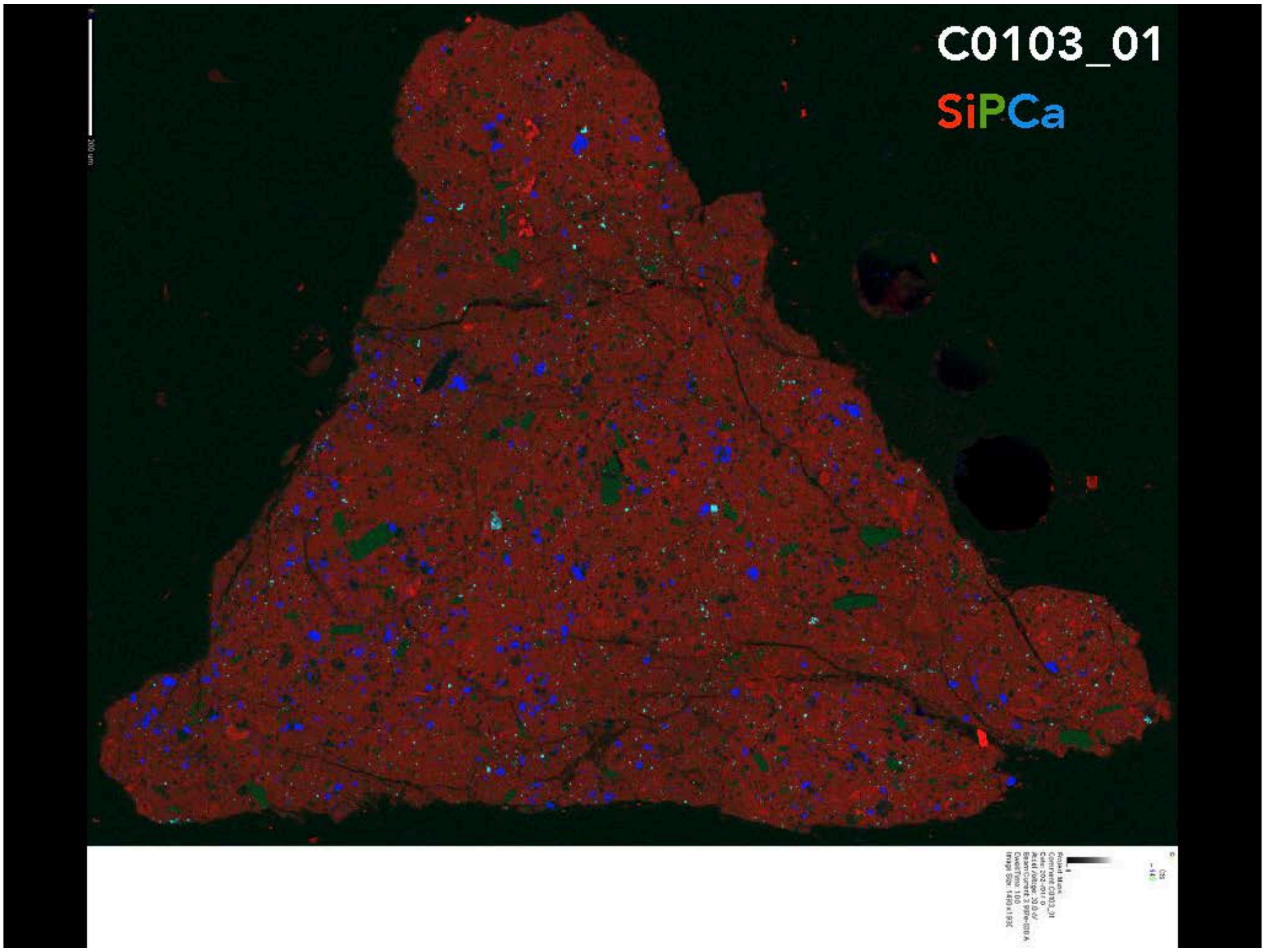

Supplementary Figure 126. Full composite X-ray element RGB image (Si = red, P = green, and Ca = blue) of C0103-01.

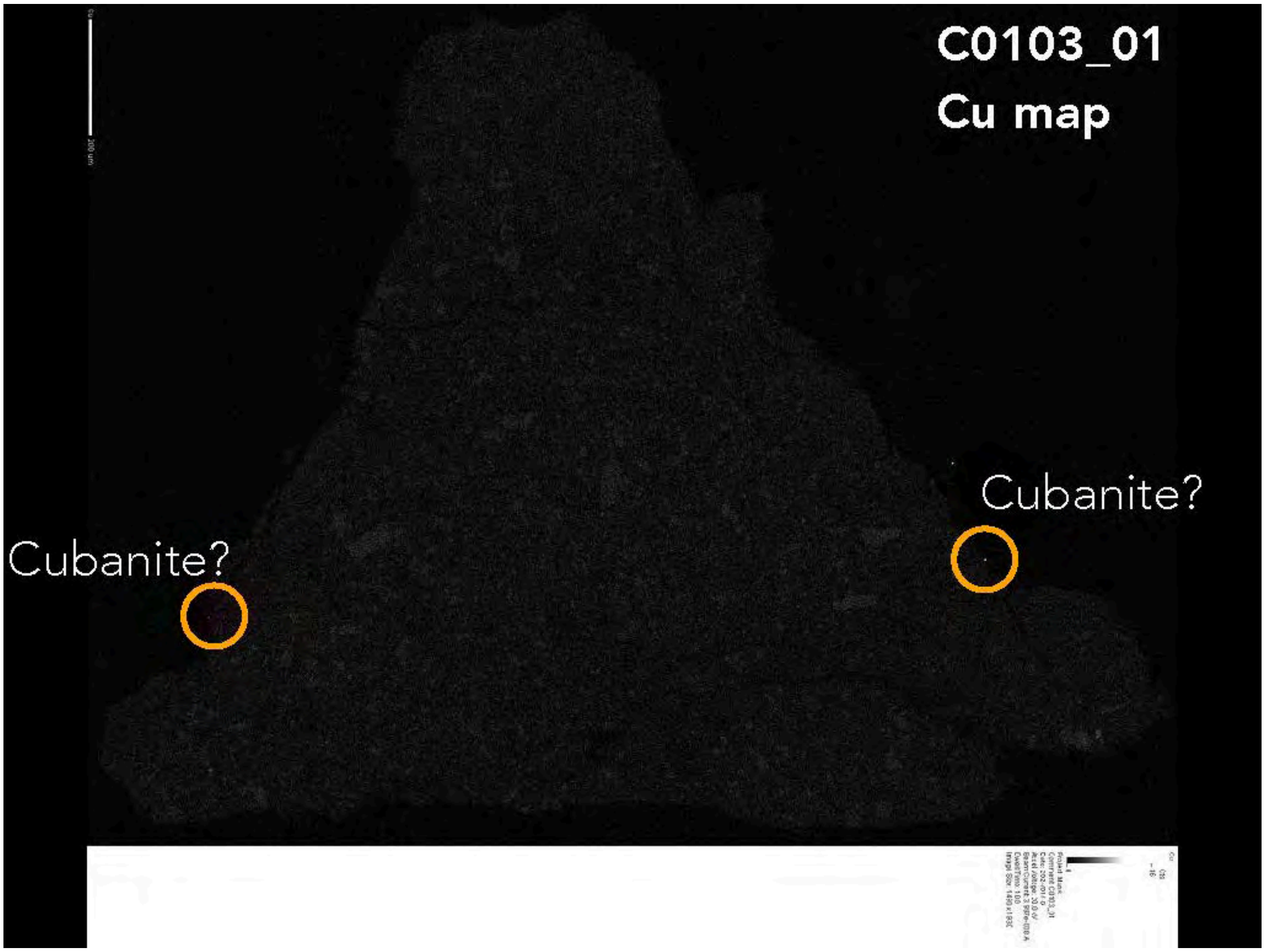

Supplementary Figure 127. Cu X-ray element map of C0103-01, showing potential Cu-sulfides too small for analysis via electron probe microanalyzer (EPMA).

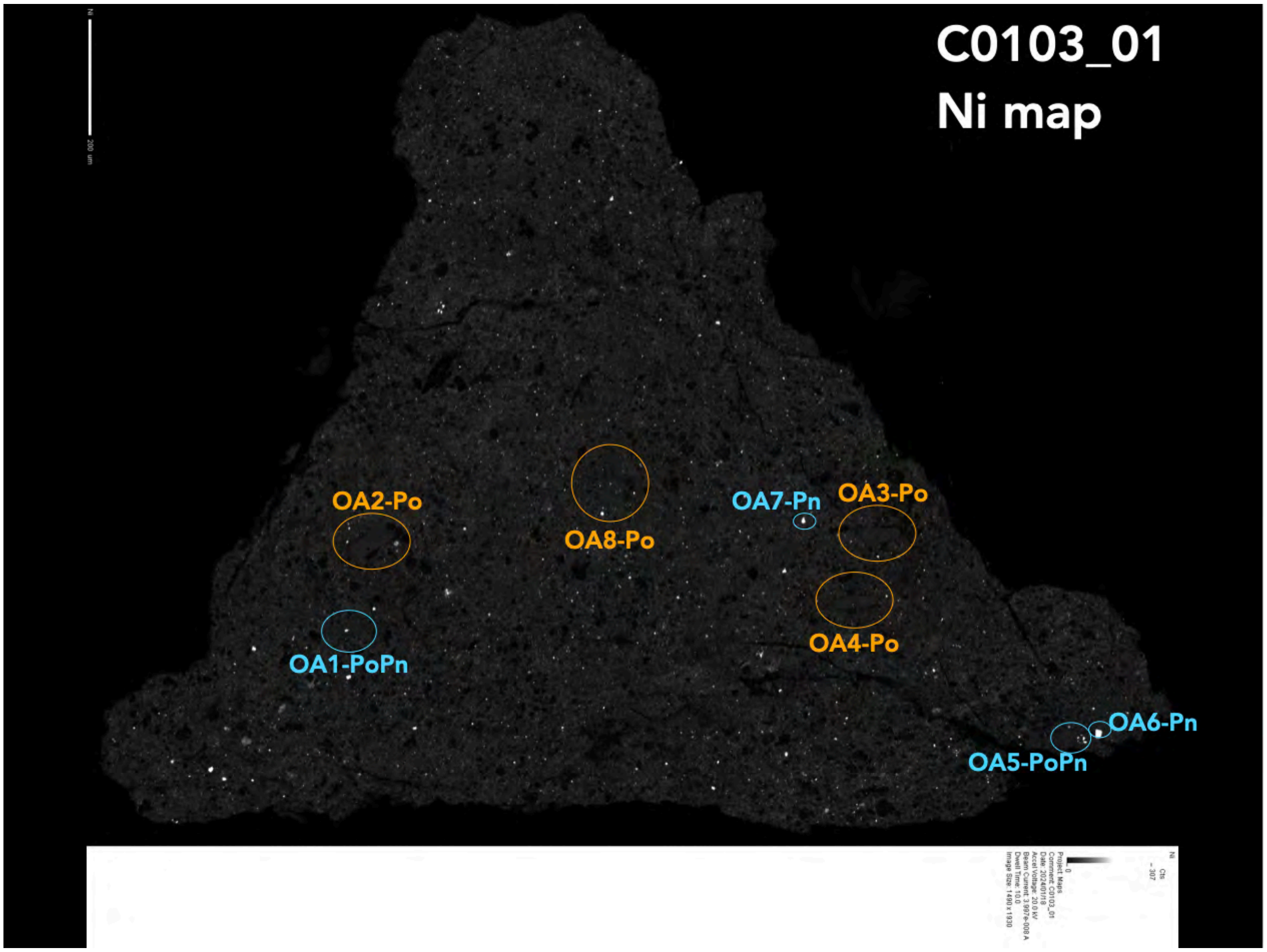

Supplementary Figure 128. Ni X-ray element map of C0103-01, with Ni-bearing sulfides analyzed marked. Where OA = opaque assemblage, Po = pyrrhotite and Pn = pentlandite.

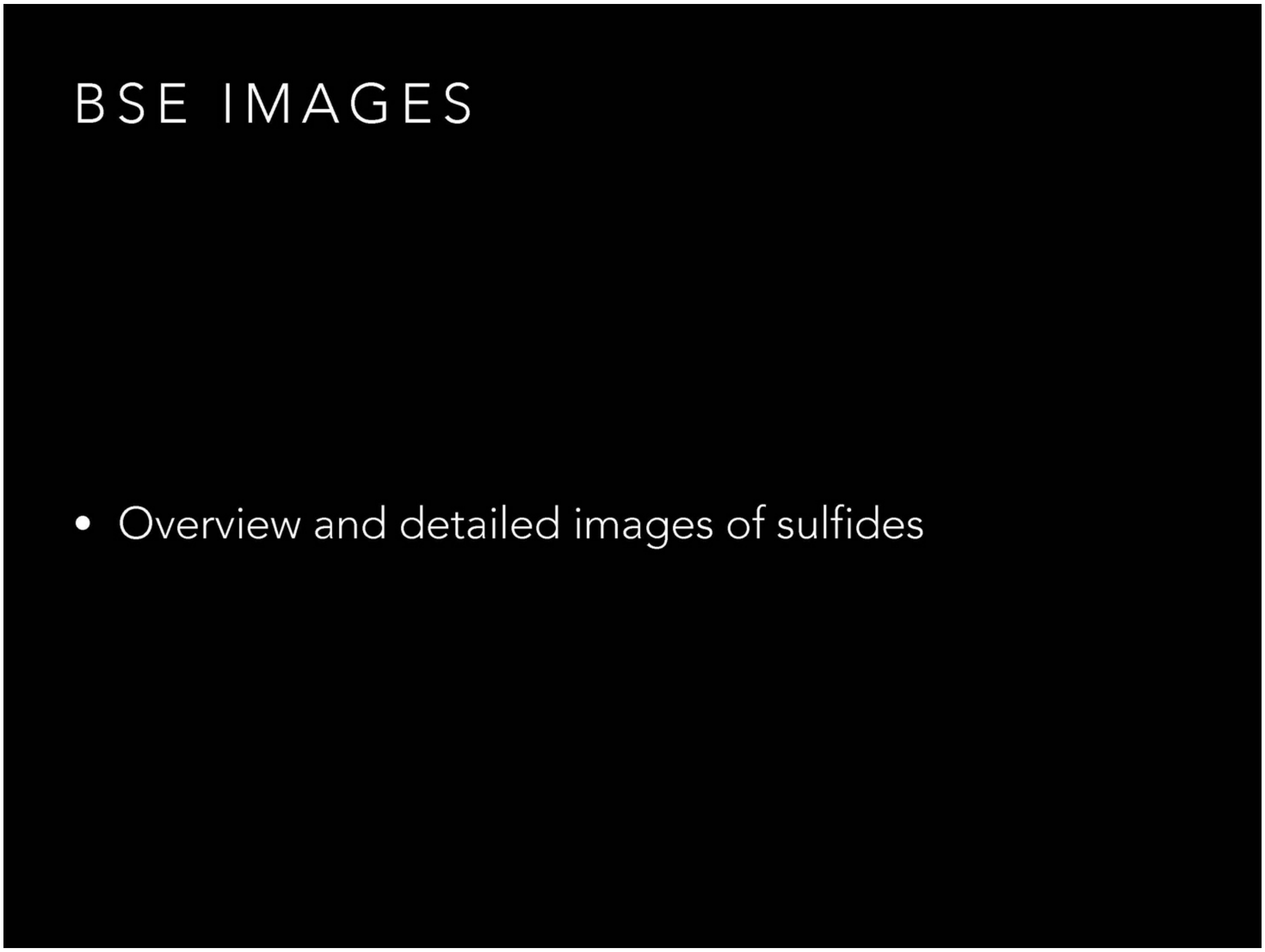

Supplementary Figure 129. Title slide for opaque assemblages in C0103-01.

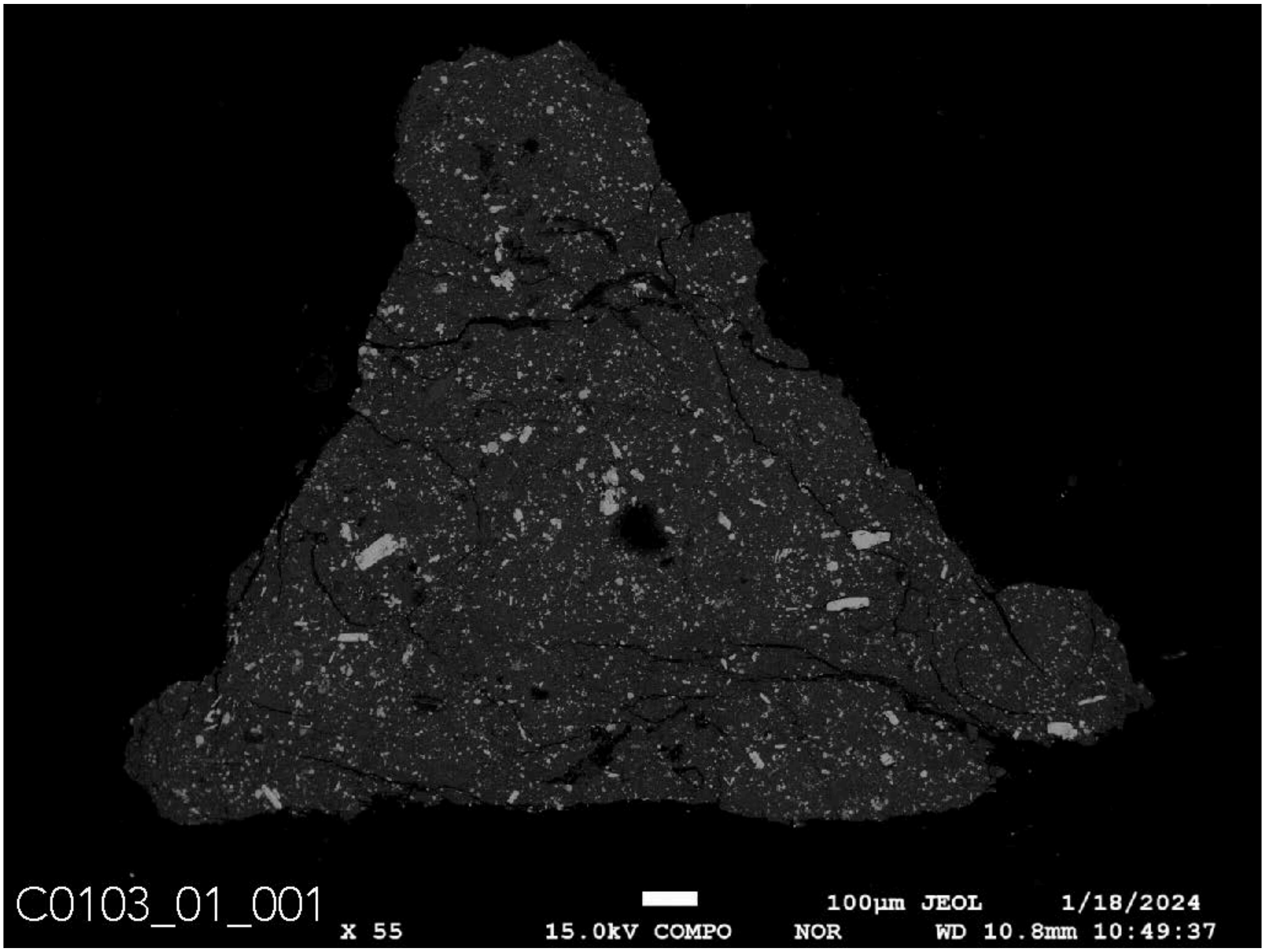

Supplementary Figure 130. Full backscattered electron (BSE) image of C0103-01.

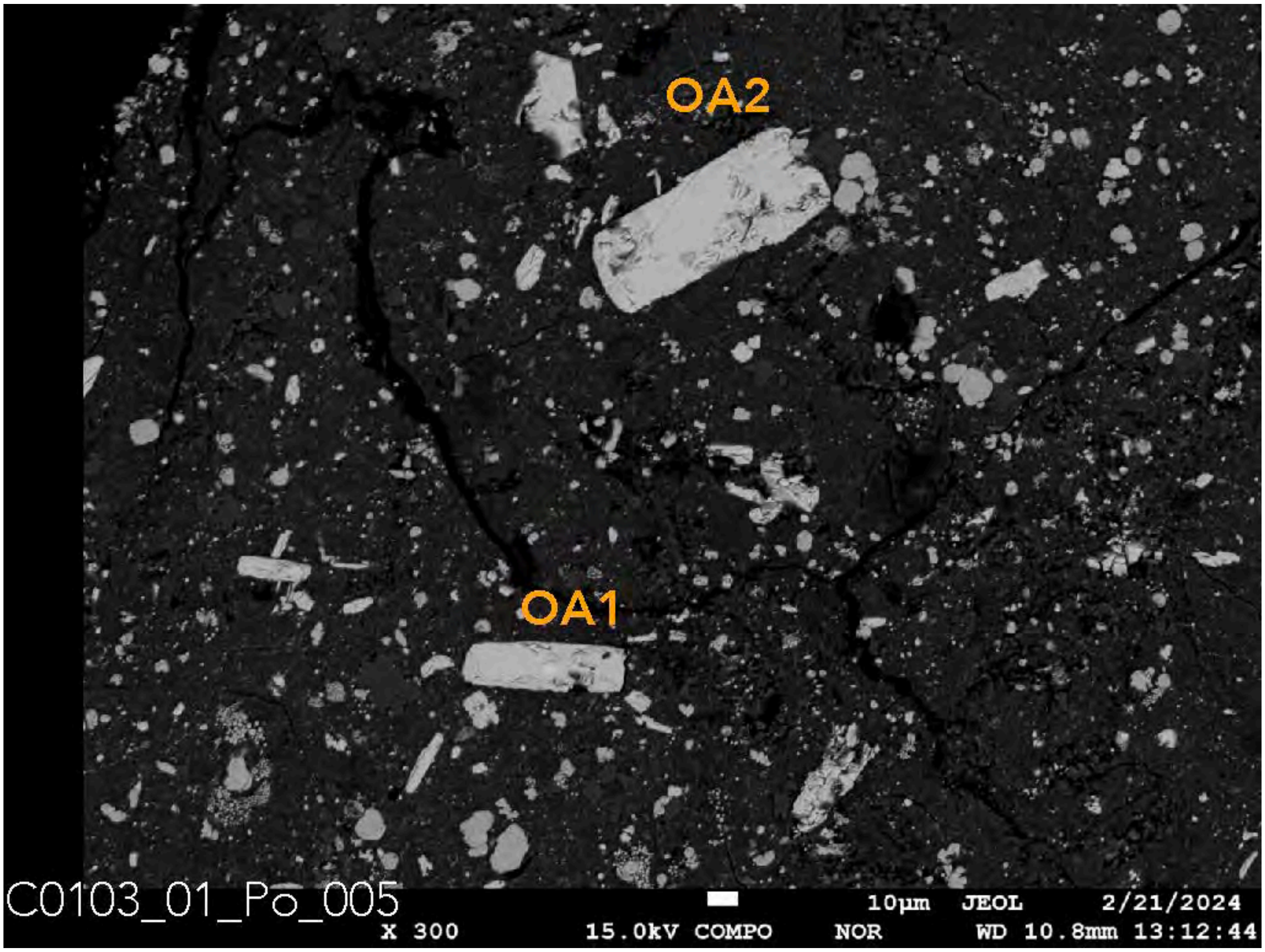

Supplementary Figure 131. Backscattered electron (BSE) image of OA1 and OA2 in C0103-01. OA = opaque assemblage.

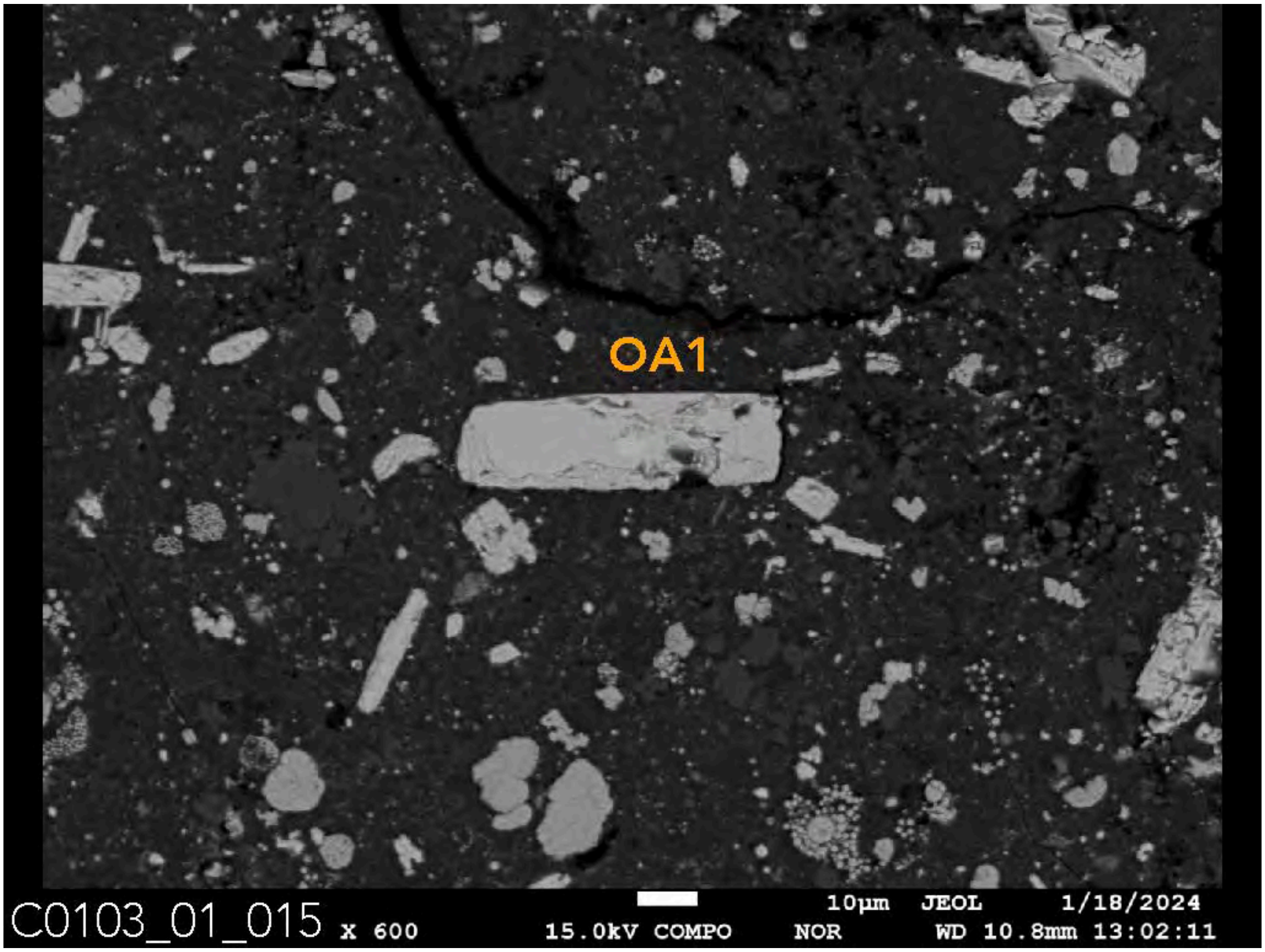

Supplementary Figure 132. Backscattered electron (BSE) image of OA1 in C0103-01. OA = opaque assemblage

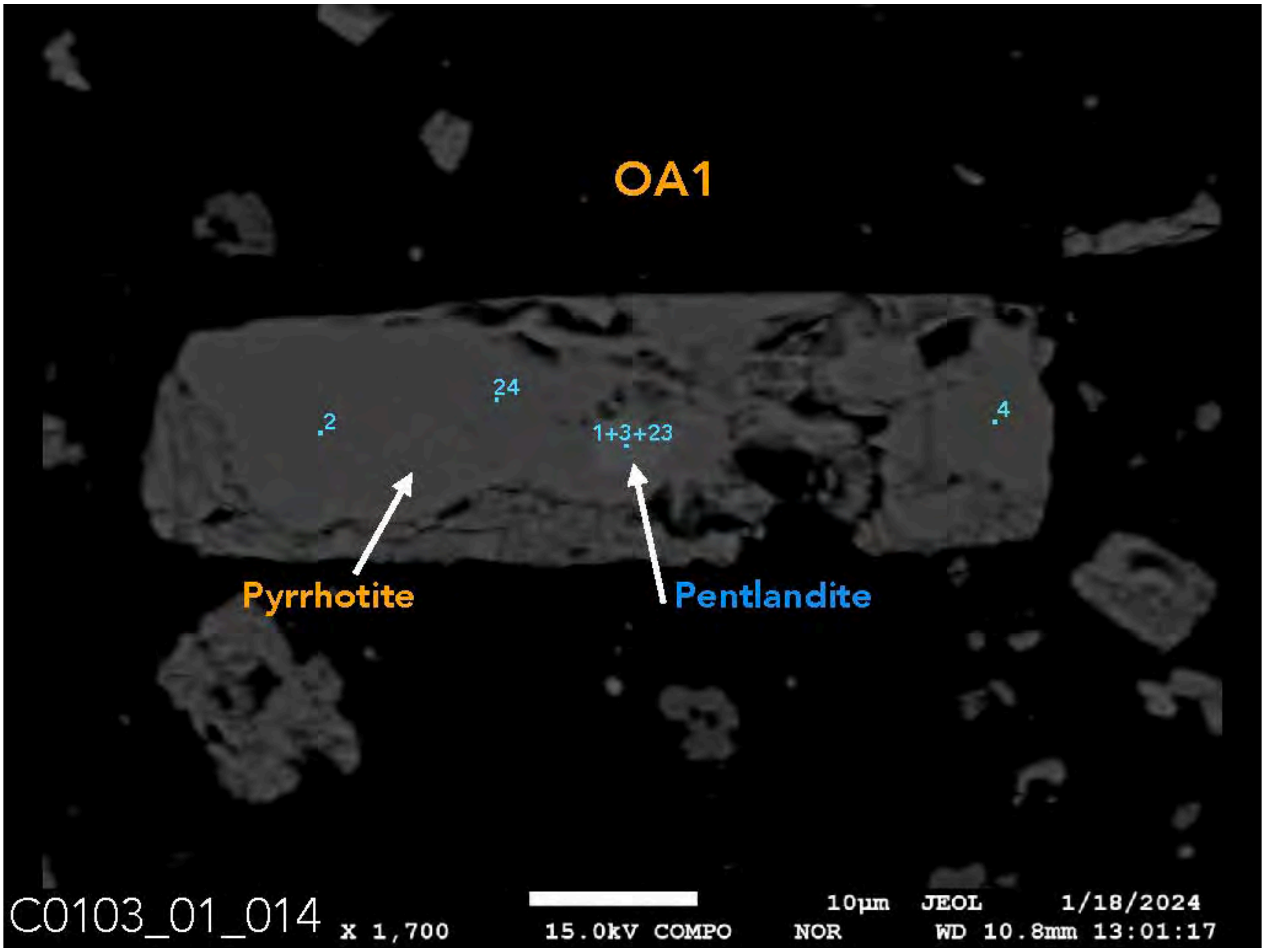

Supplementary Figure 133. Backscattered electron (BSE) image of OA1 in C0103-01, with spot analyses marked. OA = opaque assemblage.

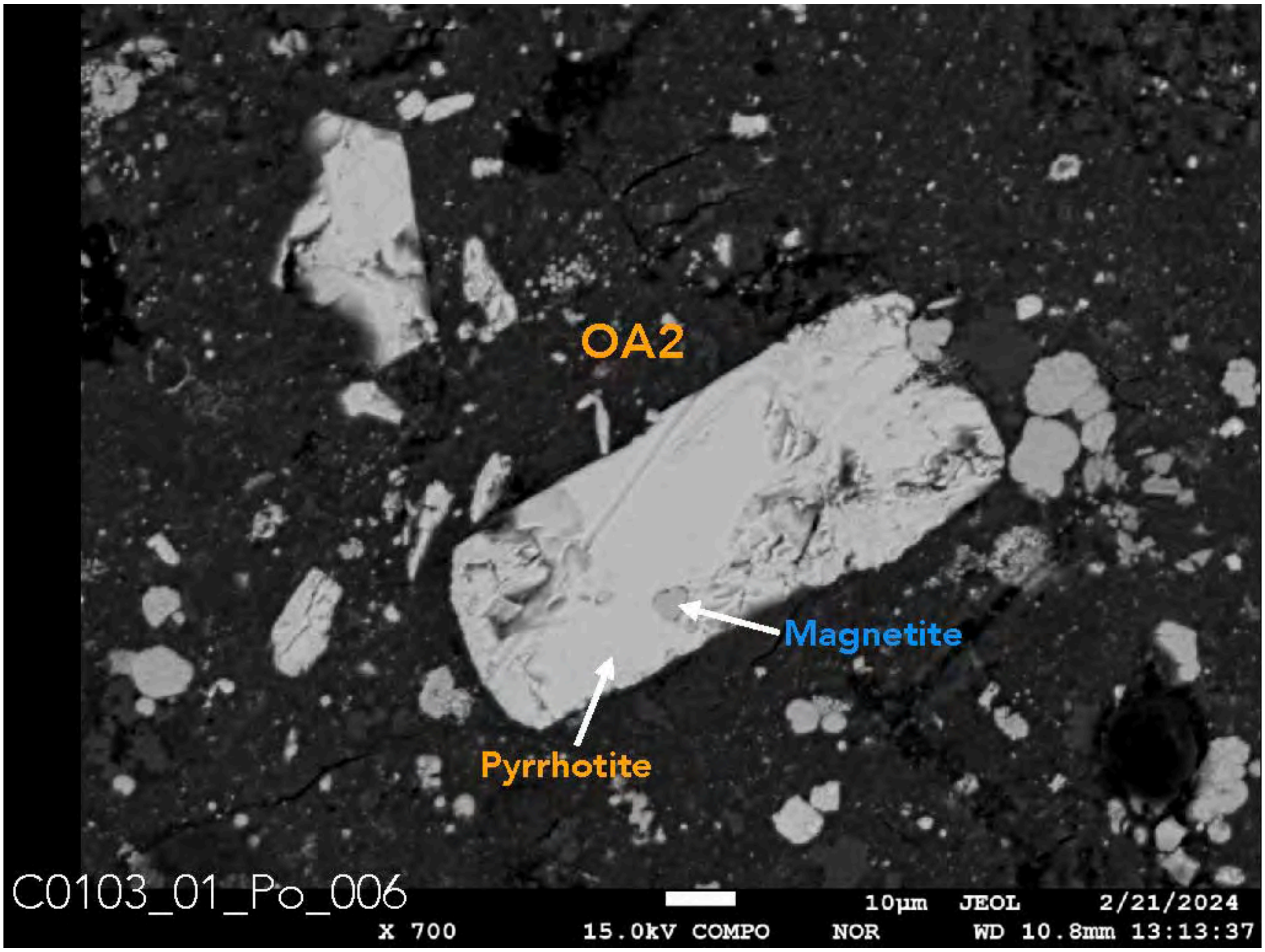

Supplementary Figure 134. Backscattered electron (BSE) image of OA2 in C0103-01. OA = opaque assemblage.

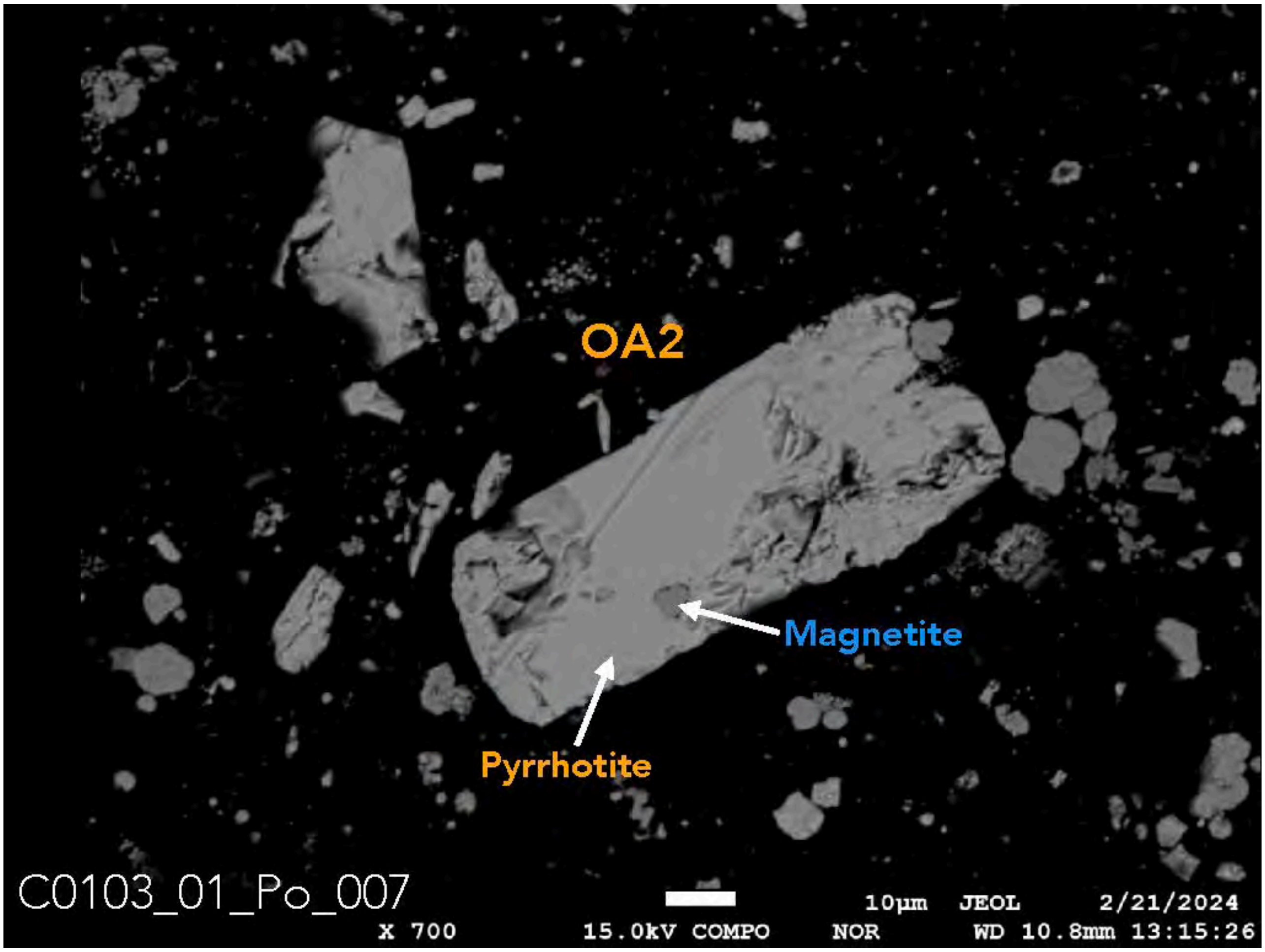

Supplementary Figure 135. Backscattered electron (BSE) image of OA2 in C0103-01. OA = opaque assemblage.

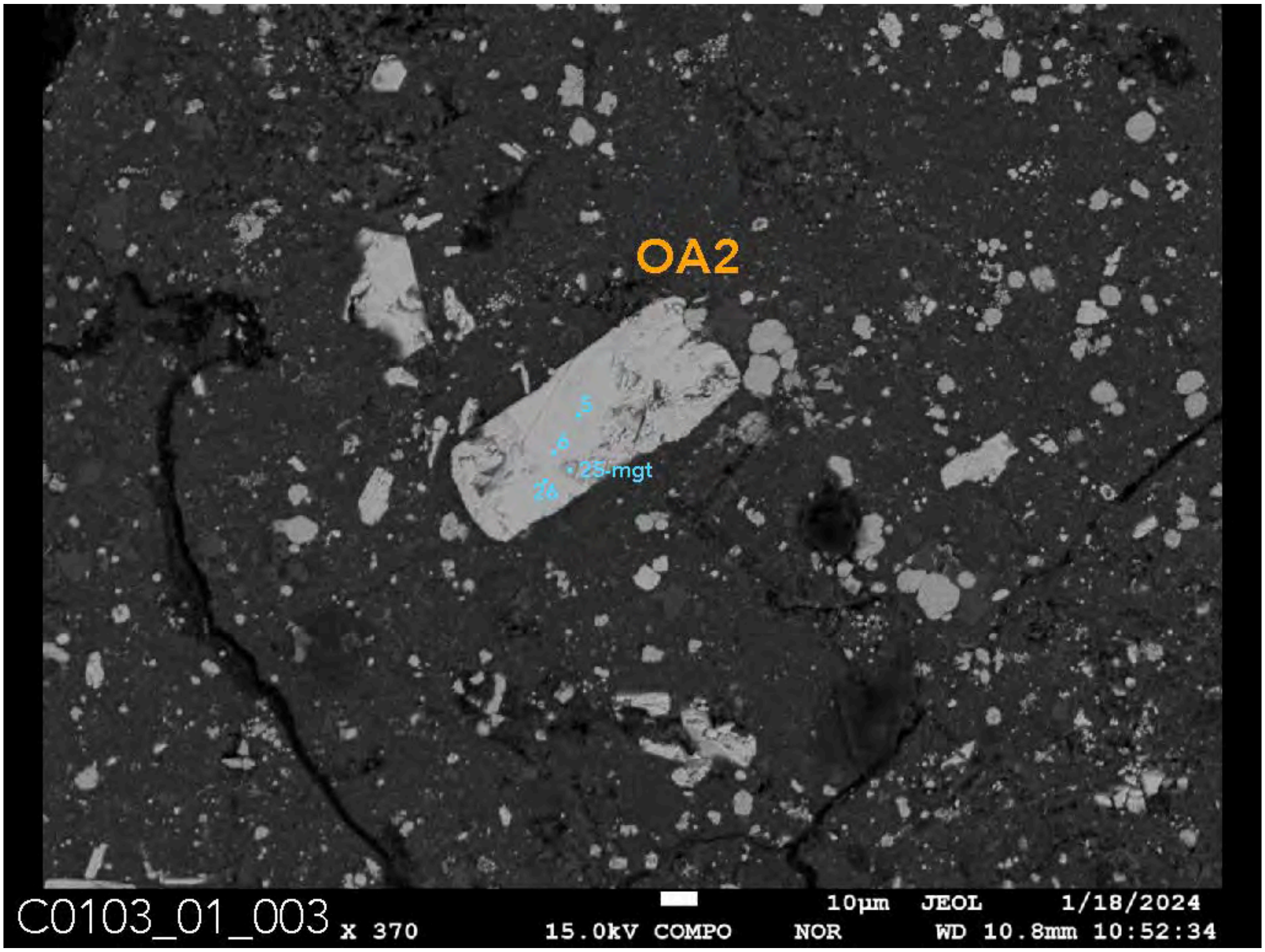

Supplementary Figure 136. Backscattered electron (BSE) image of OA2 in C0103-01, with spot analyses marked. OA = opaque assemblage and mgt = magnetite.

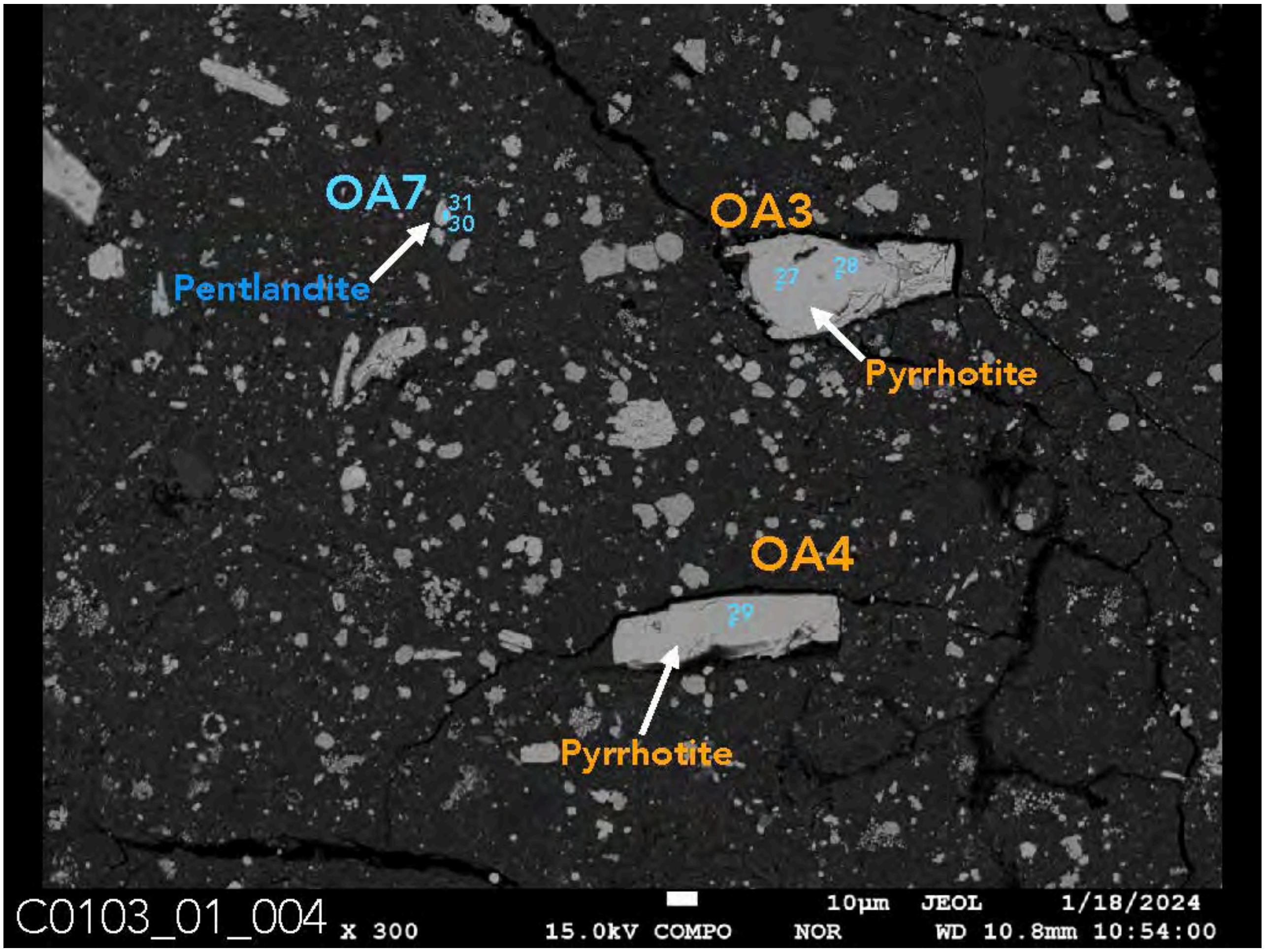

Supplementary Figure 137. Backscattered electron (BSE) image of OA3, OA4, and OA7 in C0103-01, with spot analyses marked. OA = opaque assemblage.

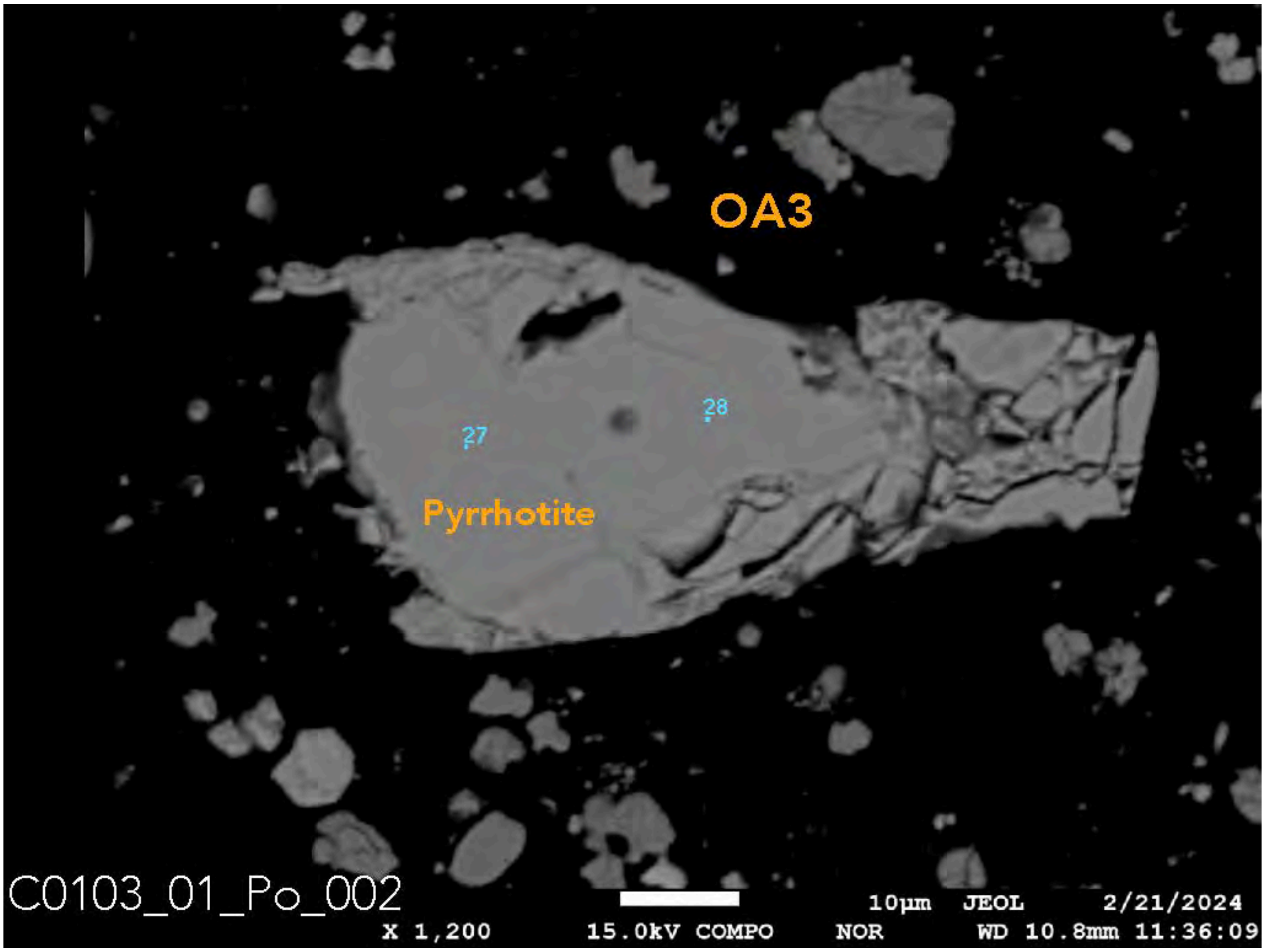

Supplementary Figure 138. Backscattered electron (BSE) image of OA3 in C0103-01, with spot analyses marked. OA = opaque assemblage.

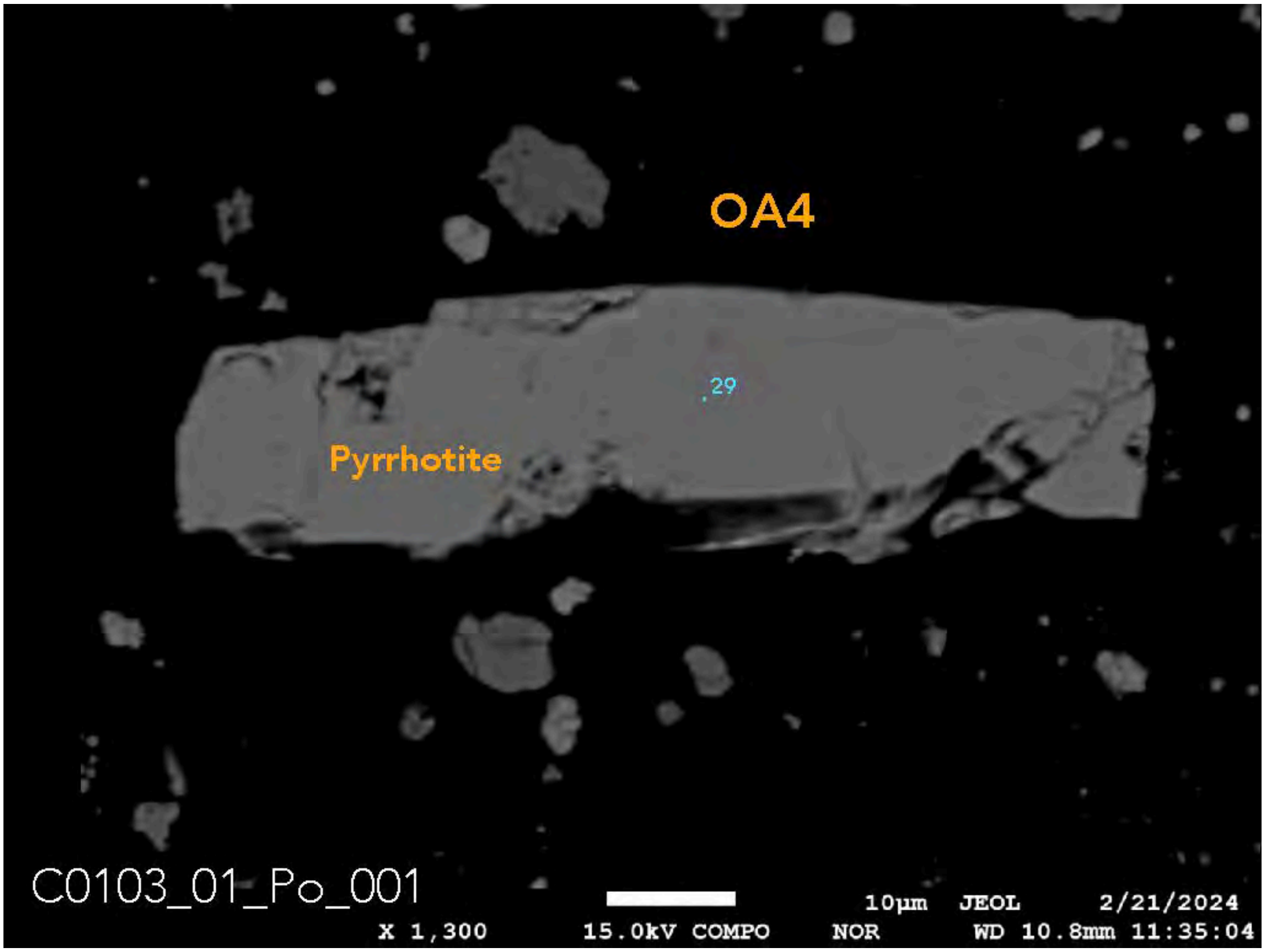

Supplementary Figure 139. Backscattered electron (BSE) image of OA4 in C0103-01, with spot analyses marked. OA = opaque assemblage.

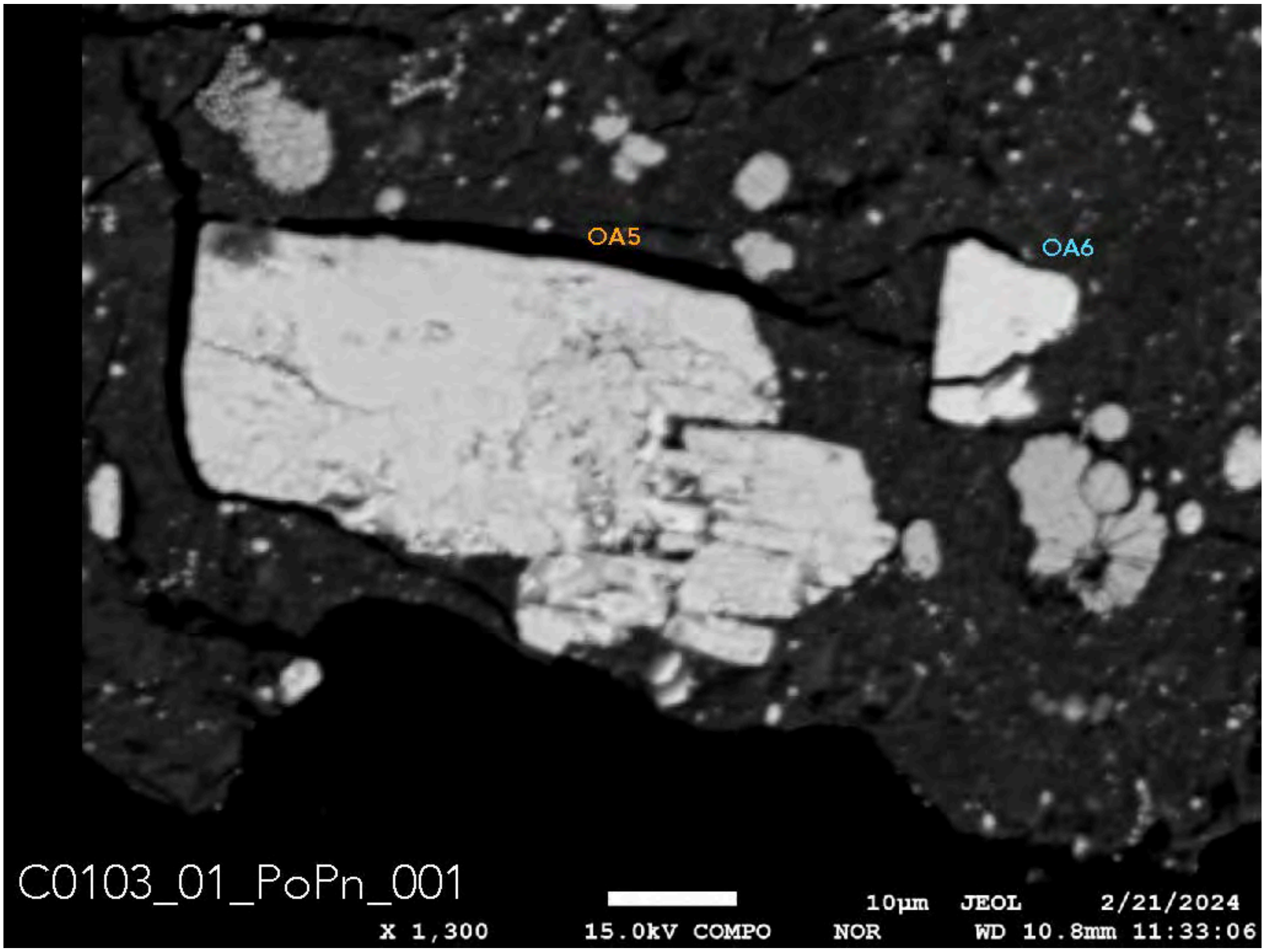

Supplementary Figure 140. Backscattered electron (BSE) image of OA5 and OA6 in C0103-01. OA = opaque assemblage.

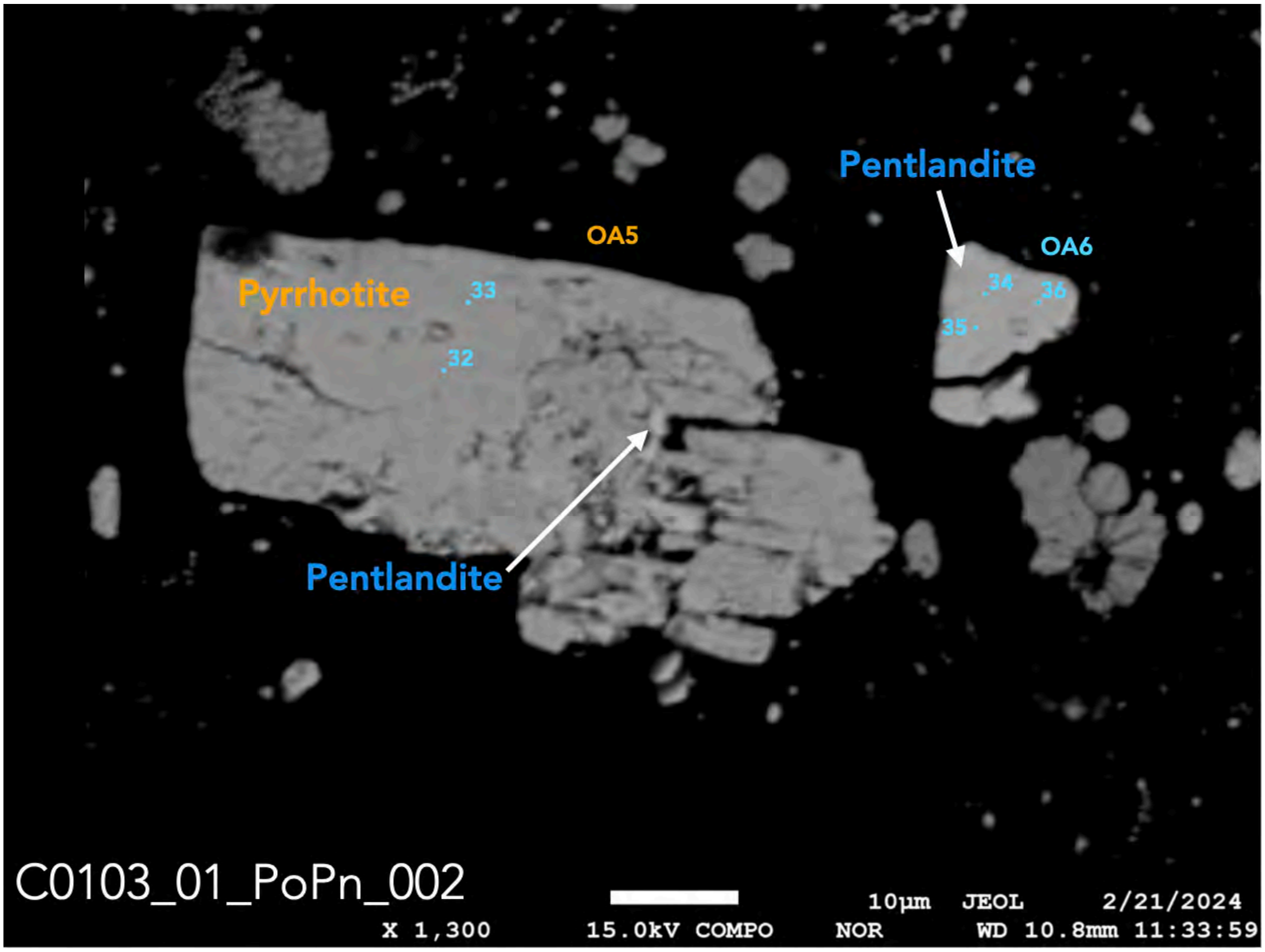

Supplementary Figure 141. Backscattered electron (BSE) image of OA5 and OA6 in C0103-01, with spot analyses marked. OA = opaque assemblage.

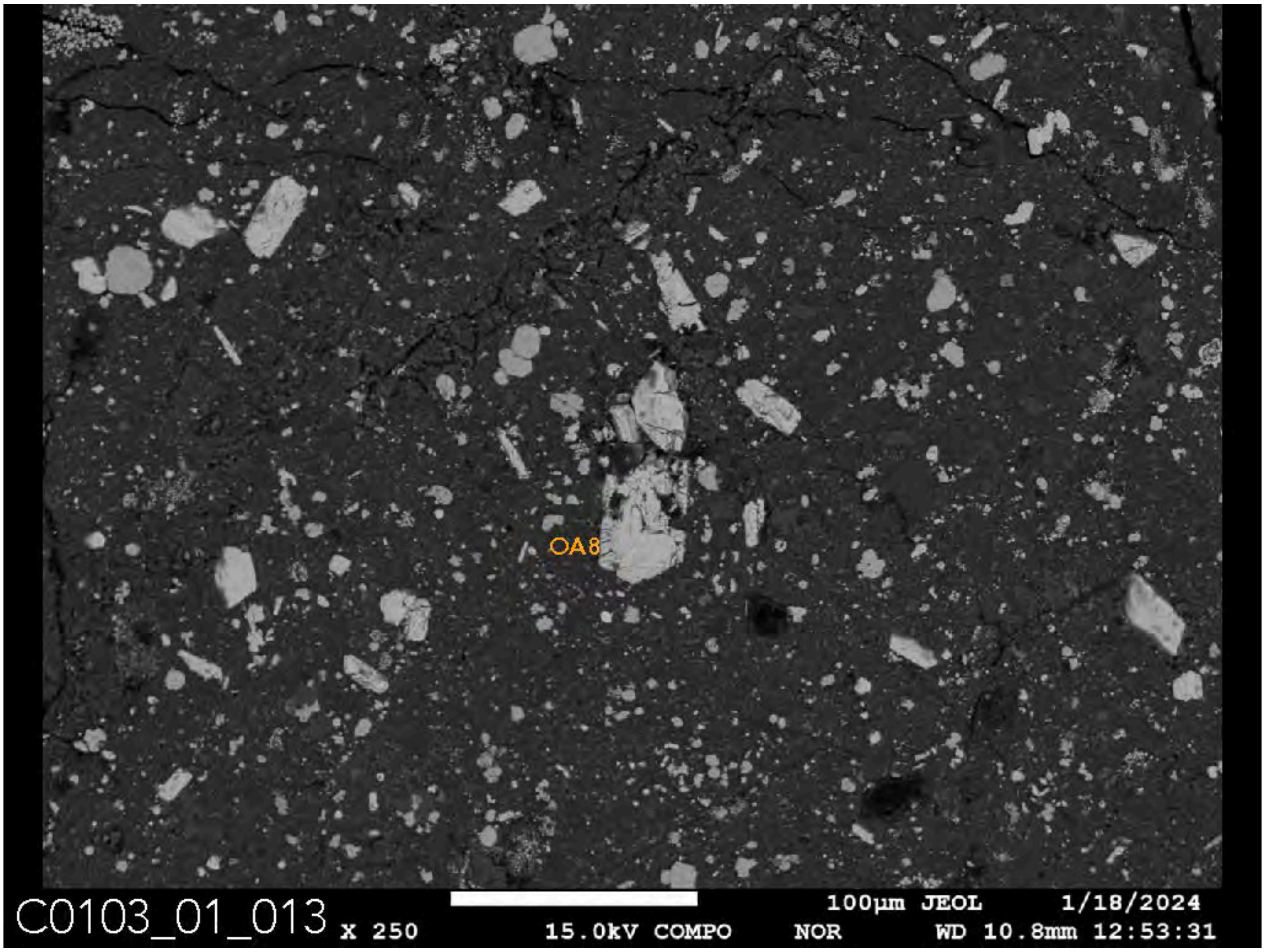

Supplementary Figure 142. Backscattered electron (BSE) image of OA8 in C0103-01. OA = opaque assemblage

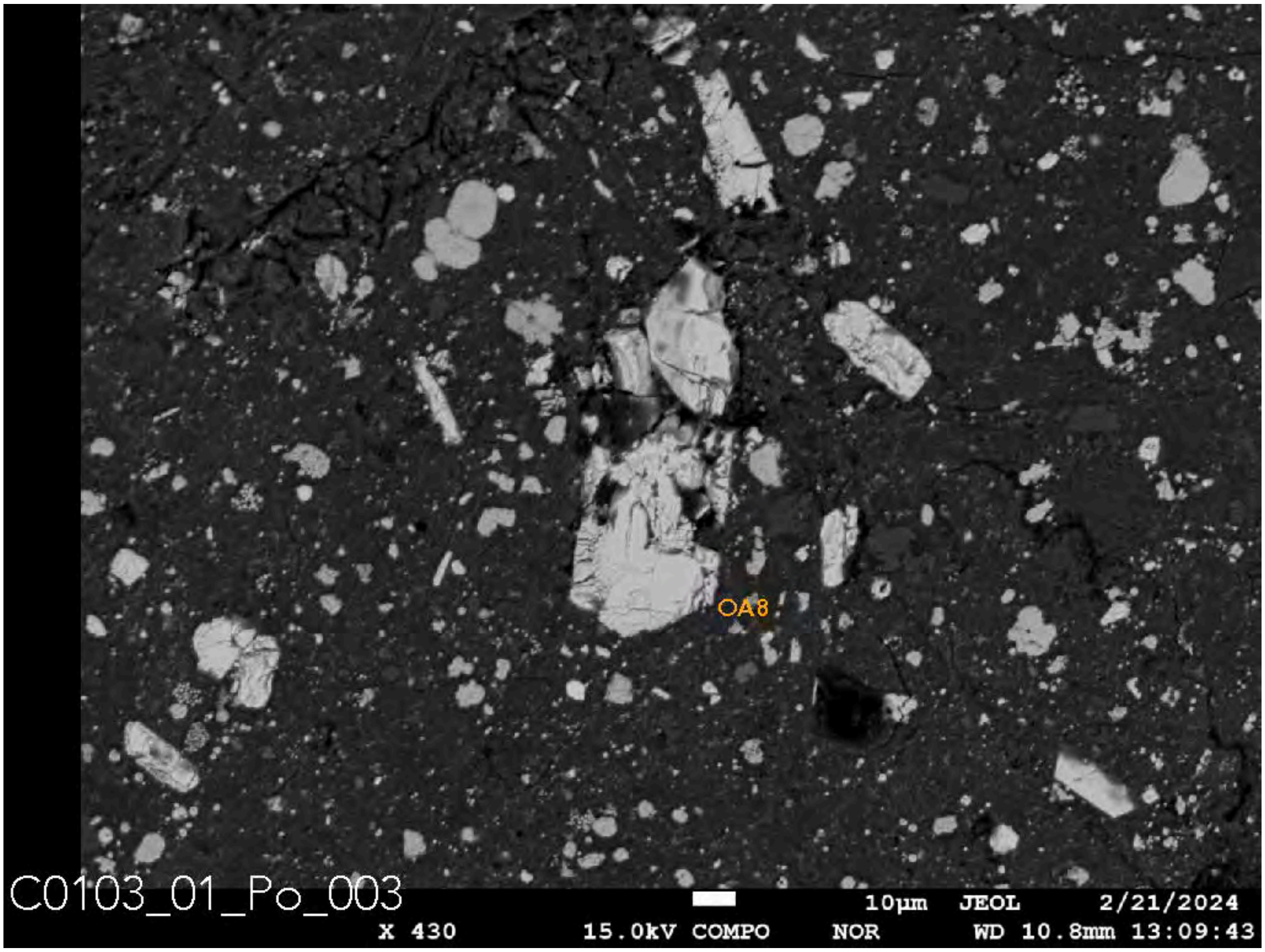

Supplementary Figure 143. Backscattered electron (BSE) image of OA8 in C0103-01. OA = opaque assemblage.

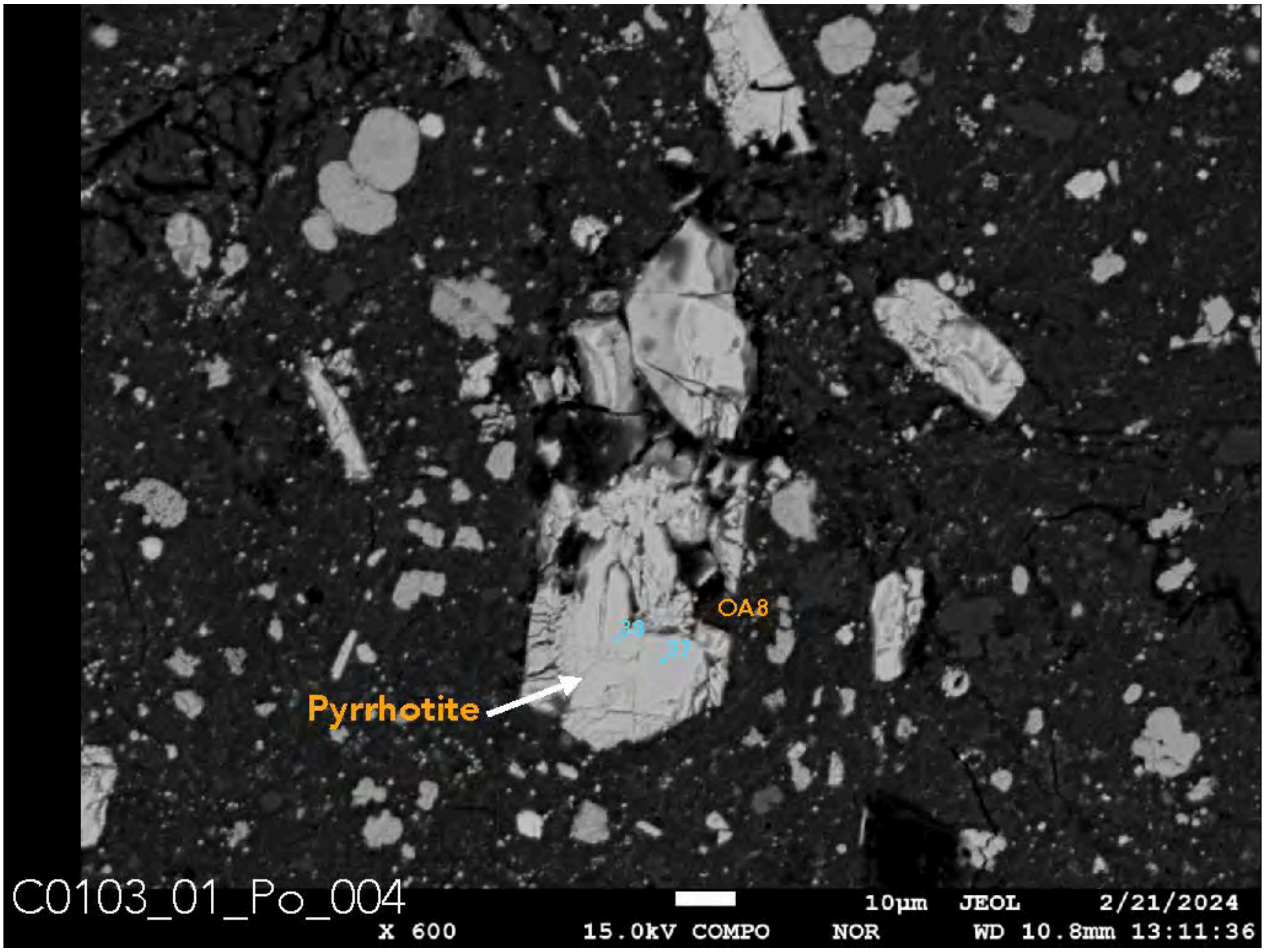

Supplementary Figure 144. Backscattered electron (BSE) image of OA8 in C0103-01, with spot analyses marked. OA = opaque assemblage.

Part III

Sulfide Ternaries for Ryugu Samples: A0016, A0094-01, and C0103-01.

Sulfide compositions from A0016, A0094-01, and C0103-01 are shown below on 500 °C , 400 °C, 230 °C, 100–135 °C, and 25 °C Ni+Co-S-Fe ternary phase diagrams to show which phase diagrams do not match the sulfide compositions and those that are consistent with the sulfide compositions (allowing sulfide equilibration temperatures to be determined).

For A0016, opaque assemblage 22 (OA22) was along the edge of the particle and was the only sulfide grain in A0016 that contained pyrrhotite-pentlandite. In case OA22 was from a lithology distinct from the dominate portion of the sample, the sulfide compositions of OA22 were plotted separately from the rest of the particle's sulfides below to investigate consistency with the equilibration temperature from the rest of the particle's sulfides. All sulfides in A0016 have the same equilibration temperature range of 230–400 °C, so are presented together in the manuscript.

Phase diagrams modified from:

500 °C: data from [1], adapted from [2]

400 °C: data from [3], adapted from [2]

230 °C: adapted from [4]

100–135 °C: adapted from [5]

25 °C: adapted from [6]

Note: The lines of the 25 °C diagram from [6] are so thick it is not possible to discern if the 25 °C diagram (using Adobe Photoshop, the tie lines shown here were drawn down the center of the lines from [6]) is a better fit than the 100–135 °C diagram for A0094-01 and C0103-01. Therefore, an equilibration range of 25–135 °C is concluded for these samples.

po = pyrrhotite, pn = pentlandite, py = pyrite, mss = monosulfide solid solution, hz = heazlewoodite (Ni_3S_2), vs = vaesite (NiS_2), viol = violarite ($FeNi_2S_4$), a = kamacite, and y = taenite.

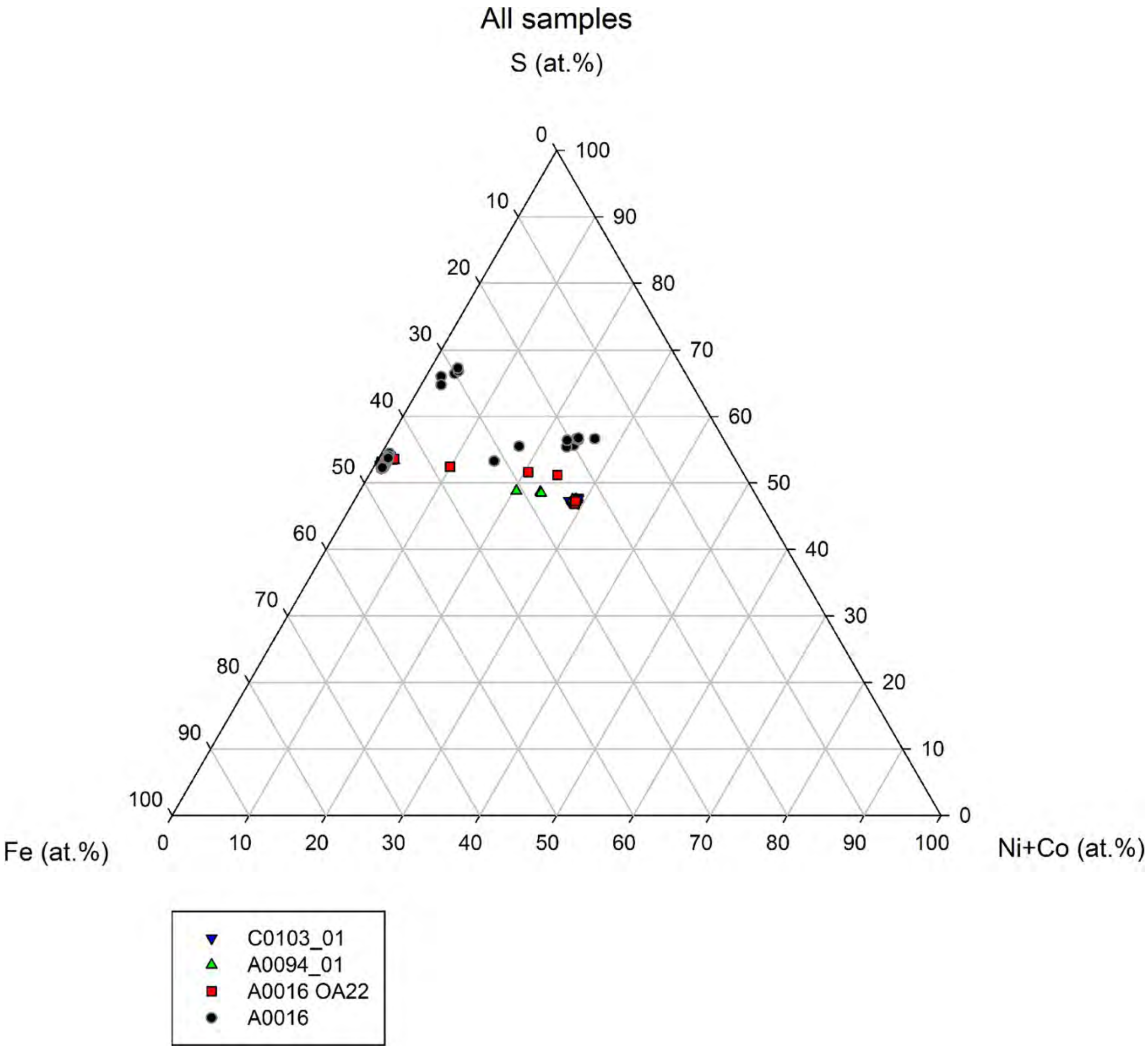

Supplementary Figure 145. In situ sulfide compositional data obtained via electron probe microanalyzer for all samples studied here (A0016, A0094-01, and C0103-01), superimposed on at.% Fe-Ni+Co-S ternary diagram. Pentlandite bearing grain from A0016, OA22, shown separately. OA = opaque assemblage.

A0016

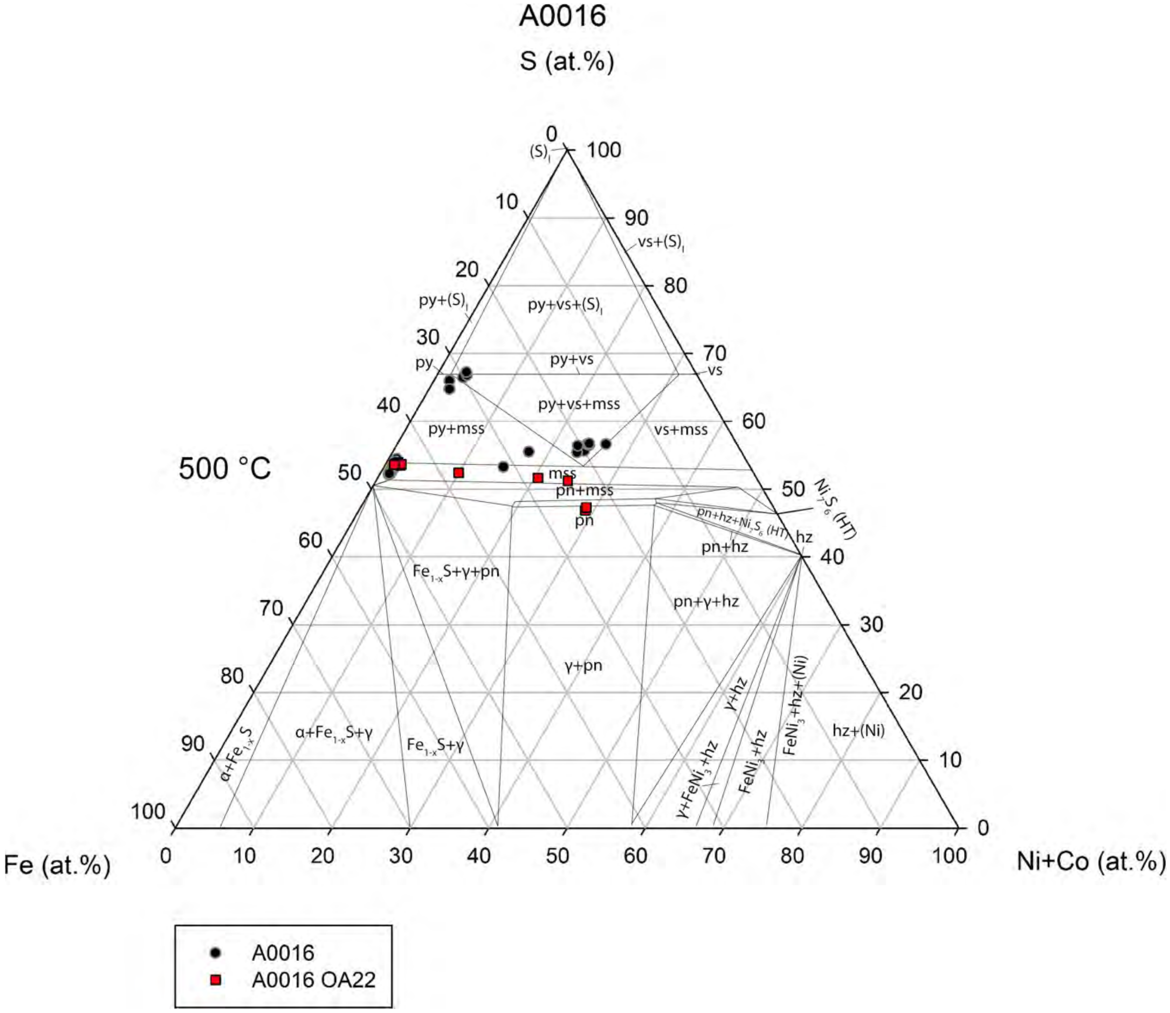

Supplementary Figure 146. In situ sulfide compositional data obtained via electron probe microanalyzer for A0016, superimposed on at.% Fe-Ni+Co-S phase diagram at 500 °C (phase diagram adapted from [1,2]). Pentlandite bearing grain from A0016, OA22, shown separately. OA = opaque assemblage. Where po = pyrrhotite, pn = pentlandite, py = pyrite, mss = monosulfide solid solution, hz = heazlewoodite (Ni_3S_2), vs = vaesite (NiS_2), viol = violarite ($FeNi_2S_4$), a = kamacite, and y = taenite.

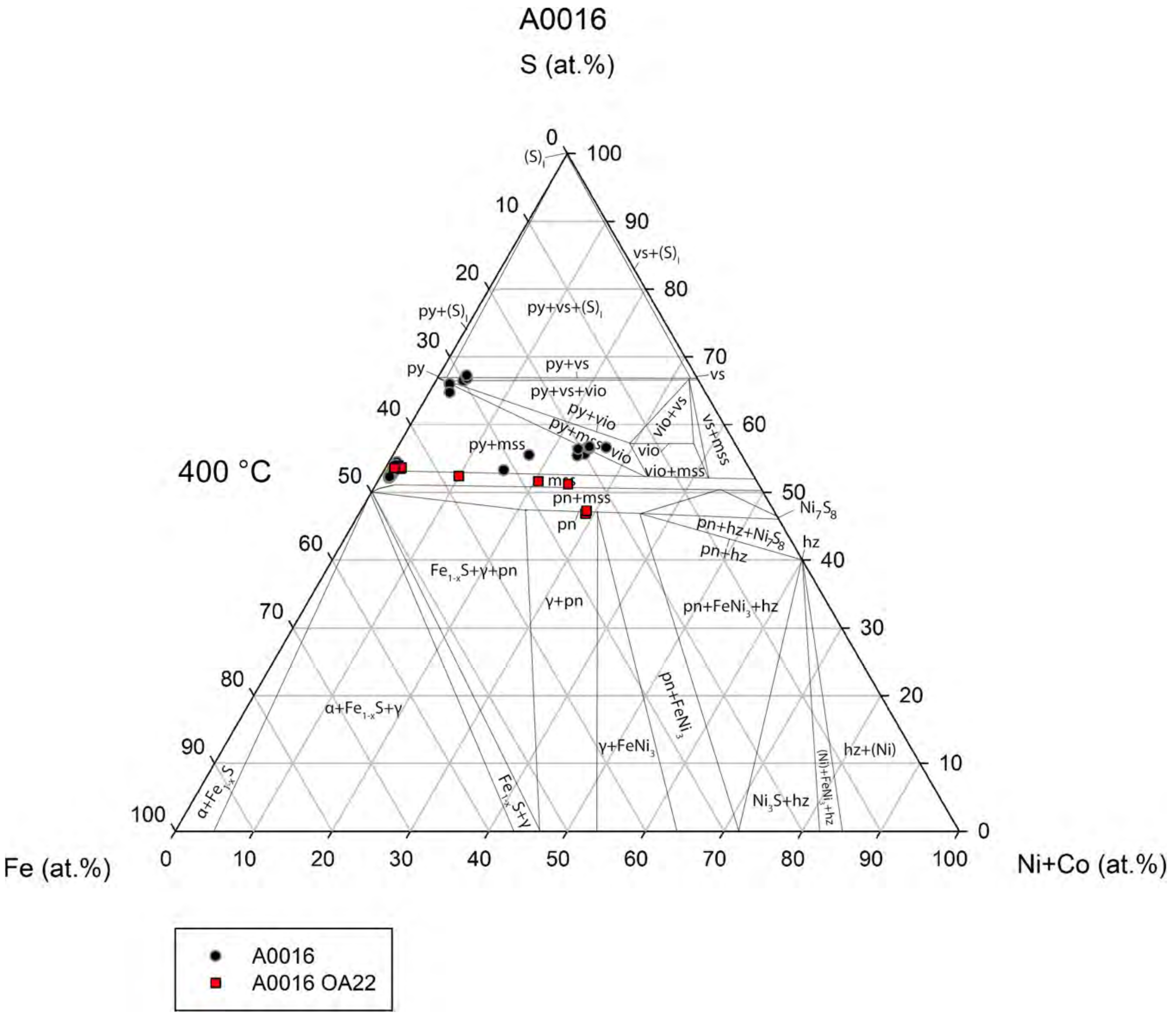

Supplementary Figure 147. In situ sulfide compositional data obtained via electron probe microanalyzer for A0016, superimposed on at.% Fe-Ni+Co-S phase diagram at 400 °C (phase diagram adapted from [2,3]). Pentlandite bearing grain from A0016, OA22, shown separately. OA = opaque assemblage. Where po = pyrrhotite, pn = pentlandite, py = pyrite, mss = monosulfide solid solution, hz = heazlewoodite (Ni_3S_2), vs = vaesite (NiS_2), viol = violarite ($FeNi_2S_4$), a = kamacite, and y = taenite.

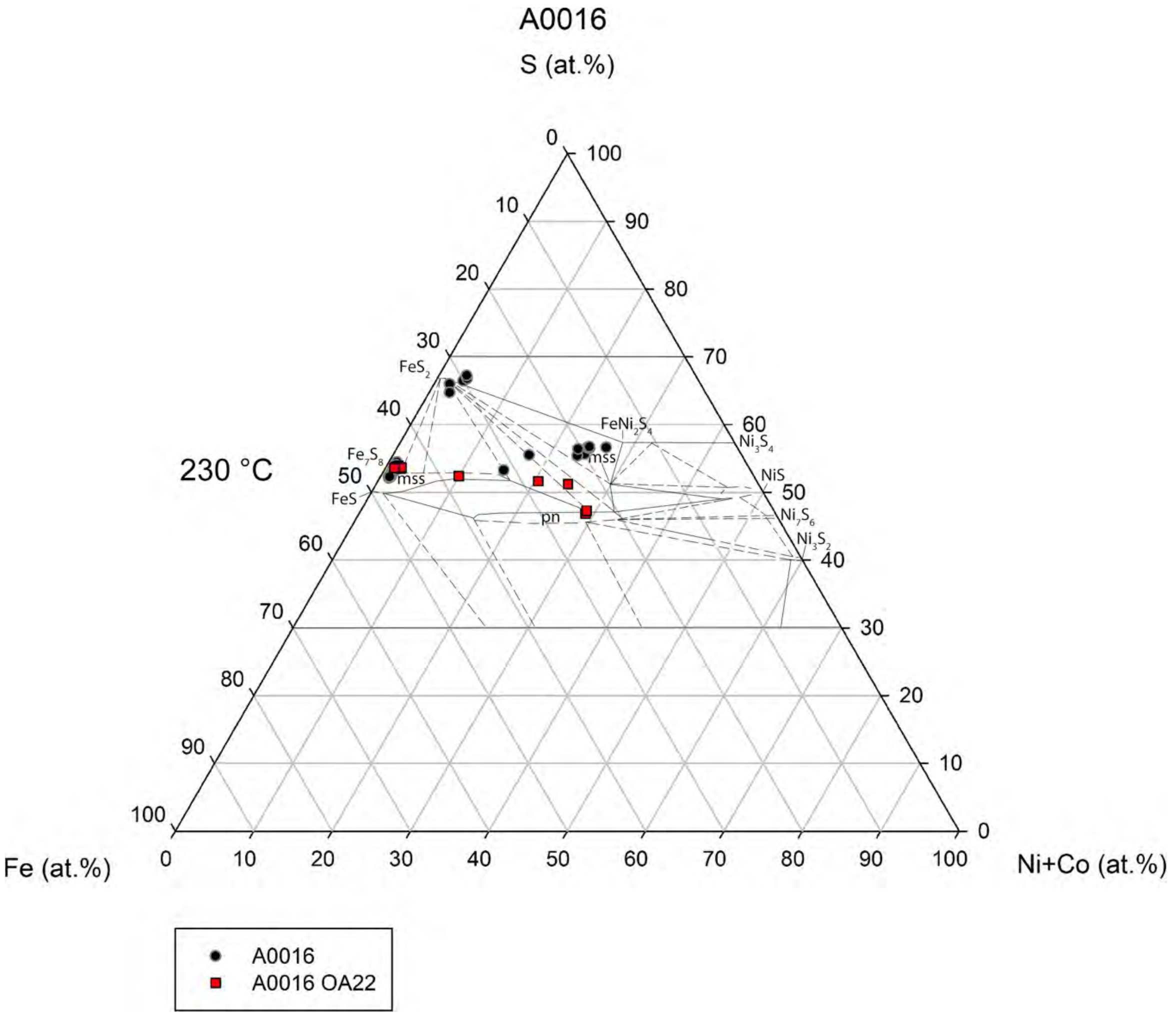

Supplementary Figure 148. In situ sulfide compositional data obtained via electron probe microanalyzer for A0016, superimposed on at.% Fe-Ni+Co-S phase diagram at 230 °C (phase diagram adapted from [4]). Pentlandite bearing grain from A0016, OA22, shown separately. OA = opaque assemblage. Where po = pyrrhotite, pn = pentlandite, py = pyrite, mss = monosulfide solid solution, hz = heazlewoodite (Ni_3S_2), vs = vaesite (NiS_2), viol = violarite ($FeNi_2S_4$), a = kamacite, and y = taenite.

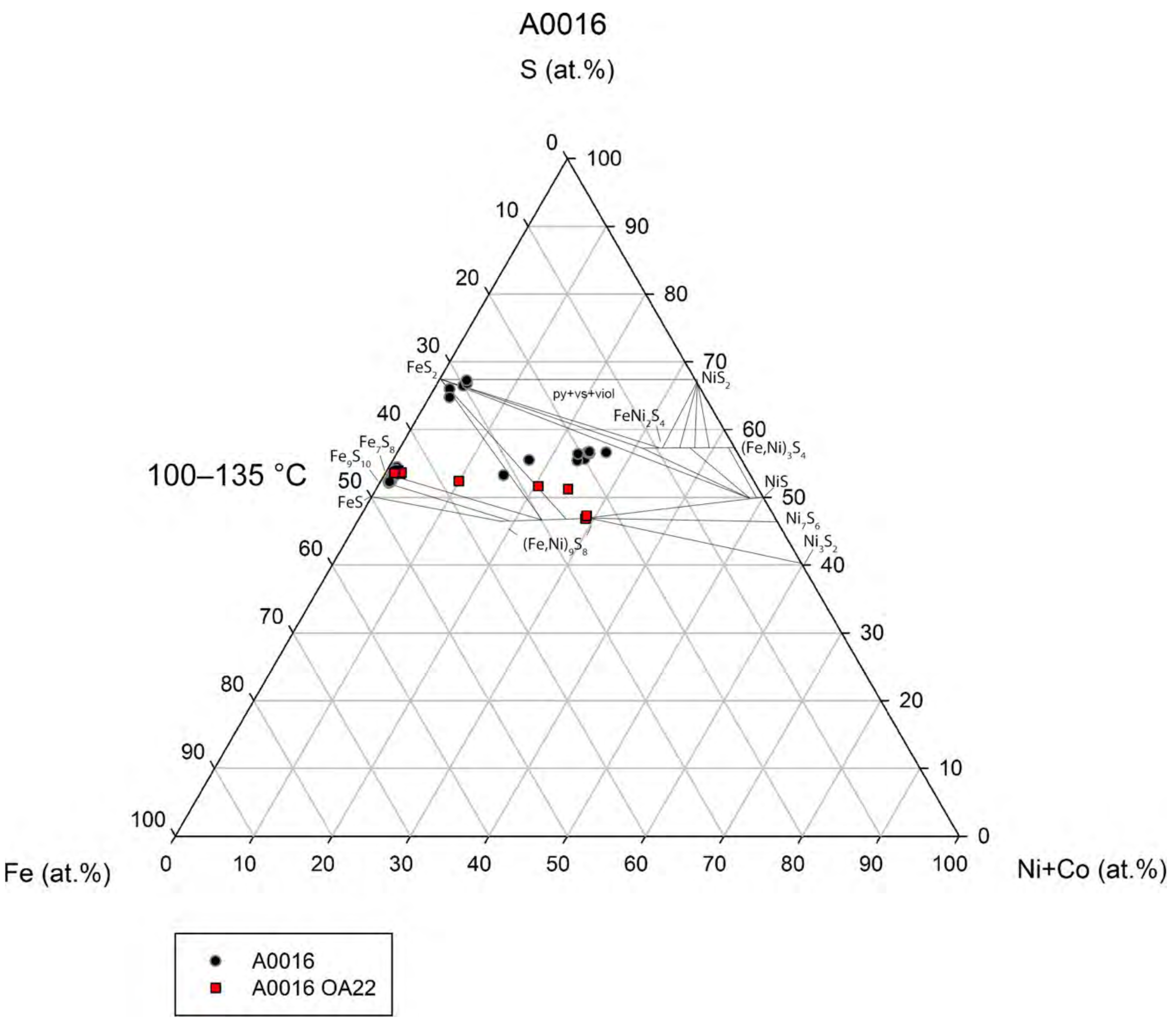

Supplementary Figure 149. In situ sulfide compositional data obtained via electron probe microanalyzer for A0016, superimposed on at.% Fe-Ni+Co-S phase diagram at 100–135 °C (phase diagram adapted from [5]). Pentlandite bearing grain from A0016, OA22, shown separately. OA = opaque assemblage. Where po = pyrrhotite, pn = pentlandite, py = pyrite, mss = monosulfide solid solution, hz = heazlewoodite (Ni_3S_2), vs = vaesite (NiS_2), viol = violarite ($FeNi_2S_4$), a = kamacite, and y = taenite.

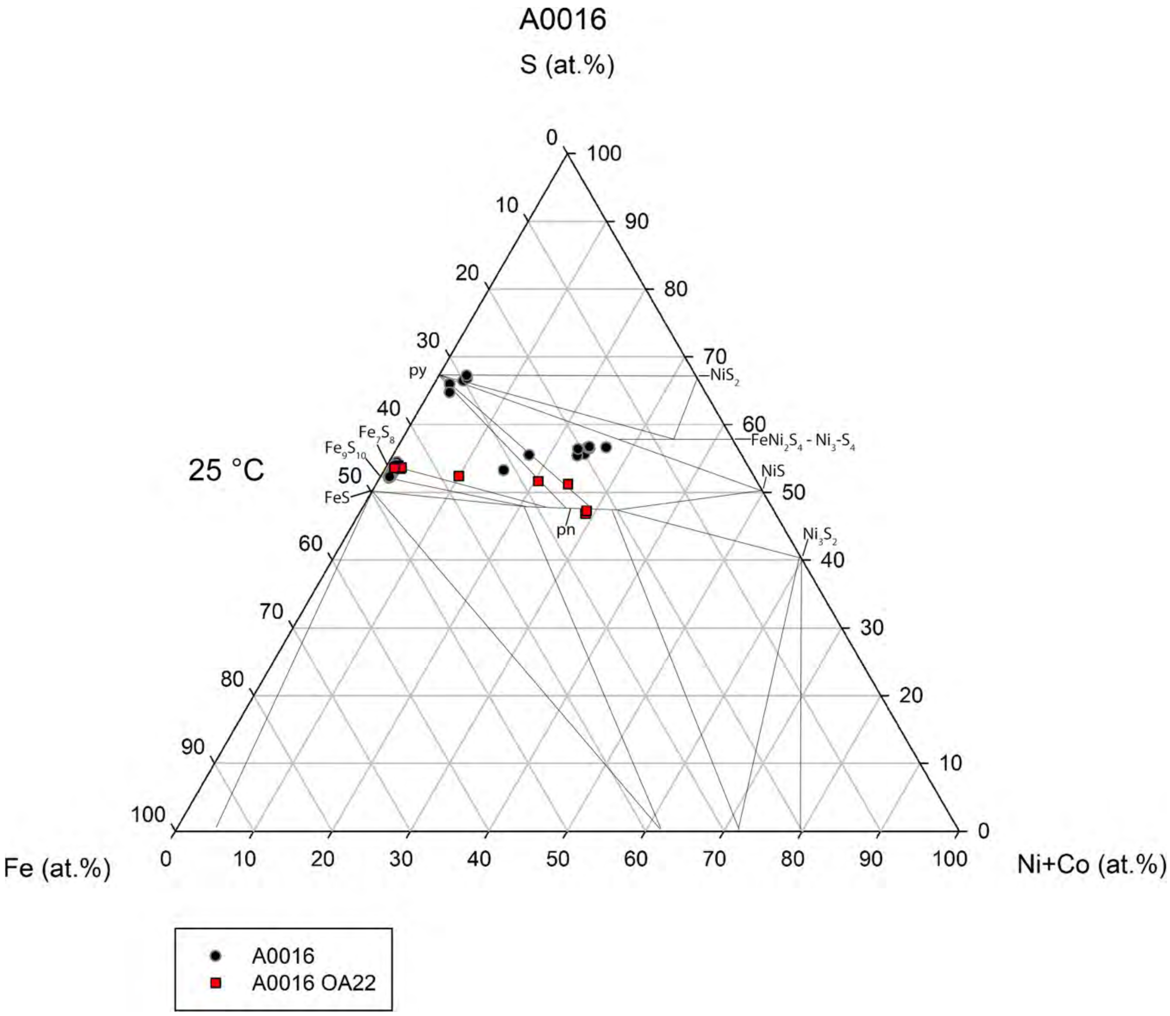

Supplementary Figure 150. In situ sulfide compositional data obtained via electron probe microanalyzer for A0016, superimposed on at.% Fe-Ni+Co-S phase diagram at 25 °C (phase diagram adapted from [6]). Pentlandite bearing grain from A0016, OA22, shown separately. OA = opaque assemblage. Where po = pyrrhotite, pn = pentlandite, py = pyrite, mss = monosulfide solid solution, hz = heazlewoodite (Ni_3S_2), vs = vaesite (NiS_2), viol = violarite ($FeNi_2S_4$), a = kamacite, and y = taenite.

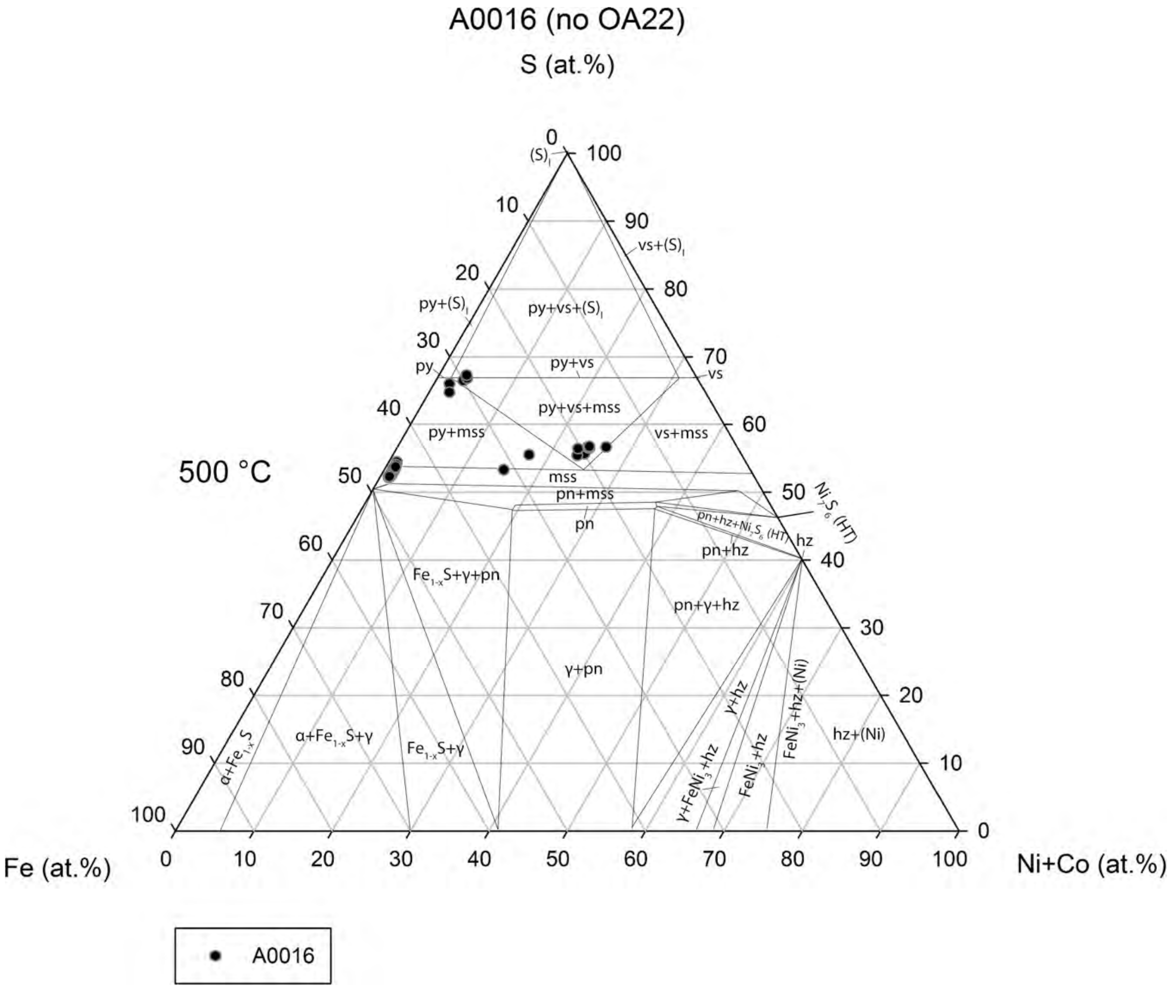

Supplementary Figure 151. In situ sulfide compositional data obtained via electron probe microanalyzer for A0016, superimposed on at.% Fe-Ni+Co-S phase diagram at 500 °C (phase diagram adapted from [1,2]). Pentlandite bearing grain from A0016, OA22, not shown. OA = opaque assemblage. Where po = pyrrhotite, pn = pentlandite, py = pyrite, mss = monosulfide solid solution, hz = heazlewoodite (Ni_3S_2), vs = vaesite (NiS_2), viol = violarite ($FeNi_2S_4$), a = kamacite, and y = taenite.

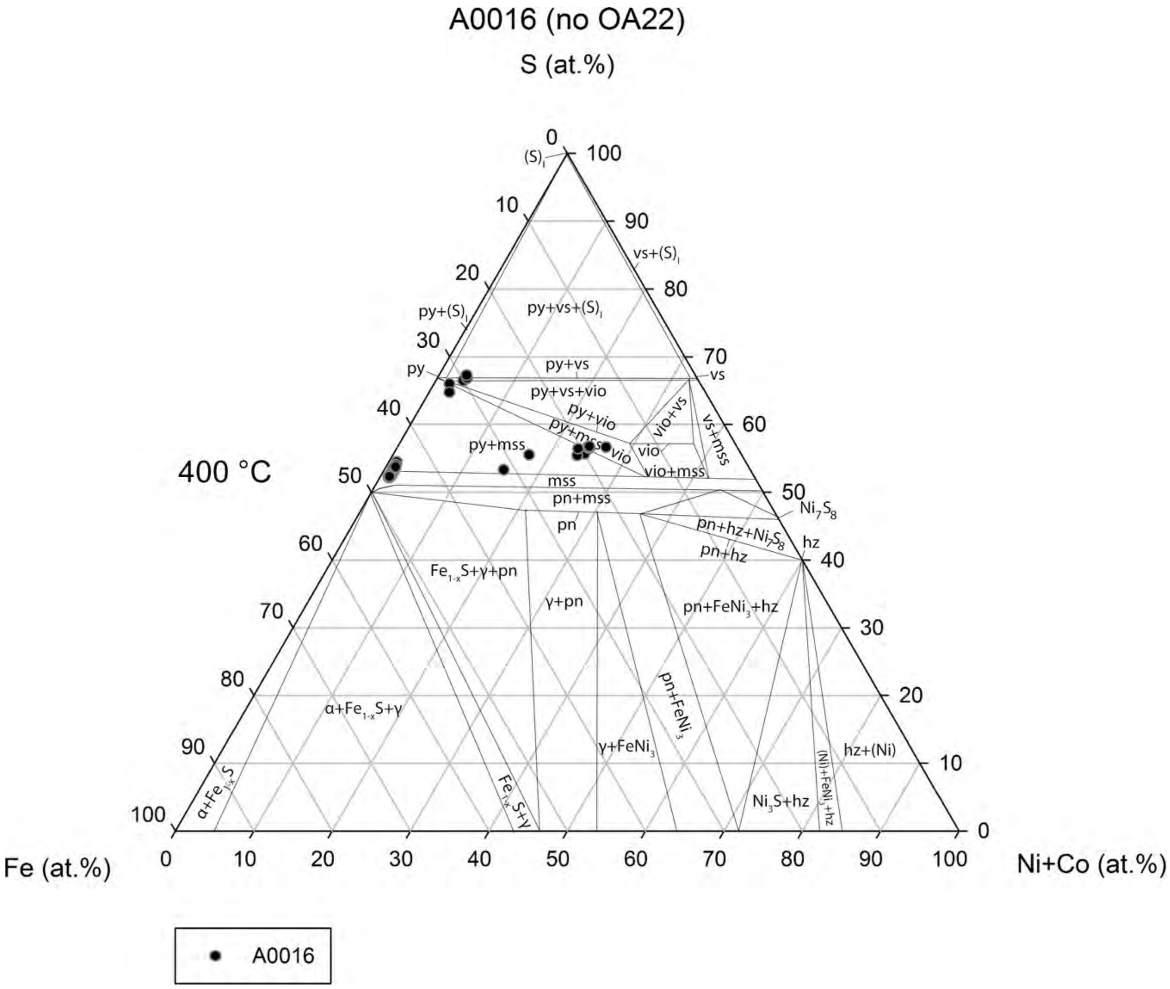

Supplementary Figure 152. In situ sulfide compositional data obtained via electron probe microanalyzer for A0016, superimposed on at.% Fe-Ni+Co-S phase diagram at 400 °C (phase diagram adapted from [2,3]). Pentlandite bearing grain from A0016, OA22, not shown. OA = opaque assemblage. Where po = pyrrhotite, pn = pentlandite, py = pyrite, mss = monosulfide solid solution, hz = heazlewoodite (Ni_3S_2), vs = vaesite (NiS_2), viol = violarite ($FeNi_2S_4$), a = kamacite, and y = taenite.

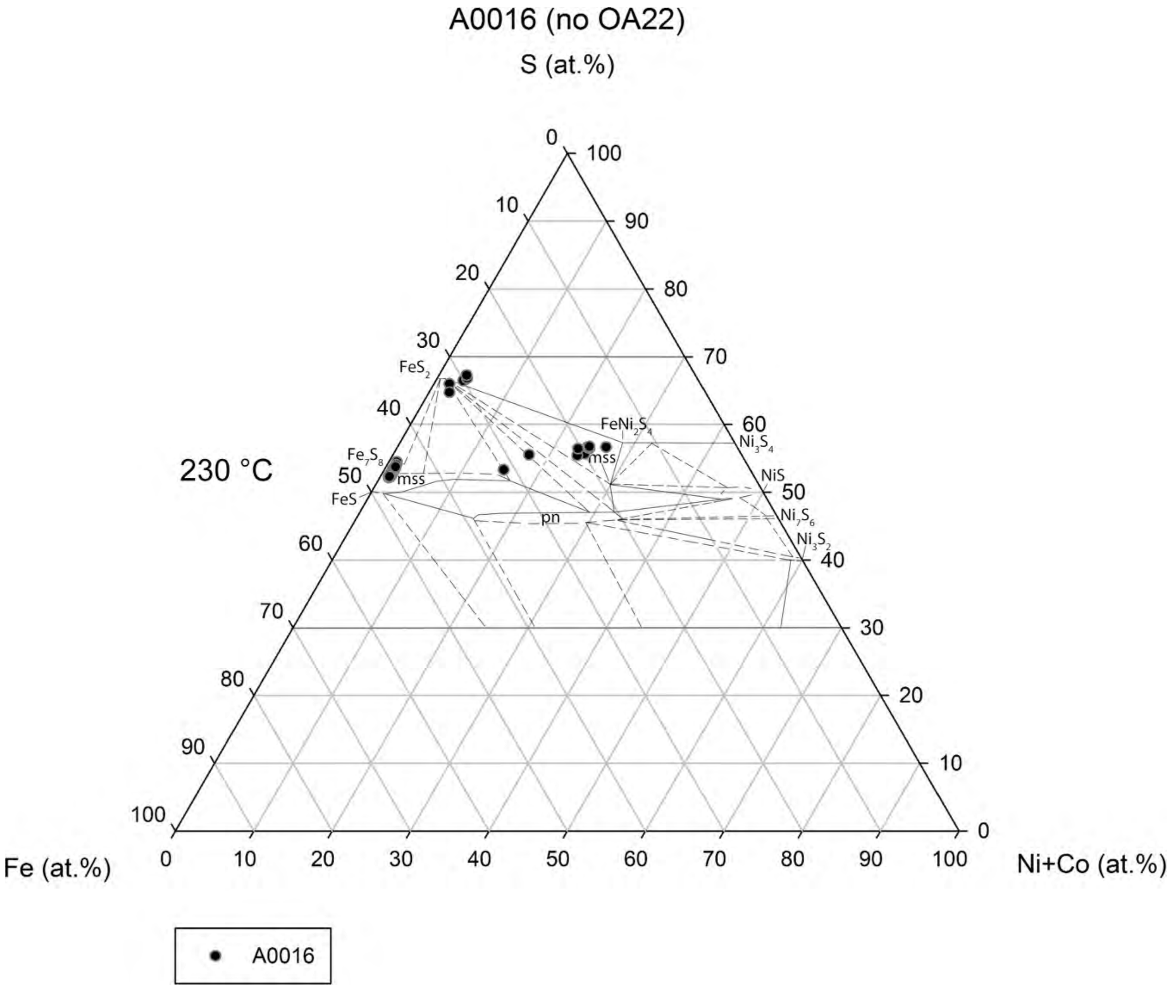

Supplementary Figure 153. In situ sulfide compositional data obtained via electron probe microanalyzer for A0016, superimposed on at.% Fe-Ni+Co-S phase diagram at 230 °C (phase diagram adapted from [4]). Pentlandite bearing grain from A0016, OA22, not shown. OA = opaque assemblage. Where po = pyrrhotite, pn = pentlandite, py = pyrite, mss = monosulfide solid solution, hz = heazlewoodite (Ni_3S_2), vs = vaesite (NiS_2), viol = violarite ($FeNi_2S_4$), a = kamacite, and y = taenite.

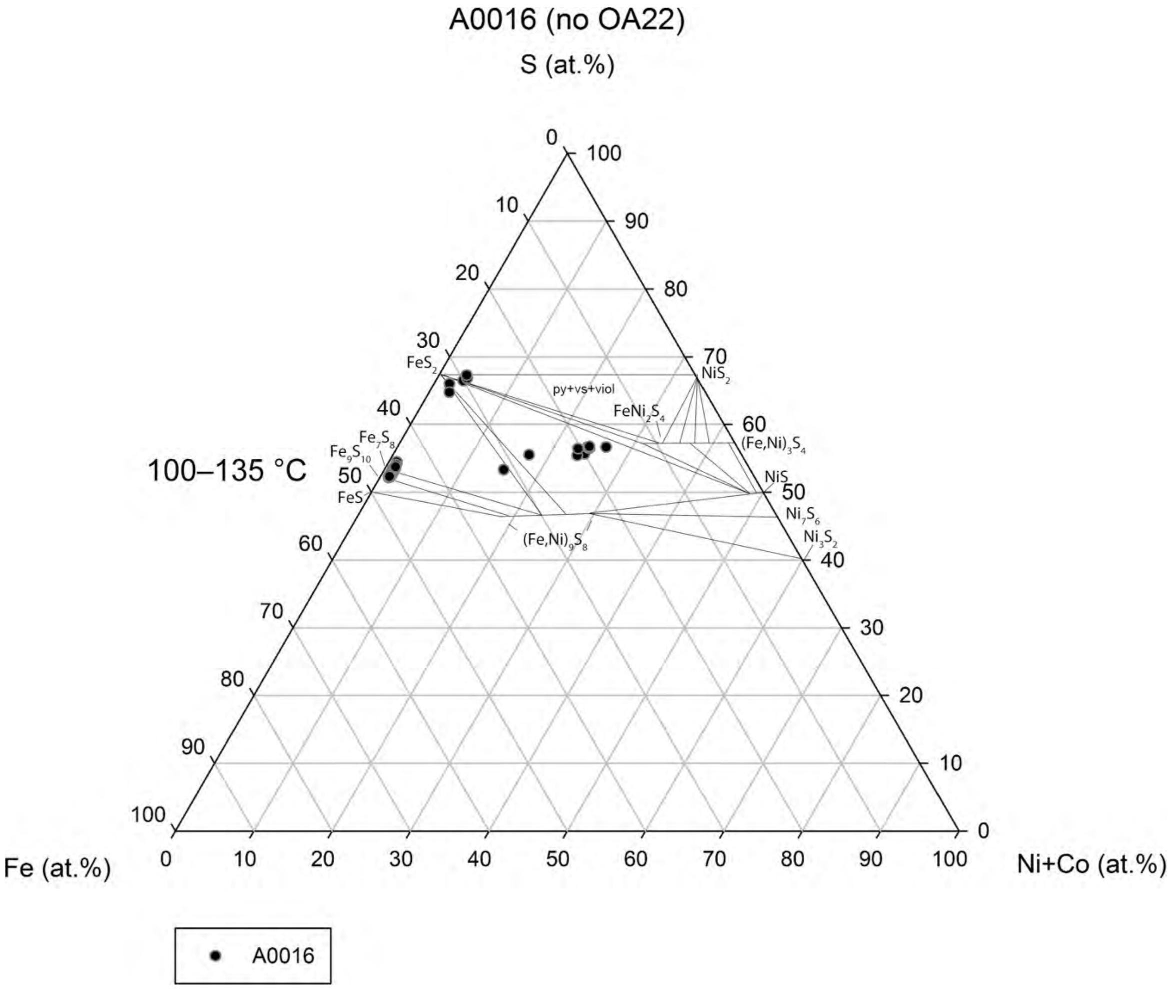

Supplementary Figure 154. In situ sulfide compositional data obtained via electron probe microanalyzer for A0016, superimposed on at.% Fe-Ni+Co-S phase diagram at 100–135 °C (phase diagram adapted from [5]). Pentlandite bearing grain from A0016, OA22, not shown. OA = opaque assemblage. Where po = pyrrhotite, pn = pentlandite, py = pyrite, mss = monosulfide solid solution, hz = heazlewoodite (Ni_3S_2), vs = vaesite (NiS_2), viol = violarite ($FeNi_2S_4$), a = kamacite, and y = taenite.

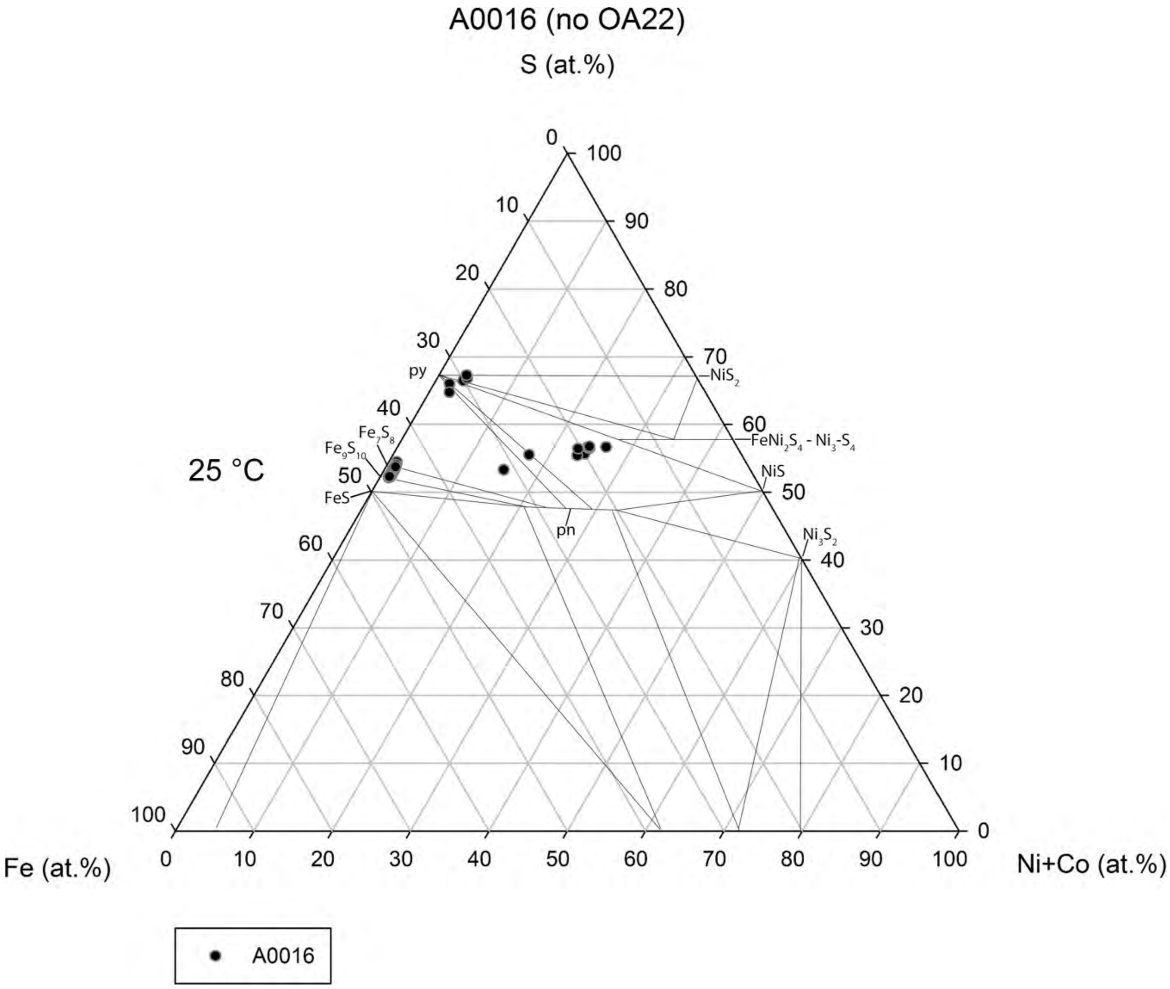

Supplementary Figure 155. In situ sulfide compositional data obtained via electron probe microanalyzer for A0016, superimposed on at.% Fe-Ni+Co-S phase diagram at 25 °C (phase diagram adapted from [6]). Pentlandite bearing grain from A0016, OA22, not shown. OA = opaque assemblage. Where po = pyrrhotite, pn = pentlandite, py = pyrite, mss = monosulfide solid solution, hz = heazlewoodite (Ni_3S_2), vs = vaesite (NiS_2), viol = violarite ($FeNi_2S_4$), a = kamacite, and y = taenite.

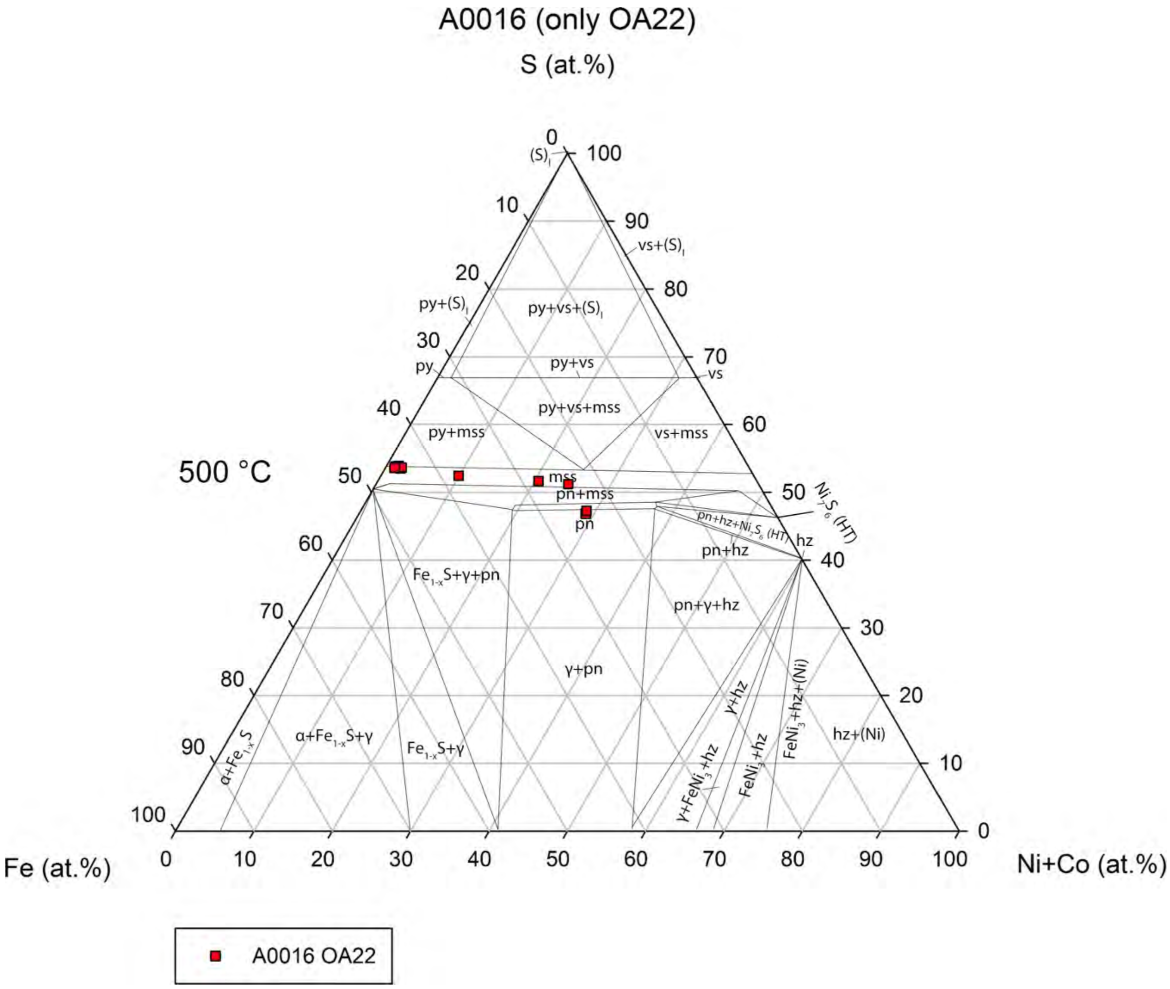

Supplementary Figure 156. In situ sulfide compositional data obtained via electron probe microanalyzer for A0016 OA22, superimposed on at.% Fe-Ni+Co-S phase diagram at 500 °C (phase diagram adapted from [1,2]). OA = opaque assemblage. Where po = pyrrhotite, pn = pentlandite, py = pyrite, mss = monosulfide solid solution, hz = heazlewoodite (Ni_3S_2), vs = vaesite (NiS_2), viol = violarite ($FeNi_2S_4$), a = kamacite, and y = taenite.

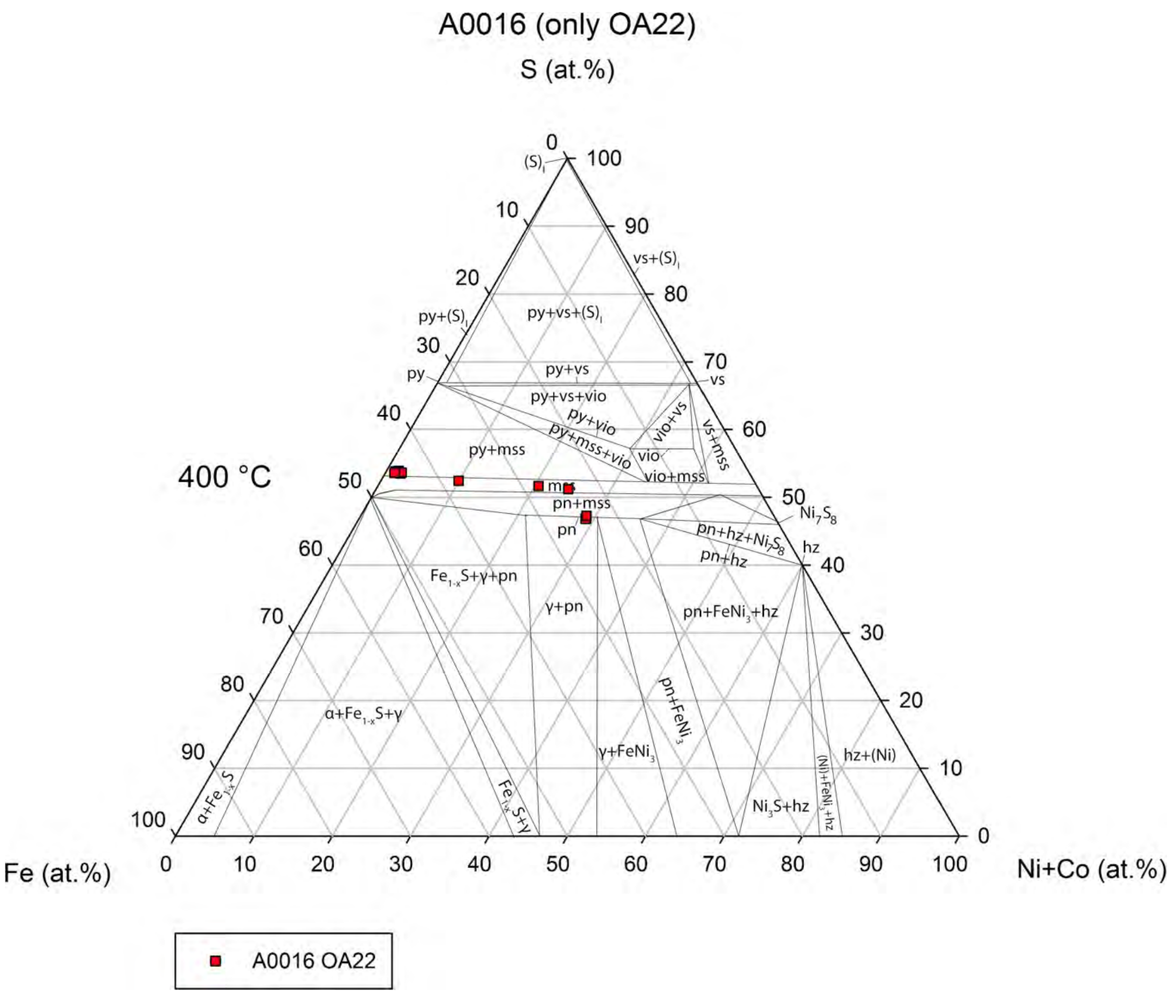

Supplementary Figure 157. In situ sulfide compositional data obtained via electron probe microanalyzer for A0016 OA22, superimposed on at.% Fe-Ni+Co-S phase diagram at 400 °C (phase diagram adapted from [2,3]). OA = opaque assemblage. Where po = pyrrhotite, pn = pentlandite, py = pyrite, mss = monosulfide solid solution, hz = heazlewoodite (Ni_3S_2), vs = vaesite (NiS_2), viol = violarite ($FeNi_2S_4$), a = kamacite, and y = taenite.

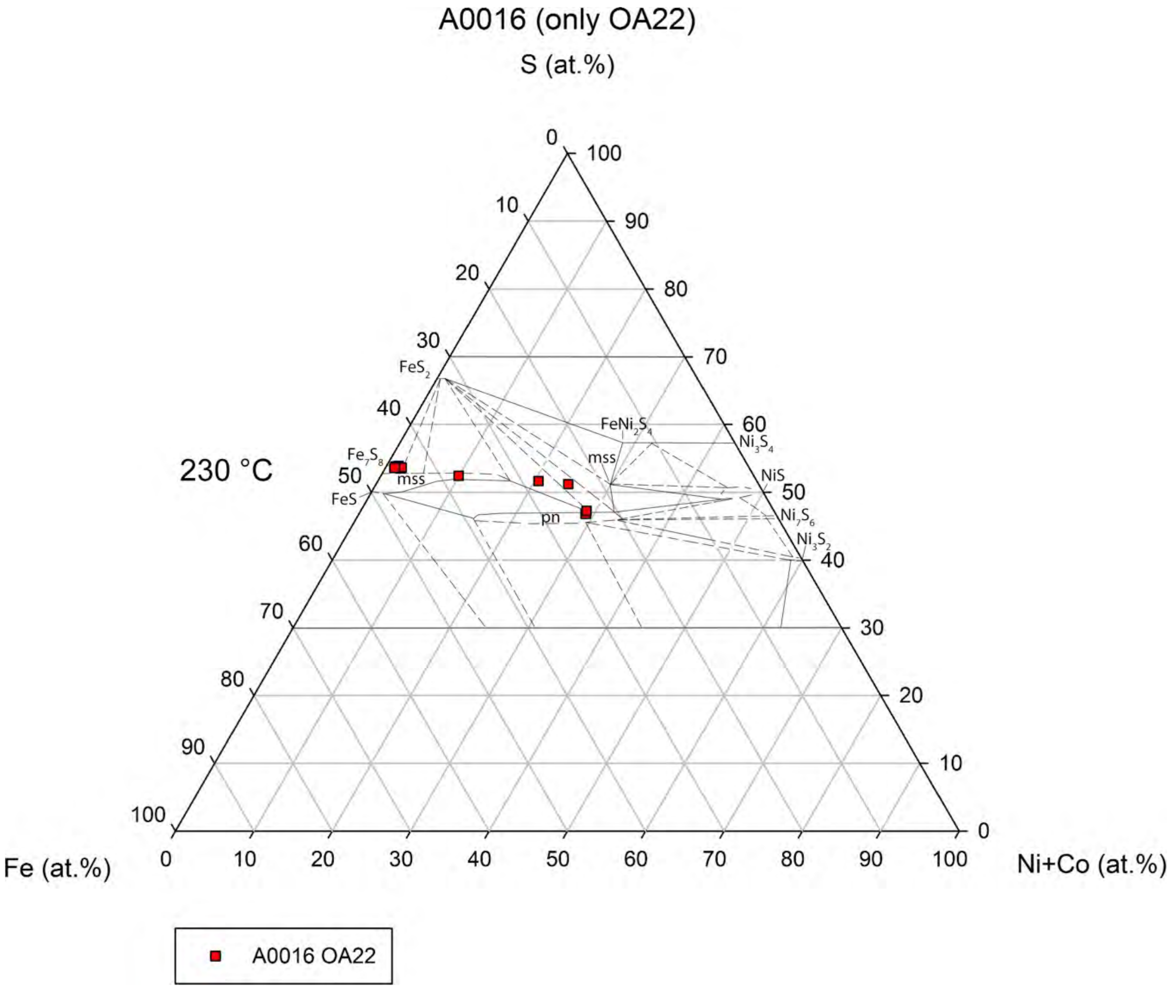

Supplementary Figure 158. In situ sulfide compositional data obtained via electron probe microanalyzer for A0016 OA22, superimposed on at.% Fe-Ni+Co-S phase diagram at 230 °C (phase diagram adapted from [4]). OA = opaque assemblage. Where po = pyrrhotite, pn = pentlandite, py = pyrite, mss = monosulfide solid solution, hz = heazlewoodite (Ni_3S_2), vs = vaesite (NiS_2), viol = violarite ($FeNi_2S_4$), a = kamacite, and y = taenite.

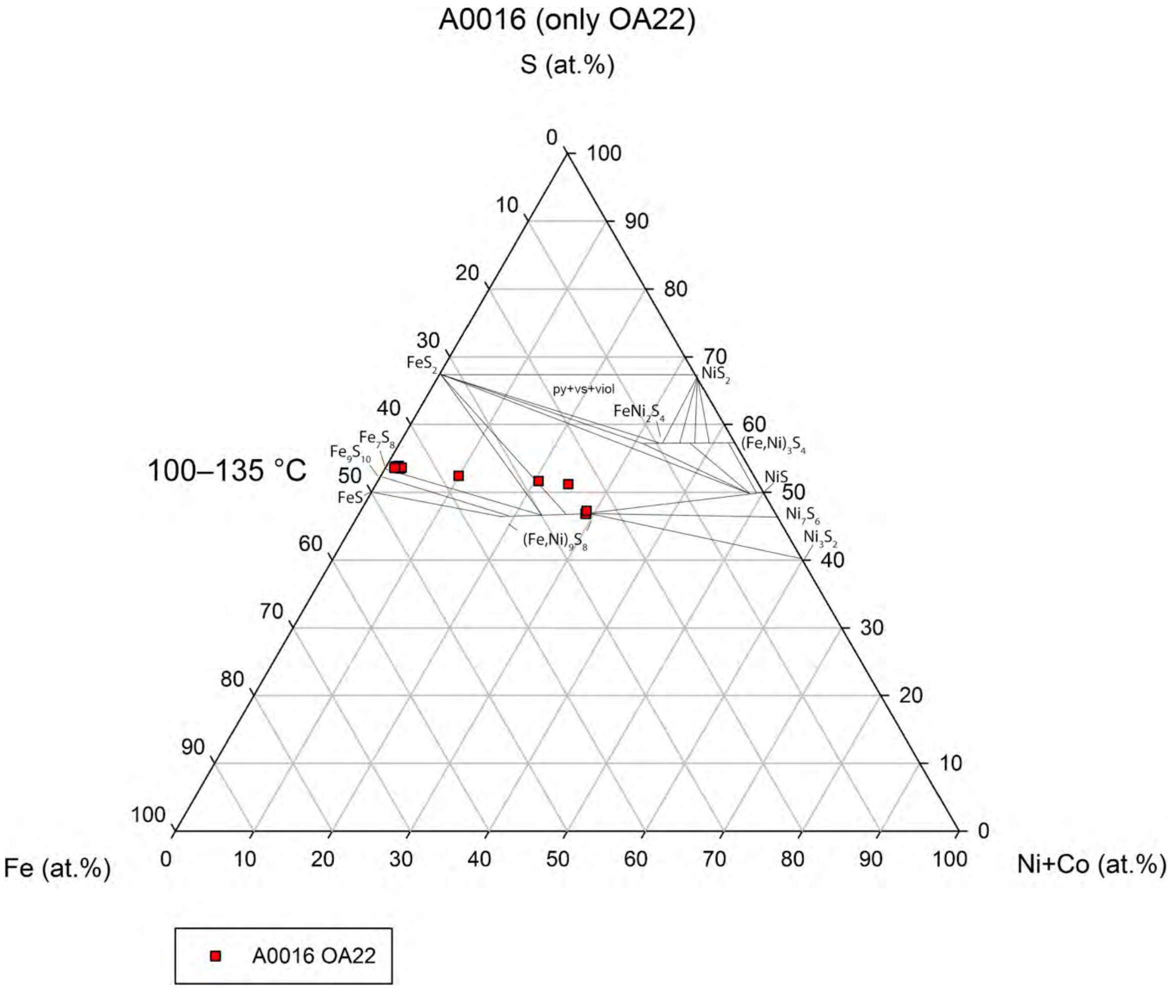

Supplementary Figure 159. In situ sulfide compositional data obtained via electron probe microanalyzer for A0016 OA22, superimposed on at.% Fe-Ni+Co-S phase diagram at 100–135 °C (phase diagram adapted from [5]). OA = opaque assemblage. Where po = pyrrhotite, pn = pentlandite, py = pyrite, mss = monosulfide solid solution, hz = heazlewoodite (Ni_3S_2), vs = vaesite (NiS_2), viol = violarite ($FeNi_2S_4$), a = kamacite, and y = taenite.

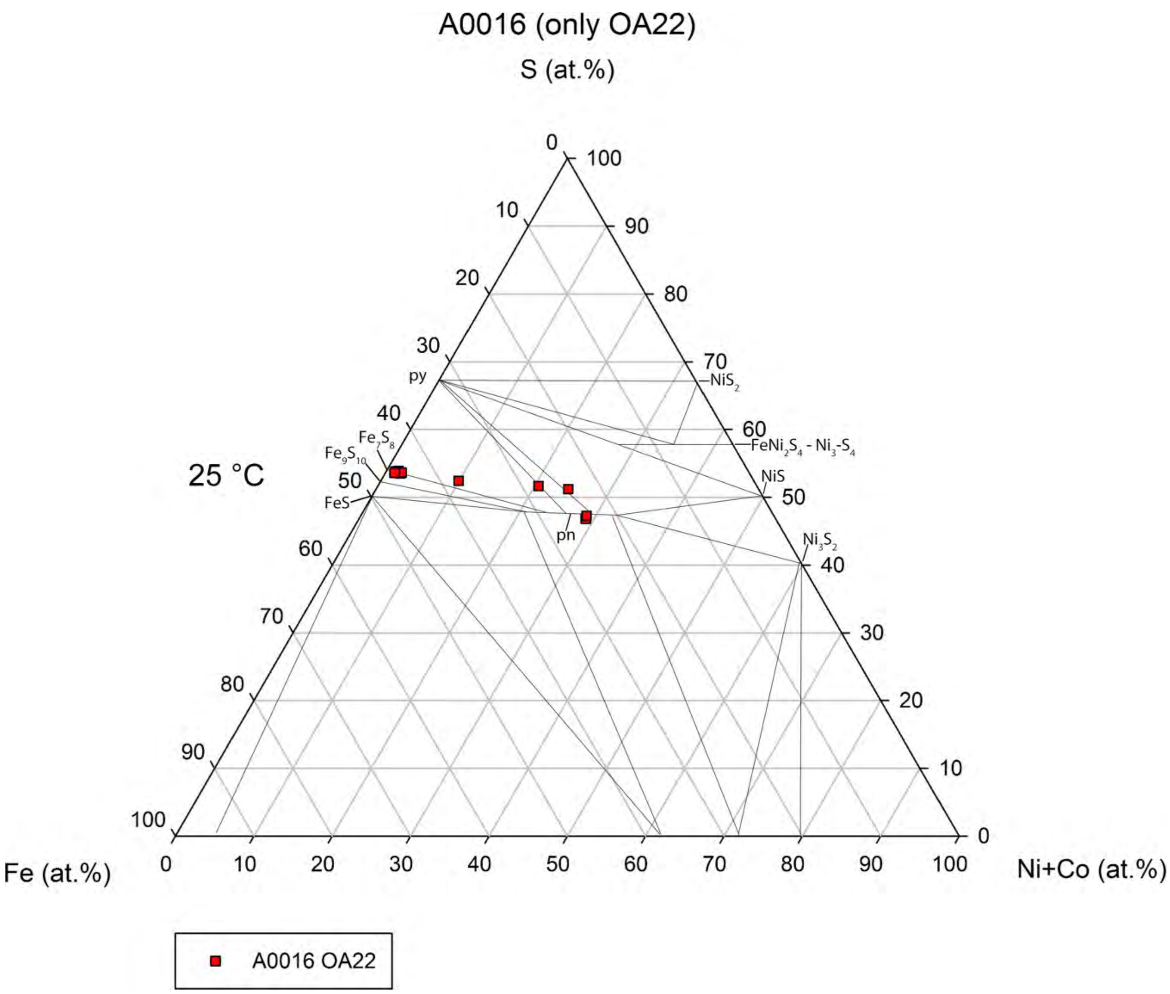

Supplementary Figure 160. In situ sulfide compositional data obtained via electron probe microanalyzer for A0016 OA22, superimposed on at.% Fe-Ni+Co-S phase diagram at 25 °C (phase diagram adapted from [6]). OA = opaque assemblage. Where po = pyrrhotite, pn = pentlandite, py = pyrite, mss = monosulfide solid solution, hz = heazlewoodite (Ni_3S_2), vs = vaesite (NiS_2), viol = violarite ($FeNi_2S_4$), a = kamacite, and y = taenite.

A0094-01 and C0103-01

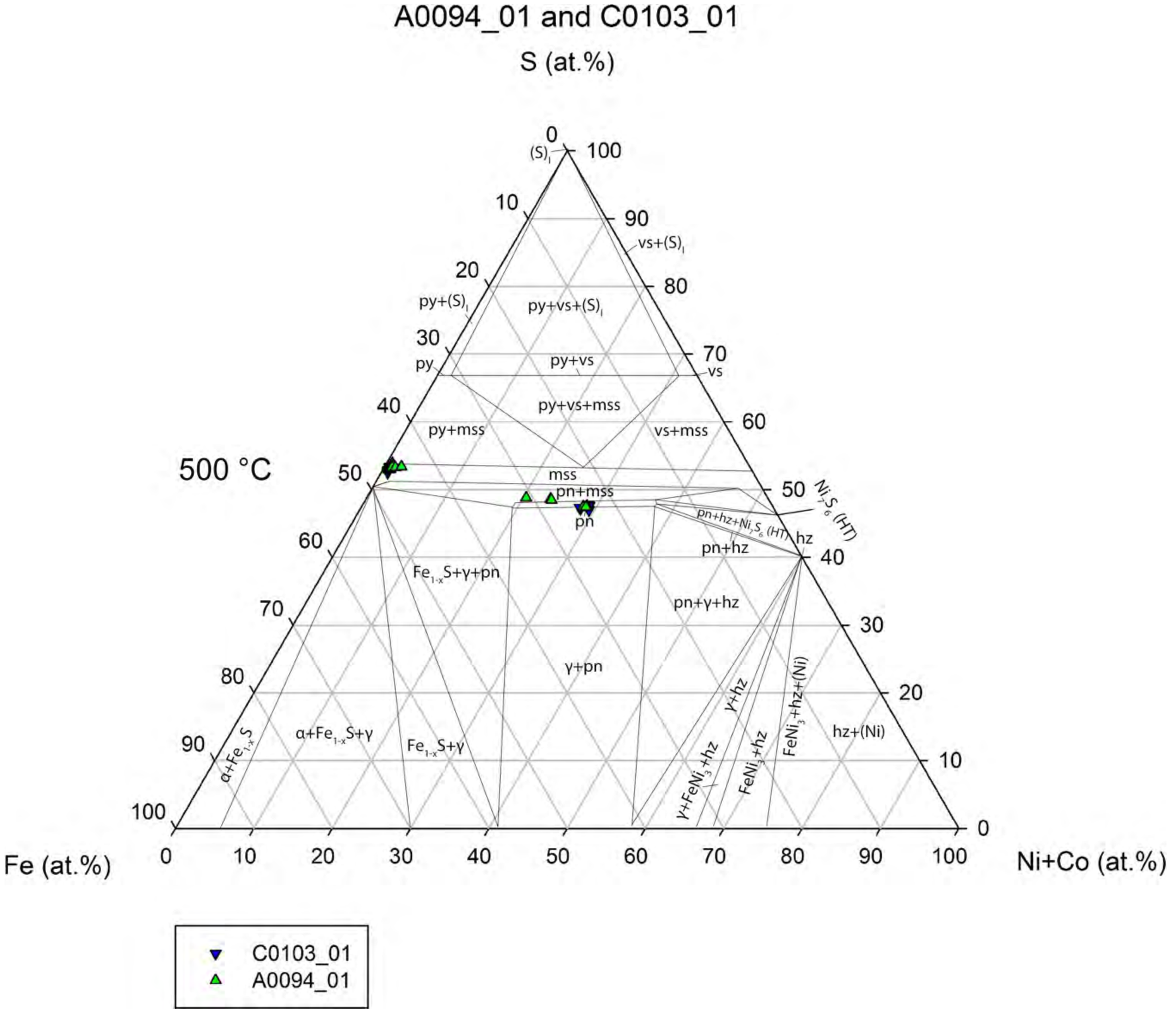

Supplementary Figure 161. In situ sulfide compositional data obtained via electron probe microanalyzer for A0094-01 and C0103-01, superimposed on at.% Fe-Ni+Co-S phase diagram at 500 °C (phase diagram adapted from [1,2]). Where po = pyrrhotite, pn = pentlandite, py = pyrite, mss = monosulfide solid solution, hz = heazlewoodite (Ni_3S_2), vs = vaesite (NiS_2), viol = violarite ($FeNi_2S_4$), a = kamacite, and y = taenite.

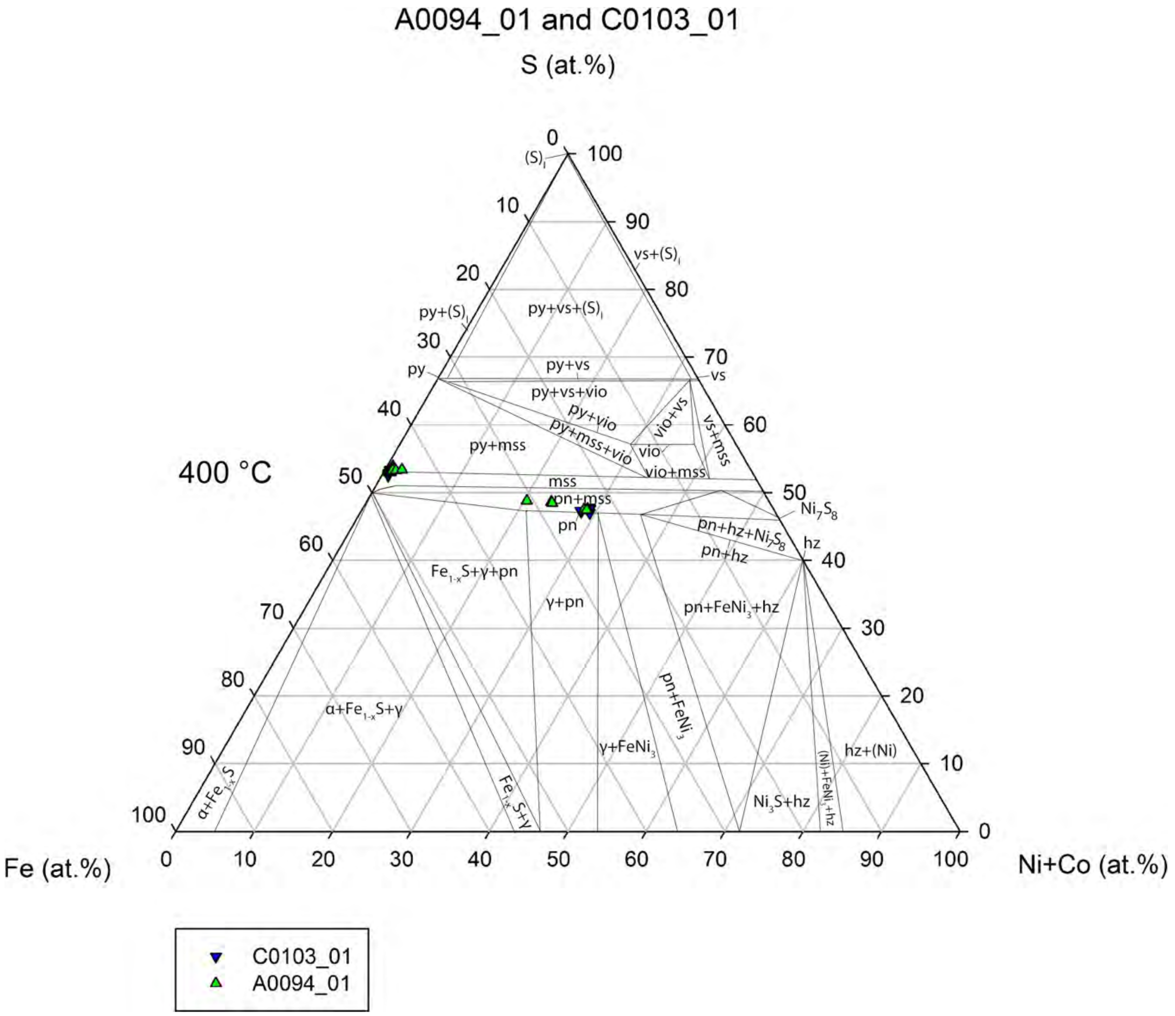

Supplementary Figure 162. In situ sulfide compositional data obtained via electron probe microanalyzer for A0094-01 and C0103-01, superimposed on at.% Fe-Ni+Co-S phase diagram at 400 °C (phase diagram adapted from [2,3]). Where po = pyrrhotite, pn = pentlandite, py = pyrite, mss = monosulfide solid solution, hz = heazlewoodite (Ni_3S_2), vs = vaesite (NiS_2), viol = violarite ($FeNi_2S_4$), a = kamacite, and y = taenite.

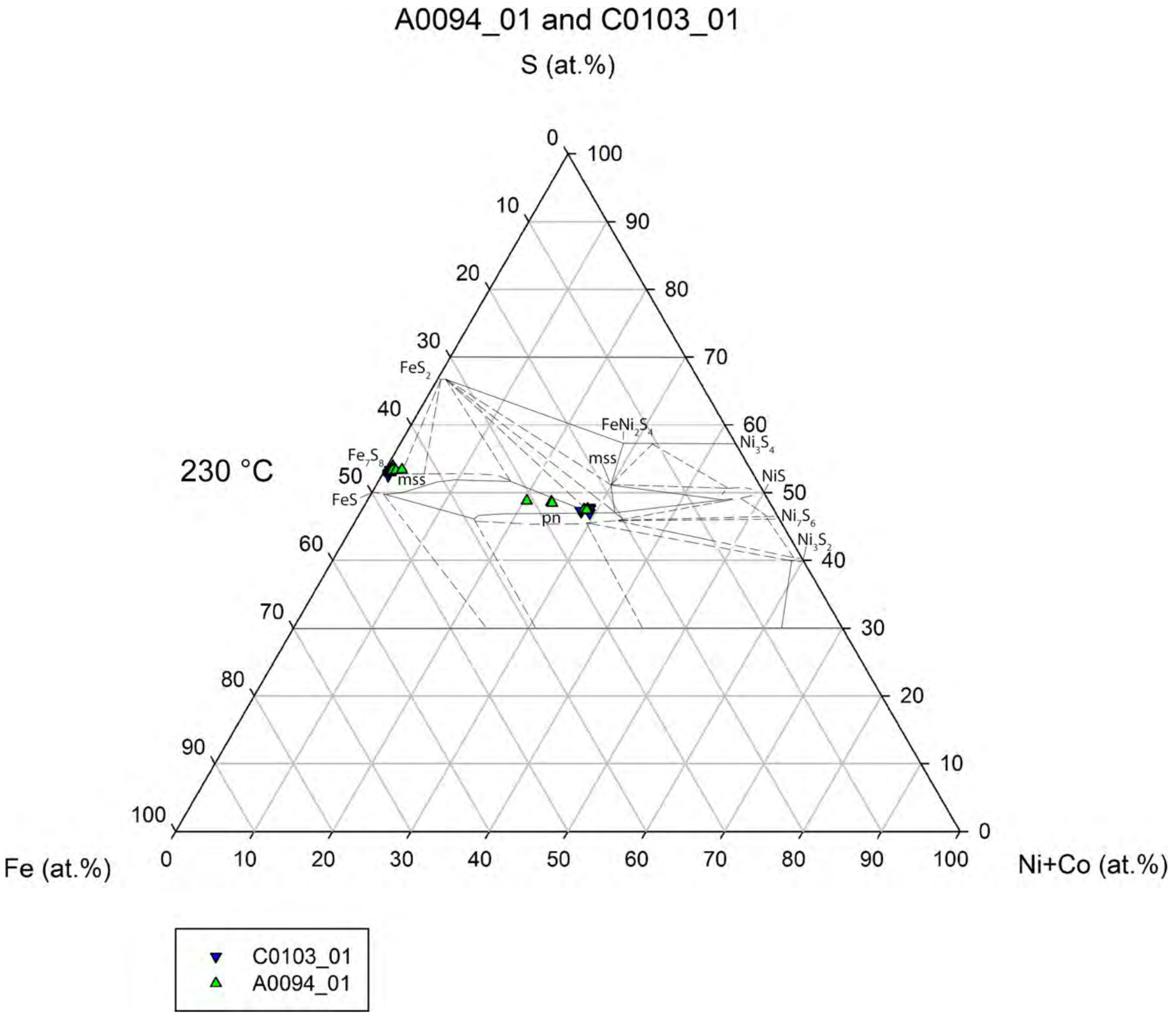

Supplementary Figure 163. In situ sulfide compositional data obtained via electron probe microanalyzer for A0094-01 and C0103-01, superimposed on at.% Fe-Ni+Co-S phase diagram at 230 °C (phase diagram adapted from [4]). Where po = pyrrhotite, pn = pentlandite, py = pyrite, mss = monosulfide solid solution, hz = heazlewoodite (Ni_3S_2), vs = vaesite (NiS_2), viol = violarite ($FeNi_2S_4$), a = kamacite, and y = taenite.

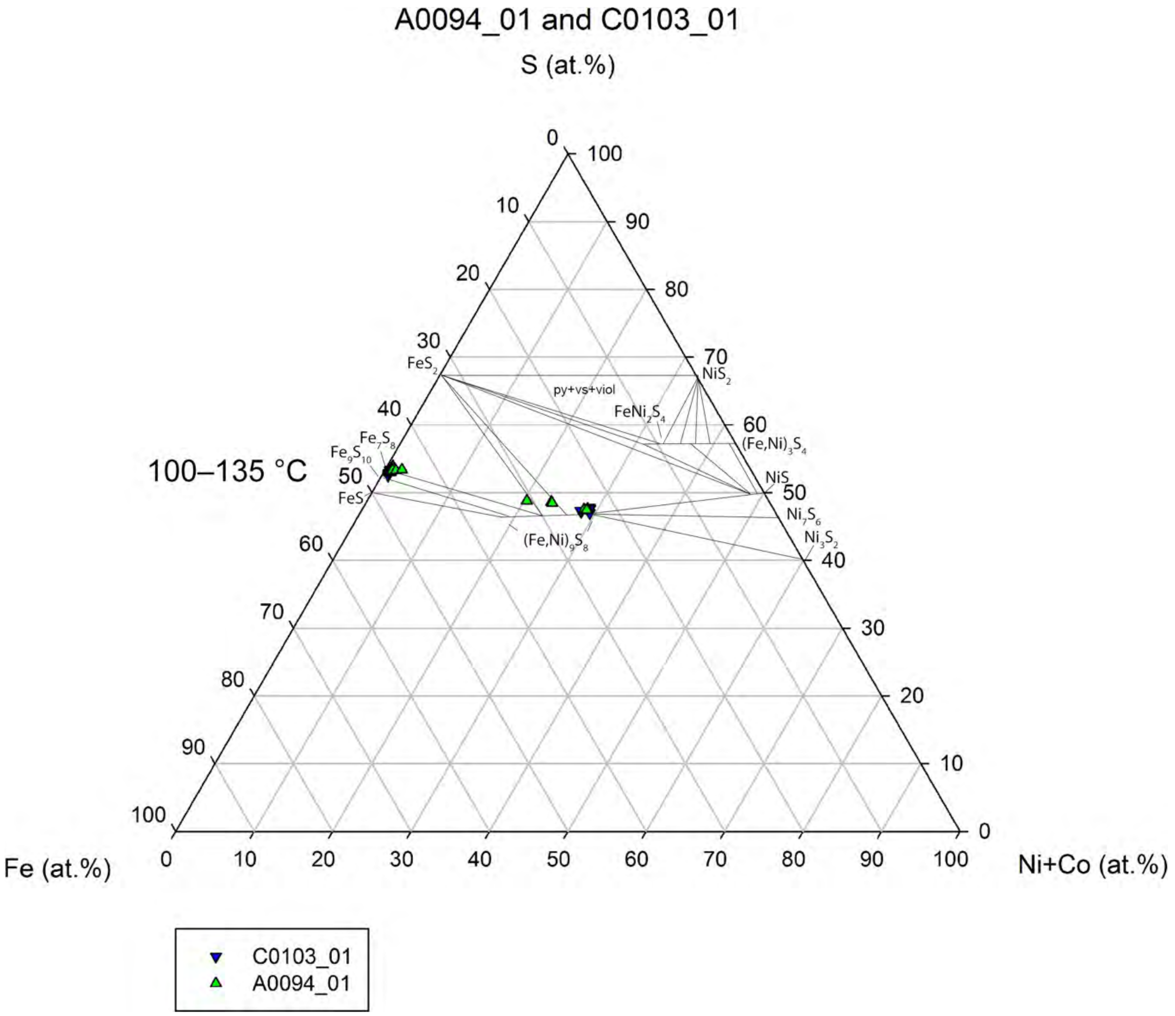

Supplementary Figure 164. In situ sulfide compositional data obtained via electron probe microanalyzer for A0094-01 and C0103-01, superimposed on at.% Fe-Ni+Co-S phase diagram at 100–135 °C (phase diagram adapted from [5]). Where po = pyrrhotite, pn = pentlandite, py = pyrite, mss = monosulfide solid solution, hz = heazlewoodite (Ni_3S_2), vs = vaesite (NiS_2), viol = violarite ($FeNi_2S_4$), a = kamacite, and y = taenite.

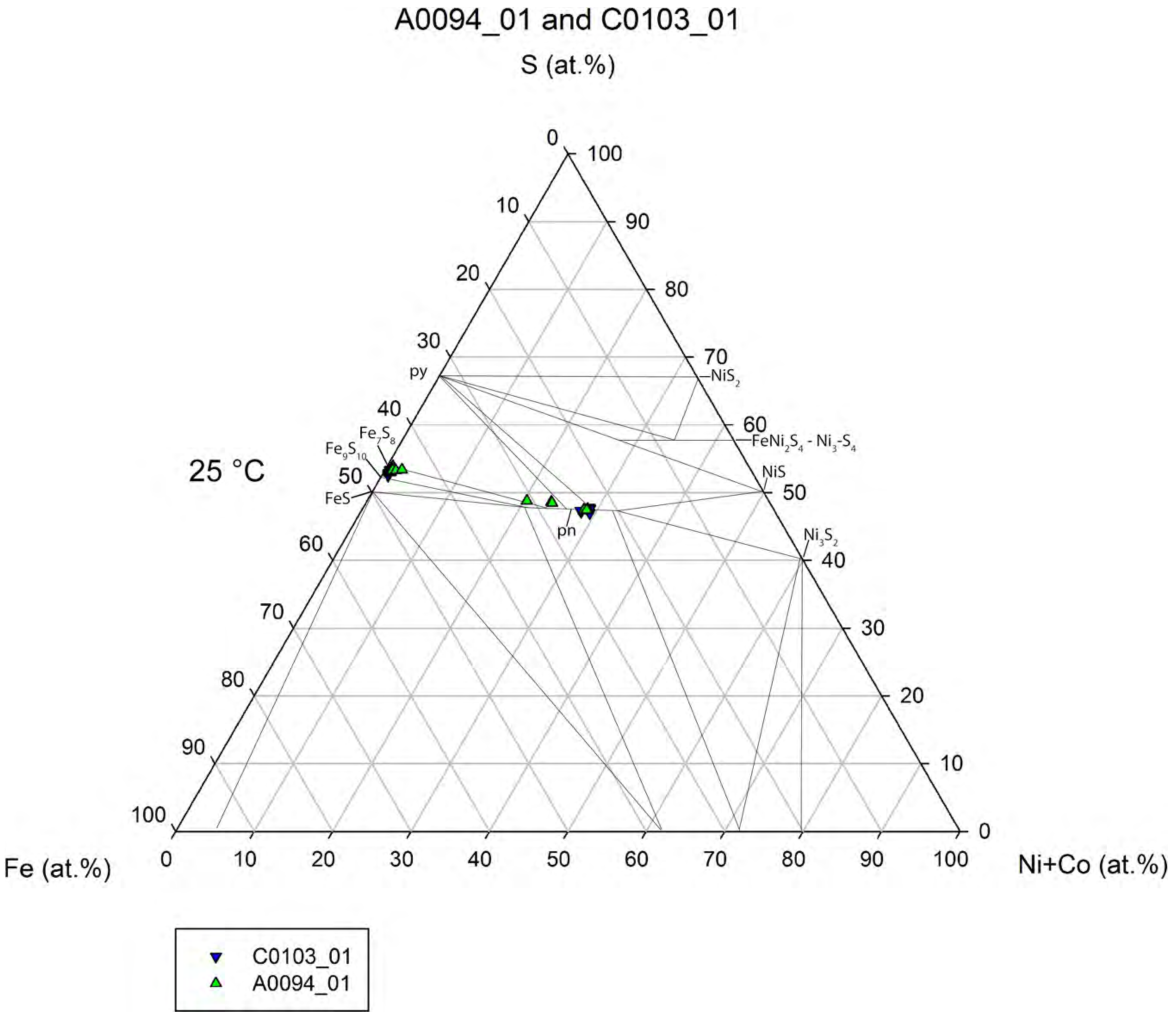

Supplementary Figure 165. In situ sulfide compositional data obtained via electron probe microanalyzer for A0094-01 and C0103-01, superimposed on at.% Fe-Ni+Co-S phase diagram at 25 °C (phase diagram adapted from [6]). Where po = pyrrhotite, pn = pentlandite, py = pyrite, mss = monosulfide solid solution, hz = heazlewoodite (Ni_3S_2), vs = vaesite (NiS_2), viol = violarite ($FeNi_2S_4$), a = kamacite, and y = taenite.

A0094-01

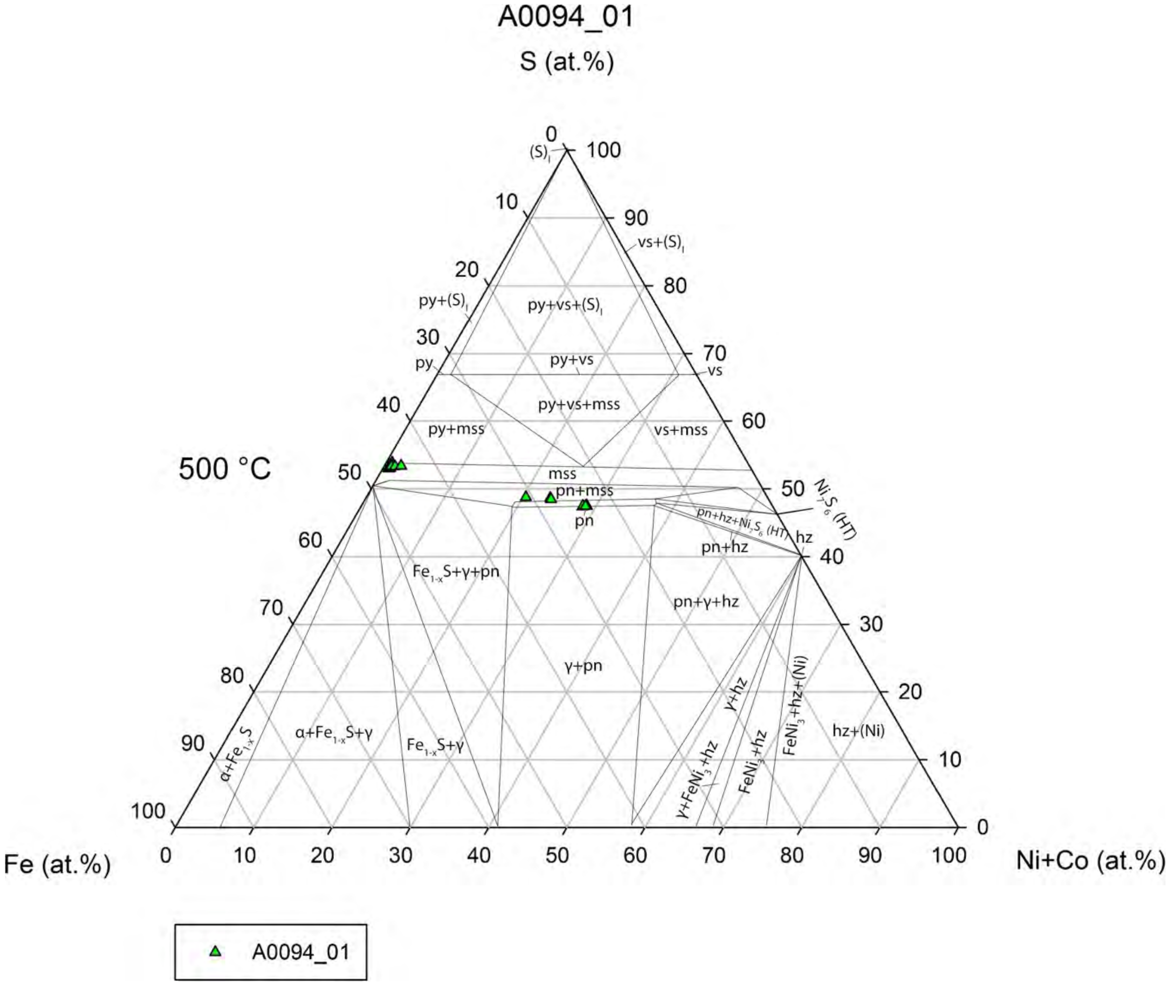

Supplementary Figure 166. In situ sulfide compositional data obtained via electron probe microanalyzer for A0094-01, superimposed on at.% Fe-Ni+Co-S phase diagram at 500 °C (phase diagram adapted from [1,2]). Where po = pyrrhotite, pn = pentlandite, py = pyrite, mss = monosulfide solid solution, hz = heazlewoodite (Ni_3S_2), vs = vaesite (NiS_2), viol = violarite ($FeNi_2S_4$), a = kamacite, and y = taenite.

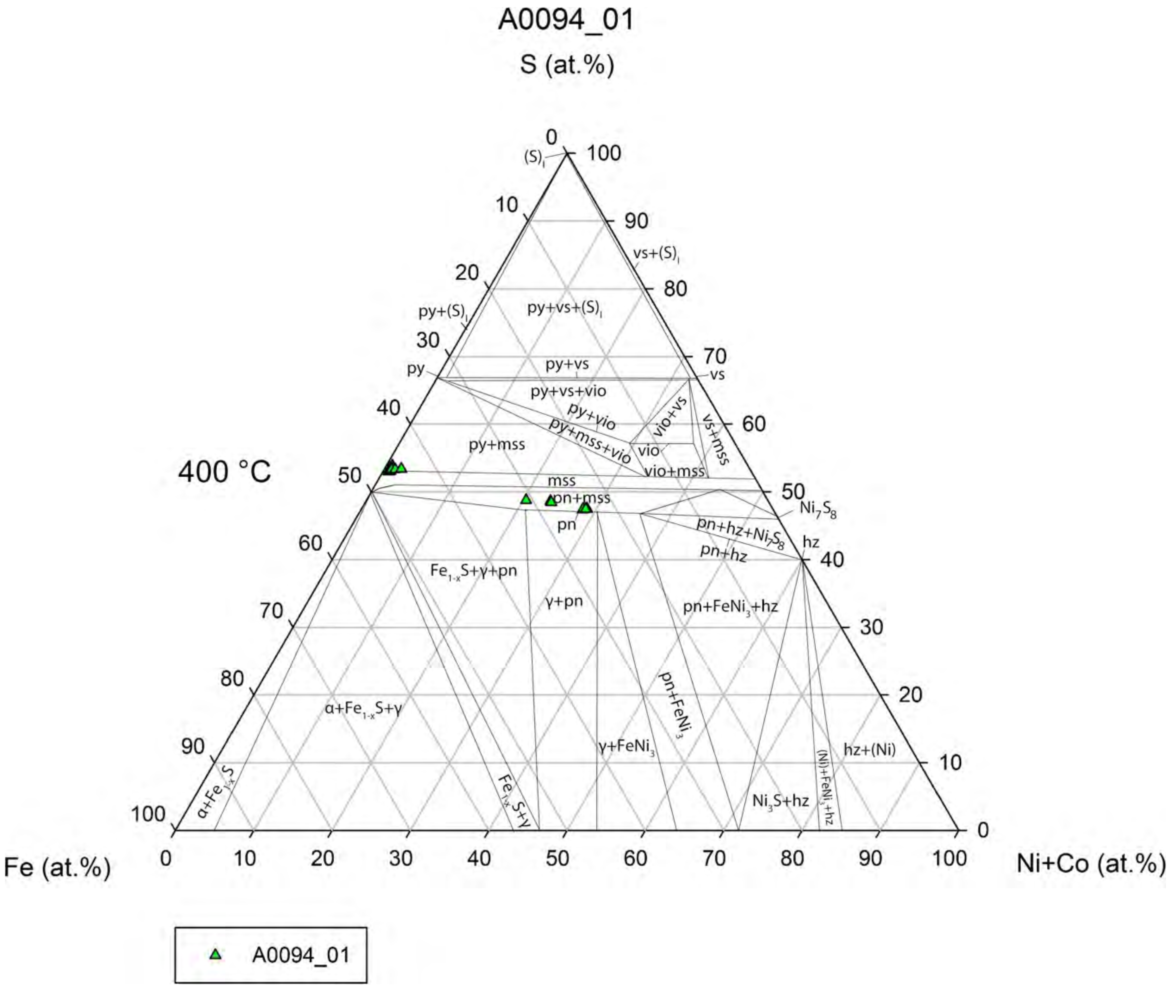

Supplementary Figure 167. In situ sulfide compositional data obtained via electron probe microanalyzer for A0094-01, superimposed on at.% Fe-Ni+Co-S phase diagram at 400 °C (phase diagram adapted from [2,3]). Where po = pyrrhotite, pn = pentlandite, py = pyrite, mss = monosulfide solid solution, hz = heazlewoodite (Ni_3S_2), vs = vaesite (NiS_2), viol = violarite ($FeNi_2S_4$), a = kamacite, and y = taenite.

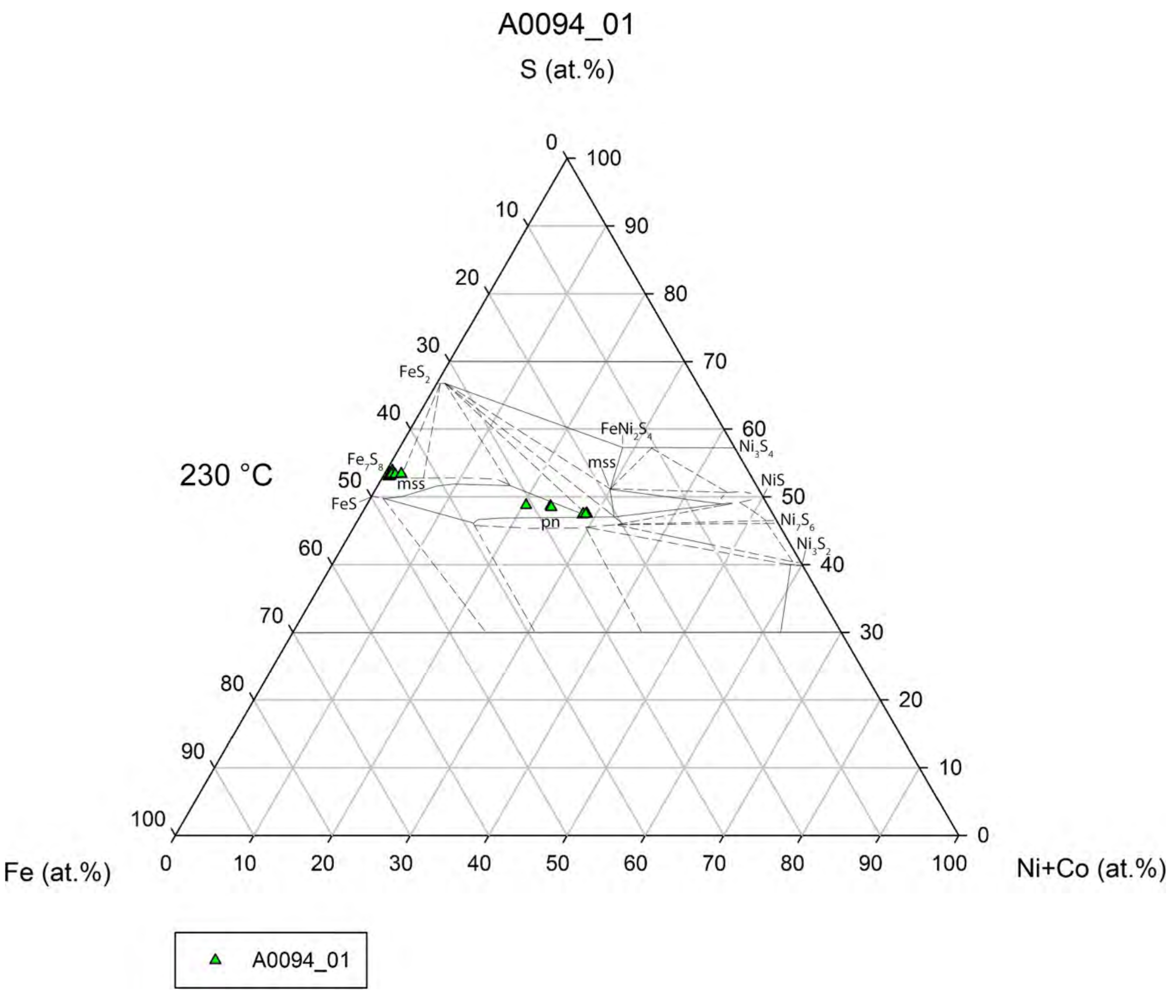

Supplementary Figure 168. In situ sulfide compositional data obtained via electron probe microanalyzer for A0094-01, superimposed on at.% Fe-Ni+Co-S phase diagram at 230 °C (phase diagram adapted from [4]). Where po = pyrrhotite, pn = pentlandite, py = pyrite, mss = monosulfide solid solution, hz = heazlewoodite (Ni_3S_2), vs = vaesite (NiS_2), viol = violarite ($FeNi_2S_4$), a = kamacite, and y = taenite.

A0094_01

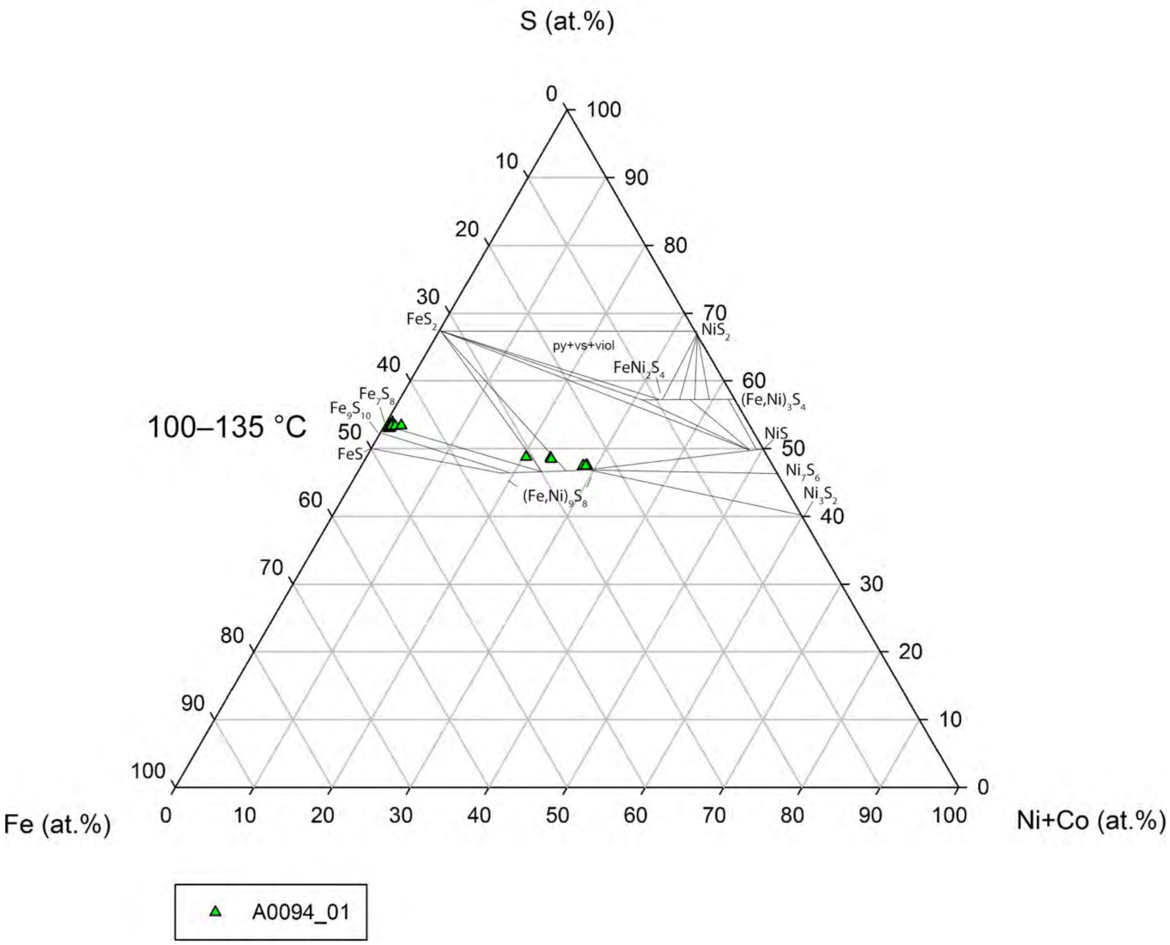

Supplementary Figure 169. In situ sulfide compositional data obtained via electron probe microanalyzer for A0094-01, superimposed on at.% Fe-Ni+Co-S phase diagram at 100–135 °C (phase diagram adapted from [5]). Where po = pyrrhotite, pn = pentlandite, py = pyrite, mss = monosulfide solid solution, hz = heazlewoodite (Ni_3S_2), vs = vaesite (NiS_2), viol = violarite ($FeNi_2S_4$), a = kamacite, and y = taenite.

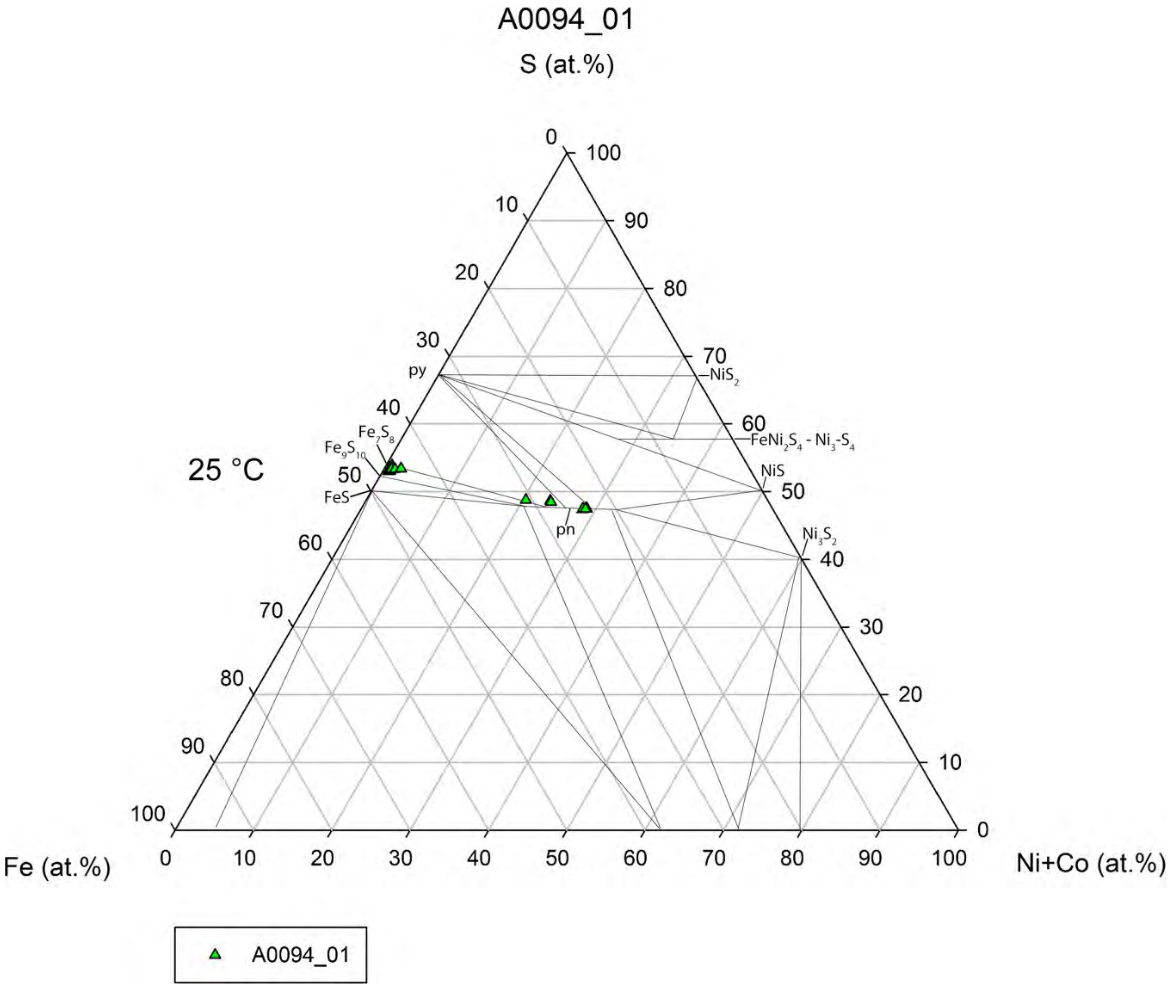

Supplementary Figure 170. In situ sulfide compositional data obtained via electron probe microanalyzer for A0094-01, superimposed on at.% Fe-Ni+Co-S phase diagram at 25 °C (phase diagram adapted from [6]). Where po = pyrrhotite, pn = pentlandite, py = pyrite, mss = monosulfide solid solution, hz = heazlewoodite (Ni_3S_2), vs = vaesite (NiS_2), viol = violarite ($FeNi_2S_4$), a = kamacite, and y = taenite.

C0103-01

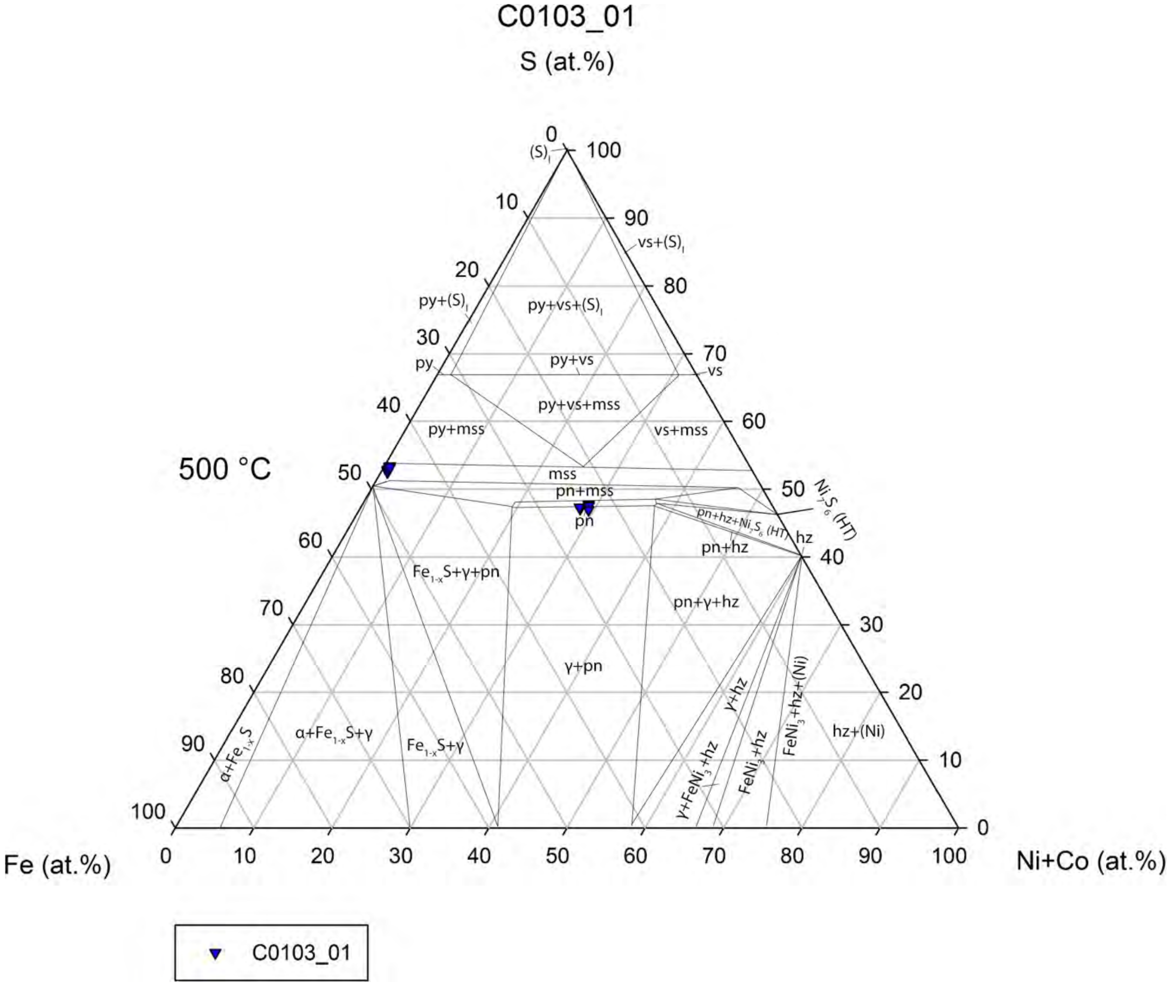

Supplementary Figure 171. In situ sulfide compositional data obtained via electron probe microanalyzer for C0103-01, superimposed on at.% Fe-Ni+Co-S phase diagram at 500 °C (phase diagram adapted from [1,2]). Where po = pyrrhotite, pn = pentlandite, py = pyrite, mss = monosulfide solid solution, hz = heazlewoodite (Ni_3S_2), vs = vaesite (NiS_2), viol = violarite ($FeNi_2S_4$), a = kamacite, and y = taenite.

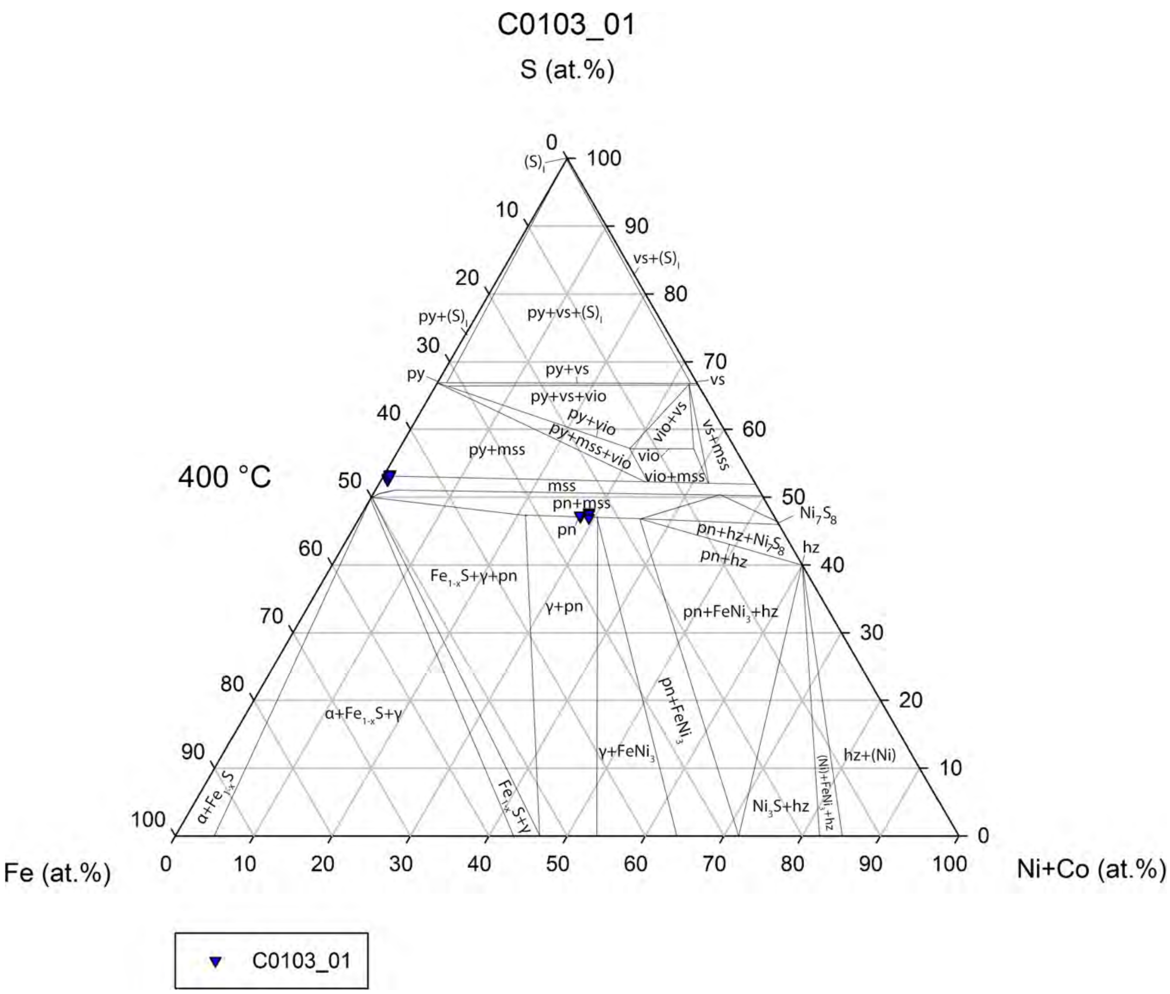

Supplementary Figure 172. In situ sulfide compositional data obtained via electron probe microanalyzer for C0103-01, superimposed on at.% Fe-Ni+Co-S phase diagram at 400 °C (phase diagram adapted from [2,3]). Where po = pyrrhotite, pn = pentlandite, py = pyrite, mss = monosulfide solid solution, hz = heazlewoodite (Ni_3S_2), vs = vaesite (NiS_2), viol = violarite ($FeNi_2S_4$), a = kamacite, and y = taenite.

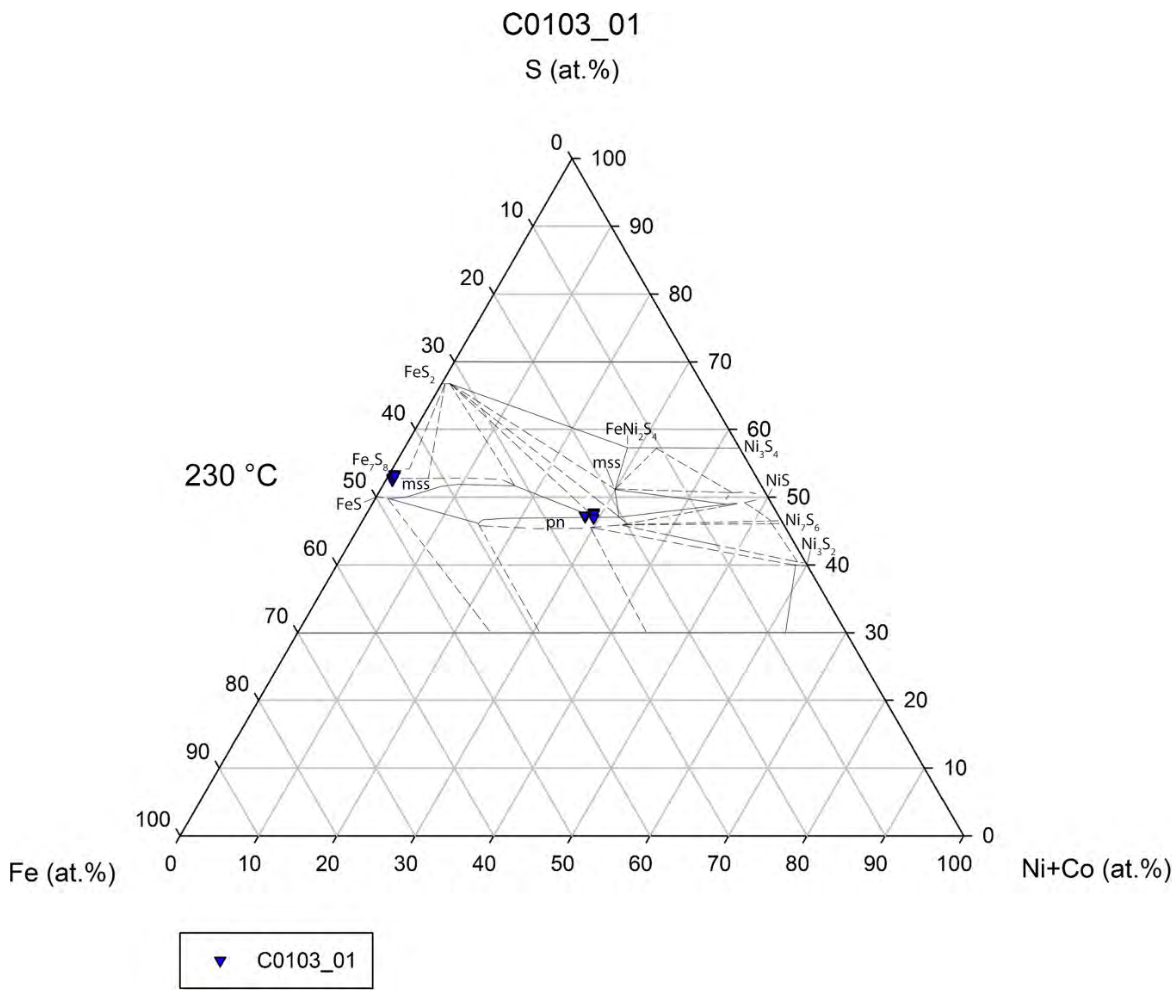

Supplementary Figure 173. In situ sulfide compositional data obtained via electron probe microanalyzer for C0103-01, superimposed on at.% Fe-Ni+Co-S phase diagram at 230 °C (phase diagram adapted from [4]). Where po = pyrrhotite, pn = pentlandite, py = pyrite, mss = monosulfide solid solution, hz = heazlewoodite (Ni_3S_2), vs = vaesite (NiS_2), viol = violarite ($FeNi_2S_4$), a = kamacite, and y = taenite.

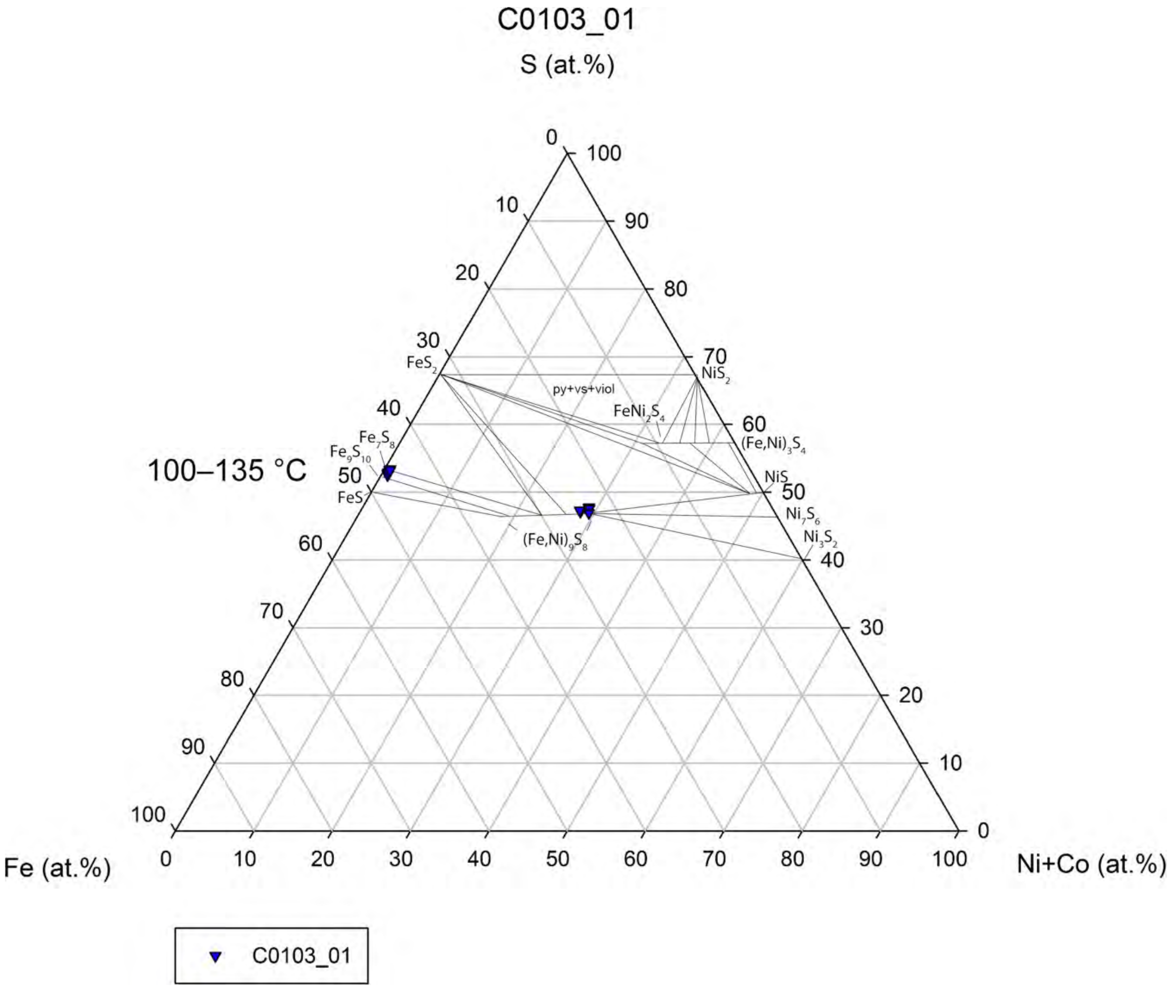

Supplementary Figure 174. In situ sulfide compositional data obtained via electron probe microanalyzer for C0103-01, superimposed on at.% Fe-Ni+Co-S phase diagram at 100–135 °C (phase diagram adapted from [5]). Where po = pyrrhotite, pn = pentlandite, py = pyrite, mss = monosulfide solid solution, hz = heazlewoodite (Ni_3S_2), vs = vaesite (NiS_2), viol = violarite ($FeNi_2S_4$), a = kamacite, and y = taenite.

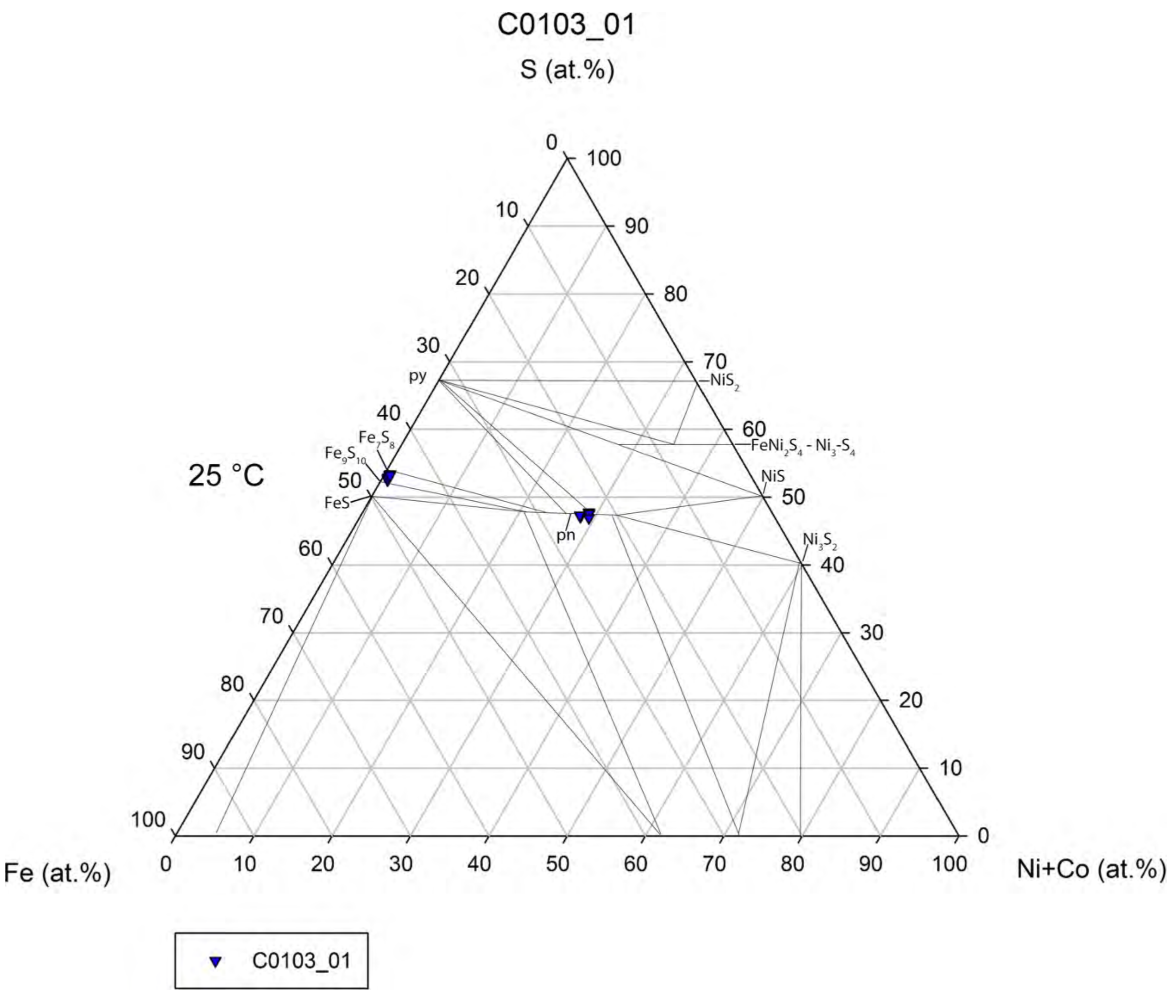

Supplementary Figure 175. In situ sulfide compositional data obtained via electron probe microanalyzer for C0103-01, superimposed on at.% Fe-Ni+Co-S phase diagram at 25 °C (phase diagram adapted from [6]). Where po = pyrrhotite, pn = pentlandite, py = pyrite, mss = monosulfide solid solution, hz = heazlewoodite (Ni_3S_2), vs = vaesite (NiS_2), viol = violarite ($FeNi_2S_4$), a = kamacite, and y = taenite.

Supplementary References

[1] Shewman, R.W., and Clark, L.A. Pentlandite Phase Relations in the Fe-Ni-S System and Notes on the Monosulfide Solid Solution. *Canadian Journal of Earth Sciences* **7**, 67–85 (1970).

[2] Raghavan, V. Fe–Ni–S (iron–nickel–sulfur). *Journal of Phase Equilibria and Diffusion* **25**, 373– 381 (2004).

[3] Craig, J.R., Naldrett, A.J., and Kullerud, G. The Fe-Ni-S system: 400 °C isothermal diagram. *Carnegie Institution of Washington Year Book* **66**, 440–441 (1968).

[4] Misra, K.C., and Fleet, M.E. The chemical compositions of synthetic and natural pentlandite assemblages. *Economic Geology* **68**, 518–539 (1973).

[5] Naldrett, A.J. Magmatic Sulphide Deposits. *Oxford University Press*, Oxford (1989).

[6] Vaughan, D.J., and Craig, J.R. Sulfide ore mineral stabilities, morphologies, and intergrowth textures. In *Geochemistry of Hydrothermal Ore Deposits, Third Edition (ed. H. L. Barnes)*. John Wiley and Sons, New York. pp. 367–434 (1997).
